# Asymmetry in the *Q*_y_ Fluorescence and Absorption Spectra of Chlorophyll *a* Pertaining to Exciton Dynamics

**DOI:** 10.3389/fchem.2020.588289

**Published:** 2020-12-02

**Authors:** Jeffrey R. Reimers, Margus Rätsep, Arvi Freiberg

**Affiliations:** ^1^School of Chemistry, The University of Sydney, Sydney, NSW, Australia; ^2^Institute of Physics, University of Tartu, Tartu, Estonia; ^3^Institute of Molecular and Cell Biology, University of Tartu, Tartu, Estonia

**Keywords:** absorption-emission asymmetry, differential fluorescence line narrowing, coherent energy redistribution, exciton transport, photosynthesis, density functional theory, Duschinsky rotation

## Abstract

Significant asymmetry found between the high-resolution *Q*_y_ emission and absorption spectra of chlorophyll-a is herein explained, providing basic information needed to understand photosynthetic exciton transport and photochemical reactions. The *Q*_y_ spectral asymmetry in chlorophyll has previously been masked by interference in absorption from the nearby *Q*_x_ transition, but this effect has recently been removed using extensive quantum spectral simulations or else by analytical inversion of absorption and magnetic circular dichroism data, allowing high-resolution absorption information to be accurately determined from fluorescence-excitation spectra. To compliment this, here, we measure and thoroughly analyze the high-resolution differential fluorescence line narrowing spectra of chlorophyll-a in trimethylamine and in 1-propanol. The results show that vibrational frequencies often change little between absorption and emission, yet large changes in line intensities are found, this effect also being strongly solvent dependent. Among other effects, the analysis in terms of four basic patterns of Duschinsky-rotation matrix elements, obtained using CAM-B3LYP calculations, predicts that a chlorophyll-a molecule excited into a specific vibrational level, may, without phase loss or energy relaxation, reemit the light over a spectral bandwidth exceeding 1,000 cm^−1^ (0.13 eV) to influence exciton-transport dynamics.

## Introduction

Chlorophyll-a (Chl-a) is the most common chlorophyllide utilized in natural photosynthesis (van Grondelle et al., 1994; Blankenship et al., 2004; Grimm et al., 2006; Laisk et al., 2009). For all processes in which this molecule is involved, including light capture, coherent and incoherent exciton transport, and charge transport, the dissipation of energy (the *reorganization energy* λ) through intramolecular nuclear relaxation is a critical process (Hush, 1958; May and Kühn, 2008). Critical for exciton transport are both the ground-state reorganization energy λ^*E*^ and excited-state reorganization energy λ^*A*^, which can be measured and partitioned into individual vibrational components using modern high-resolution techniques (Rebane and Avarmaa, 1982; Avarmaa and Rebane, 1985; Jaaniso and Avarmaa, 1986; Renge et al., 1987; Sild and Haller, 1988; Gillie et al., 1989; den Hartog et al., 1998; Rätsep et al., 1998, 2009a, 2011, 2019b; Zazubovich et al., 2001; Rätsep and Freiberg, 2003; Hughes et al., 2004; Purchase and Völker, 2009; Jankowiak et al., 2011; Pieper and Freiberg, 2014; Adolphs et al., 2016; Leiger et al., 2017). For excited-state properties, hole-burning techniques are commonly applied, but there are still ambiguities in the baseline limit quantitative analysis, with fluorescence-excitation being a more robust technique provided that the contribution from the zero-phonon line (ZPL) can be determined by other means (Reimers et al., 2013; Pieper et al., 2018). For ground-state vibrations, only relatively recently has the differential fluorescence line-narrowing techniques (ΔFLN) (Rätsep and Freiberg, 2003, 2007; Rätsep et al., 2009a, 2019a) been developed to deliver similar results. These techniques provide critical vibrational data required for studies of, e.g., coherent energy transport in photosystems (Renger et al., 1996; Huo and Coker, 2010; Rivera et al., 2013; Kreisbeck et al., 2014; Müh et al., 2014; Romero et al., 2014; Malý et al., 2016; Duan et al., 2017; Ren et al., 2018; Cao et al., 2020; Tomasi and Kassal, 2020).

It is very convenient in quantum dynamics calculations of energy and/or electron transport to assume that the reorganization energy has the same value and the same distribution amongst normal modes on both the ground and excited state (Rebane, 1970). In this situation, both low-resolution and high-resolution absorption and emission spectra are symmetric, i.e., after appropriate normalization and reflection of (say) the emission spectrum about its origin, the two spectra are identical. The observed low-resolution spectra for Chl-a (Rätsep et al., 2009a), bacteriochlorophyll-a (BChl-a) (Rätsep et al., 2011), and pheophytin–a (Pheo-a) (Rätsep et al., 2019b) are significantly asymmetric. Various explanations of this could be envisaged suggestive that quantum dynamics calculations preformed assuming symmetry should give realistic results: the vibrational frequency of some key modes could be different in the ground and excited states, or the most intense lines in absorption and emission could be of the same nature, with just their intensities modulated. Nevertheless, the observation of high-resolution data showed that such scenarios do not apply: many of the intense modes in either absorption or emission are just not seen at all in the other spectrum (Rätsep et al., 2011, 2019b), suggesting the possibility of serious failure for symmetry-based quantum dynamics models for exciton transfer. As before, only through the measurement of both high-resolution absorption and emission spectra can the implications of this for quantum dynamics be made apparent. Of important note, the asymmetry is shown to be environment-dependent (Rätsep et al., 2011, 2019b), allowing, in principle, metal chelation and changes to nearby residues and solvent location to modulate transport in a protein environment.

For Chl-a, BChl-a, and Pheo-a, the lowest-energy excited state is *Q*_*y*_ and the next state is *Q*_*x*_ (Gouterman et al., 1963). For Chl-a, a critical qualitative feature contributing to absorption-emission asymmetry is that the *Q*_*x*_–*Q*_*y*_ energy spacing is small, resulting in overlapping absorptions. Indeed, the nature of *Q*_x_ and its spectral impact had been debated for over 50 years, with all issues being resolved in 2013 using a model involving strong resonant non-adiabatic coupling mixing the natures of the *Q*_*x*_ and *Q*_*y*_ states (Reimers et al., 2013). Non-adiabatic coupling splits the absorption intensity of the *Q*_*x*_ state into two separate spectral regions, the lower-energy component centered around a strongly perturbed *Q*_*y*_ vibrational mode in the region of the *Q*_*y*_ origin plus 300–1,000 cm^−1^, with the higher-energy component placed 1,000–1,300 cm^−1^ further away. Before this assignment was established, it was hypothesized that just one of the two resolved component bands depicted *Q*_*x*_, leading to two competitive assignments based upon the chosen component. Neither of the assignments could account for all observed (and calculated) data, leading to the extended debate. That strong non-adiabatic coupling dominates the spectral properties is a rather unusual situation in molecular spectroscopy. As a result, no approach based on the Born-Oppenheimer approximation (Huang Rhys factors, Herzberg-Teller couplings, etc.) can account for the observed spectra properties. In particular, the *Q*_*x*_ origin transition energy, as perceived by quantum-chemical calculations, pertains to a region of *minimal* absorption *between* the two observed *x-*polarized spectral peaks. Nevertheless, the naïve assignment of *Q*_*x*_ to one of the two observed peaks still remains common practice (Sirohiwal et al., 2020), resulting in serious overestimation of the observed *Q*_*x*_–*Q*_*y*_ spacing.

Despite the complexity of observed absorption spectra owing to the strong *Q*_*x*_–*Q*_*y*_ non-adiabatic interaction, a simple interpretation of the observed spectral properties is still possible (Reimers et al., 2013). Details pertaining to the location, width, and intensity of each of the two *x-*polarized absorption components can be understood in terms of one parameter: the energy difference Δ*E* between the *Q*_*x*_ and *Q*_*y*_ states. This is controlled by the magnesium coordination, as well as external environmental influences (Reimers et al., 2013). In gas-phase complexes, and 5-coordinate solvents and most protein environments, the *x-*polarized absorption becomes hidden underneath the strong Chl-a Franck-Condon 0–1 vibrational sideband centered at the origin plus ~1,300 cm^−1^. However, in 6-coordinate environments, this component moves into a sparser spectral region at about half this spacing and, hence, often becomes clearly resolved.

Understanding the breakdown of the Born-Oppenheimer approximation for Chl-a is critical to any quantitative analysis pertaining to both low-resolution and high-resolution absorption spectra. It allows the observed (Avarmaa and Rebane, 1985) high-resolution fluorescence-excitation spectra to be separated into broad underlying contributions associated with *Q*_*x*_, as well as high-resolution features depicting the intrinsic nature of *Q*_*y*_ (Reimers et al., 2013). This has led to the first quantitative high-resolution description of *Q*_*y*_ absorption. Details of the non-adiabatic coupling between *Q*_*y*_ and *Q*_*x*_ in chlorophyllides have also been studied recently using two-dimensional spectroscopies (Bukarte et al., 2020), revealing properties critical to function. Ultrafast relaxation of light energy absorbed with *x*-polarization to energy emitted with *y-*polarization is controlled largely by Δ*E* (Reimers et al., 2013).

To investigate the absorption-emission asymmetry of Chl-a in high resolution, what therefore remains is the measurement and interpretation of its high-resolution emission spectrum. Herein, we measure and analyze the *Q*_*y*_ emission spectra of Chl-a using the ΔFLN technique in two different solvents: trimethylamine (TEA) and 1-propanol. The identified asymmetry in combination with low-resolution absorption-emission spectra is then interpreted based on the results of density-functional theory (DFT) simulation of the electronic and nuclear structures. A self-consistent set of spectroscopic approximations is used in both the experimental data analysis and in the interpretation of the DFT simulations.

All calculations reported in the main text are performed in the gas phase. Treatments of solvent through implicit and/or explicit methods can be very successful (Hush and Reimers, 2000; Tomasi, 2011; Mennucci, 2012; Skyner et al., 2015; Zuehlsdorff and Isborn, 2018; Caricato, 2019; Cerezo et al., 2020), but no such approach has been demonstrated as yet to be successful for understanding chlorophyll bandshapes. Indeed, results presented in Supplementary Table 1 using commonly applied solvation treatments fail to provide qualitatively useful information, as has been previously noted for bacteriochlorophyll-a (Higashi et al., 2014). Spectra of Chl-a complexes have been recorded in the gas phase (Shafizadeh et al., 2011; Kjær et al., 2020), but as yet no high-resolution information is available. We demonstrate that use of gas-phase calculations allows for the key qualitative features that control absorption-emission asymmetry in Chl-a to be identified. In this process, a map is created that shows how the most important high-resolution vibrational lines observed in absorption-type experiments relate to those observed in emission-type experiments.

## Methods

### Sample Preparation and Spectral Measurements

Chl-a powder purchased from Sigma-Aldrich was stored in the dark at −18°C before dissolving in high-grade solvents of 1-propanol and TEA. Plastic (polymethyl methacrylate) cuvettes of 5 mm path length were used as sample containers. Pigment concentrations below 4 × 10^−6^ M were used in fluorescence measurements, providing optical densities <0.05 at the *Q*_y_ absorption maximum. This low concentration of Chl-a largely eliminated aggregation and self-absorption effects.

Absorption and fluorescence measurements were made using an experimental setup that has been previously described (Rätsep et al., 2009a, 2019a). It consists of a Andor Shamrock 0.3 m spectrograph equipped with a thermo-electrically cooled CCD camera, a highly stabilized tungsten light source, and a He bath cryostat. Selective excitation of fluorescence was performed using a model 375 dye laser of <0.5 cm^−1^ linewidth, pumped by a Spectra Physics Millennia solid state laser. The inhomogeneous spectral resolution of the measurements was 6.6 cm^−1^.

### Dipole Strength Representation of the Observed Spectra

Absorption and emission band strengths were obtained from raw observed spectra through the application of appropriate frequency scaling. Absorption spectra were scaled by ν^−1^ while emission spectra were scaled by ν^−3^, as absorption and emission strengths are proportional to ν*M*^2^ and ν^3^*M*^2^, respectively, where *M*^2^ ≡ *I* is the band strength (Einstein, 1916) and ν is the absorption frequency. As all emission spectra were recorded linearly in wavelength, the total scaling of the emission spectra used was ν^−5^, with the additional factor of ν^−2^ required so as to preserve the integrated emission probability.

### Vibrational Structure of the Spectra

In the Huang-Rhys approximation (Huang et al., 1950), the relative band strength of the 0-*n*_*i*_ transition delivering *n*_*i*_ quanta into vibrational mode *i* is given by the Poisson distribution

(1) $$\begin{array}{l} I_{i}=e^{-S_{i}}S_{i}^{n_{i}}/n_{i}! \end{array}$$

where *S*_*i*_ is the Huang-Rhys factor for mode *i*. The relative band strength of the transitions from the zero-point level of one state to an arbitrary level ***n*≡{*n***_**1**,_***n***_**2**_**, ...*n***_***i***_**...}** of the other state compared to that of the origin line strength, *I*_00_, is given by

(2) $$\begin{array}{l} \frac{I_{n}}{I_{00}}=\underset{i}{\prod }\frac{S_{i}^{n_{i}}}{n_{i}} \end{array}$$

which reduces to

(3) $$\begin{array}{l} \frac{I_{i}}{I_{00}}=S_{i} \end{array}$$

for the 0–1 transition in mode *i*. The total Huang-Rhys factor for the electronic transition is thus given as

(4) $$\begin{array}{l} S_{vib}=\sum_{i}S_{i}=\frac{1}{I_{00}}\sum_{i}I_{i}=\frac{I_{vib}}{I_{00}} \end{array}$$

where *I*_*vib*_ is the total band strength attributable to 0–1 transitions, and the total band strength is decomposed as

(5) $$\begin{array}{l} I_{tot}=I_{00}+I_{vib}+I_{mq} \end{array}$$

where *I*_*mq*_ is the component attributable to multi-quanta (i.e., $\sum_{i}n_{i}>1$) transitions. The reorganization energy distributed by each mode is given by

(6) $$\begin{array}{l} \lambda_{i}=h\nu_{i}S_{i} \end{array}$$

and the total reorganization energy by.

(7) $$\begin{array}{l} \lambda =\sum_{i}\lambda_{i}. \end{array}$$

### DFT Calculations

Ground and *Q*_y_ excited-state optimized structures for Chl-a, modified with methyl replacing phytyl, are evaluated by the CAM-B3LYP (Yanai et al., 2004), MN15 (Yu et al., 2016), ωB97xD (Chai and Head-Gordon, 2008), and B3LYP (Becke, 1993) methods using Gaussian-16 (Frisch et al., 2016) with the 6-31G^*^ basis set (Hehre et al., 1972). Replacement of phytyl with methyl is not expected to have noticeable influences on the properties of interest herein, although its replacement with H (making a carboxylic acid) can be influential (Fiedor et al., 2003, 2008; Palm et al., 2019). Also, the choice of basis set has a small influence (Rätsep et al., 2011), but as this is much less than the effect of solvent variation, it is not pursued herein. As was already mentioned in the introduction, only gas-phase calculations are reported in the main text, but in the Supplementary Material results are presented using both implicit solvation, applying the polarizable-continuum model (PCM) (Tomasi et al., 2005), and/or explicit solvation. All such calculations include the D3(BJ) dispersion correction (Goerigk and Grimme, 2011).

The normal modes for both states are determined analytically and projected onto curvilinear coordinates using the DUSHIN program (Reimers, 2001). This approach expresses the geometry changes calculated by the various methods between the ground and excited states in terms of bond-length, bond-angle, and bond-torsion changes, these then being projected onto ground- and excited-state normal coordinates evaluated by Gaussian-16. The force-constant matrix after transformation to these redundant internal coordinates (Wilson et al., 1955) may optionally be scaled by factors of 0.95, 0.95, and 0.99 for the stretching, bending, and torsional motions, respectively. These factors were previously obtained by fitting the observed and CAM-B3LYP/6-31G^*^ calculated vibrational frequencies of free-base porphyrin for all modes except the CH and NH stretches (Rätsep et al., 2011).

## Results and Discussion

### Interpretation of Low-Resolution and High-Resolution Spectra

**Figure 1:**
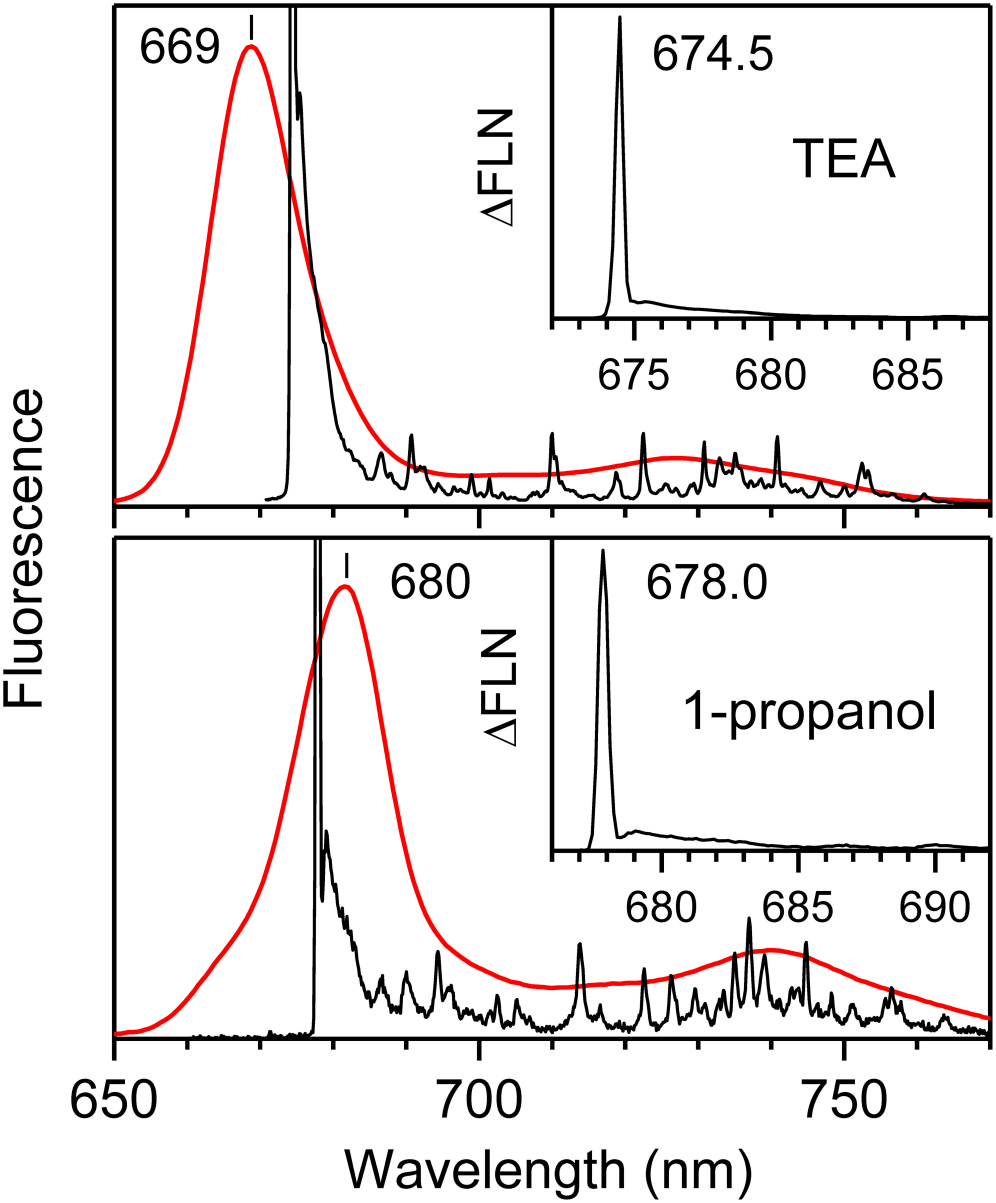
Low-resolution (red, excited at 407 nm) and high-resolution (black) ΔFLN fluorescence spectra of Chl-a in solid solutions of TEA and 1-propanol measured at 4.5 K. The ΔFLN spectra are vertically amplified to highlight the rich vibrational sideband structure. Insets show the in-scale origin parts of the ΔFLN spectra revealing intense ZPLs and weak PSBs in the red side.

The observed low-resolution and high-resolution (ΔFLN) fluorescence spectra of Chl-a in solid matrices of TEA and 1-propanol at 4.5 K are shown in Figure 1. The low-resolution fluorescence spectra were obtained following non-resonant excitation at 407 nm and are broad; in contrast, the resonantly excited ΔFLN spectra demonstrate a clear-cut vibrational structure, demonstrating the significant advantages of this technique. According to Equation (5), the ΔFLN spectrum displays an origin band that comprises the ZPL and PSB components, with intertwined *I*_*vib*_ + *I*_*mq*_ vibrational sidebands extending toward longer wavelengths. The shape of the origin band *I*_00_(ν) is known to depend on the excitation wavelength, with significant variations observed even for small changes within the inhomogeneous spread of ZPL frequencies, showing enhancement of the electron-phonon coupling strength with increasing transition wavelength (Rätsep et al., 2009b; Renge et al., 2011). Comparing the low-resolution spectra obtained in different environments, one may conclude that the vibrational sideband is more intense in 1-propanol than in TEA, details of which are revealed by the ΔFLN spectra. These important variances will be in detail elaborated below.

**Figure 2:**
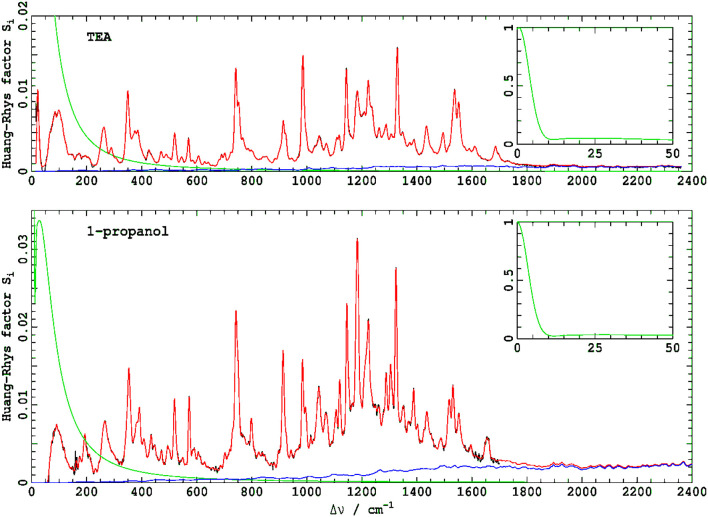
Vibrational parts of the ΔFLN spectra of Chl-a in TEA and 1-propanol recorded at 4.5 K. The green curves, shown on a different scale in the insets, indicate origin band profiles *I*_00_(ν) that are subtracted from experimental ΔFLN spectra to enhance clarity in the low-frequency region. The Huang-Rhys model fits (red) to the experimental vibrational contribution (black) was performed with the lineshape of every vibrational transition set to that observed for the 0–0 line, *I*_00_(ν). Contributions to the fit arising from multi-quanta transitions are shown in blue. The spectra are normalized to give a peak height of the 0–0 line of unity, allowing the Huang-Rhys factor *S*_*i*_ for any non-overlapped 0–1 transition to be read off the *y* axis from its peak height. The *I*_00_(ν) profiles (green) are expressed as a sum of two contributions: a Gaussian ZPL fitted to an observed resolution of 6.6 cm^−1^ and a PSB fitted to a standard form (Pajusalu et al., 2014).

Figure 2 shows the vibrational parts of the ΔFLN spectra, presented as a function of the frequency difference Δν from the ZPL maximum. These are displayed with their identified origin band profiles *I*_00_(ν) subtracted to enhance clarity in the low-frequency region up to about 600 cm^−1^. The *I*_00_(ν) profiles, shown in green in the main figures and their insets, are expressed as a sum of two contributions: a Gaussian ZPL function fitted to an observed resolution of 6.6 cm^−1^ and a phonon side band (PSB) fitted to a standard form (Pajusalu et al., 2014).

**Figure 3:**
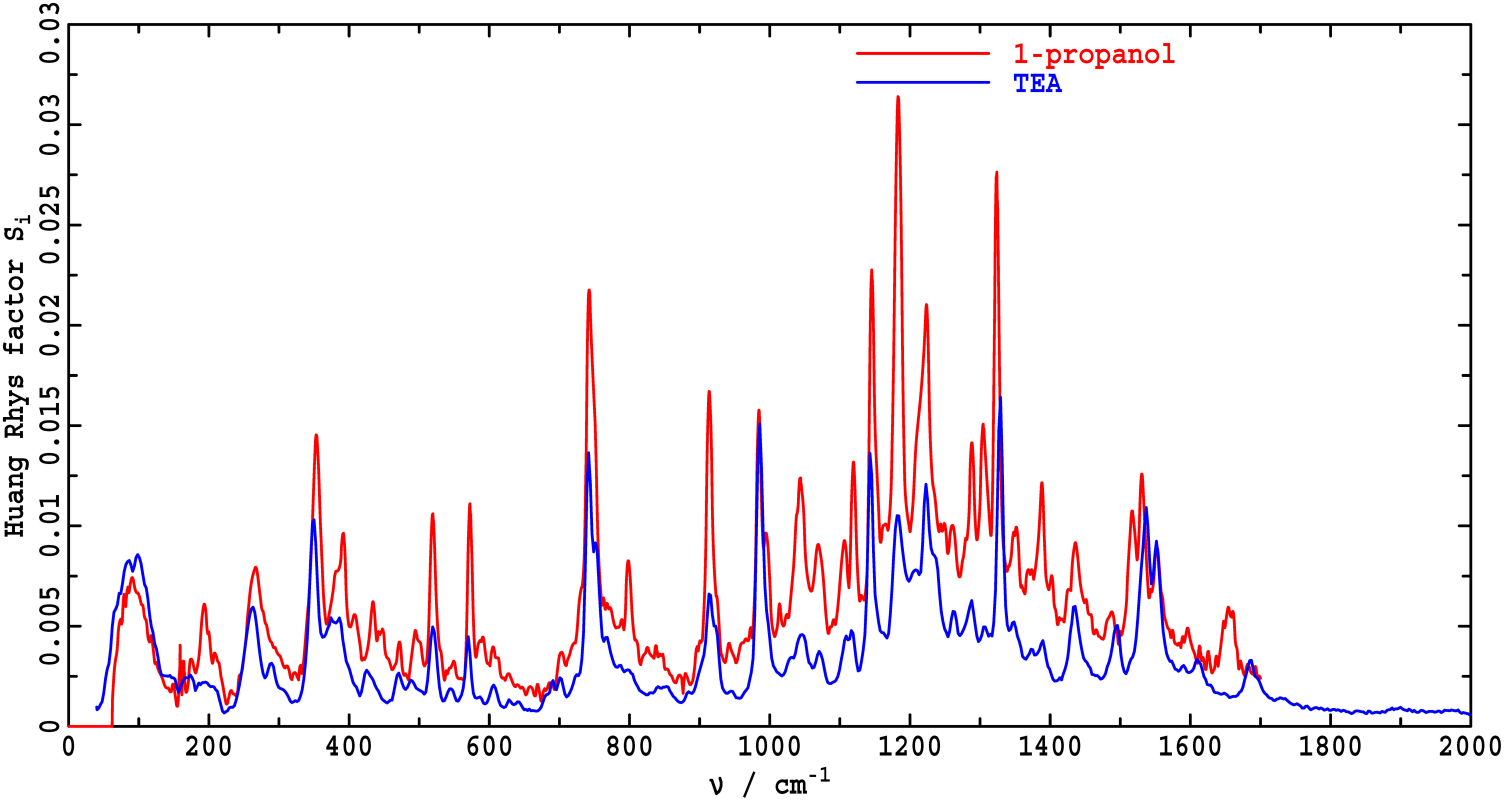
Comparison of vibrational parts of the ΔFLN spectra of Chl-a in 1-propanol and TEA recorded at 4.5 K. The *y*-scale is such that the value of the Huang-Rhys factor for a vibrational fundamental that is not overlapped by other peaks is given by the peak height, see text for further details.

In Figure 3, the two ΔFLN spectra are overlaid to facilitate detailed comparison: most lines appear to share common frequencies, with in only a few cases frequency shifts being apparent that are most likely associated with specific solvation effects. In contrast, it is clear that the intensity patters change significantly. Hence, vibrational frequencies are mostly insensitive to the environment, whereas the associated intensities, which depend on the details of the vibrational motions, are very sensitive.

For quantitative evaluation, the observed ΔFLN spectra were fitted with Equation (5), using a model containing 215 vibrational modes for Chl-a in 1-propanol and 296 models for Chl-a in TEA. For the spectrum in TEA, this was done using a fully automated procedure in which all frequency intervals were set to be less than the spectral resolution of the measurement, whereas for the 1-propanol spectrum, the frequencies were optimized to create a model containing the fewest-possible number of modes. These two approaches, full automation and human perceptive intuition, yield results of similar quality and usefulness. In both cases, the associated Huang-Rhys factors *S*_*i*_ were optimized to minimize the difference between the observed and fitted spectra. By using *I*_00_(ν) as the profile for each and every vibrational line (Rebane, 1970), the fundamental and multi-quanta contributions *I*_*vib*_(ν) + *I*_*mq*_(ν) were determined simultaneously during the fitting.

In Figure 2, the fitted spectra in red are overlaid on top of the observed spectra shown in black. That very little black color can be seen in the figures is indicative of the very high quality of the fitted results. Contributions to the spectra from multi-quanta excitations *I*_*mq*_(ν) indicated in blue are small yet significant to the quantitative analysis. Note that, in these figures, the *y*-axis is labeled as “Huang-Rhys factor *S*_*i*_,” reflecting traditional analysis methods. If a 0-1 vibrational line is isolated from all others, then the height of the line in this presentation is its Huang-Rhys factor. However, as the shape of each line is taken to be *I*_00_(ν), both presented spectra embody strong line overlap and hence interpretation of line data in this way is qualitatively indicative but not quantitatively accurate.

The obtained {ν_*i*_, *S*_*i*_} data sets provide a quantitative description of the observed spectra and can qualitatively be partitioned into regions associated with resolved spectral peaks and diffuse background regions. The present data could be partitioned into 44 regions/blocks for 1-propanol and 72 regions for TEA, with each region identified with some particular resolved emission (see Tables 1, 2 as well as the related Supplementary Tables 4, 5, respectively). This partitioning was done so as to preserve the originally fitted total reorganization energy within each region, with an effective Huang-Rhys factor determined to suit. This results in reduced region-based data sets {ν_*j*_, *S*_*j*_} defined with

(8) $$\begin{array}{l} \;\lambda_{j}=\overset{\text{region }j}{\underset{i}{\sum }}\lambda_{i},\;S_{j}=\frac{1}{\nu_{max,j}}\lambda_{j}\;, \end{array}$$

where ν_*max,j*_ is the frequency of the identified peak in region *j*. In this way, the background signal, which provides a substantial fraction to the whole, is accumulated to its embodied ΔFLN peak. The resulting description, therefore, achieves simplicity whilst retaining key features required in, e.g., quantum simulations of exciton transport. Further similar simplifications retaining just a few critical modes may be required to enhance computational efficiency in such applications.

**Table 1:** Resolved component regions *j* fitted according to Equation (8) in Supplementary Table 5 to the observed ΔFLN spectrum of Chl-a in 1-propanol at 4.5 K, depicting the component peak frequencies ν_*j*_ (cm^−1^), the range of individual frequencies included in each region (cm^−1^), the net emission reorganization energy $\lambda_{j}^{E}$ (cm^−1^) for each region, and the effective emission Huang-Rhys factor $S_{j}^{E}$ required to reproduce this.

**ν*_***j***_***	**Range**	**1,000 ${\text{S}}_{\text{j}}^{\text{E}}$**	**$\lambda_{\text{j}}^{\text{E}}$**	**ν*_***j***_***	**Range**	**1,000 ${\text{S}}_{\text{j}}^{\text{E}}$**	**$\lambda_{\text{j}}^{\text{E}}$**
92^a^	66–151	41.2	3.8	985	958–1,008	31.7	31.2
194	157–210	16.5	3.2	1,016	1,015	3.0	3.0
267	218–315	33.1	8.8	1,043	1,023–1,049	23.8	24.8
353	322–366	30.6	10.8	1,064	1,055–1,080	16.5	17.5
380	372–379	7.1	2.7	1,107	1,086–1,107	11.5	12.7
392	385–392	11.1	4.3	1,120	1,113–1,135	16.3	18.3
408	400–414	7.0	2.9	1,146	1,145–1,167	36.3	41.6
434	422–435	5.9	2.6	1,183	1,174–1,192	46.8	55.4
448	442–456	5.2	2.3	1,224	1,198–1,250	55.9	68.5
472	463–479	3.3	1.6	1,261	1,258–1,271	7.8	9.9
495	487–502	6.0	3.0	1,288	1,277–1,289	13.5	17.5
519	510–541	14.3	7.4	1,306	1,296–1,311	18.1	23.6
549	549–560	2.2	1.2	1,324	1,317–1,339	29.5	39.1
573	573–584	11.8	6.8	1,352	1,347–1,359	8.5	11.5
591	592	2.5	1.5	1,374	1,368–1,381	7.4	10.1
606	599–613	4.3	2.6	1,388	1,388–1,395	8.6	12.0
664	620–697	5.7	3.8	1,402	1,403	2.8	4.0
703	703	2.2	1.6	1,436	1,414–1,465	15.7	22.6
743	712–755	47.7	35.5	1,488	1,471–1,495	3.8	5.6
763	763–775	8.0	6.1	1,517	1,502–1,521	12.0	18.3
798	782–805	11.2	8.9	1,531	1,525–1,536	11.4	17.5
838	810–882	8.4	7.1	1,552	1,542–1,585	11.0	17.0
914	903–933	27.5	25.1	1,596	1,596	1.2	1.9
942	942–951	2.7	2.6	1,654	1,648–1,661	6.3	10.4
Total						715	650

a *It is unclear as to whether all or part of this emission should be attributed to intramolecular vibrations, as reported in this table and elsewhere, or else to intermolecular phonons*.

**Table 2:** Resolved component regions *j* fitted according to Equation (8) in Supplementary Table 6 to the observed ΔFLN spectrum of Chl-a in TEA at 4.5 K, depicting the component peak frequencies ν_*j*_ (cm^−1^), the range of individual frequencies included in each region (cm^−1^), the net emission reorganization energy $\lambda_{j}^{E}$ (cm^−1^) for each region, and the effective emission Huang-Rhys factor $S_{j}^{E}$ required to reproduce this.

**ν*_***j***_***	**Range**	**1,000 ${\text{S}}_{\text{j}}^{\text{E}}$**	**$\lambda_{\text{j}}^{\text{E}}$**	**ν*_***j***_***	**Range**	**1,000 ${\text{S}}_{\text{j}}^{\text{E}}$**	**$\lambda_{\text{j}}^{\text{E}}$**
22	10–41	13.1	0.3	1,032	1,018–1,036	2.8	2.9
66	41–87	20.1	1.3	1,046	1,036–1,060	6.2	6.5
99	87–115	20.3	2.0	1,071	1,060–1,090	4.5	4.8
123	115–155	4.2	0.5	1,109	1,090–1,113	4.8	5.4
174	155–190	3.3	0.6	1,117	1,113–1,127	4.2	4.7
206	190–220	2.4	0.5	1,144	1,127–1,150	15.9	18.2
263	220–280	14.6	3.8	1,157	1,150–1,170	6.3	7.3
289	280–312	4.5	1.3	1,183	1,170–1,192	15.2	18.0
330	312–335	1.1	0.4	1,209	1,192–1,213	14.0	16.9
349	335–362	17.3	6.0	1,223	1,213–1,229	13.6	16.6
375	362–380	7.0	2.6	1,236	1,229–1,242	7.5	9.3
386	380–412	6.7	2.6	1,263	1,242–1,272	5.9	7.5
425	412–432	1.6	0.7	1,275	1,272–1,283	2.9	3.7
436	432–458	1.0	0.5	1,288	1,283–1,301	5.7	7.4
471	458–477	2.2	1.1	1,306	1,301–1,311	2.7	3.6
489	477–500	3.1	1.5	1,315	1,311–1,320	2.0	2.7
520	500–530	7.0	3.6	1,329	1,320–1,335	17.0	22.5
545	530–558	1.5	0.8	1,342	1,335–1,347	3.1	4.1
570	558–575	4.2	2.4	1,354	1,347–1,367	2.7	3.7
587	575–593	0.8	0.5	1,374	1,367–1,378	1.6	2.3
606	593–612	1.6	0.9	1,390	1,378–1,419	4.0	5.6
628	612–632	0.7	0.4	1,435	1,419–1,460	10.6	15.2
642	632–648	0.6	0.4	1,467	1,460–1,472	0.8	1.1
691	648–695	3.5	2.4	1,478	1,472–1,485	1.1	1.7
702	695–718	3.3	2.3	1,495	1,485–1,502	5.1	7.7
725	718–730	2.2	1.6	1,507	1,502–1,513	1.4	2.2
742	730–745	16.2	12.0	1,519	1,513–1,525	2.9	4.5
752	745–756	8.5	6.4	1,537	1,525–1,542	14.2	21.8
767	756–775	5.0	3.8	1,552	1,542–1,557	9.9	15.4
798	775–803	3.2	2.6	1,562	1,557–1,579	2.7	4.2
811	803–820	0.6	0.5	1,590	1,579–1,597	0.9	1.4
854	820–860	2.4	2.0	1,610	1,597–1,634	2.7	4.4
885	860–890	1.7	1.5	1,649	1,634–1,667	0.1	0.1
915	890–930	14.2	12.9	1,686	1,667–1,693	3.4	5.7
946	930–955	1.2	1.1	1,698	1,693–1,705	1.2	2.0
986	955–1018	25.3	24.9	1,710	1,705–1,723	0.2	0.3
Total						426	370

From the fits, the total Huang-Rhys factors *S*_*vib*_ are determined to be 0.72 in 1-propanol and 0.43 in TEA. Similarly, the total emission reorganization energies λ^*E*^ are determined to be 650 cm^−1^ and 370 cm^−1^, respectively; these and other deduced reorganization energies are collected into Table 3.

**Table 3:** Calculated (for methyl Chl-a) and observed (for Chl-a) reorganization energies (in cm^−1^) for *y*-polarized Franck-Condon allowed *Q*_*y*_ absorption (λ^***A***^) and emission (λ^***E***^), compared to analogous results for Pheo-a (Rätsep et al., 2019b) and BChl-a (Rätsep et al., 2011).

**Method**	**Mg Coord**.	**Pheo-a**		**BChl-a**		**Chl-a**	
		**λ*^***A***^***	**λ*^***E***^***	**λ*^***A***^***	**λ*^***E***^***	**λ*^***A***^***	**λ*^***E***^***
Obs.^j1^ 4.5 K TEA ΔFLN and SEF	-	317	395				
Obs.^j1^ low res. 4.5 K TEA	-	355	402				
Obs.^j2^ 1.7 K EtOH/MeOH low res. MCD	-	424					
Obs.^j3^ 4 K TEA ΔFLN	6				219		
Obs.^j3,j4^ 5 K HB	5			378			
Obs.^j3^ 295 K TEA	5			335	185		
Obs.^j3^ 4.5 K TEA low res.	6			196	236		
Obs.^j3^ 4.5 K TEA low res.	−5	-	-	335	260		
Obs. 5 K TEA, low res.	5					~300^h^	458
Obs. 5 K TEA, ΔFLN	5						370
Obs. 295 K TEA, low res.	5						413
Obs.^j2^ 4 K wet ether FE	5^f^					262^e^	
Obs. 295 K wet ether, low res.	5					264^i^	438
Obs.^j5^ 4 K 1-propanol ΔFLN	6						650
Obs.^j2^ 4 K 1-propanol low res.	6					~420^b^	~650^a^
Obs.^j5^ 295 K 1-propanol low res.	5					~490^c^	492
Obs.^j2,j6^ PS-I-200 HB	5					547^g^	
Obs.^j2,j7^ WSCP HB	5					645^g^	
CAM-B3LYP/6-31G* from calc. energies	4	1,214^d^^,^^j1^	481^j1^	350^j3^	210^j3^	607^j2^	517^j2^
CAM-B3LYP/6-31G* from scaled frequencies	4			300^j3^	200^j3^	567	492
B3LYP/6-31G* from calc. energies	4	184^j1^	165^j1^	130^j3^	150^j3^	243	245
MN15/6-31G* from calc. energies	4	493^j1^	282^j1^	184	167	390	358
ωB97XD/6-31G* from calc. energies	4	1,609^d^^,^^j1^	594^j1^	310^j3^	1,140^j3^	713	606

a *Observed spectrum (Rätsep et al., 2009a) is deconvoluted (see Figure 5) into two bands representing the dominant 6-coordinate species (85%) and a secondary 5-coordinate one using the spectral bandshape from ΔFLN, indicating that the ΔFLN results accurately depict traditional low-resolution data*.

b *Observed absorption and MCD spectra are fitted to a model depicting 85% 6-coordinate species, scaling the bandshape observed in wet ether by FE (see Figure 6) (Reimers et al., 2013)*.

c *Very crude estimate for the 5-coordinate species assuming that the x polarized absorption commences is located 1,000 cm^-1^ above Q_x_ origin and has the same intensity as that observed for the 6-coordinate species*.

d *One poorly represented mode depicting aromaticity in Q_y_ involving interactions with nitrogen lone-pair orbitals; neglecting this results become CAM-B3LYP 465 cm^-1^, ωB97XD 475 cm^−1^*.

e *After removal of Q_x_ using full-quantum spectral simulations (Reimers et al., 2013)*.

f *Ligand is water (Reimers et al., 2014)*.

g *From hole-burning (HB) data, but qualitatively unreliable owing to baseline uncertainties (Reimers et al., 2013)*.

h *After approximate removal of Q_x_ band using the bandshape deduced in wet ether (Reimers et al., 2013)*.

i *From analytical inversion (Reimers and Krausz, 2014) of absorption and MCD data (Umetsu et al., 1999)*.

j *References: 1- (Rätsep et al., 2019b), 2- (Reimers et al., 2013), 3- (Rätsep et al., 2011), 4- (Zazubovich et al., 2001), 5- (Rätsep et al., 2009a), 6- (Gillie et al., 1989), 7- (Hughes et al., 2010)*.

**Figure 4:**
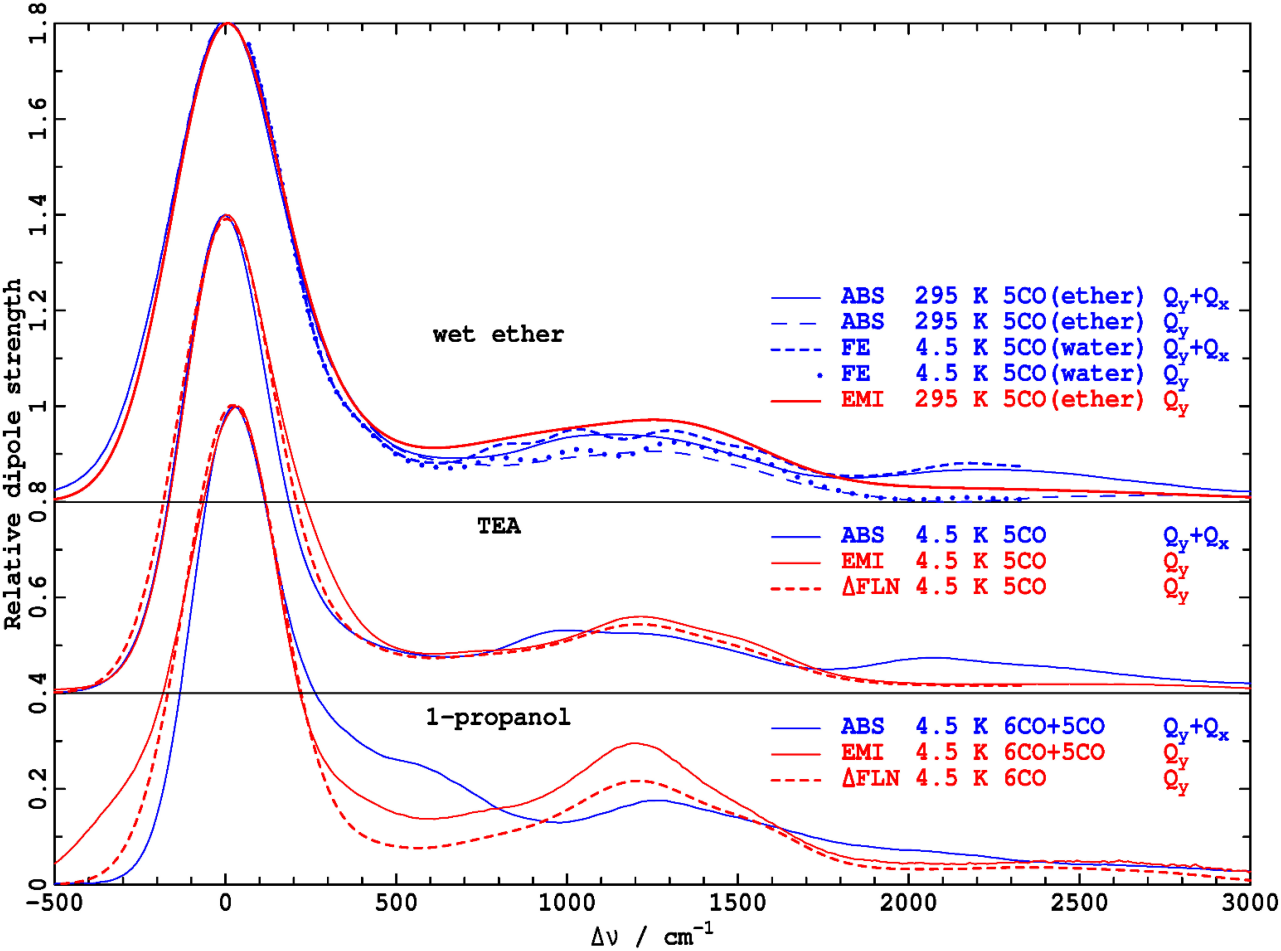
Absorption dipole strengths *A*(ν−ν_max_)/ν, blue, and reflected emission dipole strengths $E\left(-\nu +\nu_{\max}\right)/\nu^{3}$, red, for Chl-a in solvents with varying Mg coordination (CO), as determined by: ABS- absorption spectroscopy (in ether (Umetsu et al., 1999), TEA, and 1-propanol), perhaps in combination with analytical MCD data inversion (Reimers and Krausz, 2014) to extract the *Q*_*y*_ component, FE- broadened high-resolution fluorescence excitation (Avarmaa and Rebane, 1985), perhaps after subtraction of the *Q*_*y*_ component obtained during full-quantum spectral simulation (Reimers et al., 2013), EMI- emission spectroscopy (in ether (Reimers et al., 2014), TEA, and 1-propanol), ΔFLN- broadened delta fluorescence line narrowing data (in TEA and 1-propanol).

Fluorescence spectra at low resolution can be predicted based on the ΔFLN data analysis by broadening the spectra from the Huang-Rhys model to match that apparent in experimental low-resolution spectra. Results so obtained are indicated by red dashed lines in Figure 4, where they are compared to directly observed low-resolution emission spectra (red solid lines, denoted as EMI). In TEA, the observed and simulated low-resolution spectra are in reasonable agreement, validating the extensive experimental and computational procedures used in obtaining and interpreting the high-resolution ΔFLN data. From low-resolution spectra, the absorption and emission reorganization energies can be determined using

(9) $$\begin{array}{l} \lambda^{A}=h\frac{\int_{0}^{\infty }A\left(\nu \right)dv}{\int_{0}^{\infty \;}A\left(\nu \right)/\nu dv}-h\nu_{00},\\ \lambda^{E}=h\nu_{00}-h\frac{\int_{0}^{\infty \;}E\left(\nu \right)/\nu^{2}dv}{\int_{0}^{\infty \;}E\left(\nu \right)/\nu^{3}dv}, \end{array}$$

respectively, where *A*(ν) is the observed absorption coefficient and *E*(ν) the observed emission intensity. Note that, as emission intensities are sensitive to the spectral scan rate, *E*(ν) corresponds to the emission observed when the spectrum is scanned at a constant rate of change of frequency; spectra *E*(λ) scanned at a constant rate of change of wavelength λ = *c*/ν are related via *E*(ν) = *E*(λ)/ν^2^. For TEA, taking ν_00_ to be the frequency of the broad ZPL maximum, this gives λ^*E*^ = 458 cm^−1^, larger than the ΔFLN value of 370 cm^−1^.

**Figure 5:**
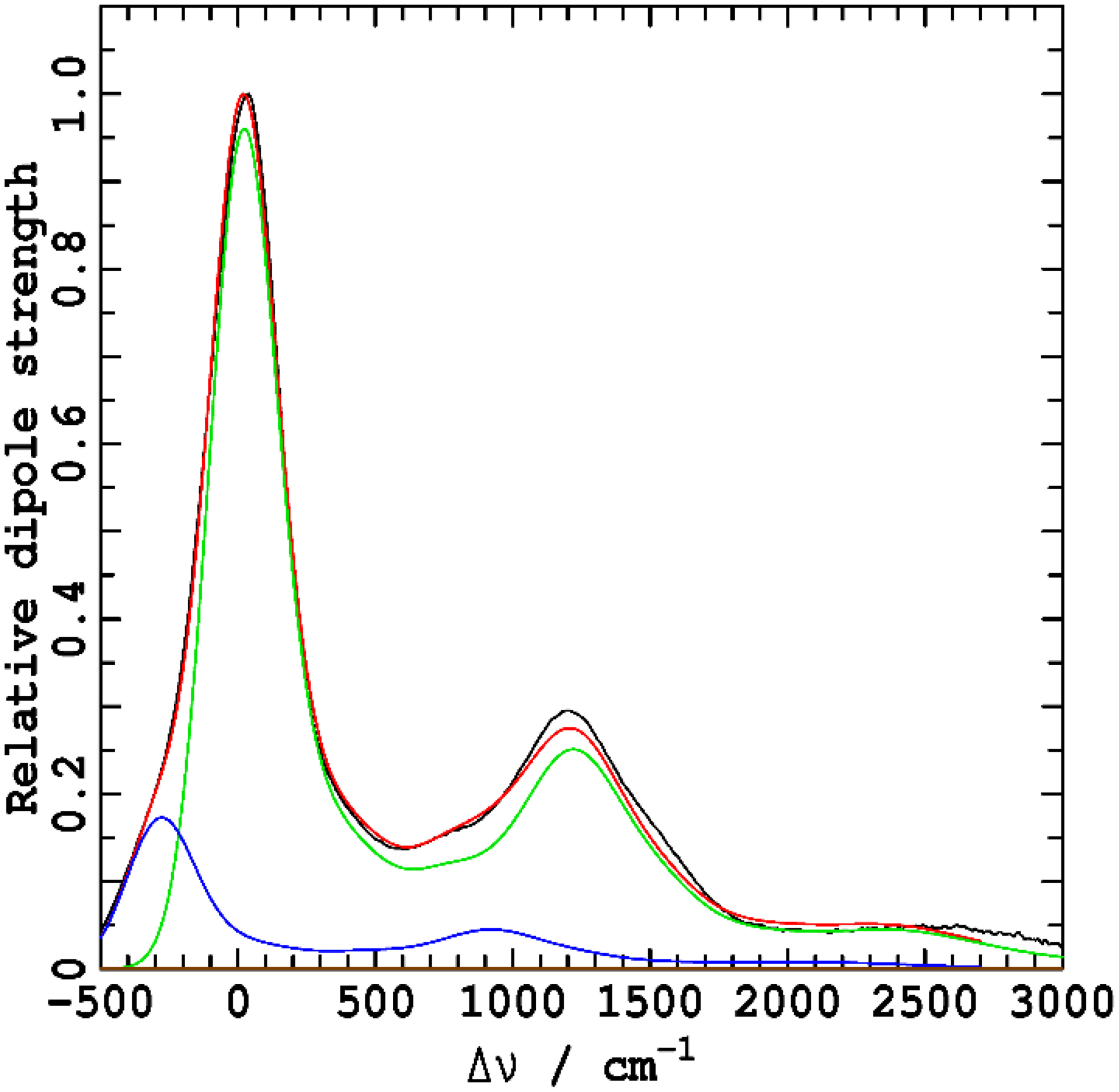
The observed emission spectrum of Chl-a in 1-propanol at 4.5 K (black, the EMI spectrum from Figure 4) is interpreted in terms of contributions from molecules with 6-coordinate Mg (green, 85%) and 5-coordinated Mg (blue, 15%). The 6-coordinate component bandshape is taken to be that as observed by ΔFLN, broadened with a Gaussian inhomogeneous distribution function fitted with FWHM = 280 cm^−1^ (taken from Figure 4). The 5-coordinate component bandshape is crudely taken to be the same as that for the 6-coordinate species. In this way, the fit is done using only three adjustable parameters: the relative component composition, the component ZPL frequency difference, and the FWHM. The sum of the two contributions is shown in red and closely approximates the observed emission.

Much larger differences are seen in Figure 4 for the analogous comparison of low-resolution spectra obtained by broadening the ΔFLN spectrum and from direct observation of emission in 1-propanol. A plausible explanation of the difference is that some Chl-a molecules have 5-coordinate Mg atoms, even at the low temperatures used; the ΔFLN spectra select only for the major (6-coordinate) component, whereas the EMI spectrum captures all emission. In Figure 5, the observed low-resolution emission spectrum is approximately separated into 5-coordinate (15%) and 6-coordinate (85%) components, yielding a 6-coordinate reorganization energy of ~650 cm^−1^, in good agreement with the value obtained for the pure 6-coordinate species using ΔFLN.

Analogous data pertaining to the *Q*_*y*_ absorption spectrum of Chl-a is difficult to obtain owing to interference from the *Q*_*x*_ band. High-resolution fluorescence excitation (FE) spectra of Chl-a have been measured by Avarmaa and Rebane (1985), and subsequently the doubly-peaked *x-*polarized intensity was subtracted using an extensive quantum-mechanical non-adiabatic coupling model, combined with a Huang-Rhys analysis of the observed vibrational structure (Reimers et al., 2013). The resulting *Q*_*y*_ vibrational frequencies and Huang-Rhys factors are reproduced in Supplementary Table 6. The solvent used by Avarmaa and Rebane is best characterized as wet ether (Reimers et al., 2014), with, at the low temperatures used, water coordinating the chlorophyll Mg atom. The original observed FE spectrum, as well as that after subtraction of *Q*_*x*_, both after broadening to match low-resolution (Umetsu et al., 1999) absorption spectra observed in ether at room temperature, are shown in Figure 4. In an alternate approach, the *Q*_*x*_ component of the room-temperature absorption (ABS) spectrum has been subtracted using an analytical technique for the inversion of observed absorption and magnetic circular dichroism (MCD) data (Reimers and Krausz, 2014). The total absorption and resulting *Q*_*y*_-component spectrum are also shown in the figure.

As seen, the full absorption spectrum is in good agreement with that obtained by broadening the observed FE spectrum, and the two *Q*_*y*_-only components are also in good agreement. Both approaches lead to the conclusion that the absorption reorganization energy is λ^*A*^ = 262 cm^−1^, much less than the emission reorganization energy determined from the low-resolution room-temperature spectrum in ether, λ^*E*^ = 438 cm^−1^. This is visually very apparent from the absorption and fluorescence spectra compared in Figure 4.

**Figure 6:**
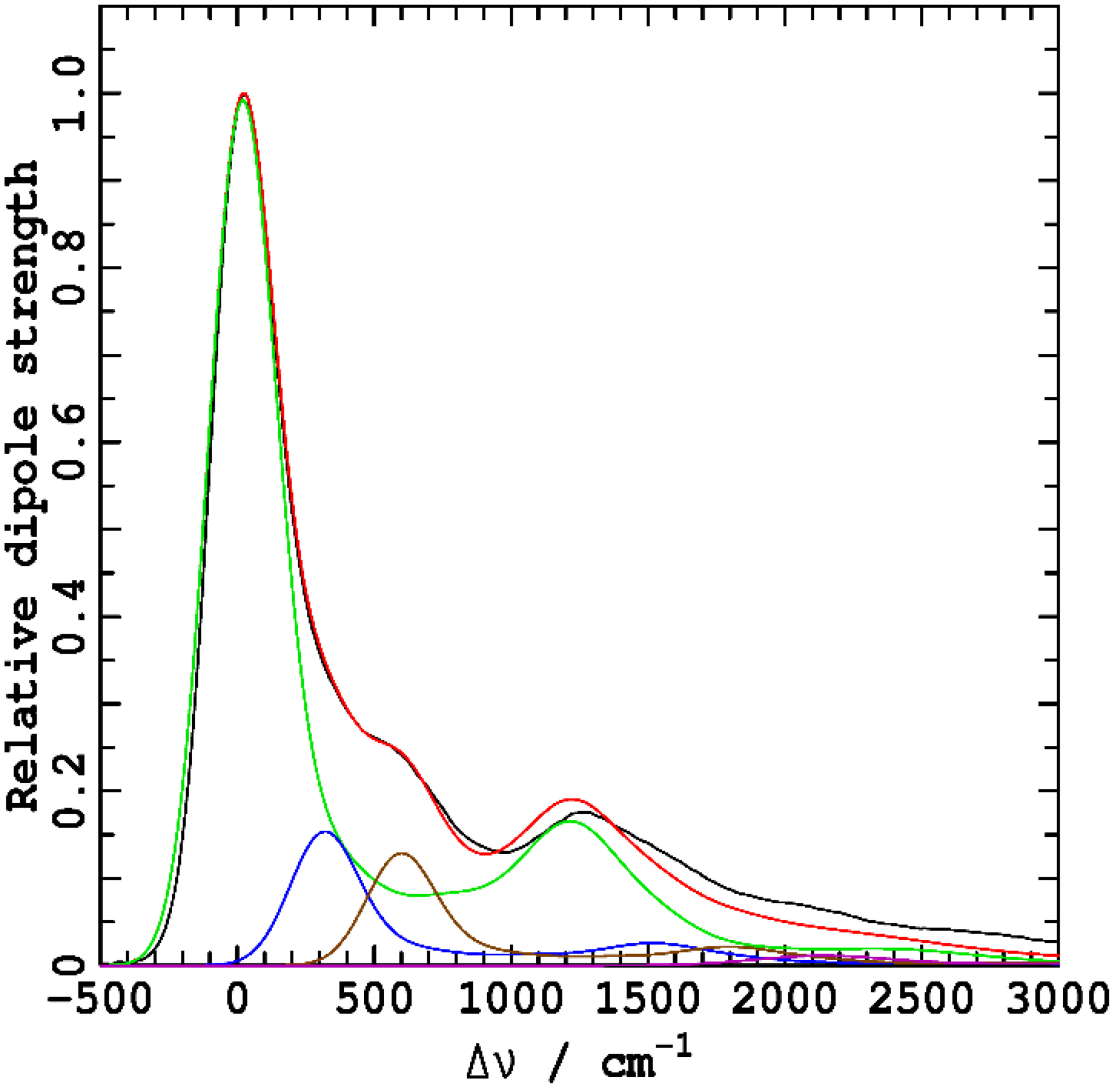
Separation of the observed (Rätsep et al., 2009a) absorption spectrum *A*(ν) of Chl-a in 1-propanol at 4.5 K, frequency-weighted as *A*(ν)/ν to reveal the dipole strength (black), into two *y*-polarized components arising from molecules with 6-coordinate magnesium (green, 85%) and 5-coordinate magnesium (blue, 15%, 295 cm^−1^ higher in energy), and the *x*-polarized intensity (blue, the result of strong non-adiabatic coupling between vibrational lines of *Q*_*y*_ and the *Q*_*x*_ origin), using for it the spectral profile extracted from MCD data in wet ether (Reimers et al., 2013). The two absorption bandshape profiles are assumed to be the same and are set to that depicted by the bandshape for Chl-a in wet ether at 4.2 K determined from FE (Supplementary Table 3), located and scaled to fit the observed absorption (red).

Similar accurate descriptions of the *Q*_*y*_ absorption in 1-propanol and TEA are not available. Yet rough estimates can be made by partitioning *x* and *y* polarization, assuming that the *Q*_*x*_ band profile is not solvent dependent, and, for 1-propanol, as in Figure 5, that the *Q*_*y*_ absorption bandshape of the 5-coordinate species is the same as that for the 6-coordinate one. Results for the separation of the two species in 1-propanol at 4 K are shown in Figure 6. They provide rough estimates of absorption reorganization energy of λ^*A*^ = 420 cm^−1^ for the 6-coordinate species and 490 cm^−1^ for the 5-coordinate one. As for ether, absorption reorganization energies appear to be much less than those in emission.

### Relating the Observed High-Resolution Absorption and Emission Properties Based Upon DFT Calculations of the Duschinsky Matrix

From Figure 3, as well as Tables 1, 2, and S6, it is clear that the vibrational frequencies in different solvents and in the *S*_0_ ground state and *Q*_*y*_ excited states are quite similar, yet the vibrational intensities (Huang-Rhys factors) and associated reorganization energies can show large differences. Vibrational motions in the two states are clearly similar, but small differences can have profound effects. For example, two (or more) modes of very similar frequencies can mix strongly with each other as a function of small environmentally introduced changes in their frequency differences, moving in and out of resonance. Alternatively, one mode can retain its basic form in the other state yet mix very slightly with a large number of modes that span a wide frequency range. While the mixing with any one mode remains small, the effects of mixing with many modes can reinforce each other to manifest profound consequences. To quantify this, the required tool is the Duschinsky rotation matrix

(10) $$\begin{array}{l} \text{D}={\left({\text{C}}^{{\text{S}}_{0}}\right)}^{T}{\text{C}}^{{\text{Q}}_{\text{y}}} \end{array}$$

where ${\text{C}}^{{\text{S}}_{0}}$ are the normal modes of vibration of the ground state and ${\text{C}}^{{\text{Q}}_{\text{y}}}$ are those for the excited state. These normal-mode matrices are expressed in terms of mass-weighted Cartesian coordinates. Duschinsky matrices can also be used to understand how the normal modes of vibration respond to other changes, e.g., solvent changes. Unfortunately, the Duschinsky matrix contains *n*^2^ elements, where *n* is the number of vibrational modes, for which little direct evidence is provided by experimental measurements, as most observed properties depend on combinations of multiple Duschinsky-matrix elements.

Following the success with similar studies on BChl-a (Rätsep et al., 2011) and Pheo-a (Rätsep et al., 2019b), we turn to DFT calculations of **D** to relate the vibrational motions of the *S*_0_ and *Q*_*y*_ states of Chl-a. To be useful, such calculations must first describe adequately the Huang-Rhys factors pertinent to absorption and emission. Unfortunately, previous experience with chlorophyllide spectral modeling has indicated that different possible density-functional approaches, as well as alternative *ab initio* or semi-empirical methods, can lead to errors in predicted reorganization energies by an order of magnitude or much more, as well as predicted absorption and/or emission profiles that do not resemble those observed (Rätsep et al., 2011, 2019b). To find a suitable computational method, we sampled four DFT approaches–CAM-B3LYP (Yanai et al., 2004; Kobayashi and Amos, 2006), B3LYP (Becke, 1993), MN15 (Yu et al., 2016), and ωB97XD (Chai and Head-Gordon, 2008)–as these have previously offered results of interest.

The observed and calculated emission and absorption reorganization energies for Chl-a are compared in Table 3, along with those for Pheo-a and BChl-a. CAM-B3LYP was previously identified (Rätsep et al., 2011, 2019b) as the best choice for BChl-a and Pheo-a, and this remains the case for Chl-a too. It is a hybrid functional with asymptotic potential correction, a type of method identified as the entry level for spectroscopic calculations on aromatic molecules (Cai et al., 2006) (as is ωB97xD, but ωB97xD is prone to larger errors). Using B3LYP, low chlorophyllide reorganization energies similar to the experiment have been predicted, but the associated spectral profiles have had no relationship to those observed. MN15 appears to be a method of interest and the only identified alternative to CAM-B3LYP. Yet CAM-B3LYP and MN15 both showed serious failures for a single, though different, mode in absorption for Pheo-a (Rätsep et al., 2019b). Hence, we see that just because a method usefully describes most vibrations of interest, it does not mean that all such vibrations are reliably defined. For CAM-B3LYP, the poorly described mode depicts aromaticity in the *Q*_*y*_ state and is affected by non-adiabatic coupling to nitrogen (*n*,π^*^) states that only exist in pheophytins, so the error is not relevant to Chl-a.

**Figure 7:**
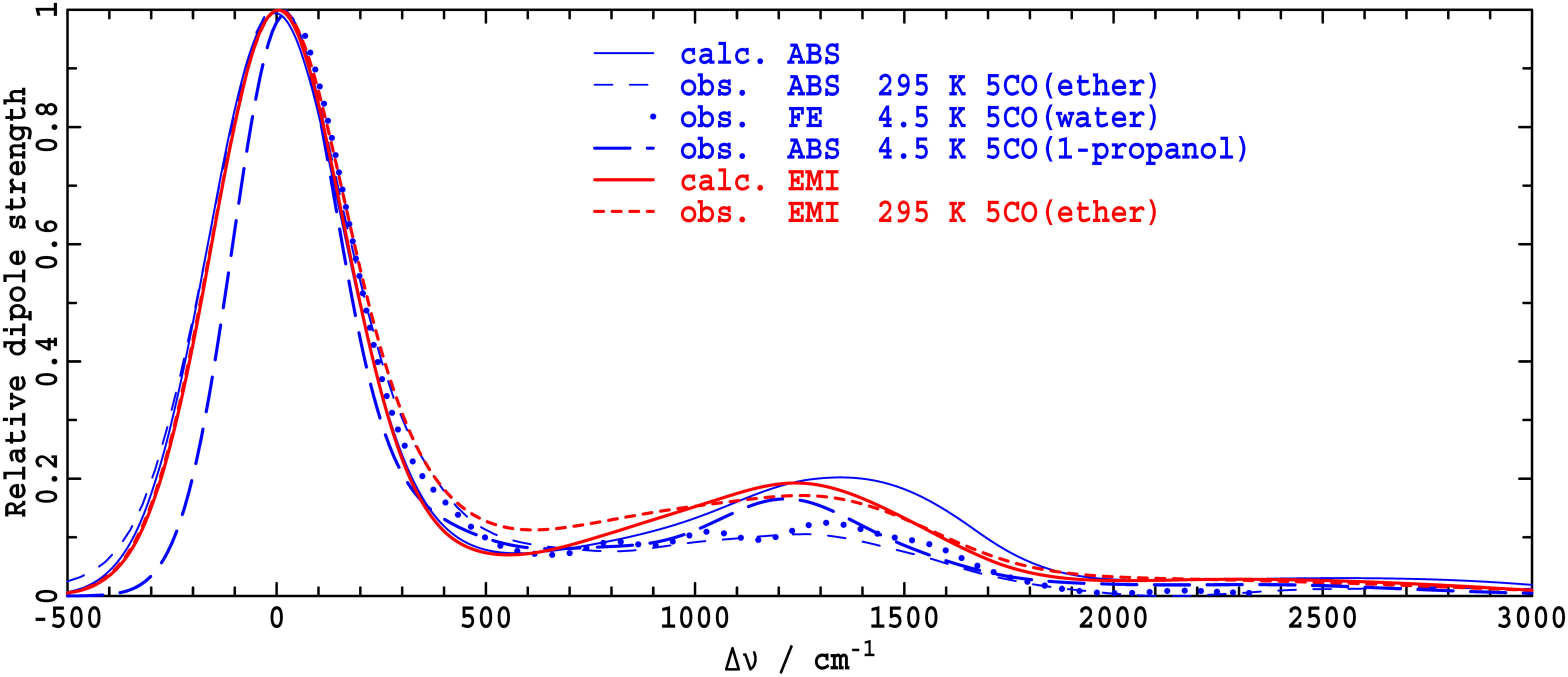
Low-resolution *Q*_*y*_ gas-phase spectra calculated from the CAM-B3LYP vibrational frequencies and displacements for absorption (ABS or FE), *A*(ν − ν_max_)/ν, and reflected emission (EMI), $E\left(-\nu +\nu_{\max}\right)/\nu^{3}$, are compared to those deduced from experimental data for 5-coordinate species. For the absorption spectra, the coordinating ligand is either ether (see Figure 4), water from wet-ether solution (see Figure 4), or 1-propanol (Figure 6), with ether being the ligand for the emission spectrum (see Figure 4).

That the CAM-B3LYP results present a useful starting point for understanding the observed high-resolution data that is demonstrated in Figure 7, where low-resolution simulated absorption and emission spectra are compared to observed results. The calculated gas-phase emission spectrum closely resembles the shown observed emission in ether at 295 K, with the differences being less than the observed changes presented in Figure 4 as a function of solvent. The calculated gas-phase absorption spectrum depicts a 0–1 vibrational sideband in the 800–1,600 cm^−1^ region that is significantly enhanced compared to the shown observed spectra taken in ether, and somewhat enhanced compared to the observed spectrum (taken from Figure 6) in 1-propanol. Such enhancements are expected based on the overestimation of the absorption reorganization energy reported in Table 3. Of note, however, is that this effect is not mode specific and hence a qualitatively indicative depiction of the high-resolution information is expected.

The computational method chosen must also accurately represent the vibrational frequencies of Chl-a. In applying CAM-B3LYP results to interpret chlorophyllide spectra, it is common to rescale the force constants as a crude correction for anharmonicity (Rätsep et al., 2011, 2019b), which slightly reduces the calculated reorganization energies, to make them closer to those observed (Table 3). Analogously scaled CAM-B3LYP/6-31G^*^ values are known to reproduce the observed high-resolution data for 90 vibrations of porphyrin to within a root-mean-square error of 22 cm^−1^ (Rätsep et al., 2019b), and hence are expected to be reliable also for Chl-a.

Table 4 presents the high-resolution results from the CAM-B3LYP calculations in terms of modes of *Q*_*y*_ predicted to dominate absorption, modes of the ground state predicted to dominate emission, and the critical Duschinsky matrix elements that interconnect them. Some key modes in one state may be directly mapped onto key modes in the other state, the simple situation that gives rise to absorption-emission asymmetry, but other key modes are extremely mixed and have no clear assignment, leading to the absence of absorption-emission asymmetry. In the table, 30 of the 37 observed modes listed for absorption and/or emission are assigned to calculated modes based on proximity and reorganization energy. This mapping includes the most important modes either observed or calculated in both absorption and emission. It is thus likely that the analysis captures the essential qualitative elements of the intrinsic relationships, without addressing important quantitative subtleties such as the observed dependence of emission on solvent and coordination. Indeed, most observed modes in emission are related to those in absorption by this procedure.

**Table 4:** Tentative relationships between observed Chl-a *Q*_*y*_ vibrational modes (Reimers et al., 2013) from fluorescence excitation (Avarmaa and Rebane, 1985) in wet (Reimers et al., 2014) ether at 4.2 K and observed *S*_0_ modes from FLN in 1-propanol or TEA at 4.5 K, see Tables 1, 2.

^****1****^**Q**_****y****_ **FE wet ether**		^****1****^**Q**_****y****_ **calc**.		**^**1**^Q_**y**_ assignment in terms of S_**0**_ modes**	**S**_****0****_ **calc**.		**S**_****0****_ **ΔFLN TEA**		**S**_****0****_ **ΔFLN 1-propanol**	
**ν_i_**	**λ_i_**	**ν_i_**	**λ_i_**		**ν_i_**	**λ_i_**	**ν_i_**	**λ_i_**	**ν_i_**	**λ_i_**
–	-						22	0.3	-	-
		64	16	42% 64, 28% 41, 19% 54	41, 64	12				
110	1	96,99	7	100% 93, 89% 99	93,99	7	99	2	92^b^	4^b^
190	1	155	5	98% 154	154	5	170–210	1	194	3
263	2	241,263	3	96% 244, 95% 267	244,267	3	263	4	267	9
344	3	343	4	98% 345	345	2	349	6	353	11
370–390	3	375,385	4	95% 388, 82% 391	388,391	3	370–390	5	370–390	7
435–470	1	464	2	75% 470	470	2	430–450	1	430–450	5
		494	2	81% 501	501	3				
515	0.3	505	3	75% 513	513	1	520	4	519	7
563	3	589	2	99% 593	593	2	570	2	573	7
739	11	731,735	20	60% 737, 51% 749, 35% 739	737,739	16	742	12	745	36
788	3	837	4	87% 839	839	2	798	3	798	9
925	3	917	7	39% 895, 24% 897	895,897	15	915	13	914	25
966	23	977,979	36	87% 977, 45% 986	977	40	986	25	985	31
1,034	6						1,046	7	1,043	25^c^
1,070	8	1,084, 1,092	8	91% 1093	1,093	1	1,071	5	1,064	18^c^
1,107	4	1,110	2	50% 1,110, 17% 1,102, 18% 1,122	1,102, 1,122	3	1,109	5	1,107	13^c^
1,132	5	1,131	15	70% 1,131, 18% 1,149	1,131	10	1,117	5	1,120	18
					1,149^a^	12	1,144	18	1,146	42
1,165	8	1,159	14	37% 1167	1,167	12	1,183	18	1,183	55
1,196	5	1,193	9	38% 1,184, 5% 1,192	1,192	5	1,209	17		
		1,212	5	33% 1,221, 60% 1,211	1,211	26	1,236	9		
1,228	15	1,224	47	23% 1,211, 60% 1,221	1,221	35	1,223	17	1,224	69
1,253	18	1,260	15	89% 1262	1,262	11	1,263	8	1,261	10
1,286	16	1,285, 1,287, 1,288	13	86% 1,283, 82% 1,286, 60% 1,287	1,286, 1,287, 1,288	9	1,288	7	1,288	18
		1,306	6	26% 1,288, 16% 1,312, 17% 1348	1,288^a^	3				
		1,323	10	46% 1324, 20% 1338, 11% 1331	1,324	17	1,306	4	1,306	24
		1,329	12	31% 1,321, 27% 11,348, 15% 1,338	1,321^a^	37	1,329	23	1,324	39
1,332	18	1,332	17	56% 1,331, 16% 1,321, 11% 1,338	1,331	0				
					1,338^a^	4	1,354	4	1,352	12
1,369	10	1,364	4	44% 1,366, 12% 1,380, 13% 1,388	1,366	2	1,374	2	1,374	10
1,393	7	1,392	7	22% 1,396, 17% 1,380, 14% 1,384	1,396	5	1,390	6	1,388	12
1,415	6	1,423	10	31% 1,458, 18% 1,441						
		1,424	5	48% 1,422, 25% 1,441	1,422	5				
					1,441	5	1,435	15	1,436	23
		1,459	0	57% 1,465	1,458	5	1,467	1	1,488	6
1,446	8	1,466	3	45% 1,470, 13% 1,522	1,470	0				
1,510^d^	18	1,501	15	55% 1,481,1,486	1,481, 1,486	24	1,519	5	1,517	18
1,530	9	1,530	66	46% 1,538, 24% 1,590	1,538	26	1,537	22	1,531	18
1,587	11	1,570	42	24% 1,562, 17% 1,568	1,568	11	1,552	15	1,552	17
		1,595	29	54% 1,617, 19% 1,590	1,617	8	1,686	6	1,654	10
1,665	4	1,608	3	45% 1,590, 24% 1,617	1,590	11	1,610	4	1,596	2
	188		415	Total, listed modes		355		301		500
	262		567	Total, all modes		492		370		650

*These are obtained by assigning the observed lines to modes calculated for methyl Chl-a using CAM-B3LYP and the calculated Duschinsky matrix elements to map the calculated modes of Q_y_ onto those of S_0_*.

a *No clear assignment, distributed over many Q_y_ modes*.

b *It is unclear as to whether all or part of this emission should be attributed to intramolecular vibrations, as reported in this table and elsewhere, or else to intermolecular phonons; modes of lower frequency are not easily identifiable in spectra*.

c *No plausible assignment*.

d *Broad band, originally listed at 1,493 cm^-1^ but the peak in this region is at 1,510 cm^−1^*.

**Figure 8:**
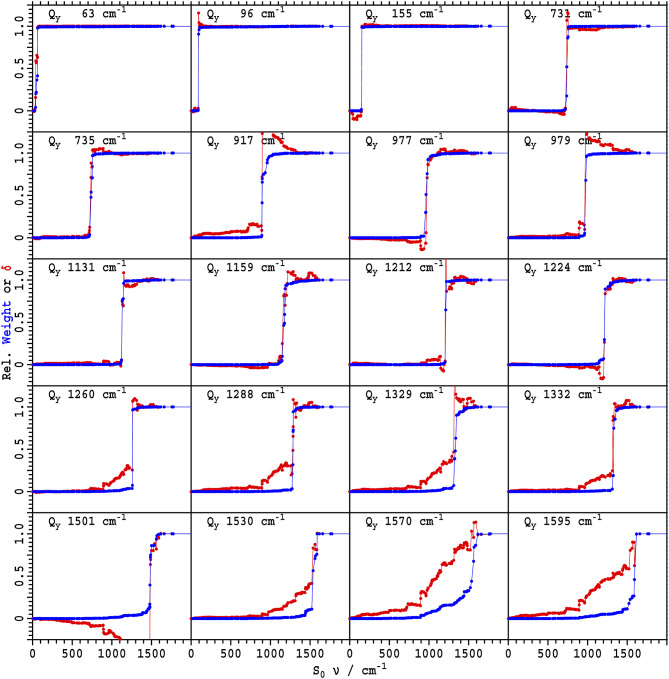
The origin of 20 significant *Q*_*y*_ normal modes of Chl-a from CAM-B3LYP/6-31G* calculations in terms of the *S*_0_ normal modes (blue), as well as the fractional contribution of each ground-state mode to the excited-state absorption displacement $\delta_{i}^{A}$ to control its Huang-Rhys factor (red); see Equation (10).

How the Duschinsky matrix elements control absorption/emission asymmetry is shown in Figure 8. In this figure, for 20 vibrational modes of *Q*_*y*_, the mode origin and the development of its Huang-Rhys factor is indicated in terms of contributions from each ground-state mode. The Huang-Rhys factors in absorption and emission, $S_{i}^{A}$ and $S_{i}^{E}$, respectively, can be expressed as

(11) $$\begin{array}{l} S_{i}^{A}={\left(\delta_{i}^{A}\right)}^{2}/2\text{ and }S_{i}^{E}={\left(\delta_{i}^{E}\right)}^{2}/2 \end{array}$$

in terms of dimensionless nuclear displacements on the ground state $\delta_{i}^{E}$ and excited state $\delta_{i}^{A}$

(12) $$\begin{array}{l} \delta_{i}^{E}=\;{-\left(\frac{2\pi \nu_{i}^{S_{0}}}{\hbar }\right)}^{1/2}\sum_{j=1}^{N}C_{ji}^{S_{0}}m_{j}^{1/2}{\Delta x}_{j},\\ \delta_{i}^{A}={\left(\frac{2\pi \nu_{i}^{Q_{y}}}{\hbar }\right)}^{1/2}\sum_{j=1}^{N}C_{ji}^{Q_{y}}m_{j}^{1/2}{\Delta x}_{j}, \end{array}$$

where Δ*x*_*j*_ is the change in *Q*_*y*_ from *S*_0_ of a Cartesian coordinate of an atom of mass *m*_*j*_, or its equivalent after curvilinear transformation (Reimers, 2001). The displacements are related through the Duschinsky matrix (Equation 10) as

(13) $$\begin{array}{l} \delta_{i}^{A}=-\overset{n}{\underset{k=1}{\sum }}{\left(\frac{\nu_{i}^{Q_{y}}}{\nu_{k}^{S_{0}}}\right)}^{1/2}D_{ki}\delta_{k}^{E},\;\text{where }1=\;\overset{n}{\underset{k=1}{\sum }}D_{ki}^{2} \end{array}$$

(Note that the Duschinsky matrix is orthogonal in rectilinear coordinates but not necessarily so in curvilinear coordinates, see Supplementary Material for the details of treatment in this case). Of interest, the frequency-weighting term in Equation (13) shows how low-frequency modes in emission can preferentially contribute to absorption intensity.

Full depictions of how each ground-state mode contributes to the vibrational density and displacement (Equation 13) are provided in Supplementary Figures 1, 2. For some key modes, enhanced descriptions are provided in Figure 8. This figure shows the cumulative contribution $D_{ik}^{2}$ of each ground-state vibration *k* to the excited state vibration *i*, which is shown in blue (also some key contributions are listed in Table 4). Typically, just a small number of ground-state modes mix to form each excited state, with the blue curves approximating Heaviside step functions. Hence a close correlation is observed between ground- and excited-state vibrational frequencies, with the resulting frequencies depending only on sums of densities $D_{ik}^{2}$. Shown also in red are the (fractional) cumulative contributions ${{\left(\nu_{i}^{Q_{y}}/\nu_{k}^{S_{0}}\right)}^{1/2}D}_{ik}\delta_{k}^{E}/\delta_{i}^{A}$ of each ground-state mode to the excited-state displacement $\delta_{i}^{A}$. As these contributions to the displacement are signed, the Huang-Rhys factors embody both constructive and destructive interferences. When the shown curves resemble Heaviside step functions, there is symmetry between absorption and emission (i.e., the transition moment in absorption matches that in emission), but in many cases interferences dominate and absorption/emission symmetry is lost.

A simple case (Figure 8, Table 4) is the mode predicted in absorption at 155 cm^−1^ and in emission at 154 cm^−1^, with a similarity of 98% as revealed by the Duschinsky matrix. These vibrations are attributed to the significant absorption and emission observed in the 190 cm^−1^ region.

Next, we consider some lines with large reorganization energy observed in *Q*_*y*_ at 1,228 cm^−1^ with λ^*A*^ = 47 cm^−1^ that is assigned to ground-state modes observed at 1,223 cm^−1^ with λ^*E*^ = 17 cm^−1^ in TEA and at 1,224 cm^−1^ with λ^*E*^ = 69 cm^−1^ in 1-propanol. The calculations indicate that two modes (23% 1,211 cm^−1^ and 60% 1,221 cm^−1^) mix to dominate the observed effects, sometimes constructively and sometimes destructively. Such mixing could explain the observed very large solvent dependence of the ΔFLN spectra, as it would be very sensitive to the environment. This mode has the largest observed reorganization energy in the ΔFLN spectra in 1-propanol, but a similar situation is found also for the 2nd-largest mode observed at 1,183 cm^−1^ in both 1-propanol and TEA, this time presenting solvent-dependent reorganization energies of 55 cm^−1^ and 18 cm^−1^, respectively. For the 2nd-largest mode, the calculations indicate that various ground-state modes contribute to the excited-state vibration (calculated at 1,159 cm^−1^), again allowing solvent effects to dramatically change mode intensities. A similar situation exists for the mode with the largest reorganization energy observed by ΔFLN in TEA (at 986 cm^−1^) that is assigned to interference between the calculated ground-state vibrations at 977 cm^−1^ and 979 cm^−1^.

Also of interest are the modes with large reorganization energy observed by ΔFLN in TEA at 1,144 cm^−1^ and in 1-propanol at 1,146 cm^−1^. These are assigned to a calculated ground-state vibration at 1,149 cm^−1^ that is made up of many small contributions from excited-state modes spanning 1,100–1,300 cm^−1^. Hence the calculations predict that this intense mode in emission has no counterpart in absorption, and indeed no counterpart is obvious from the observed fluorescence-excitation spectra.

Lastly, we consider eight example modes from the important high-frequency region calculated in *Q*_*y*_ between 1,260 and 1,595 cm^−1^ (Figure 8, Table 4). For these, the absorption Huang-Rhys factor is predicted to arise following extensive constructive interference involving many ground-state modes ranging often over 1,000 cm^−1^ in frequency. Emission/absorption symmetry is intrinsically lost through this process. The larger the number of modes involved and the greater the spread of frequencies, the more similar the reorganization energies observed by ΔFLN in TEA and in 1-propanol become. Hence, for the high-frequency modes, the major effect of solvent variation becomes frequency shifts associated with localized aspects of solvation. Also, energy absorbed by one of these high-frequency modes, in the absence of relaxation processes, will be re-emitted by all coupled ground-state modes and hence can be spread out into a band that is over 1,000 cm^−1^ wide.

In Supplementary Material, analogous results to those in Table 4 are presented in Supplementary Table 2 for the situation in which one 1-propanol ligand is bound to the Mg of Chl-a. Unfortunately, as detailed in Supplementary Table 1, approaches such as this using explicit solvation and/or implicit solvation fail to reproduce the basic observed changes in the absorption and emission reorganization energies from Table 3. Hence, details in the differences found between the high-resolution analyses presented in Table 4, Supplementary Table 2 are not expected to be meaningful. There are two qualitative features of interest, however. First, the basic pattern depicted in Table 4 does not change, indicating that many key calculated features are invariant to the treatment of solvation. Second, the calculations predict that some bands undergo small frequency changes but factors of three change in intensity, in going from the isolated molecule to the 1-propanol cluster, just as observed modes show large changes in intensity on changing the solvent from TEA to 1-propanol (Supplementary Table 3). Therefore, in principle, the calculations embody all features needed to interpret the experimental data.

## Conclusions

The experimental data fitting is done adopting a Huang-Rhys model (Huang et al., 1950). This assumes that just two electronic states are involved, that the Born-Oppenheimer (Born and Oppenheimer, 1927) and Franck-Condon (Condon, 1928) approximations hold, that the potentials-energy surfaces are harmonic, that the ground- and excited-state vibrational frequencies are identical, and that the Duschinsky rotation matrix (Duschinsky, 1937) is the unit matrix, yielding vibrational frequencies ν_*i*_, Huang-Rhys factors *S*_*i*_, and associated reorganization energies *h*ν_*i*_*S*_*i*_ for each state. Unfortunately, this analysis is lacking in that it allows different vibrational frequencies to be determined for each state yet is internally based on the assumption that the same vibrational frequencies occur in each state. To resolve this paradox, the experimental data is augmented with the Duschinsky rotation matrices from the DFT calculations, providing links between the observed modes in absorption and those in emission. The simulated spectra therefore correspond to a modified Huang-Rhys model, in which the initial-state vibrational frequency is taken to be the average initial-state frequency, as weighted by the Duschinsky-matrix elements pertaining to each excited-state frequency (Reimers, 2001). A feature of the calculations, which is sometimes critical and sometimes not, is the use of a harmonic potential approximation in curvilinear coordinates to take into account the often very large anharmonic effects operative in large molecules (Reimers, 2001). The net result is that each intense high-resolution line in absorption is mapped onto an absorption mode predicted by the calculations. This mapping is accomplished by: (1) assigning a key observed line in absorption to lines predicted in the calculations, (2) applying the calculated Duschinsky matrix to the predicted line onto one or more lines calculated in emission, and (3) assigning the predicted emission line(s) onto an observed emission line(s). This allows the observed high-resolution absorption-emission asymmetry to be understood.

Theoretical prediction of absorption and emission spectra, for a molecule the size of chlorophyll, to the accuracy needed to interpret either low-resolution or high-resolution spectra, remains a serious challenge. Required is the determination of the optimized geometry, vibrational frequencies, and associated normal modes of vibration in both the ground and excited electronic states. The only readily applicable approach of reasonable accuracy is DFT, applied using its time-dependent formalism (Casida, 1995) (TD-DFT) to model excited states. One needs to choose between a wide variety of available density functionals, however, with a desired outcome being that any sensible choice leads to the same basic qualitative conclusions. Also, previous similar studies for BChl-a and Pheo-a indicate that different modern approaches can predict results showing great disparity, with most not even qualitatively depicting the basic observed absorption and emission spectral properties (Rätsep et al., 2011, 2019b). A reason for this is that a critical property responsible for the usefulness of the chlorophyllides in light harvesting, transport, and energy conversion is the very low value of their emission and absorption reorganization energies, resulting in difficult quantitative calculations that portray related properties. The only satisfactory density functional so far identified is CAM-B3LYP (Yanai et al., 2004; Kobayashi and Amos, 2006); the conclusion confirmed also here in case of Chl-a. This is a functional embodying long-range correction of the potential so as to be able to treat charge-transfer states, without which the lowest-energy non-charge transfer states like *Q*_*y*_ and *Q*_*x*_ are poorly represented (Cai et al., 2002, 2006; Magyar and Tretiak, 2007; Peach et al., 2008).

While the highly environment sensitive contribution of the *Q*_x_ state to absorption has long been known as a major source of asymmetry between observed low-resolution absorption and emission spectra of Chl-a, the measurement before (Rätsep et al., 2009a) and herein of phonon-sideband-free high resolution emission spectra using ΔFLN, to complement existing high-resolution absorption spectra (Avarmaa and Rebane, 1985), indicates that significant asymmetry remains even after the effects (Reimers et al., 2013) of *Q*_x_ absorption are negated. Further, measuring ΔFLN in two different solvents, TEA which results in 5-coordinate magnesium, and 1-propanol, with the coordination increasing to 6, reveals strong solvent-dependence for vibrational line intensities. With the exception of vibrational modes involved in specific solvation effects, only small changes in vibrational frequency are found to accompany these large changes in line intensity. Also, as found for BChl-a (Rätsep et al., 2011) and Pheo-a (Rätsep et al., 2019b), the most intense vibrational lines observed in either one of absorption or emission can be absent in the other spectroscopic results, indicating that absorption-emission asymmetry arises from fundamental changes in the vibrational descriptions of the *S*_0_ and *Q*_*y*_ states.

The large absorption-emission asymmetry, as well as the strong solvent dependence of the emission line strengths, is attributed to properties of the Duschinsky rotation matrix that maps the form of the vibrational motions in *Q*_*y*_ onto those of *S*_0_. Four distinct types of properties are predicted and correlated with experimental observations: (1) some modes retain their form in the two electronic states, leading to high-resolution absorption/emission symmetry; (2) some modes mix strongly with just a few other modes of similar frequency, making line intensities strongly dependent on subtle solvent-induced changes in frequency of the coupled modes, as well as providing significant absorption/emission asymmetry; (3) some modes mix very strongly, so strong that dominant lines in either absorption or emission have no counterpart; and (4) strong mixing, particularly for key high-frequency modes, can result in absorption at one vibrational frequency, without phase loss or energy relaxation, producing emission spanning over a thousand cm^−1^.

These results are particularly pertinent to computational modes describing exciton transport in photosystems. If vibrational relaxation occurs before reemission, then quantum coherence of the energy-transport process is lost, reducing the process to one of classical kinetics, with re-emission expected over the broad allowed range. If there is no vibrational relaxation and full absorption-emission asymmetry, then energy is simply stored on a chromophore and then passed on coherently. The modeling of exciton transport usually involves understanding the competition between these processes, competition between coherent and incoherent transport mechanisms. A new dimension to this is demanded by the results obtained: a chromophore that absorbs an exciton at a specific energy can also coherently reemit it, in a very mode-specific way, over a wide energy range. Energy change and decoherence are therefore no longer intrinsically coupled.

Finally, we note that the calculations embody all effects needed for a detailed understanding of the effects of solvent on high-resolution spectroscopic properties, but still fail to qualitatively describe the actual observed effects. It is likely that large-sample modern treatments that fully solvate the chromophore with explicitly represented solvent molecules, combined with inclusion of long-range dielectric properties (Hush and Reimers, 2000; Skyner et al., 2015; Zuehlsdorff and Isborn, 2018; Cerezo et al., 2020), are required for further progress.

## Data Availability Statement

The original contributions presented in the study are included in the article/Supplementary Material, further inquiries can be directed to the corresponding author.

## Author Contributions

JR designed and performed the calculations and spectral data fitting. MR performed the sample preparation and experimental measurements. AF designed the research. All authors contributed to the manuscript.

## Conflict of Interest

The authors declare that the research was conducted in the absence of any commercial or financial relationships that could be construed as a potential conflict of interest.

## Supplementary Material

Supplementary Material provided for (i) additional results tables, (ii) data files providing the optimized coordinates **x**, vibrational frequencies **ν**, and normal modes **C** of methyl Chl-a porphyrin in its ground state *Q*_y_ state, as well as the associated Duschinsky matrix **D** and curvilinear projections **δ**^A^ and **δ**^E^ of the ground-to-excited state geometry changes onto the two sets of normal modes, and the associated reorganization energies, and (iii) pdf files showing pictorially the form of the ground-state and excited-state normal modes.
