# Supplementary material for: Asymmetry in the Qy Fluorescence and Absorption Spectra of Chlorophyll a Pertaining to Exciton Dynamics
Source: Front Chem. 2020 Dec 2;8:588289. doi: 10.3389/fchem.2020.588289 (PMC7738624; doi:10.3389/fchem.2020.588289)
Supplement: Supplementary file 2 [file Data_Sheet_2.PDF]

In this file, the forms of the normal modes are depicted graphically. For each mode is listed:

- Vibration frequency ν, in cm^{-1}.
- Reorganization energy in absorption, λ^A, in cm^{-1}.
- ^{15}N is the sensitivity of this vibration to N isotopic substitution. The isotopically substituted frequency differs by $\nu \times$ the shown sensitivity / 1000.
- ^{26}Mg is the sensitivity of this vibration to Mg isotopic substitution. The isotopically substituted frequency differs by $\nu \times$ the shown sensitivity / 1000.
- The percentage of the motion that is in the *xy* plane (ca. the macrocyclic plane).
- The percentage of the motion that is in the *z* direction (ca. normal to the macrocyclic plane).
- The percentages of the motion describable as bond stretches (S), bond-angle bends (B), and bond-angle torsions (T). Key motions are shown in different colours for clarity.

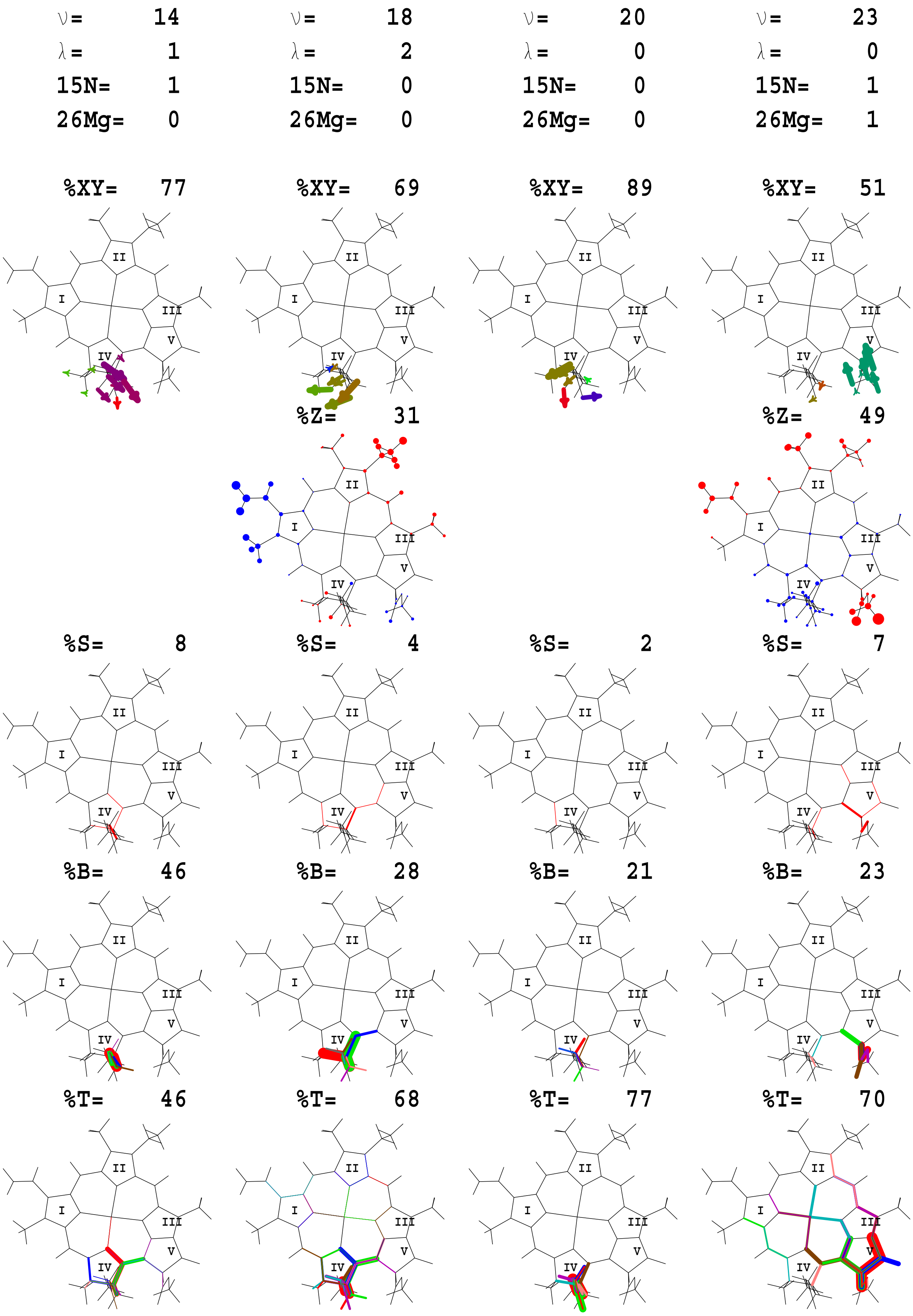

ν= 14
λ= 1
15N= 1
26Mg= 0
%XY= 77
%S= 8
%B= 46
%T= 46
ν= 18
λ= 2
15N= 0
26Mg= 0
%XY= 69
%Z= 31
%S= 4
%B= 28
%T= 68
ν= 20
λ= 0
15N= 0
26Mg= 0
%XY= 89
%S= 2
%B= 21
%T= 77
ν= 23
λ= 0
15N= 1
26Mg= 1
%XY= 51
%Z= 49
%S= 7
%B= 23
%T= 70
I
II
III
IV
V

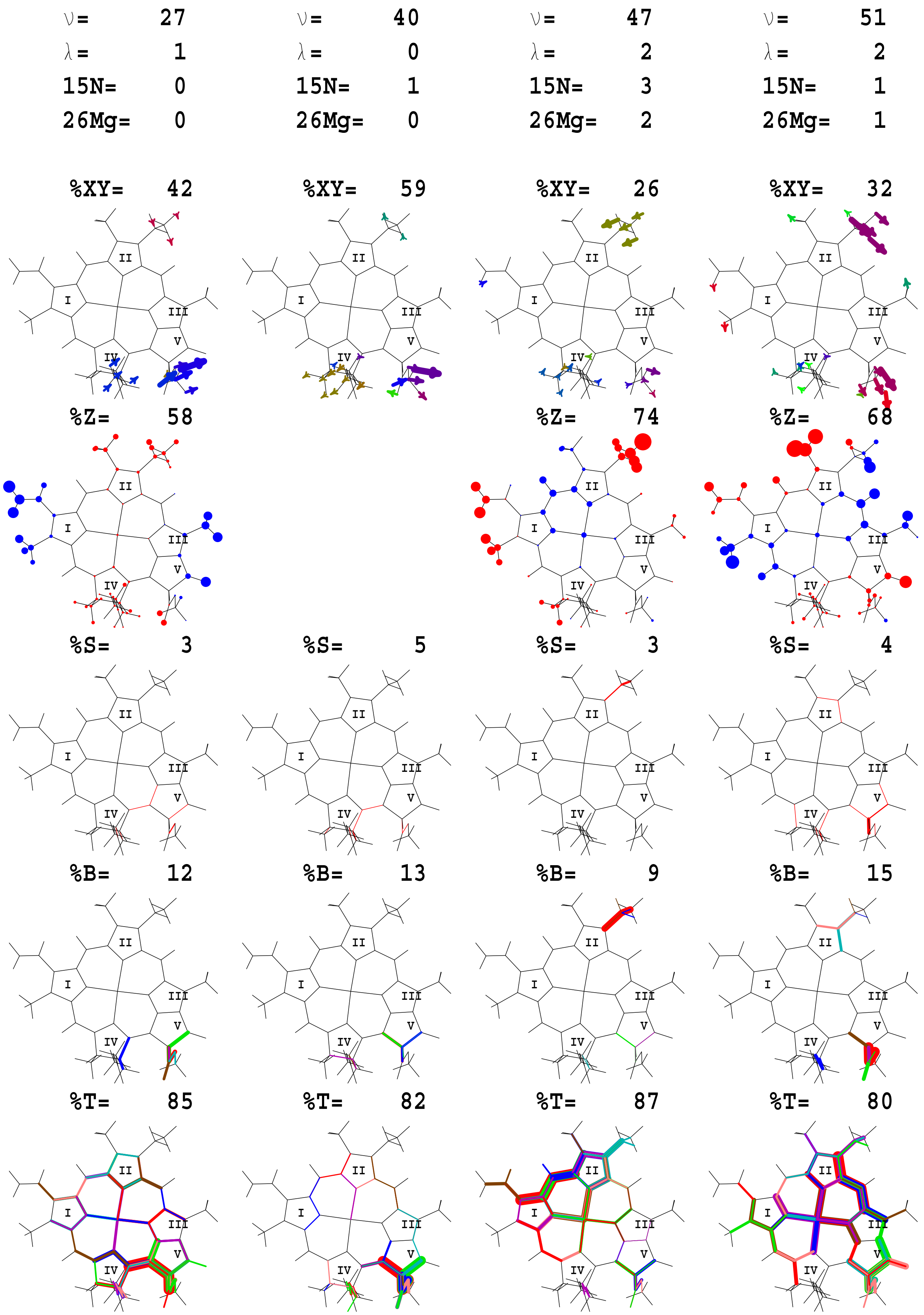

ν= 27
λ= 1
15N= 0
26Mg= 0
ν= 40
λ= 0
15N= 1
26Mg= 0
ν= 47
λ= 2
15N= 3
26Mg= 2
ν= 51
λ= 2
15N= 1
26Mg= 1
%XY= 42
%XY= 59
%XY= 26
%XY= 32
%Z= 58
%Z= 74
%Z= 68
%S= 3
%S= 5
%S= 3
%S= 4
%B= 12
%B= 13
%B= 9
%B= 15
%T= 85
%T= 82
%T= 87
%T= 80
I
II
III
IV
V

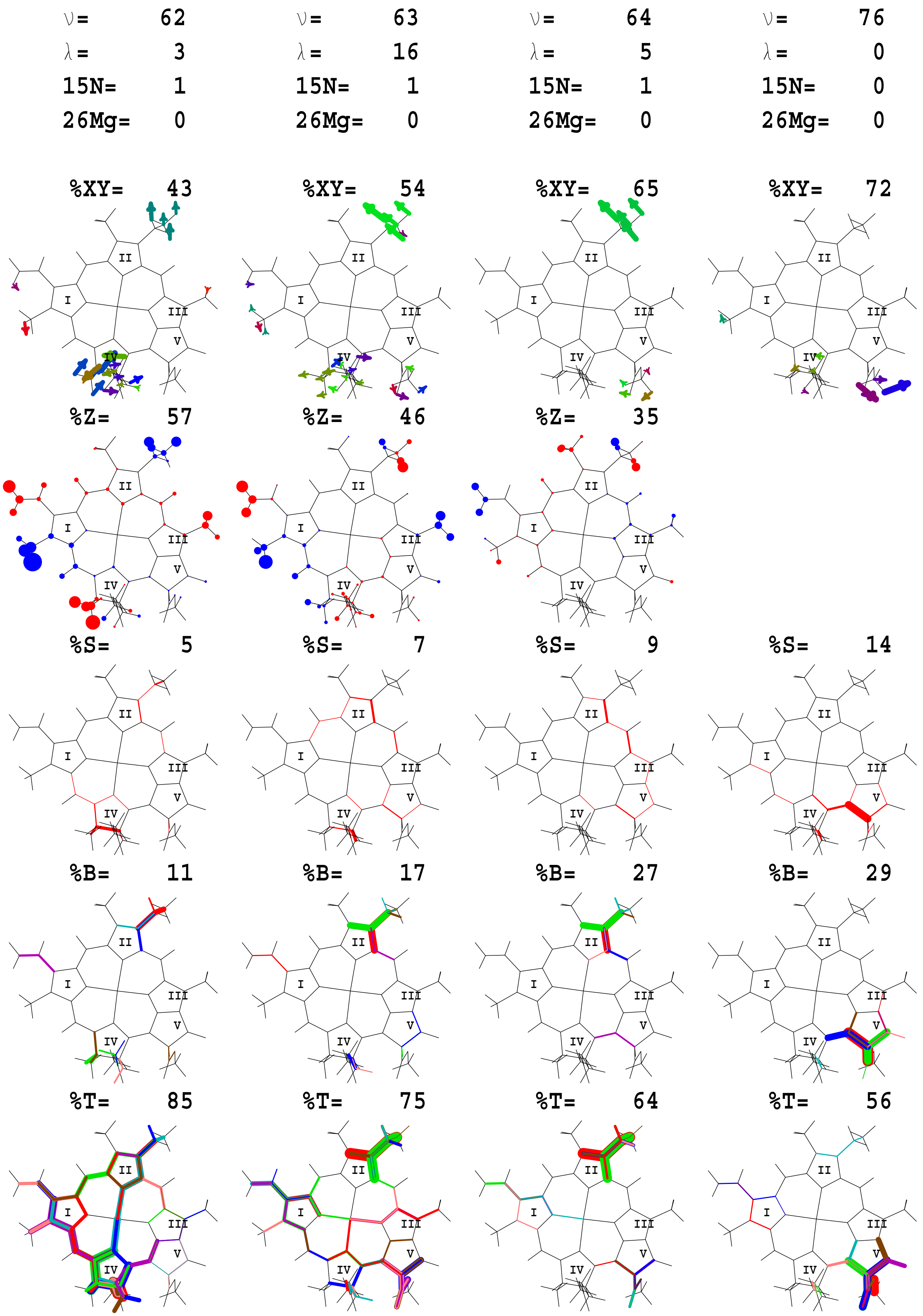

ν= 62
λ= 3
15N= 1
26Mg= 0
ν= 63
λ= 16
15N= 1
26Mg= 0
ν= 64
λ= 5
15N= 1
26Mg= 0
ν= 76
λ= 0
15N= 0
26Mg= 0
%XY= 43
%XY= 54
%XY= 65
%XY= 72
%Z= 57
%Z= 46
%Z= 35
%S= 5
%S= 7
%S= 9
%S= 14
%B= 11
%B= 17
%B= 27
%B= 29
%T= 85
%T= 75
%T= 64
%T= 56
I
II
III
IV
V

ν=	82	96	99	106
λ=	0	4	3	1
15N=	2	2	2	2
26Mg=	2	0	0	0

%XY=	28	33	50	58

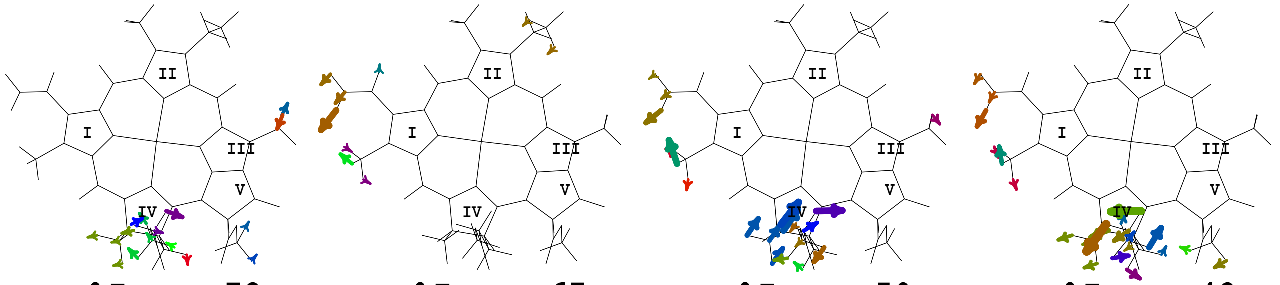

%Z=	72	67	50	42

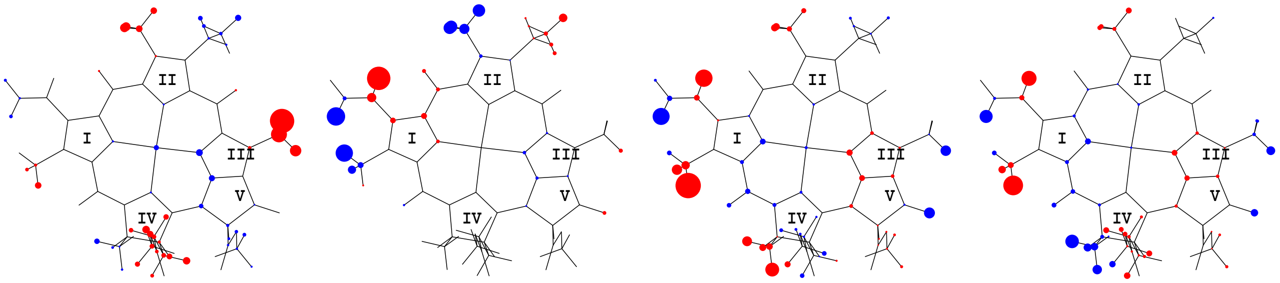

%S=	11	5	11	9

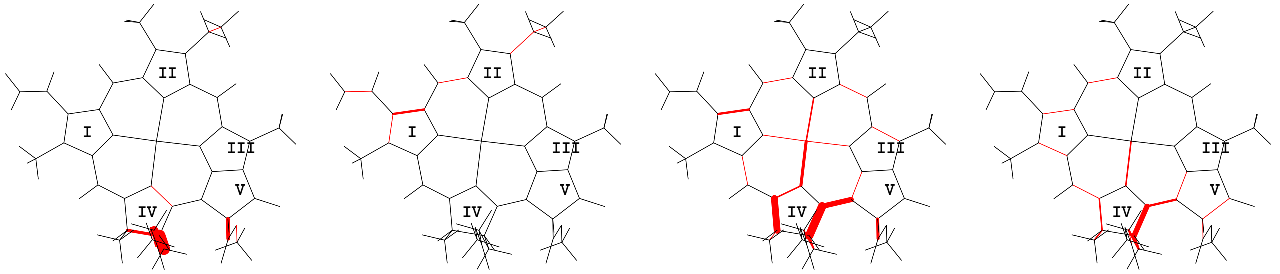

%B=	19	17	12	18

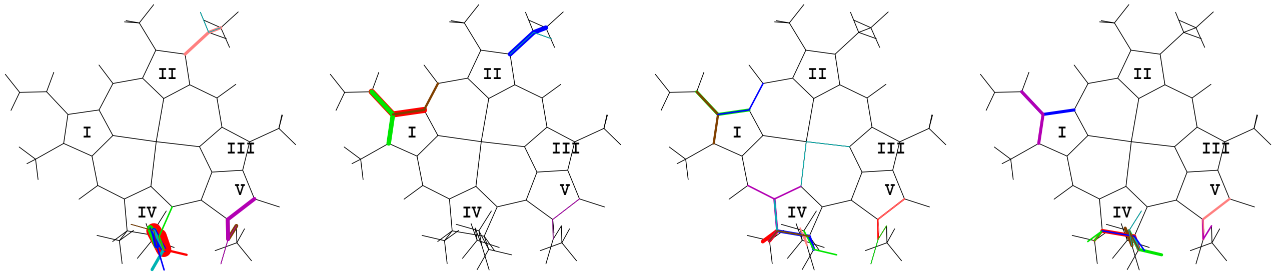

%T=	71	78	76	73

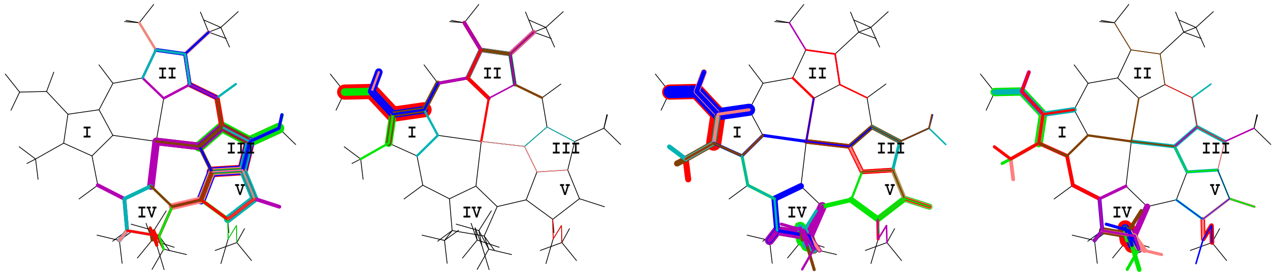

ν= 108	ν= 118	ν= 122	ν= 126
λ= 1	λ= 1	λ= 0	λ= 0
15N= 4	15N= 0	15N= 1	15N= 2
26Mg= 0	26Mg= 0	26Mg= 0	26Mg= 0

%XY= 30	%XY= 51	%XY= 54	%XY= 51

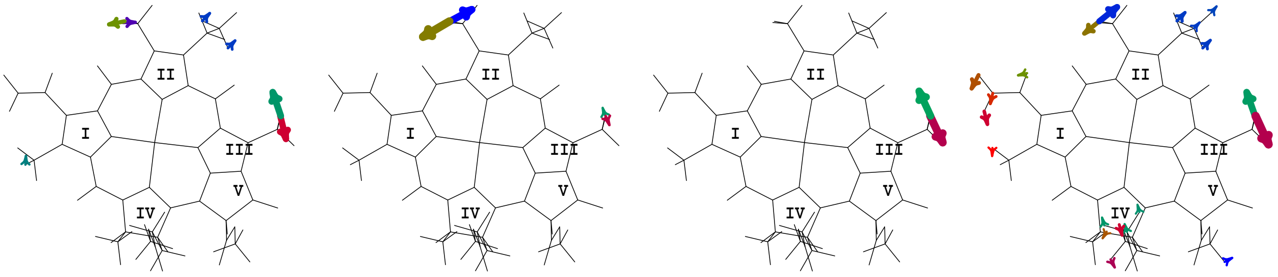

%Z= 70	%Z= 49	%Z= 46	%Z= 49

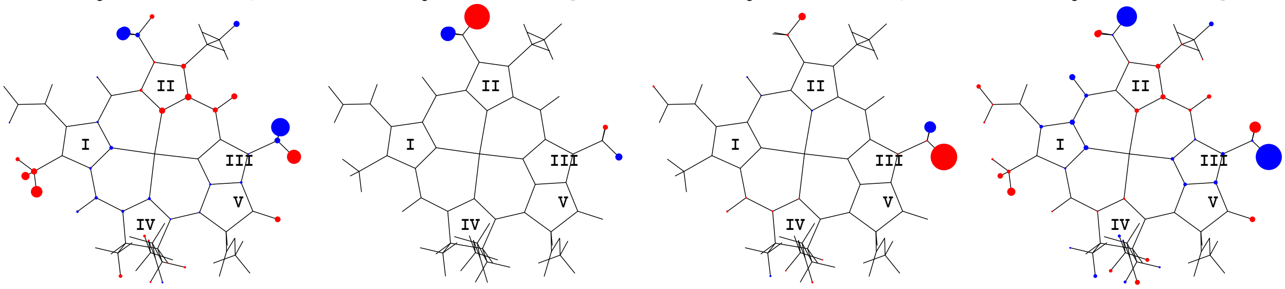

%S= 2	%S= 1	%S= 6	%S= 9

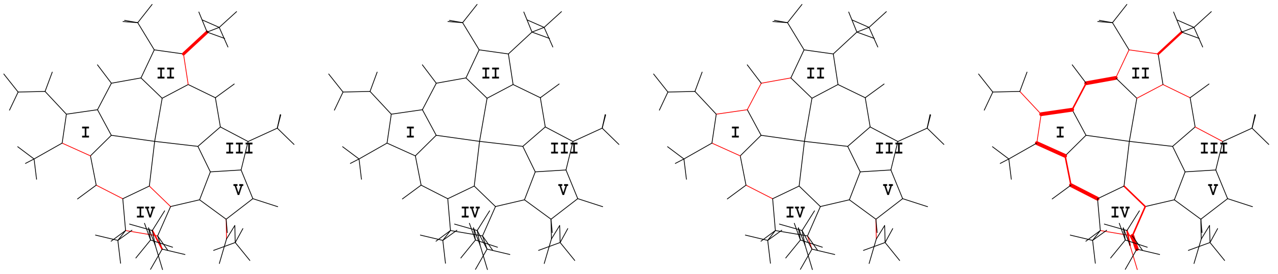

%B= 14	%B= 4	%B= 21	%B= 29

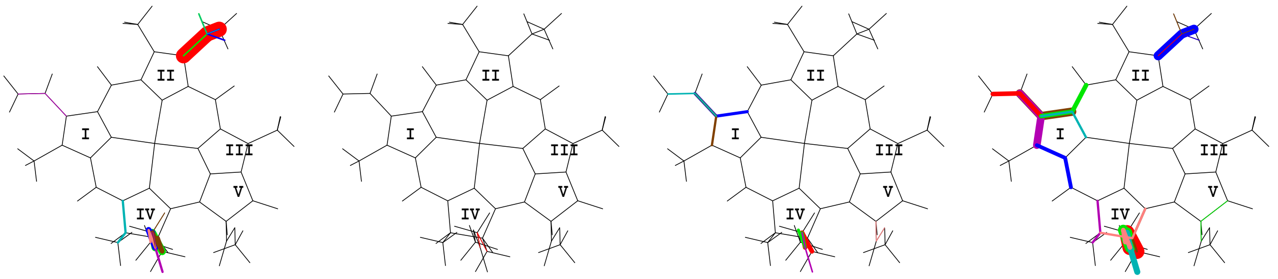

%T= 84	%T= 95	%T= 73	%T= 61

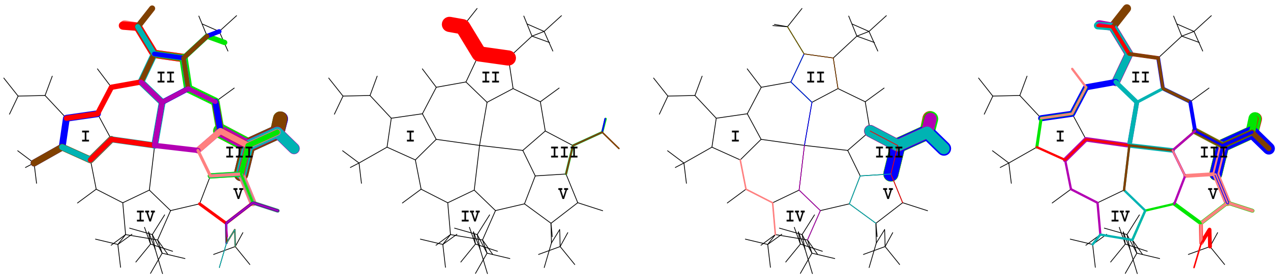

ν=	135	139	144	145
λ=	3	0	1	0
15N=	1	1	0	4
26Mg=	0	0	0	1

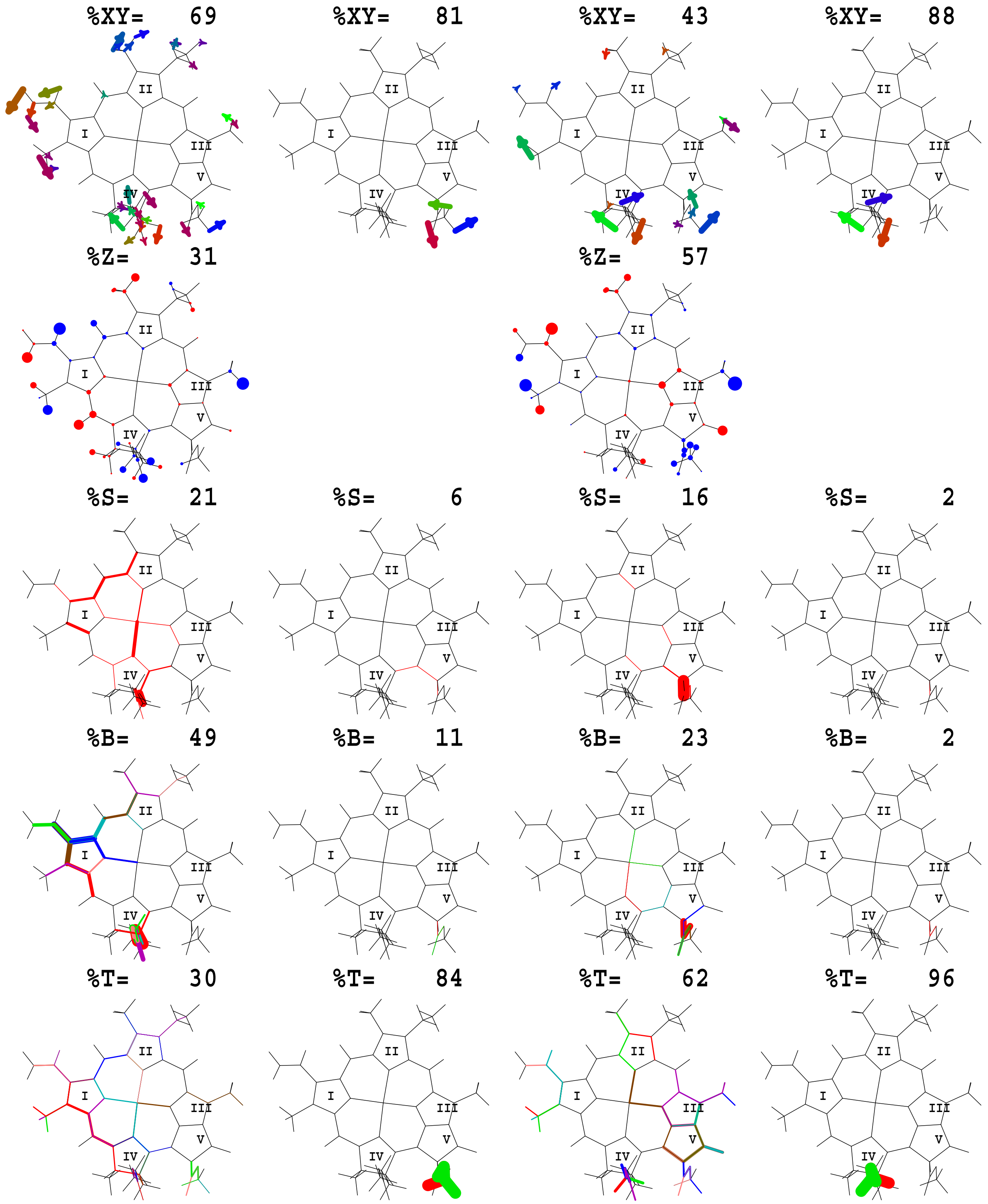

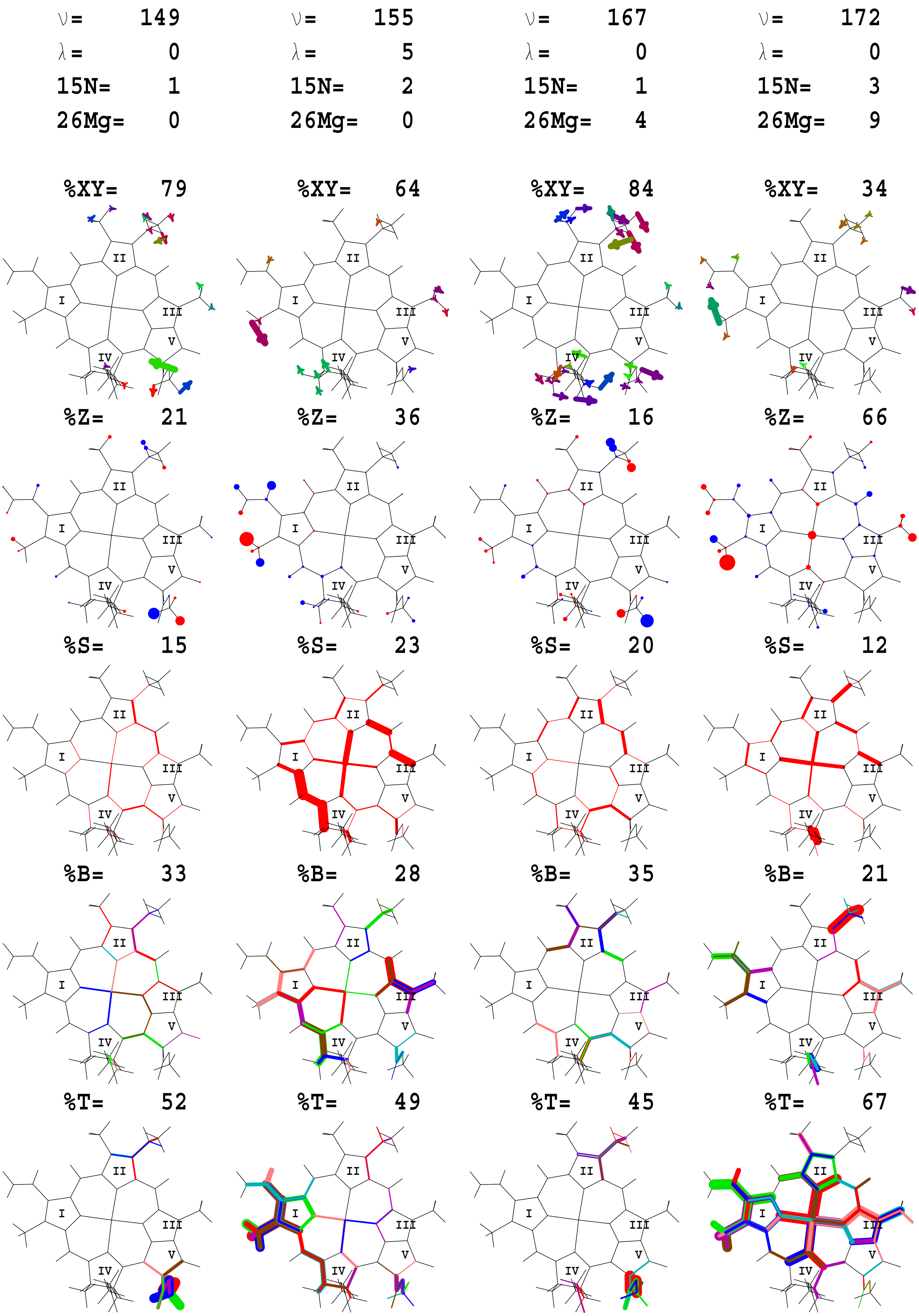

ν= 149
λ= 0
15N= 1
26Mg= 0
%XY= 79
%Z= 21
%S= 15
%B= 33
%T= 52
ν= 155
λ= 5
15N= 2
26Mg= 0
%XY= 64
%Z= 36
%S= 23
%B= 28
%T= 49
ν= 167
λ= 0
15N= 1
26Mg= 4
%XY= 84
%Z= 16
%S= 20
%B= 35
%T= 45
ν= 172
λ= 0
15N= 3
26Mg= 9
%XY= 34
%Z= 66
%S= 12
%B= 21
%T= 67
I
II
III
IV
V

ν=	178	183	187	191
λ=	0	0	0	1
15N=	0	4	2	2
26Mg=	0	2	0	1
%XY=	89	69	72	74
%Z=	11	31	28	26
%S=	2	34	17	22
%B=	6	29	40	43
%T=	92	37	43	34

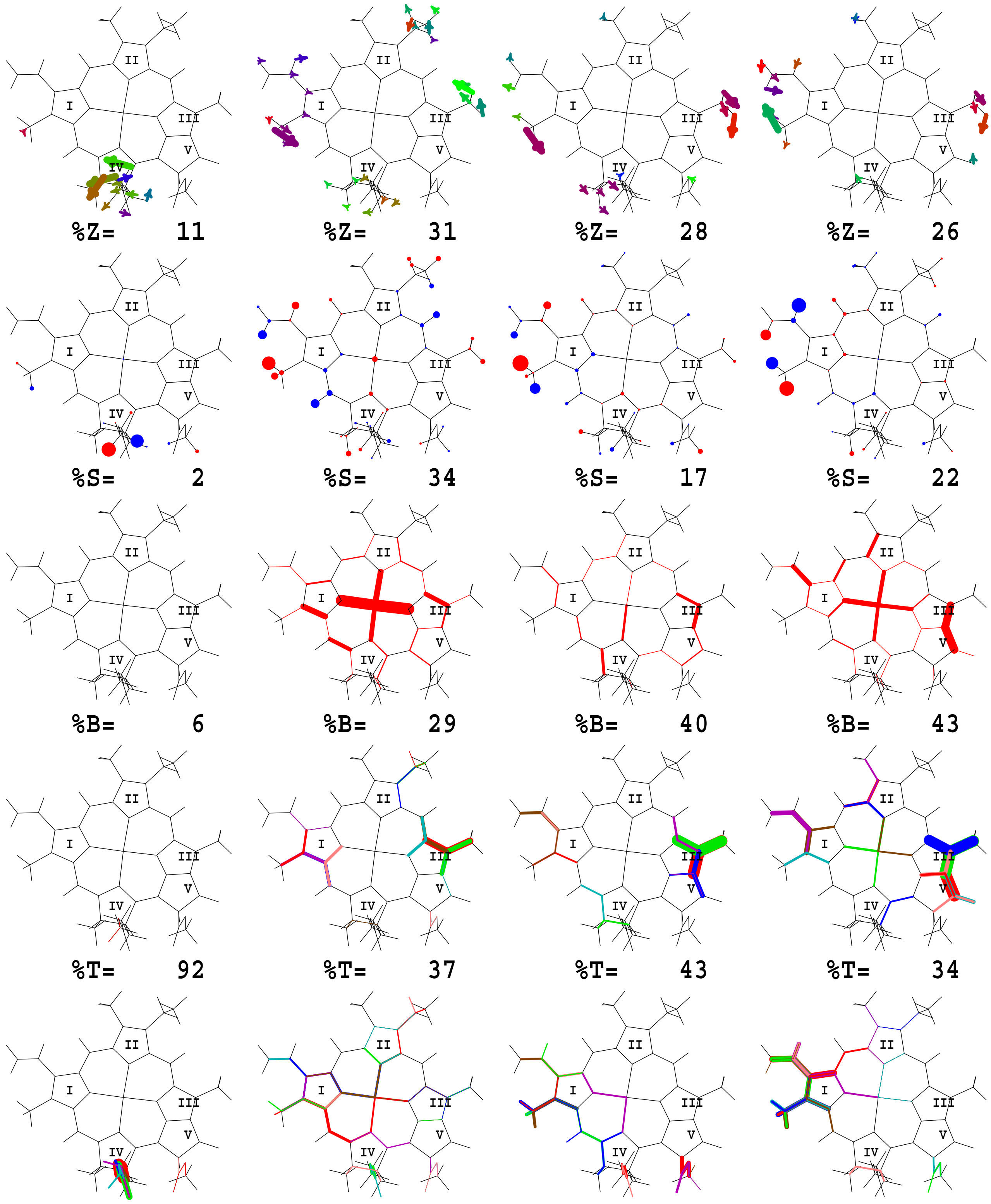

ν=	197	202	210	221
λ=	0	0	1	1
15N=	2	4	3	1
26Mg=	1	3	2	0

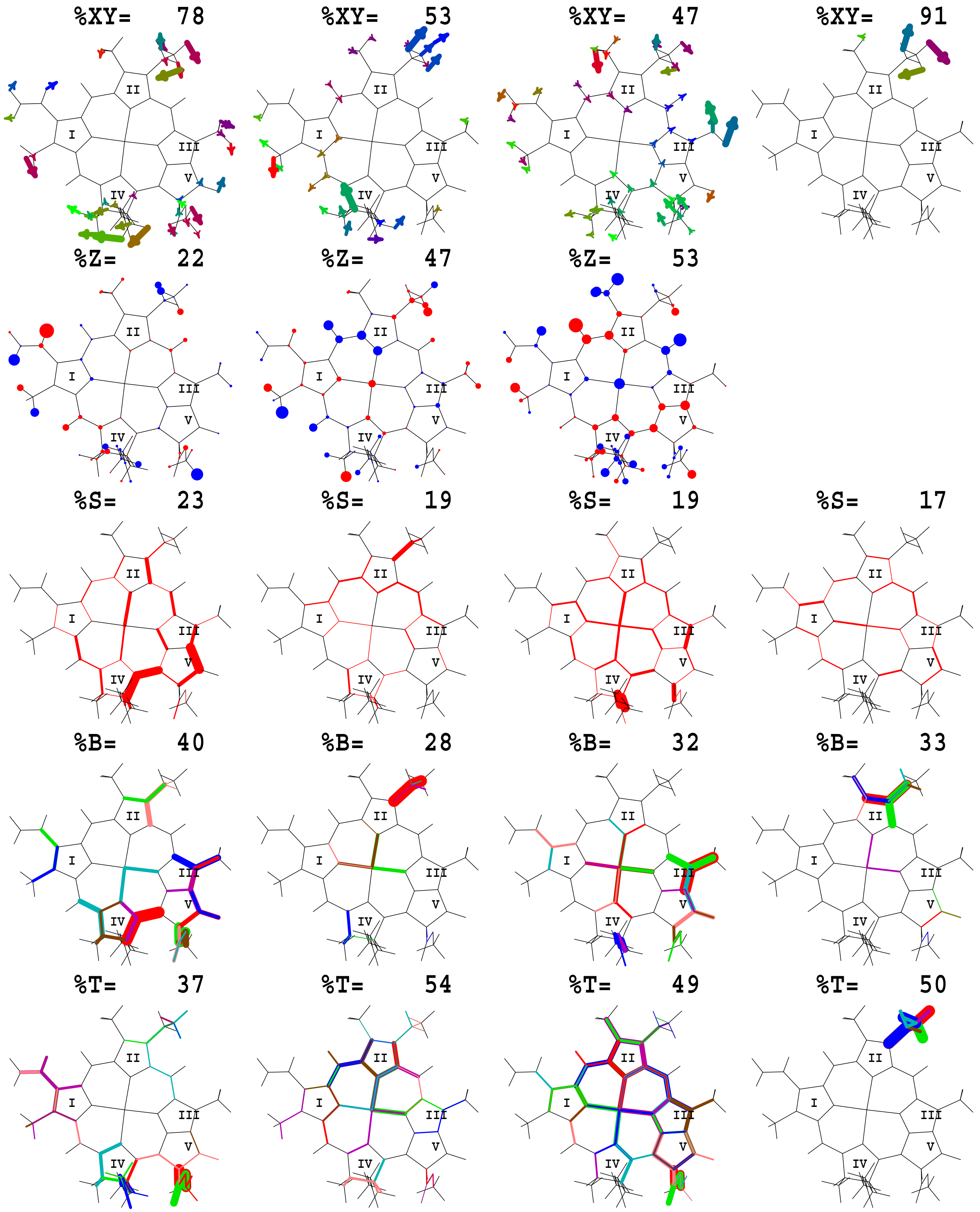

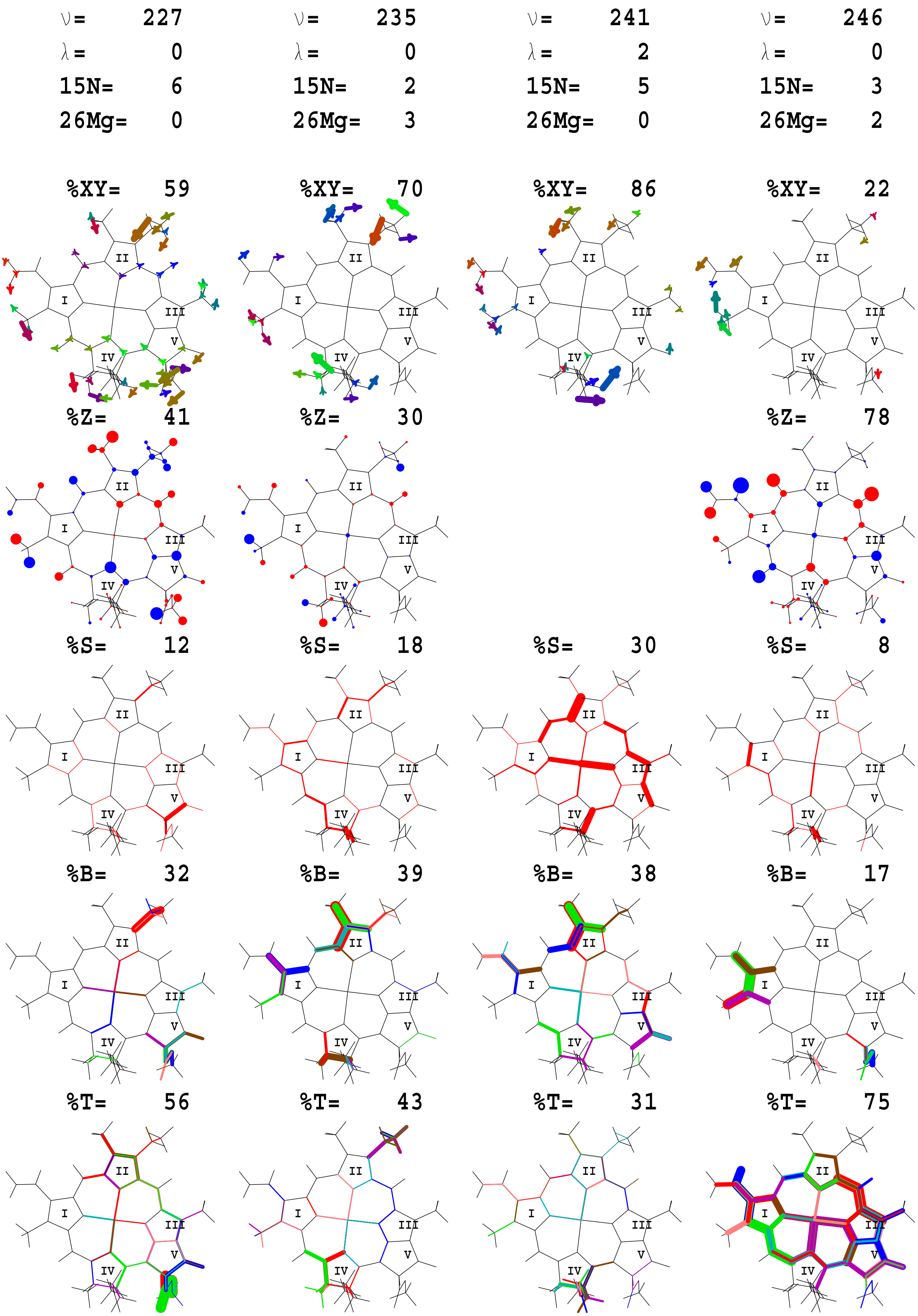

ν= 227
λ= 0
15N= 6
26Mg= 0
%XY= 59
%Z= 41
%S= 12
%B= 32
%T= 56
ν= 235
λ= 0
15N= 2
26Mg= 3
%XY= 70
%Z= 30
%S= 18
%B= 39
%T= 43
ν= 241
λ= 2
15N= 5
26Mg= 0
%XY= 86
%S= 30
%B= 38
%T= 31
ν= 246
λ= 0
15N= 3
26Mg= 2
%XY= 22
%Z= 78
%S= 8
%B= 17
%T= 75
I
II
III
IV
V

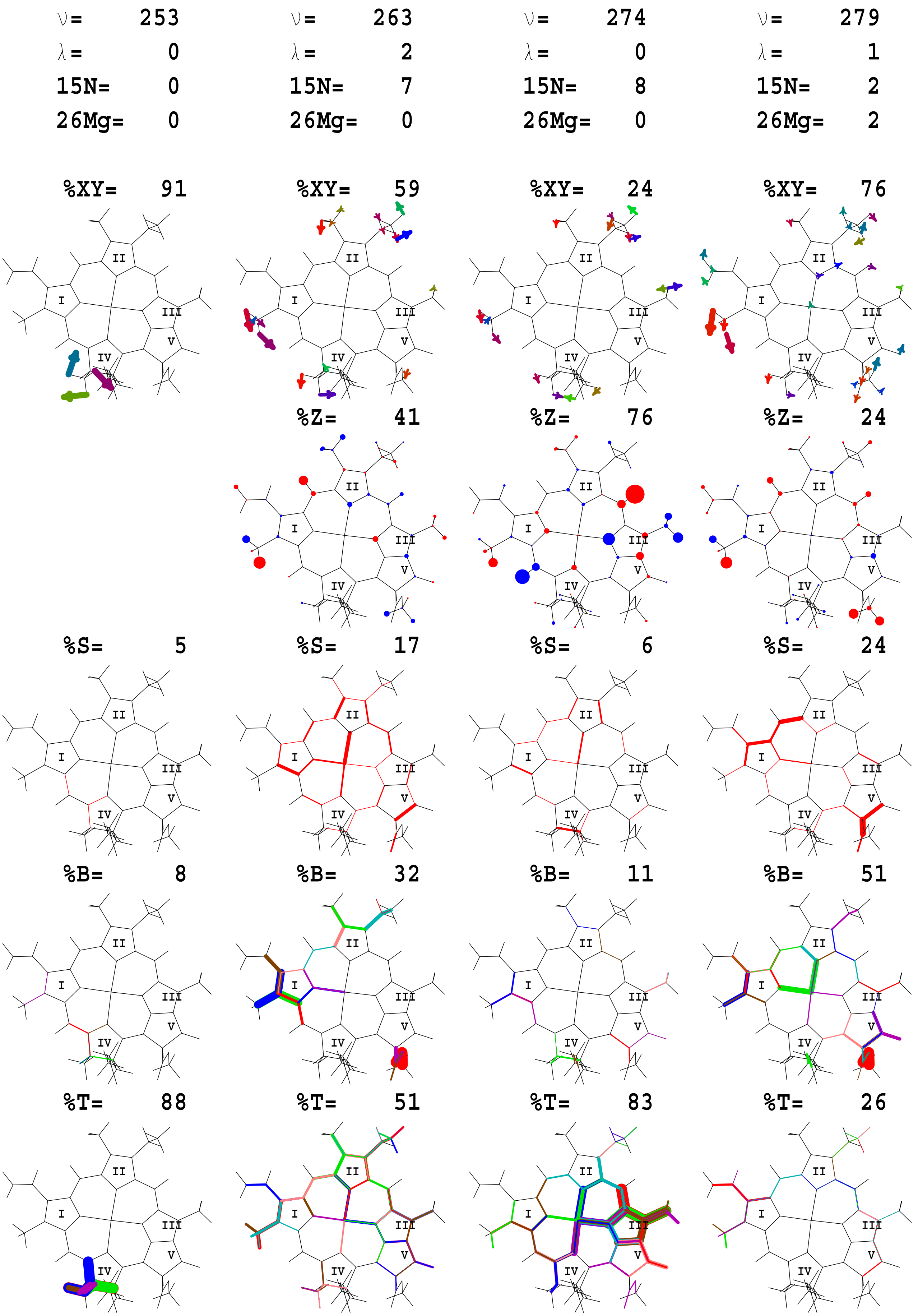
ν= 253
λ= 0
15N= 0
26Mg= 0
ν= 263
λ= 2
15N= 7
26Mg= 0
ν= 274
λ= 0
15N= 8
26Mg= 0
ν= 279
λ= 1
15N= 2
26Mg= 2
%XY= 91
%XY= 59
%XY= 24
%XY= 76
%Z= 41
%Z= 76
%Z= 24
%S= 5
%S= 17
%S= 6
%S= 24
%B= 8
%B= 32
%B= 11
%B= 51
%T= 88
%T= 51
%T= 83
%T= 26
I
II
III
IV
V

ν= 286	ν= 292	ν= 297	ν= 301
λ= 1	λ= 1	λ= 0	λ= 0
15N= 3	15N= 7	15N= 7	15N= 2
26Mg= 1	26Mg= 2	26Mg= 1	26Mg= 0

%XY= 61	%XY= 52	%XY= 44	%XY= 35

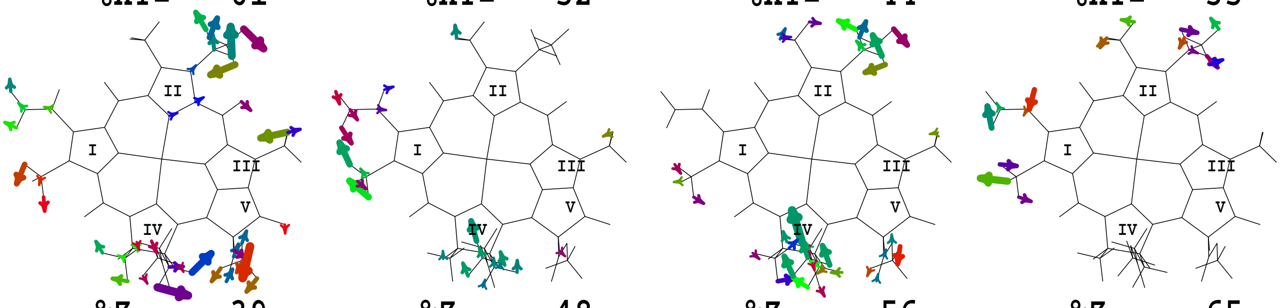

%Z= 39	%Z= 48	%Z= 56	%Z= 65

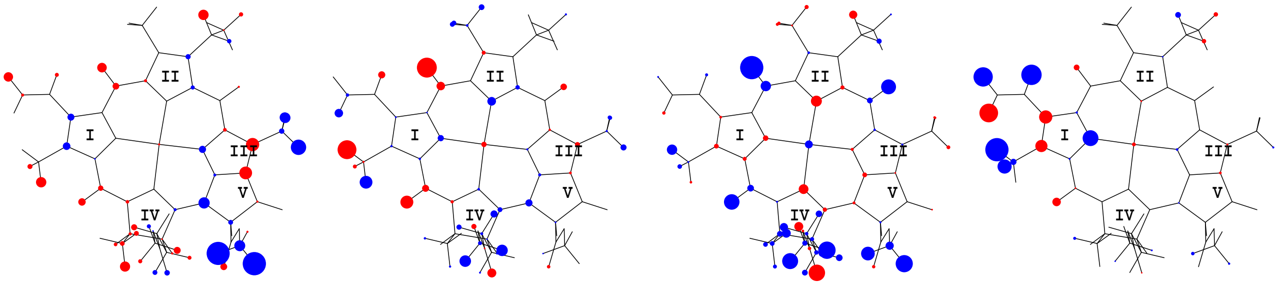

%S= 18	%S= 15	%S= 10	%S= 7

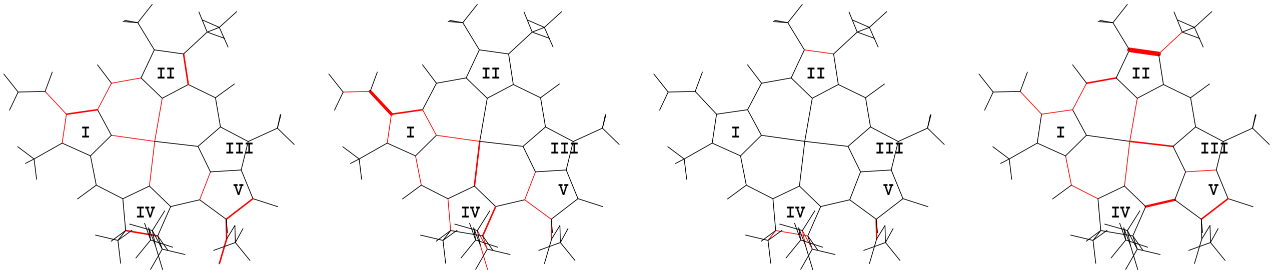

%B= 41	%B= 44	%B= 50	%B= 19

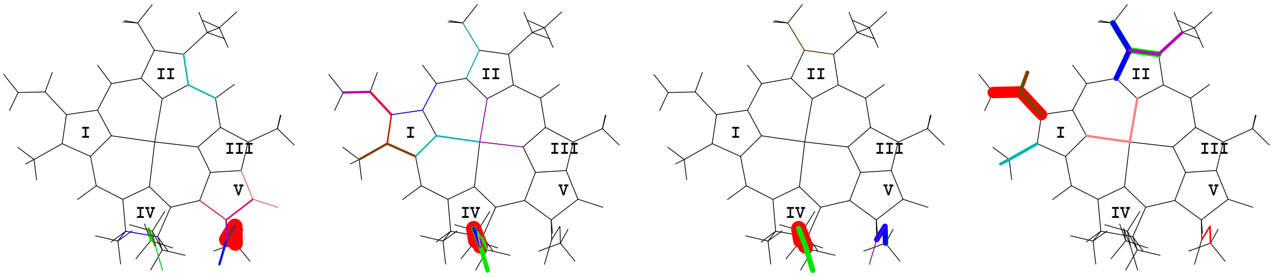

%T= 41	%T= 42	%T= 40	%T= 73

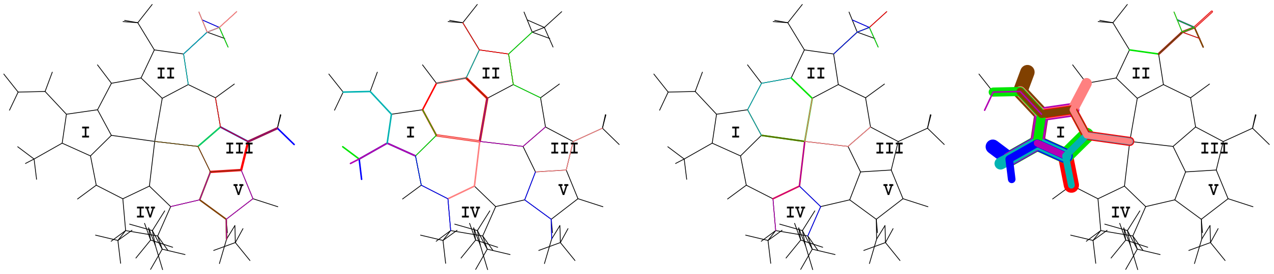

ν=	309	313	335	343
λ=	0	0	0	2
15N=	5	4	4	4
26Mg=	6	0	2	1

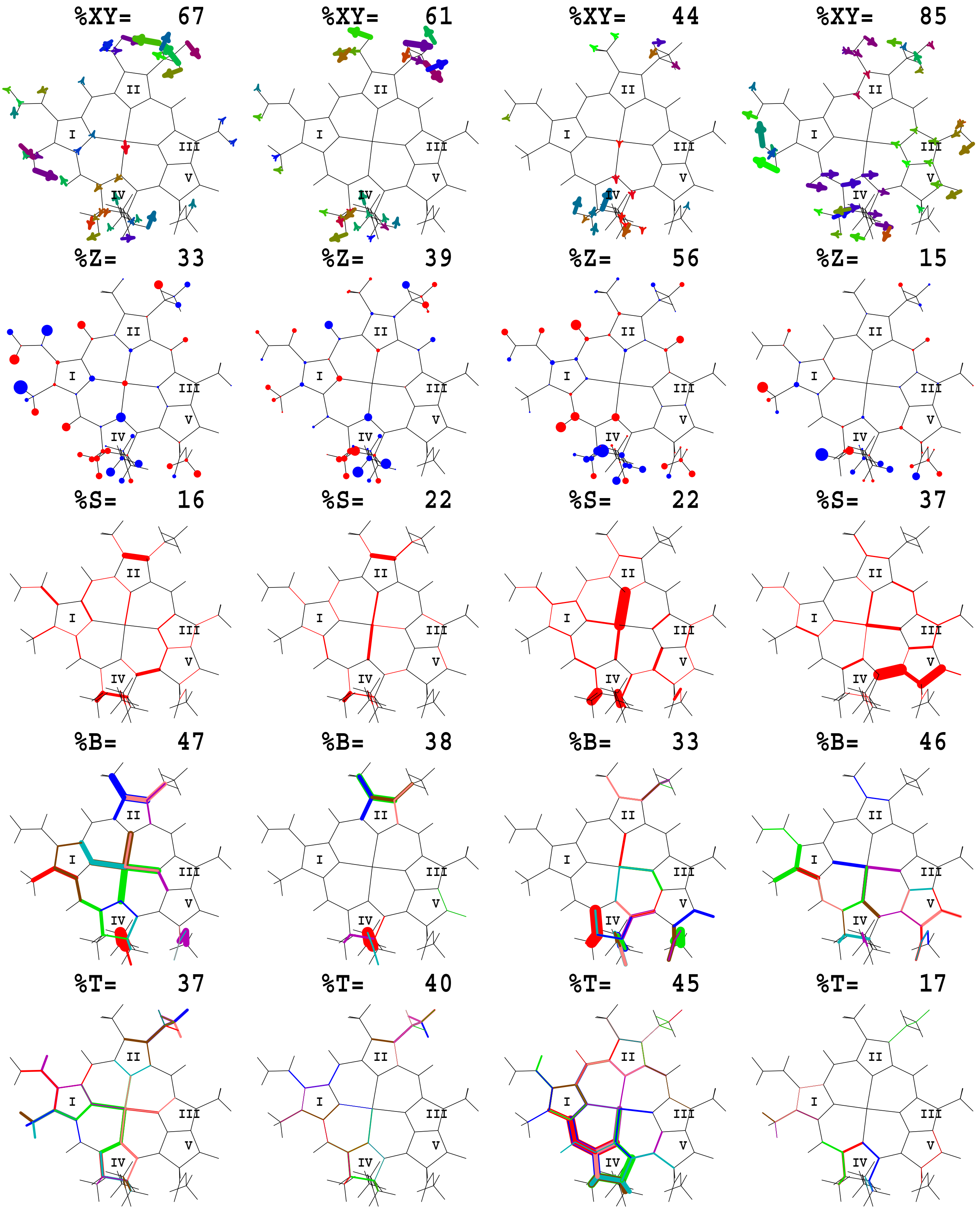

ν= 356	ν= 370	ν= 375	ν= 385
λ= 1	λ= 0	λ= 1	λ= 3
15N= 2	15N= 2	15N= 4	15N= 1
26Mg= 2	26Mg= 6	26Mg= 4	26Mg= 12

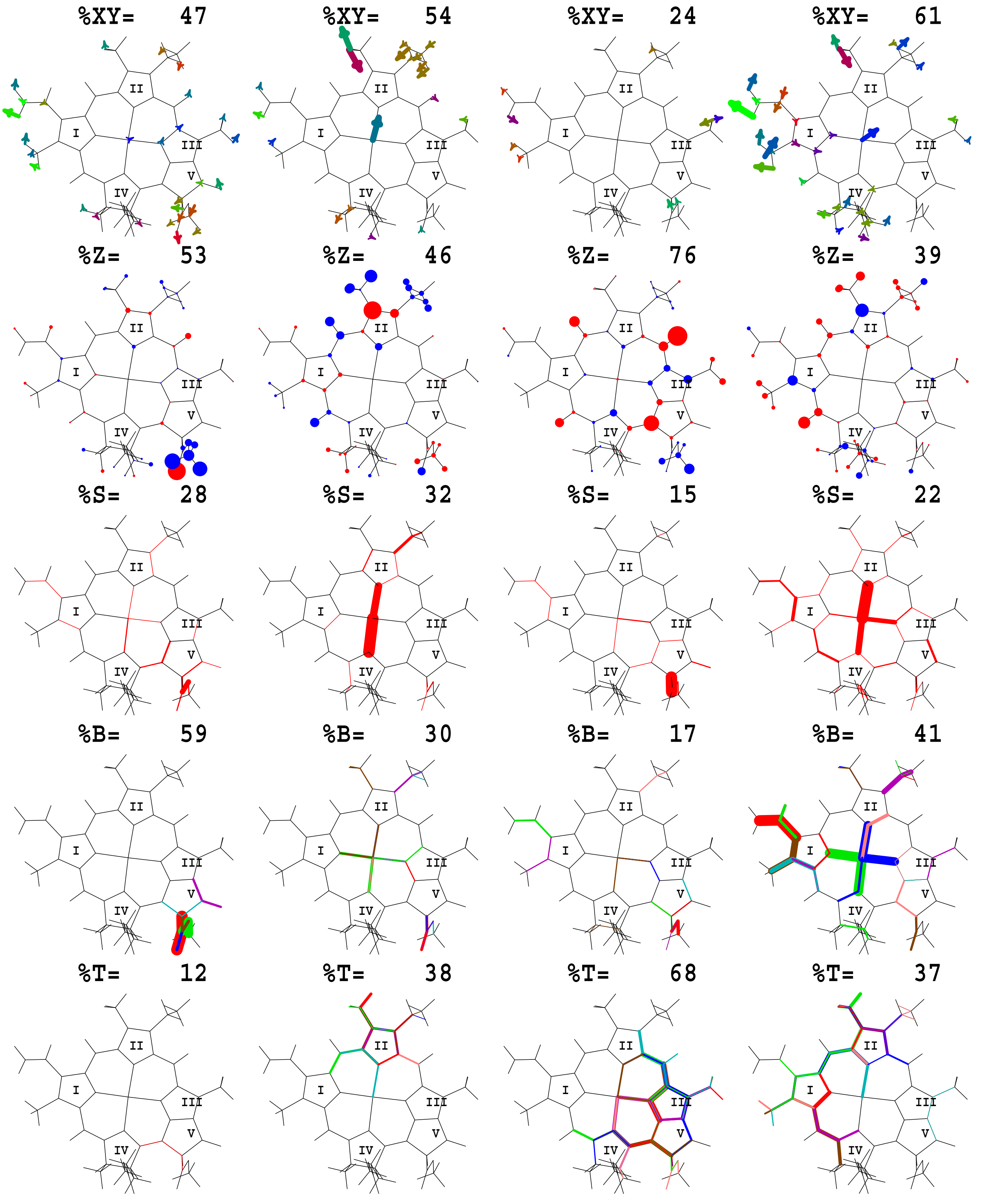

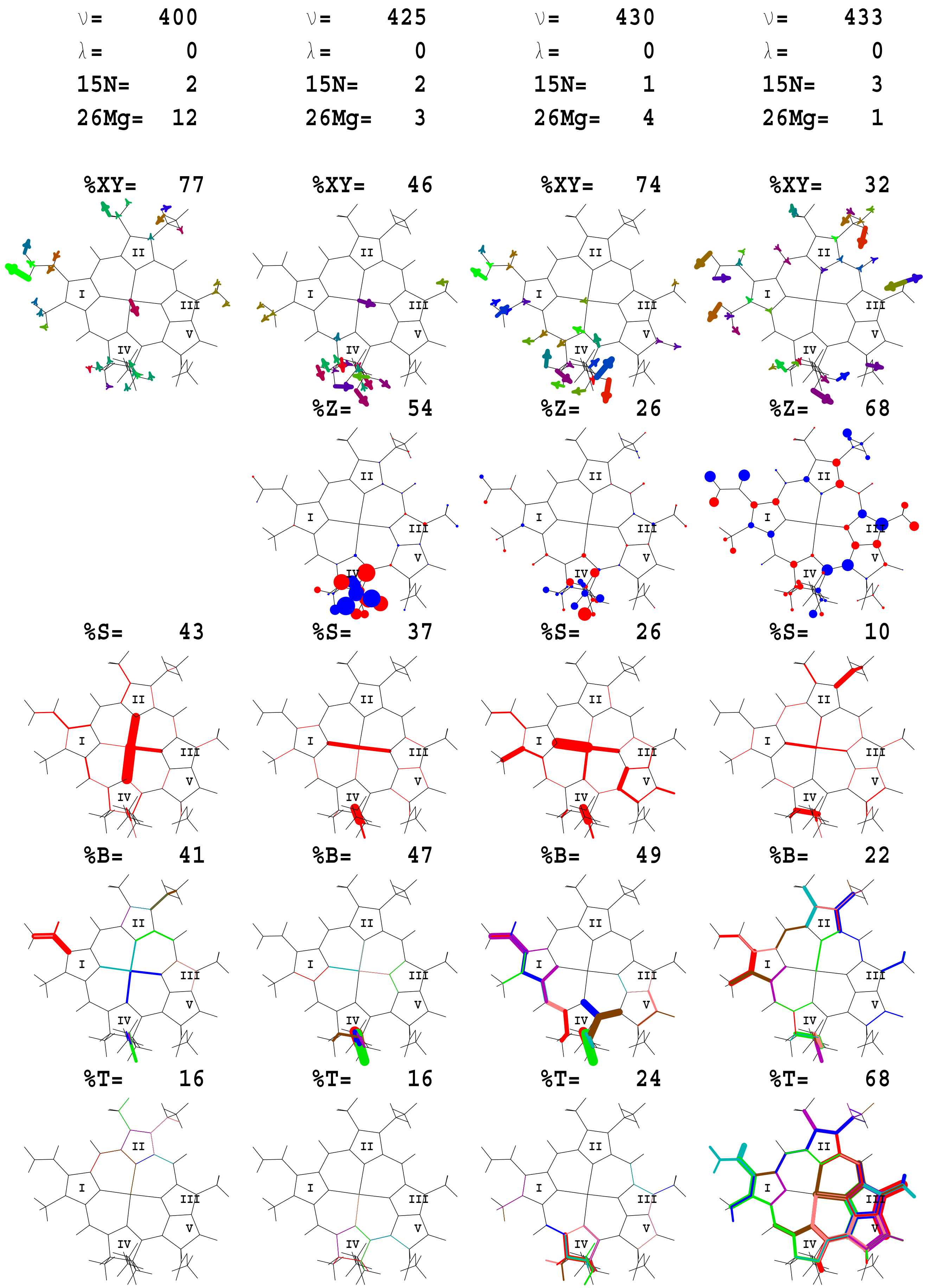

ν= 400
λ= 0
15N= 2
26Mg= 12
%XY= 77
%S= 43
%B= 41
%T= 16
ν= 425
λ= 0
15N= 2
26Mg= 3
%XY= 46
%Z= 54
%S= 37
%B= 47
%T= 16
ν= 430
λ= 0
15N= 1
26Mg= 4
%XY= 74
%Z= 26
%S= 26
%B= 49
%T= 24
ν= 433
λ= 0
15N= 3
26Mg= 1
%XY= 32
%Z= 68
%S= 10
%B= 22
%T= 68
I
II
III
IV
V

ν=	453	464	476	494
λ=	1	2	0	2
15N=	1	1	2	1
26Mg=	10	1	1	3
%XY=	89	57	59	73
%Z=	11	43	41	27
%S=	34	17	20	28
%B=	45	39	37	46
%T=	21	44	43	27

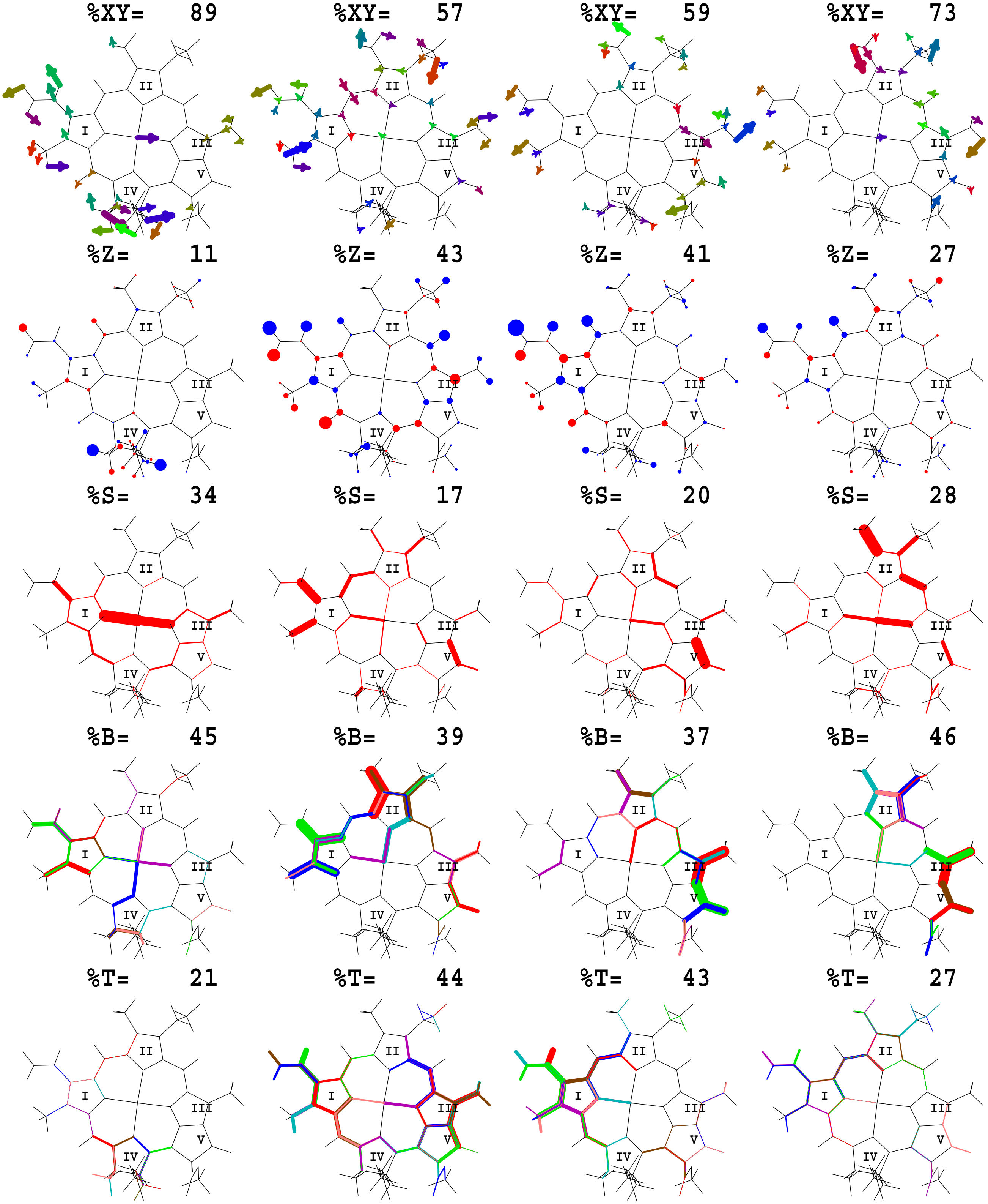

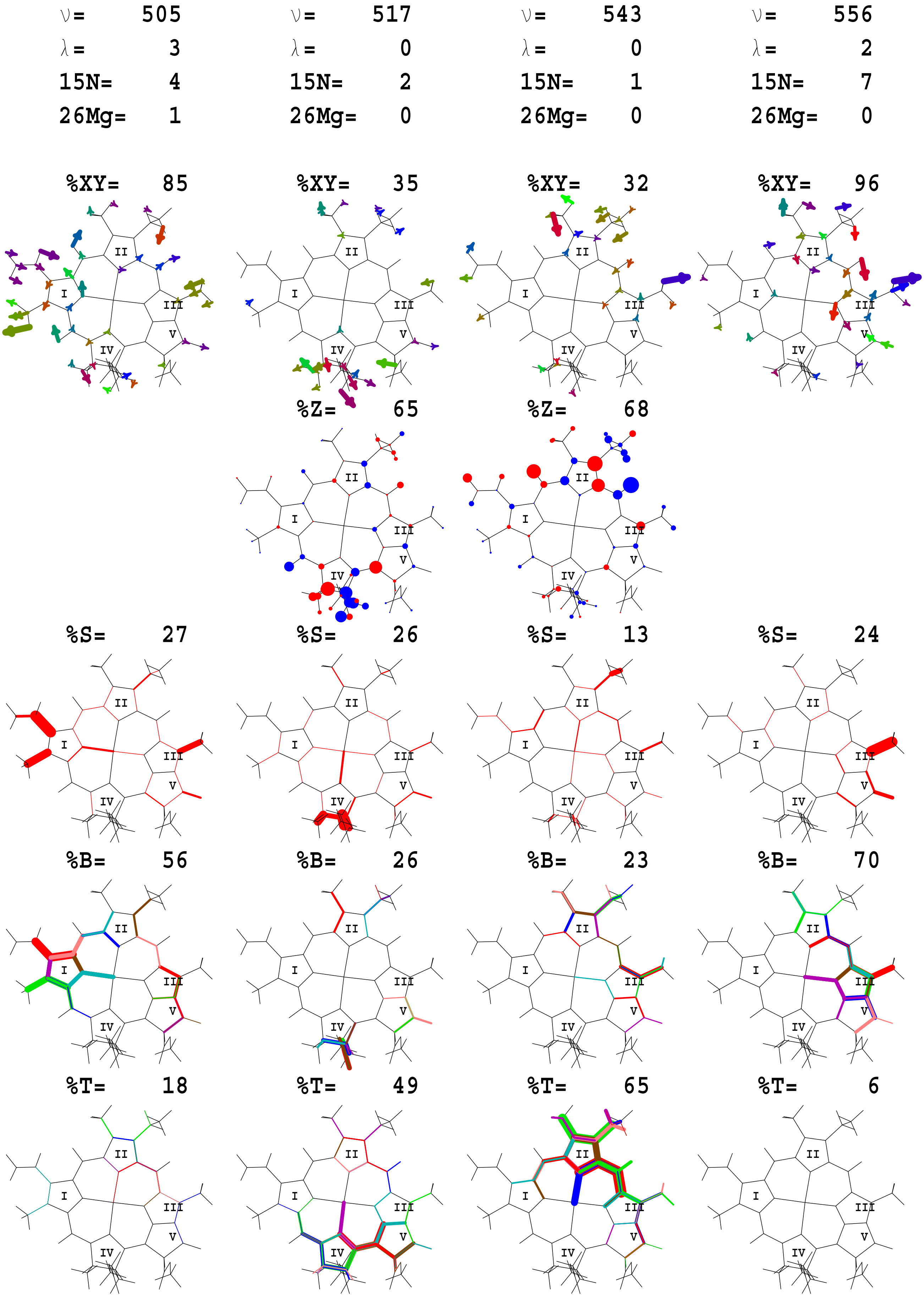

ν= 505
λ= 3
15N= 4
26Mg= 1
%XY= 85
%S= 27
%B= 56
%T= 18
ν= 517
λ= 0
15N= 2
26Mg= 0
%XY= 35
%Z= 65
%S= 26
%B= 26
%T= 49
ν= 543
λ= 0
15N= 1
26Mg= 0
%XY= 32
%Z= 68
%S= 13
%B= 23
%T= 65
ν= 556
λ= 2
15N= 7
26Mg= 0
%XY= 96
%S= 24
%B= 70
%T= 6
I
II
III
IV
V

ν=	569	584	589	623
λ=	0	0	2	1
15N=	1	0	0	1
26Mg=	0	0	0	0

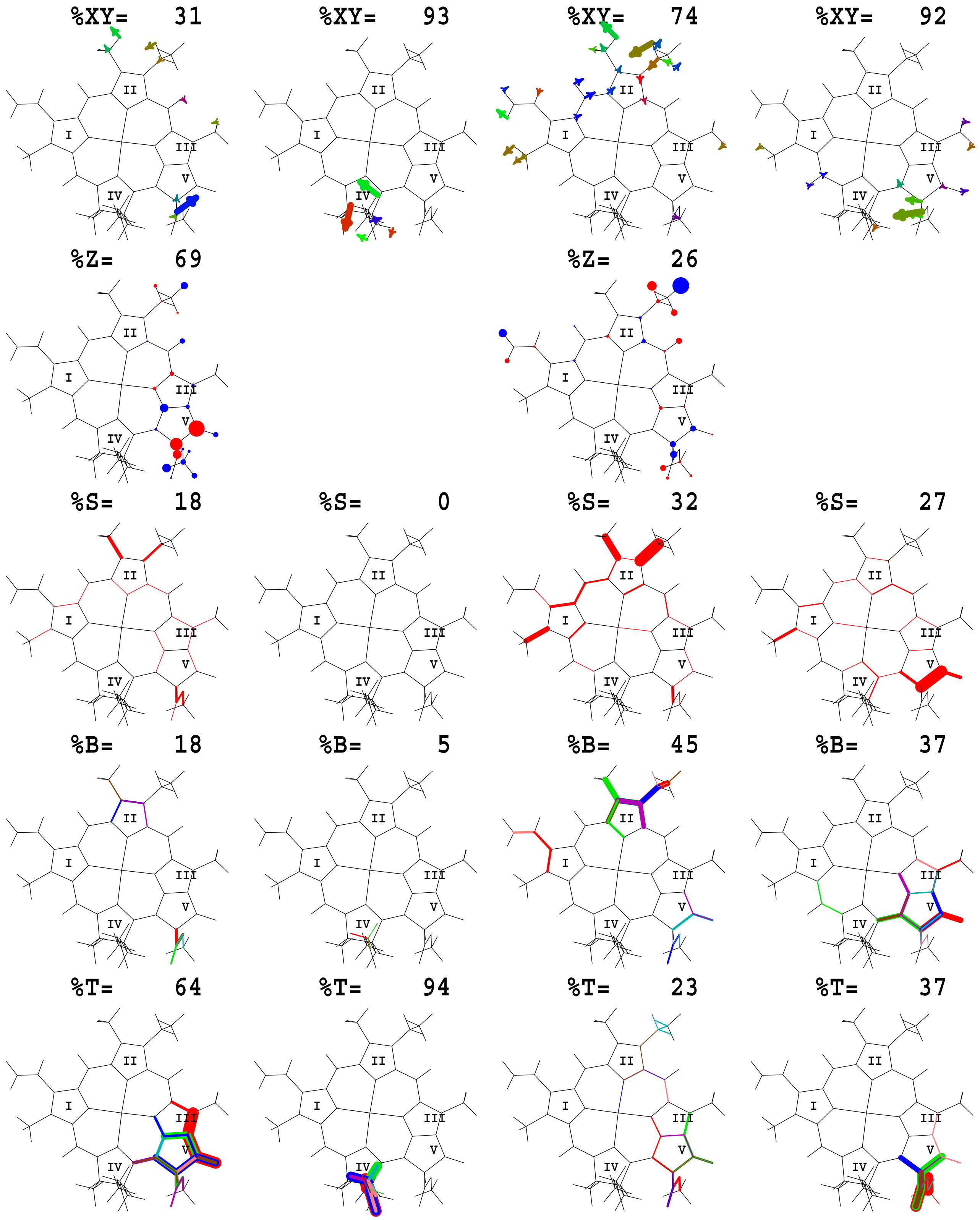

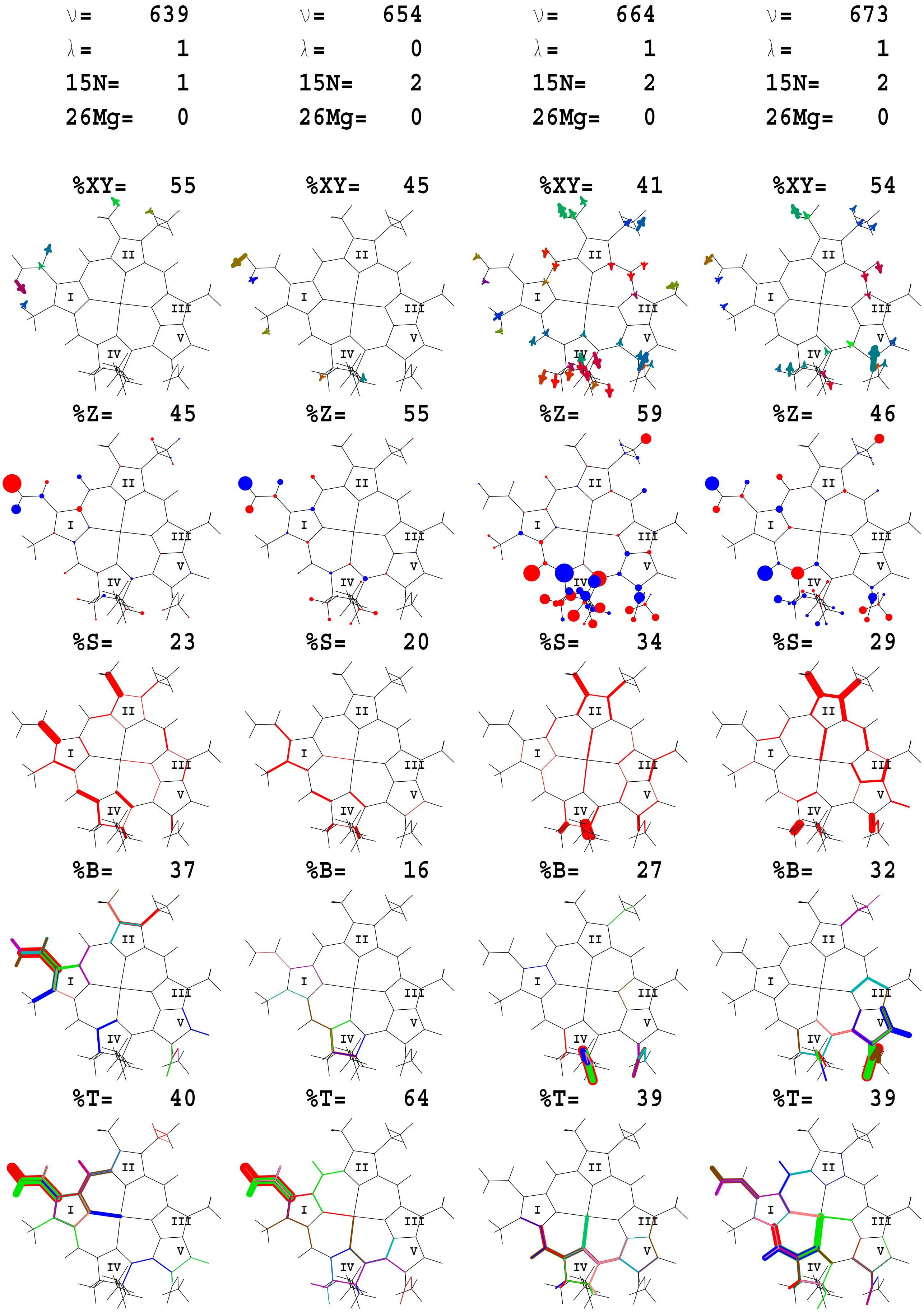

ν= 639
λ= 1
15N= 1
26Mg= 0
%XY= 55
%Z= 45
%S= 23
%B= 37
%T= 40
ν= 654
λ= 0
15N= 2
26Mg= 0
%XY= 45
%Z= 55
%S= 20
%B= 16
%T= 64
ν= 664
λ= 1
15N= 2
26Mg= 0
%XY= 41
%Z= 59
%S= 34
%B= 27
%T= 39
ν= 673
λ= 1
15N= 2
26Mg= 0
%XY= 54
%Z= 46
%S= 29
%B= 32
%T= 39
I
II
III
IV
V

ν= 681	ν= 691	ν= 703	ν= 709
λ= 0	λ= 0	λ= 2	λ= 0
15N= 3	15N= 4	15N= 3	15N= 4
26Mg= 0	26Mg= 0	26Mg= 0	26Mg= 0

%XY= 69	%XY= 76	%XY= 74	%XY= 71

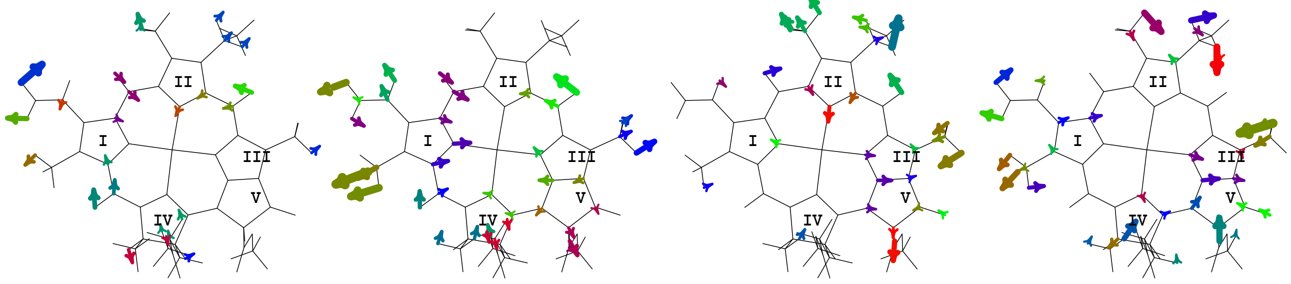

%Z= 31	%Z= 24	%Z= 26	%Z= 29

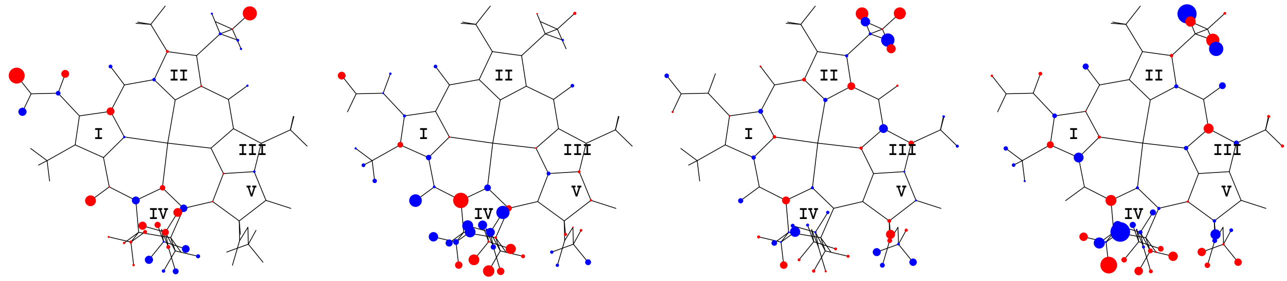

%S= 34	%S= 50	%S= 39	%S= 36

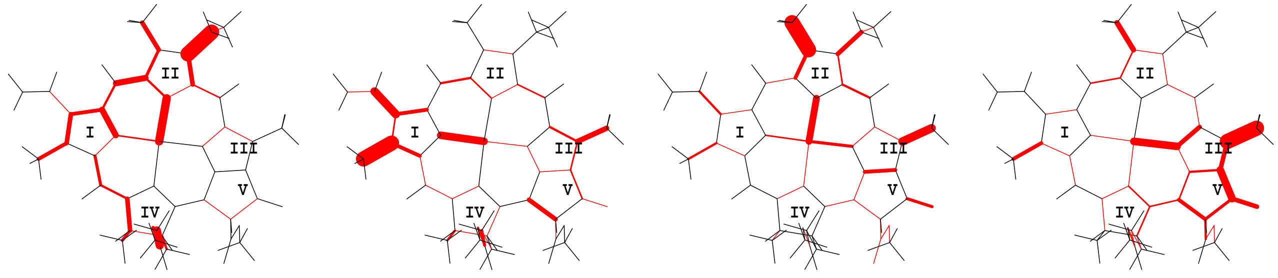

%B= 41	%B= 34	%B= 37	%B= 36

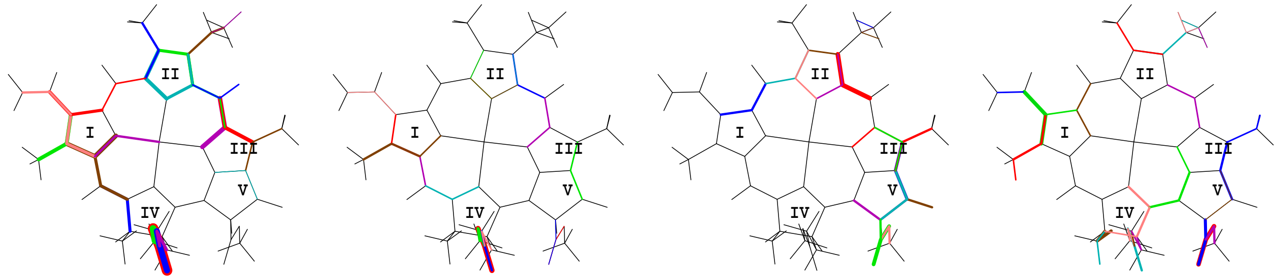

%T= 25	%T= 16	%T= 24	%T= 27

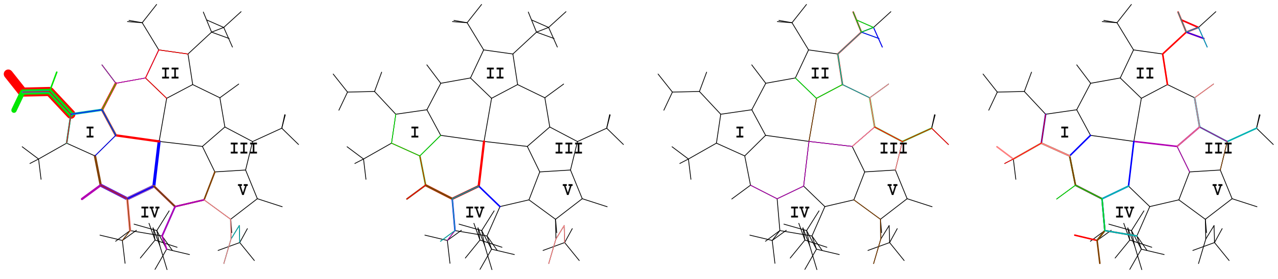

ν=	712	719	723	731
λ=	0	2	0	7
15N=	5	5	4	3
26Mg=	0	0	0	0

%XY= 17 | %XY= 56 | %XY= 18 | %XY= 47

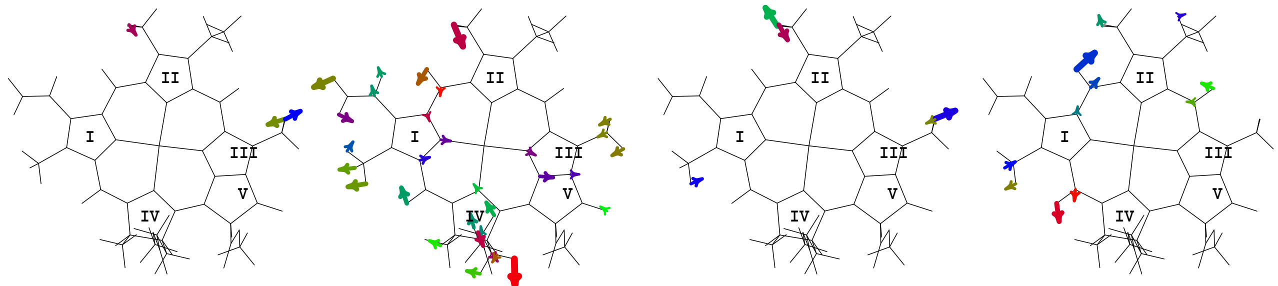

%Z= 83 | %Z= 44 | %Z= 82 | %Z= 53

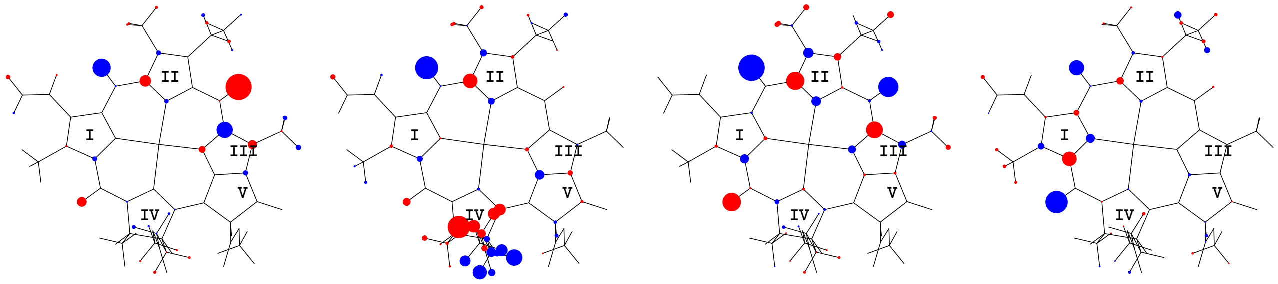

%S= 5 | %S= 31 | %S= 10 | %S= 16

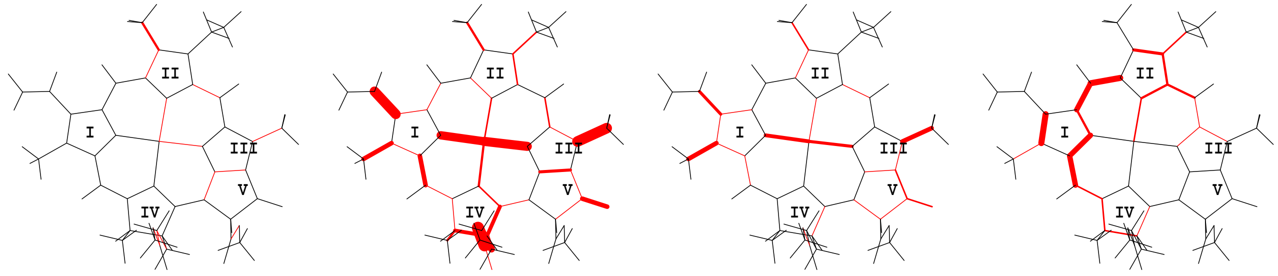

%B= 12 | %B= 37 | %B= 9 | %B= 30

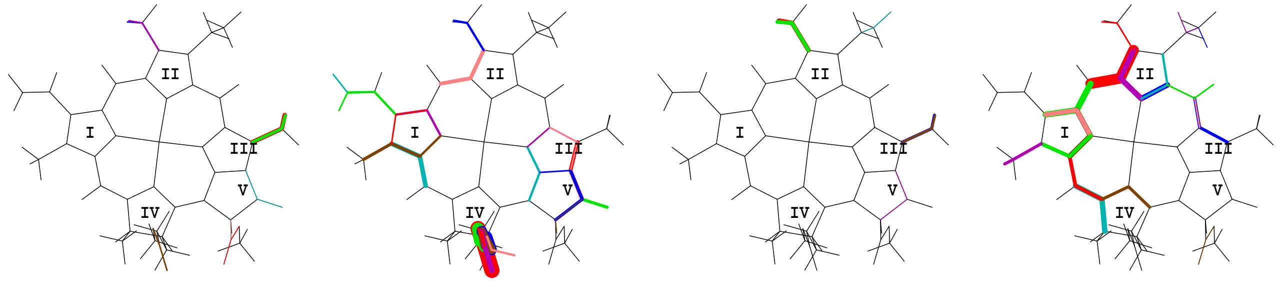

%T= 83 | %T= 32 | %T= 82 | %T= 54

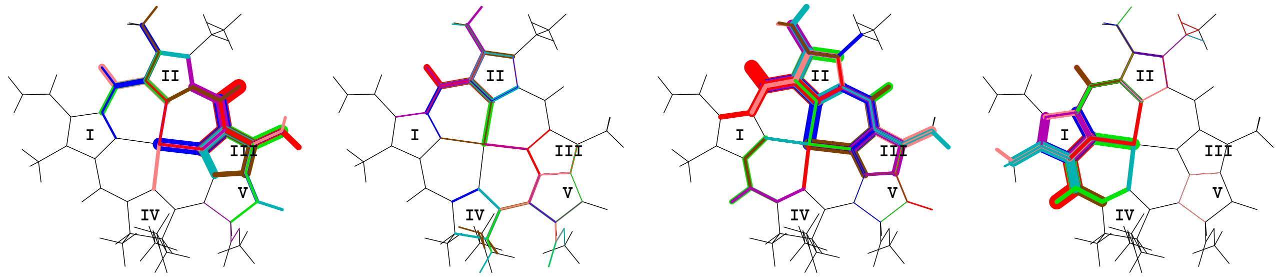

ν= 735	ν= 744	ν= 750	ν= 762
λ= 13	λ= 1	λ= 1	λ= 2
15N= 3	15N= 5	15N= 3	15N= 1
26Mg= 0	26Mg= 1	26Mg= 0	26Mg= 0

%XY= 45		%XY= 60	%XY= 63

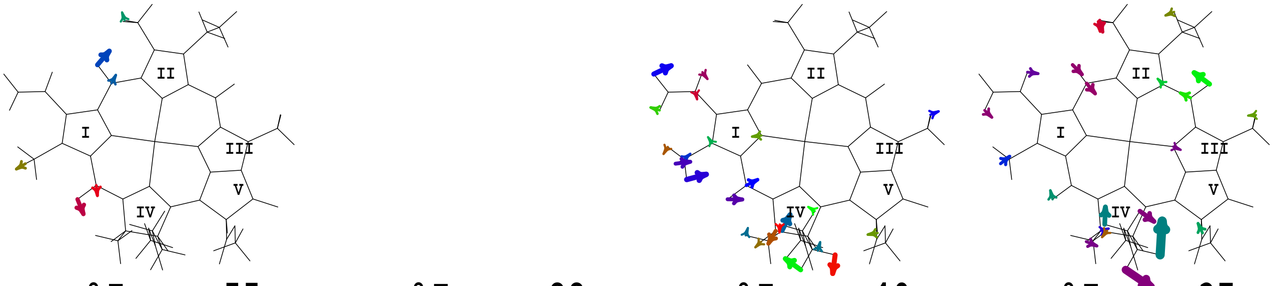

%Z= 55	%Z= 88	%Z= 40	%Z= 37

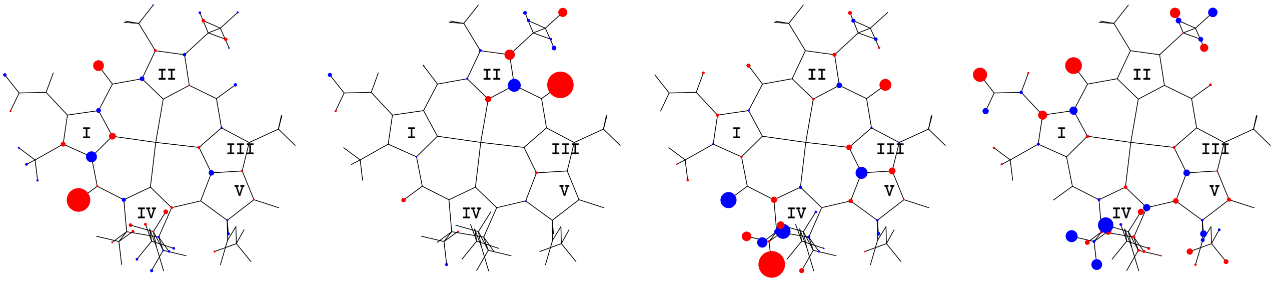

%S= 18	%S= 4	%S= 26	%S= 23

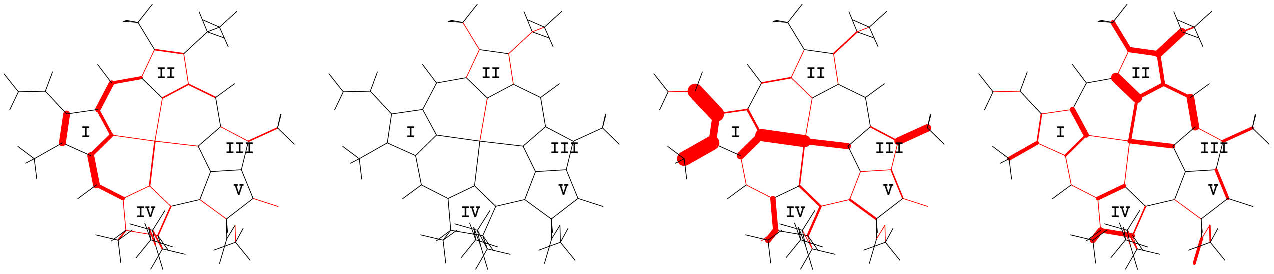

%B= 30	%B= 9	%B= 27	%B= 33

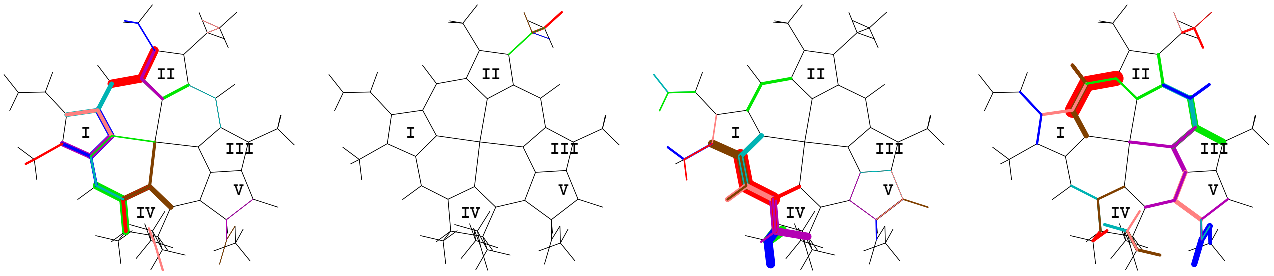

%T= 52	%T= 87	%T= 47	%T= 44

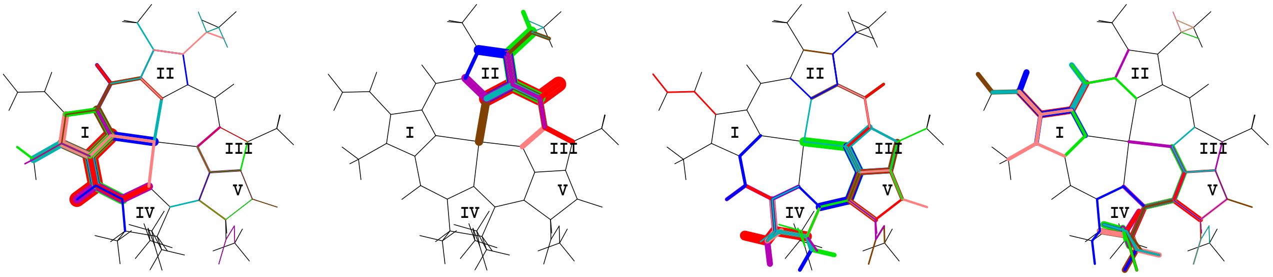

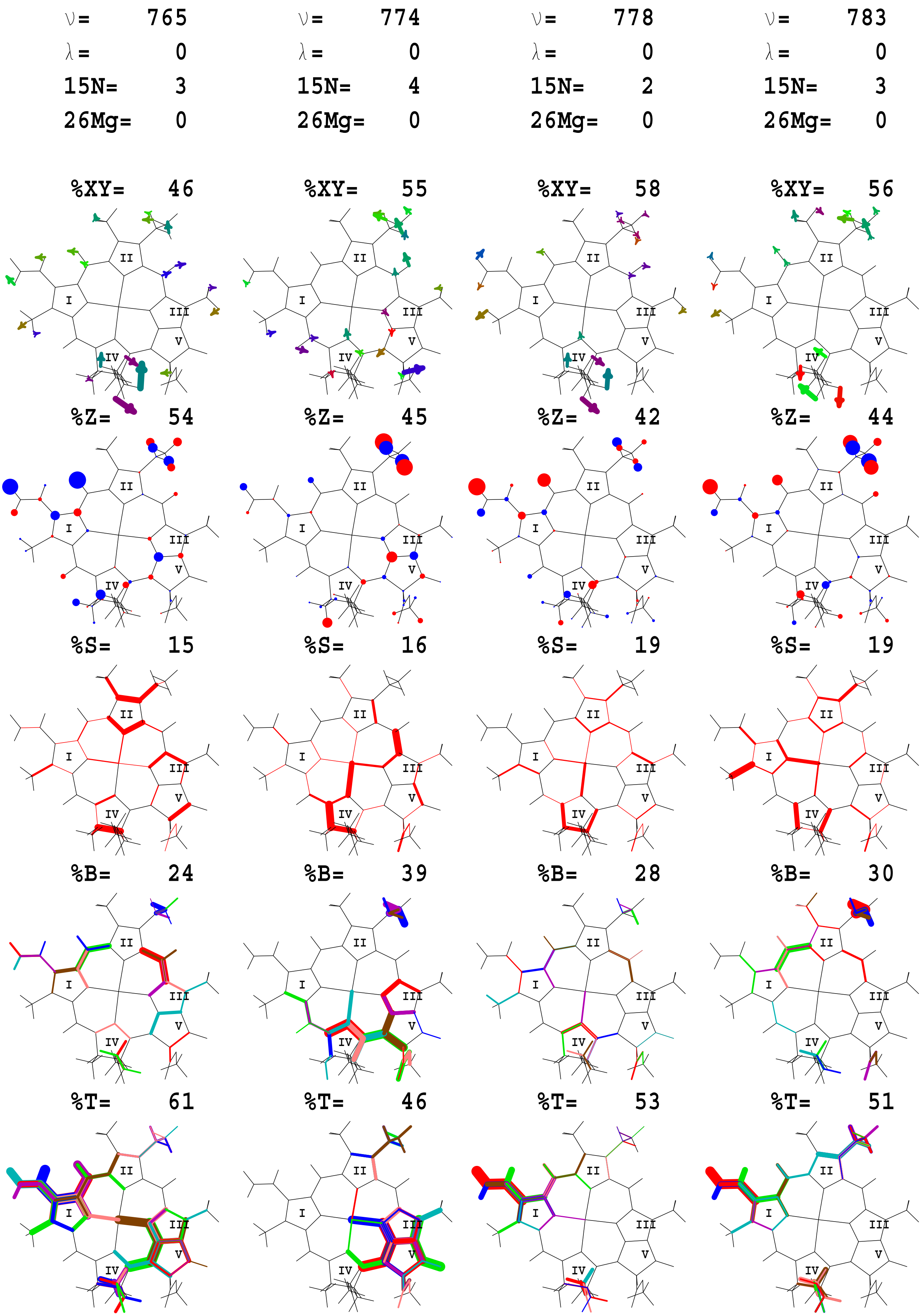

ν= 765
λ= 0
15N= 3
26Mg= 0
%XY= 46
%Z= 54
%S= 15
%B= 24
%T= 61
ν= 774
λ= 0
15N= 4
26Mg= 0
%XY= 55
%Z= 45
%S= 16
%B= 39
%T= 46
ν= 778
λ= 0
15N= 2
26Mg= 0
%XY= 58
%Z= 42
%S= 19
%B= 28
%T= 53
ν= 783
λ= 0
15N= 3
26Mg= 0
%XY= 56
%Z= 44
%S= 19
%B= 30
%T= 51
I
II
III
IV
V

ν=	791	804	827	837
λ=	1	1	0	4
15N=	3	2	0	1
26Mg=	0	0	0	0
%XY=	69	66	52	
%Z=	31	34	48	45
%S=	18	16	17	33
%B=	44	31	30	30
%T=	37	53	53	37

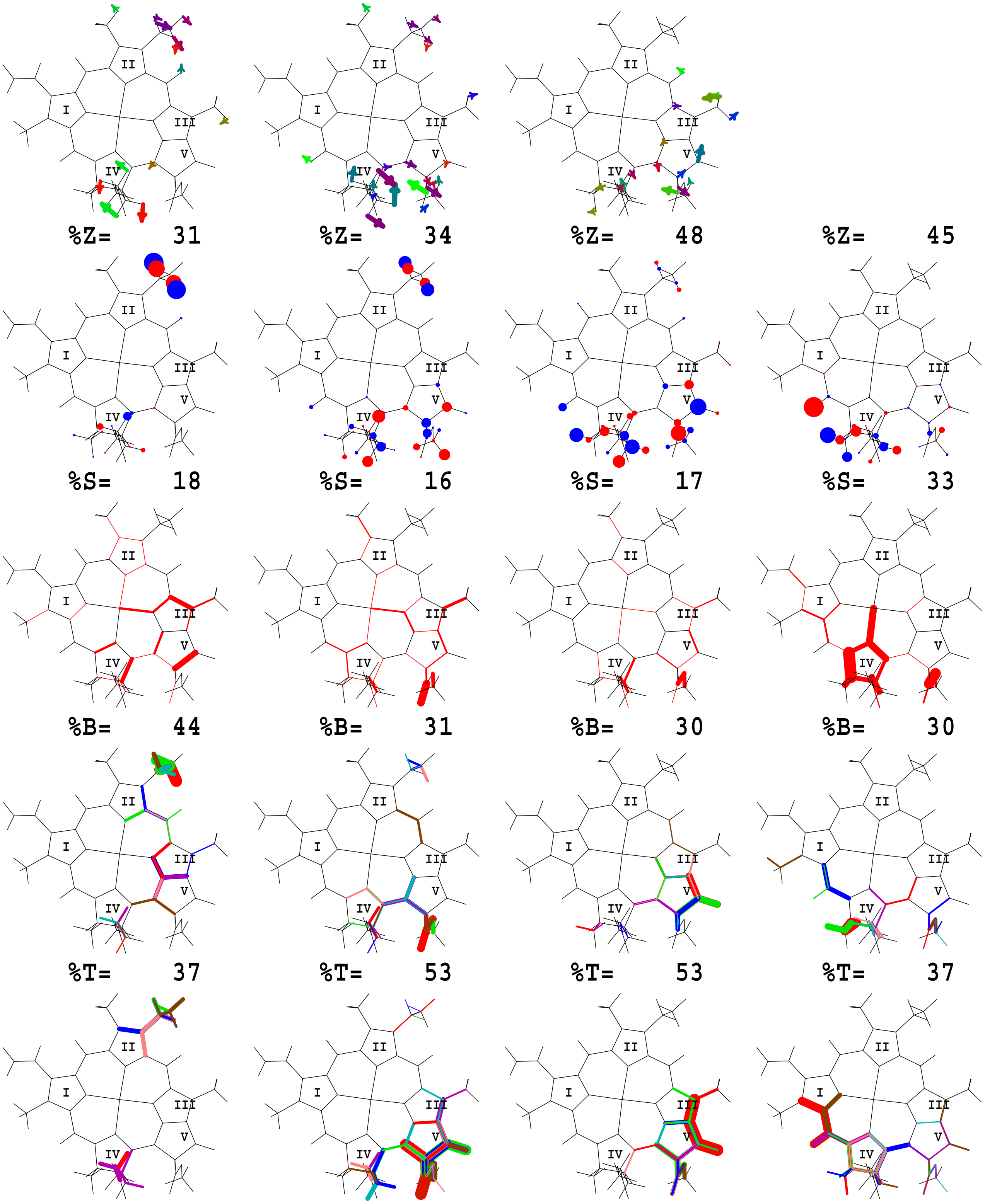

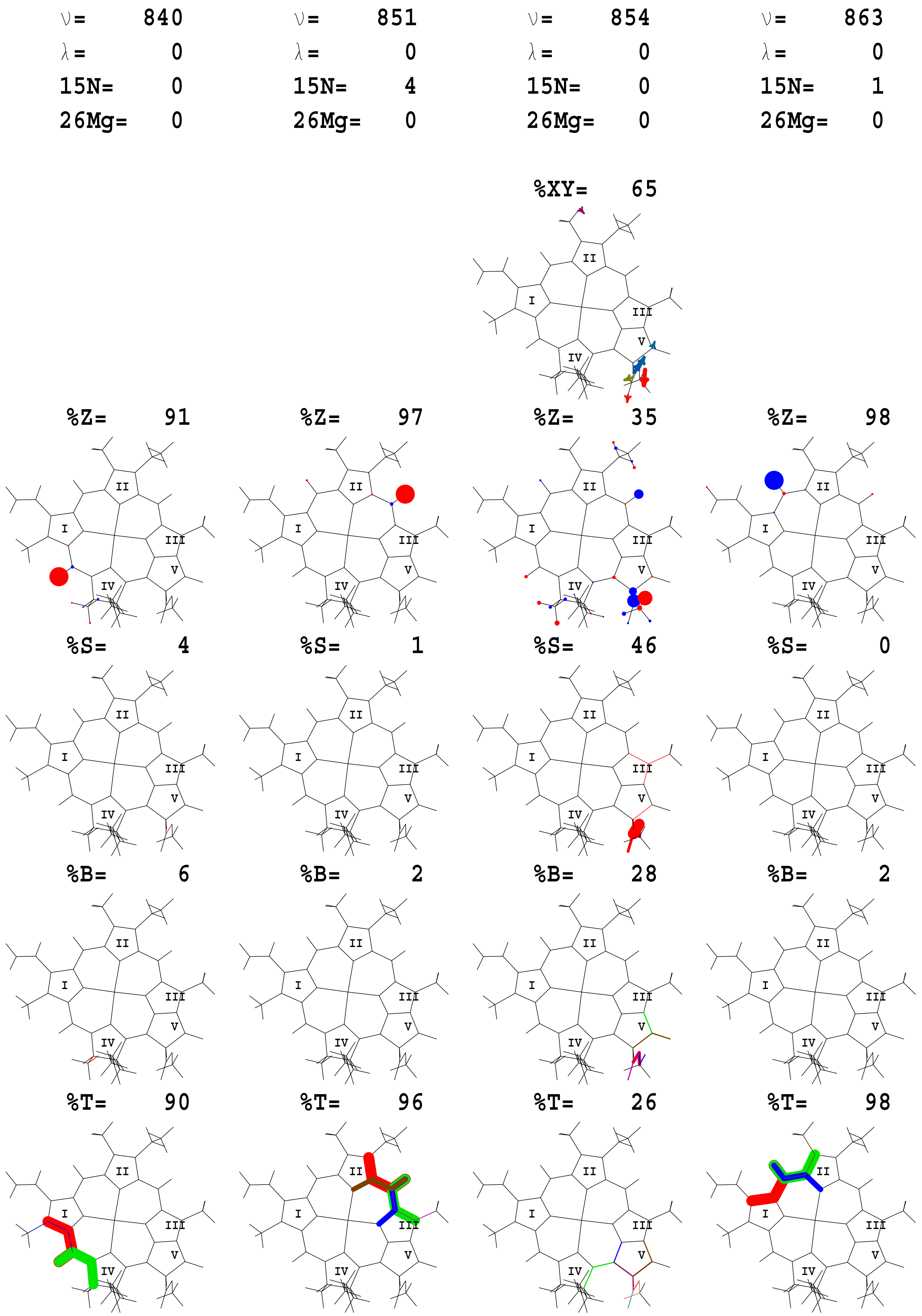

ν= 840
λ= 0
15N= 0
26Mg= 0
ν= 851
λ= 0
15N= 4
26Mg= 0
ν= 854
λ= 0
15N= 0
26Mg= 0
ν= 863
λ= 0
15N= 1
26Mg= 0
%XY= 65
%Z= 91
%Z= 97
%Z= 35
%Z= 98
%S= 4
%S= 1
%S= 46
%S= 0
%B= 6
%B= 2
%B= 28
%B= 2
%T= 90
%T= 96
%T= 26
%T= 98

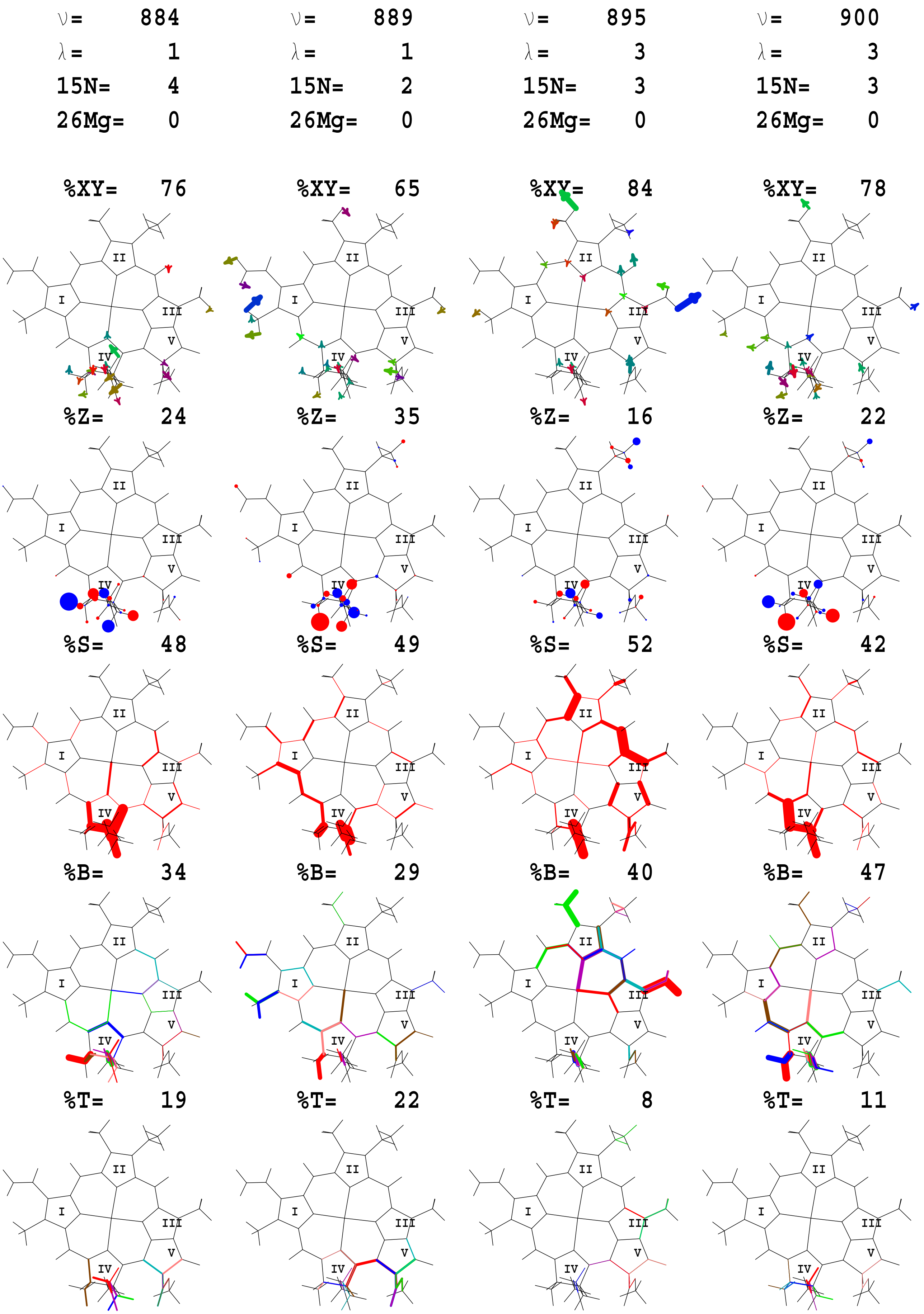
ν= 884
λ= 1
15N= 4
26Mg= 0
%XY= 76
%Z= 24
%S= 48
%B= 34
%T= 19
ν= 889
λ= 1
15N= 2
26Mg= 0
%XY= 65
%Z= 35
%S= 49
%B= 29
%T= 22
ν= 895
λ= 3
15N= 3
26Mg= 0
%XY= 84
%Z= 16
%S= 52
%B= 40
%T= 8
ν= 900
λ= 3
15N= 3
26Mg= 0
%XY= 78
%Z= 22
%S= 42
%B= 47
%T= 11
I
II
III
IV
V

ν= 917	ν= 931	ν= 944	ν= 958
λ= 7	λ= 2	λ= 0	λ= 1
15N= 0	15N= 6	15N= 6	15N= 5
26Mg= 0	26Mg= 0	26Mg= 0	26Mg= 0

%XY= 85	%XY= 81	%XY= 27	%XY= 66

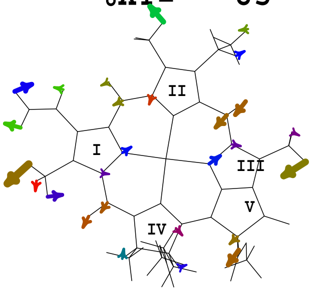

%Z= 15	%Z= 19	%Z= 73	%Z= 34

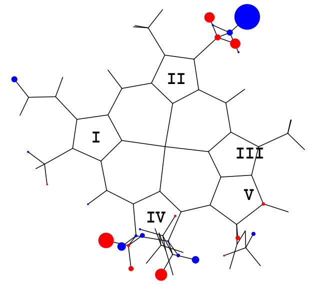

%S= 41	%S= 37	%S= 0	%S= 49

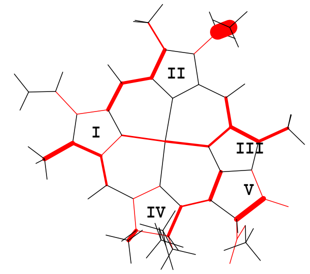

%B= 50	%B= 57	%B= 0	%B= 43

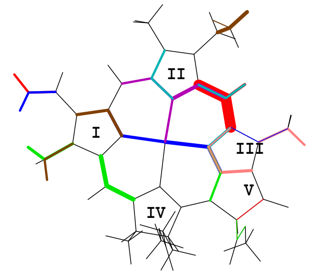

%T= 8	%T= 6	%T= 99	%T= 9

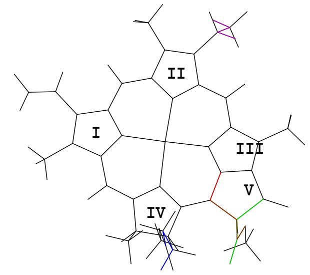

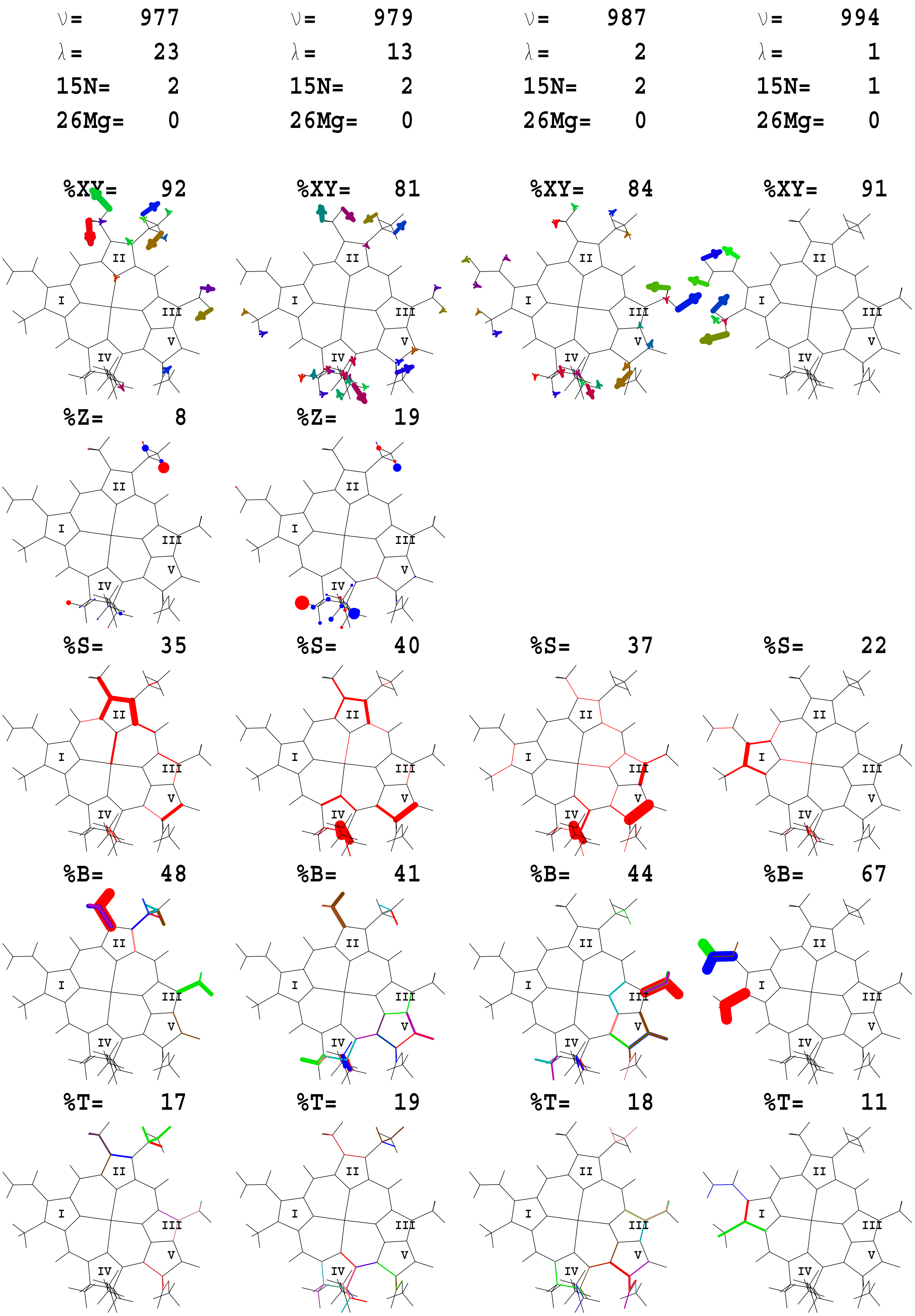

ν= 977
λ= 23
15N= 2
26Mg= 0
%XY= 92
%Z= 8
%S= 35
%B= 48
%T= 17
ν= 979
λ= 13
15N= 2
26Mg= 0
%XY= 81
%Z= 19
%S= 40
%B= 41
%T= 19
ν= 987
λ= 2
15N= 2
26Mg= 0
%XY= 84
%S= 37
%B= 44
%T= 18
ν= 994
λ= 1
15N= 1
26Mg= 0
%XY= 91
%S= 22
%B= 67
%T= 11
I
II
III
IV
V

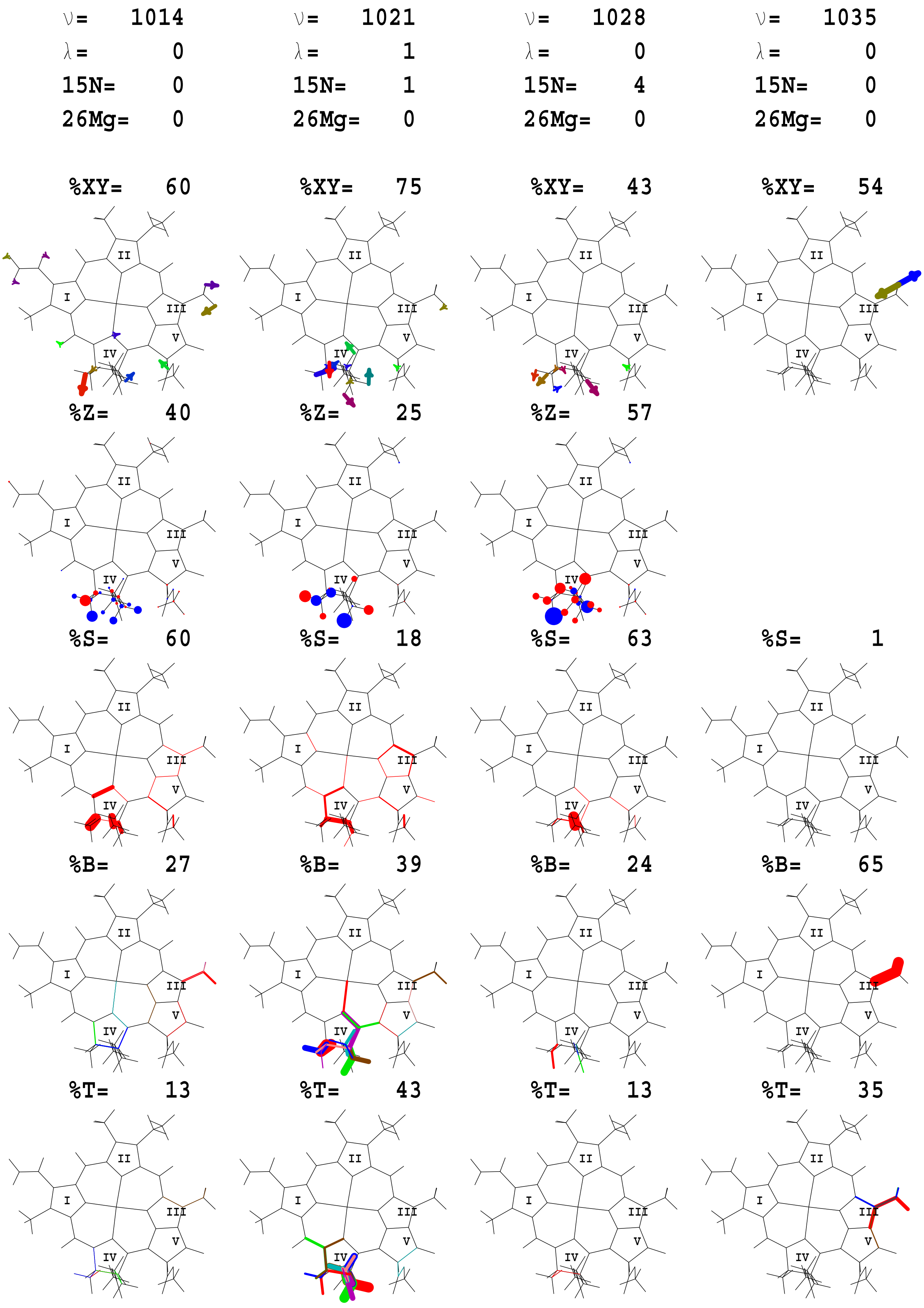

ν= 1014
λ= 0
15N= 0
26Mg= 0
%XY= 60
%Z= 40
%S= 60
%B= 27
%T= 13
ν= 1021
λ= 1
15N= 1
26Mg= 0
%XY= 75
%Z= 25
%S= 18
%B= 39
%T= 43
ν= 1028
λ= 0
15N= 4
26Mg= 0
%XY= 43
%Z= 57
%S= 63
%B= 24
%T= 13
ν= 1035
λ= 0
15N= 0
26Mg= 0
%XY= 54
%S= 1
%B= 65
%T= 35
I
II
III
IV
V

ν=	1036	ν=	1037	ν=	1038	ν=	1039
λ=	0	λ=	0	λ=	0	λ=	0
15N=	0	15N=	0	15N=	2	15N=	4
26Mg=	0	26Mg=	0	26Mg=	0	26Mg=	0

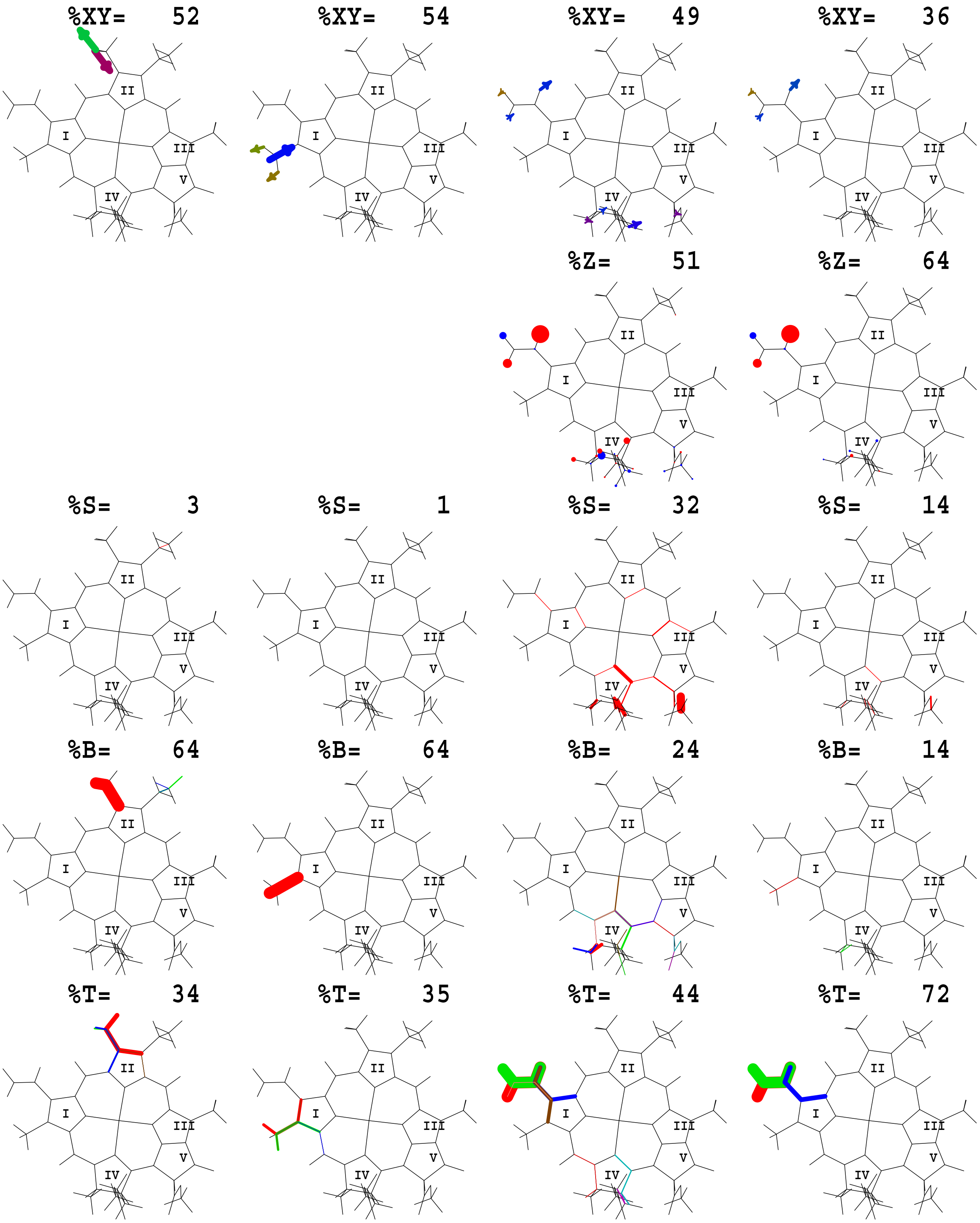

ν=	1049	1058	1064	1070
λ=	0	1	0	0
15N=	1	1	2	5
26Mg=	0	0	0	0

%XY= 43 %XY= 61 %XY= 63 %XY= 51

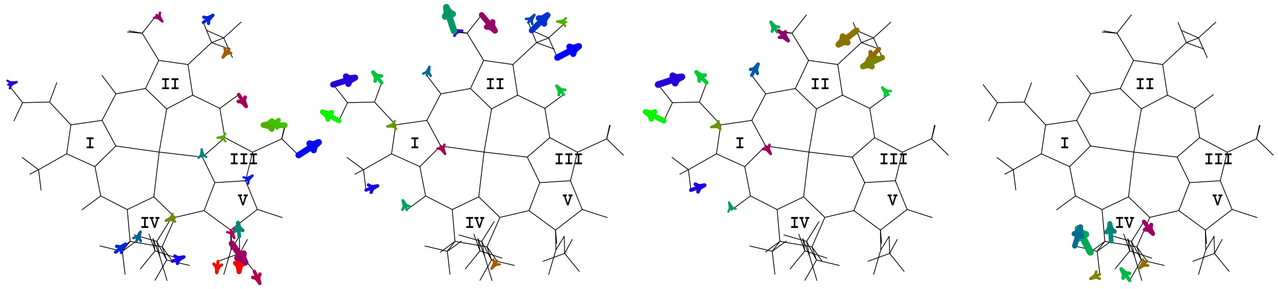

%Z= 57 %Z= 39 %Z= 37 %Z= 49

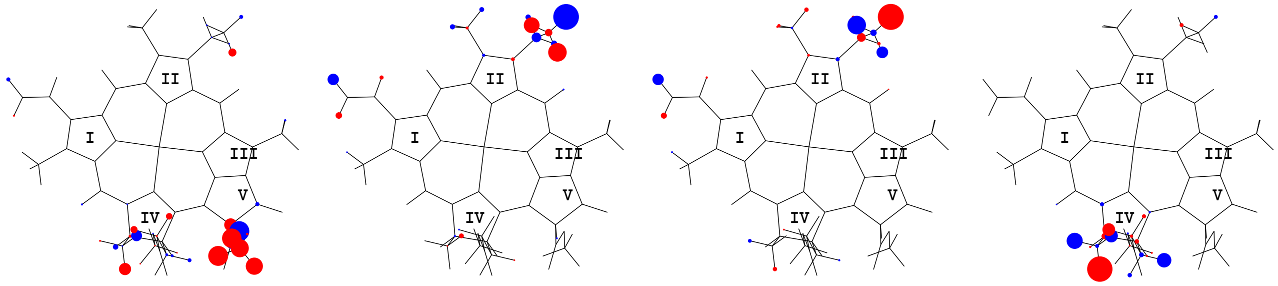

%S= 73 %S= 30 %S= 28 %S= 25

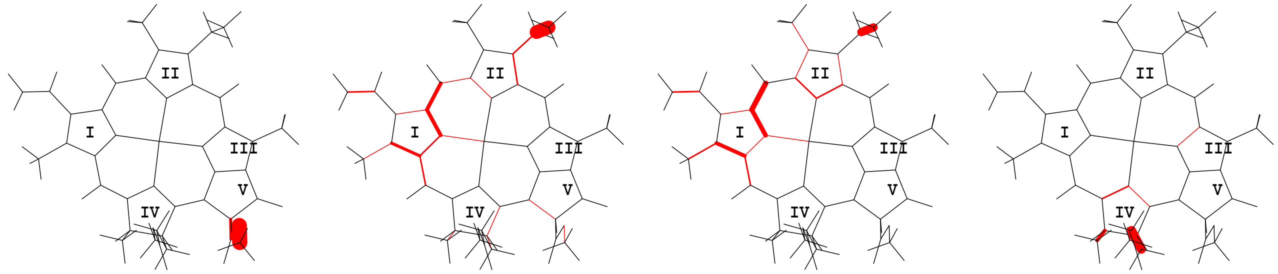

%B= 19 %B= 54 %B= 56 %B= 45

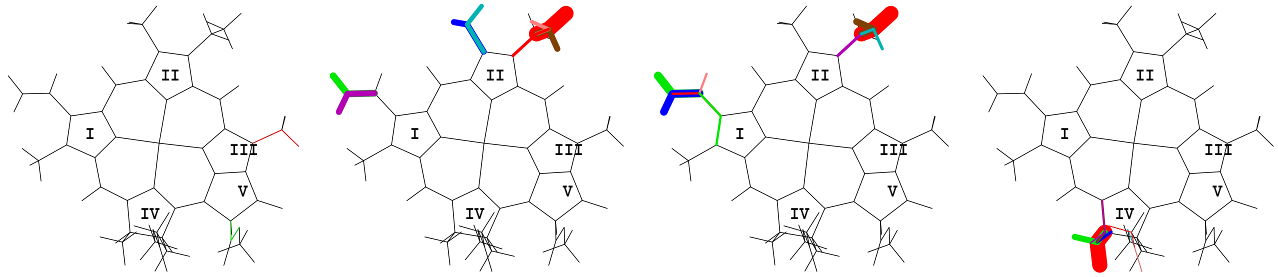

%T= 9 %T= 17 %T= 16 %T= 31

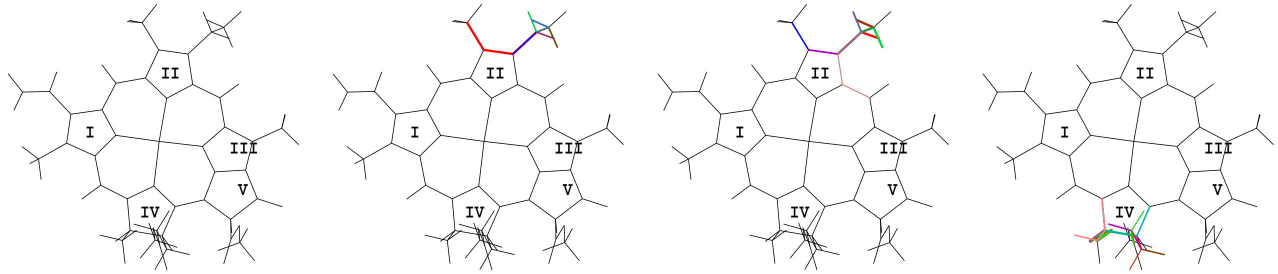

ν= 1084	ν= 1092	ν= 1105	ν= 1110
λ= 2	λ= 5	λ= 1	λ= 2
15N= 2	15N= 4	15N= 2	15N= 5
26Mg= 0	26Mg= 0	26Mg= 0	26Mg= 0

%XY= 56 %XY= 78 %XY= 92 %XY= 69

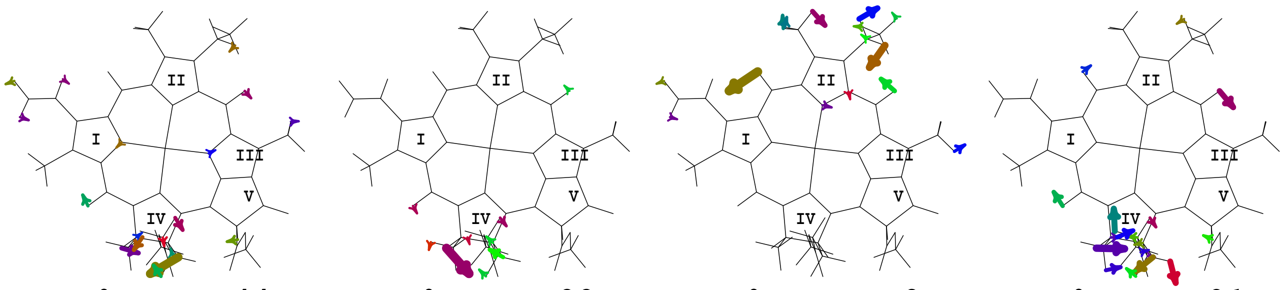

%Z= 44 %Z= 22 %Z= 8 %Z= 31

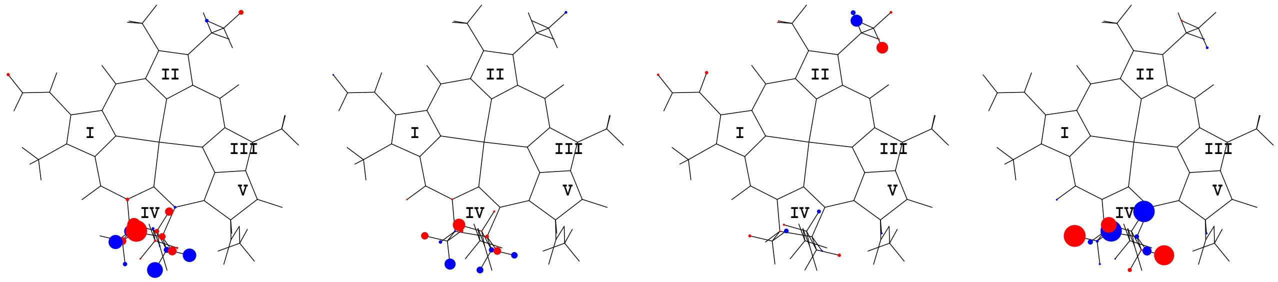

%S= 49 %S= 42 %S= 33 %S= 18

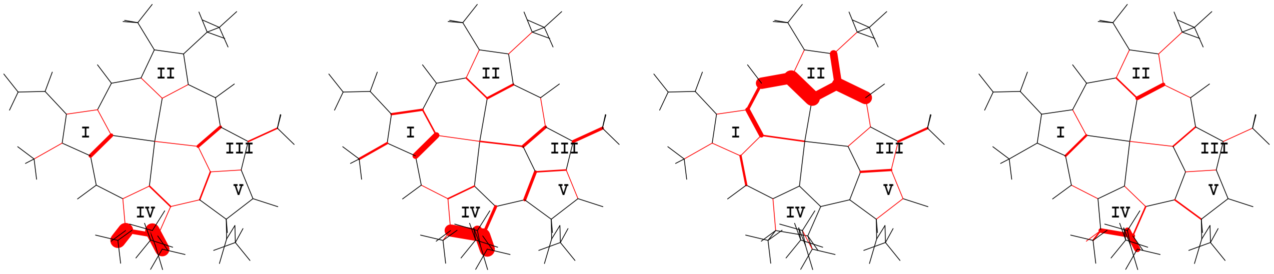

%B= 30 %B= 35 %B= 48 %B= 41

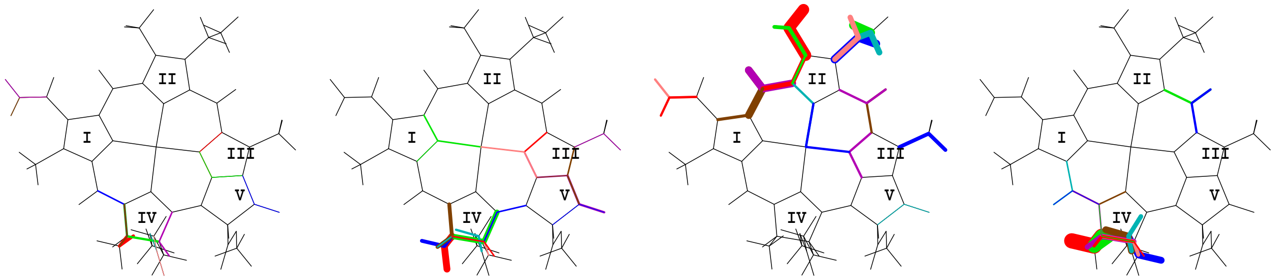

%T= 21 %T= 23 %T= 19 %T= 41

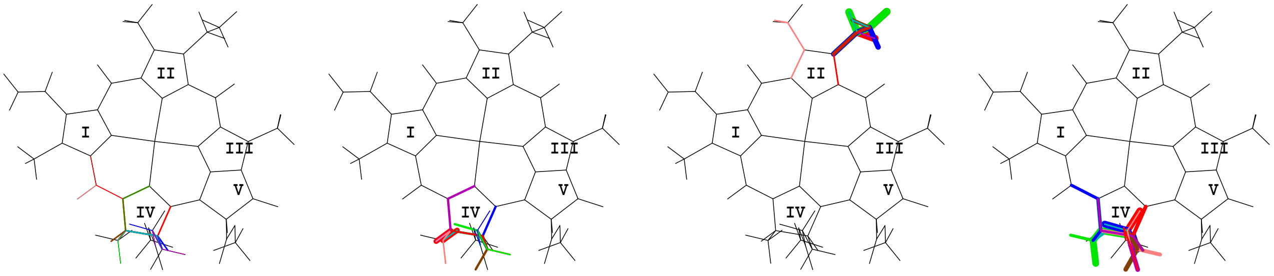

ν= 1116	ν= 1117	ν= 1127	ν= 1131
λ= 0	λ= 1	λ= 1	λ= 14
15N= 6	15N= 11	15N= 7	15N= 8
26Mg= 0	26Mg= 0	26Mg= 0	26Mg= 0

%XY= 96	%XY= 99	%XY= 94	%XY= 95

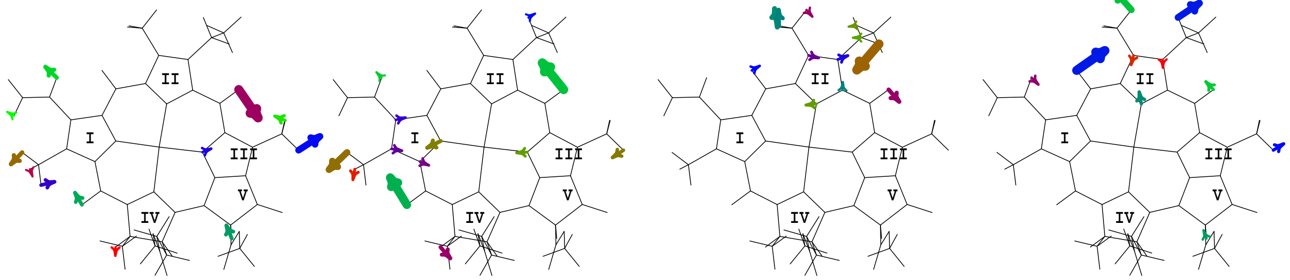

%S= 36	%S= 49	%S= 49	%S= 33

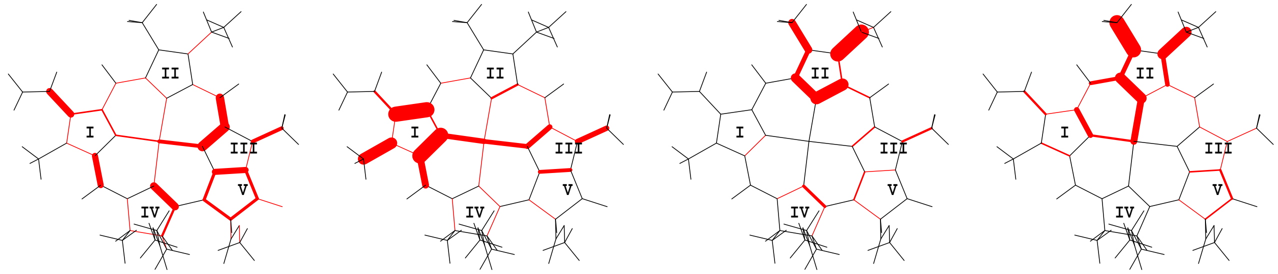

%B= 53	%B= 47	%B= 37	%B= 54

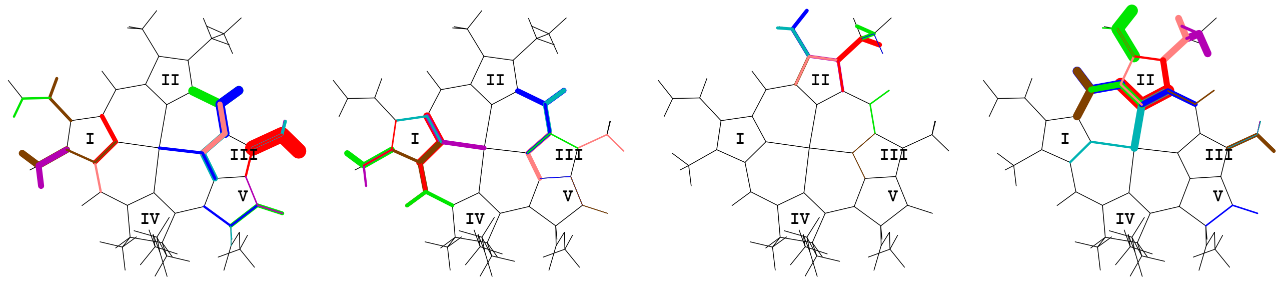

%T= 10	%T= 4	%T= 14	%T= 13

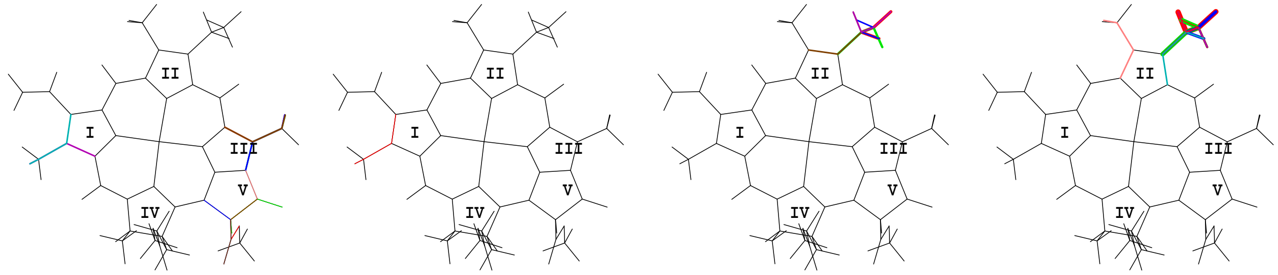

ν=	1142	1145	1146	1159
λ=	1	0	0	14
15N=	4	0	0	6
26Mg=	0	0	0	0
%XY=	92	58	48	97
%Z=		42	52	3
%S=	38	1	1	61
%B=	50	84	84	33
%T=	12	15	15	6

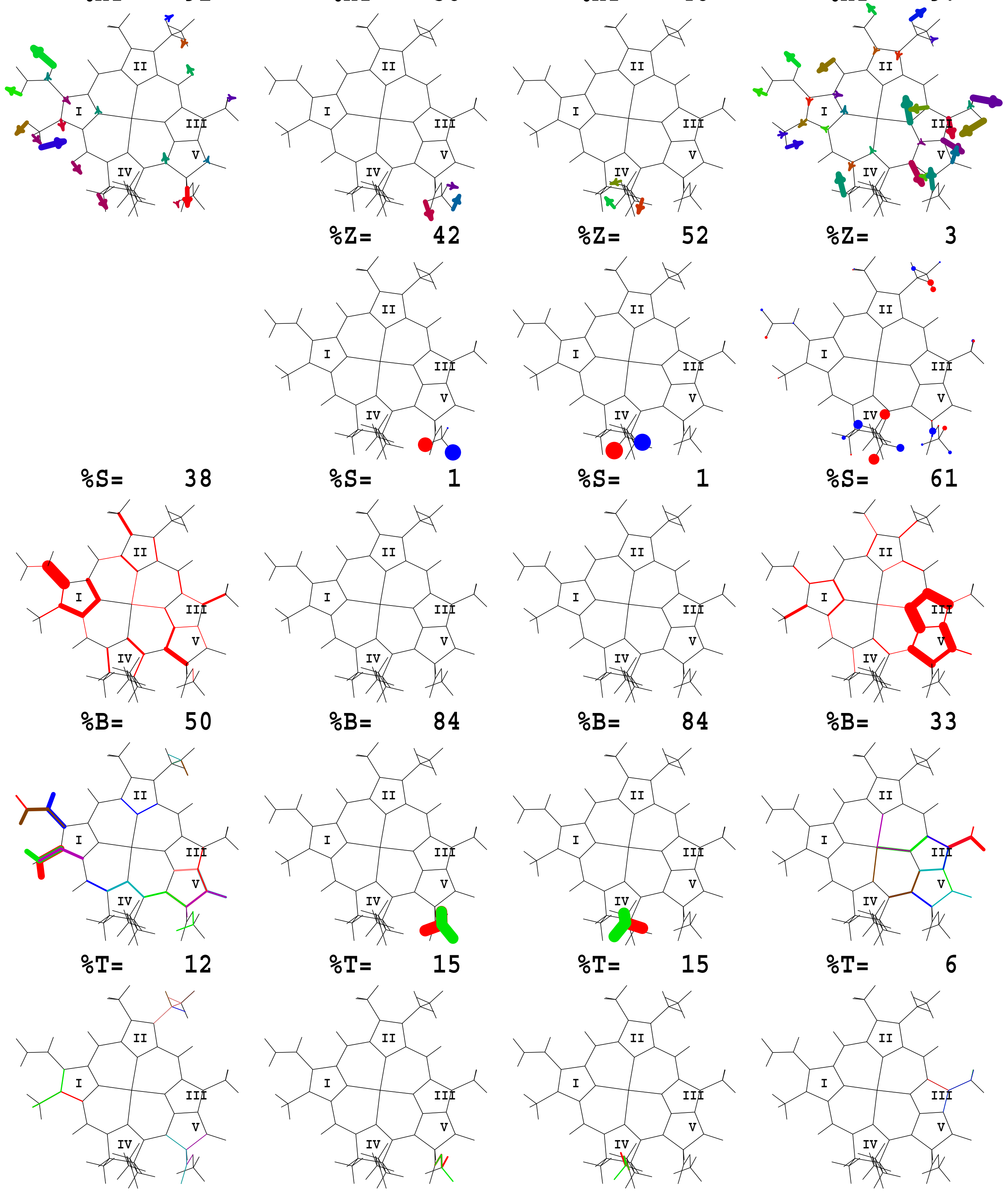

ν=	1173	1178	1179	1183
λ=	0	0	1	0
15N=	1	0	0	4
26Mg=	0	0	0	0

%XY= 37	%XY= 41	%XY= 47	%XY= 87

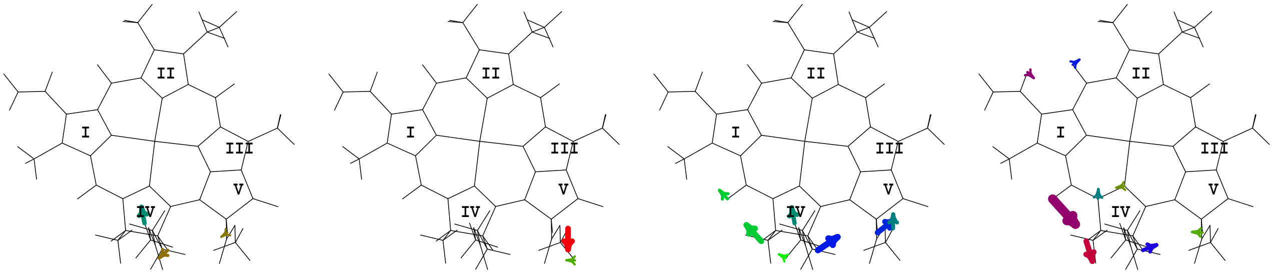

%Z= 63	%Z= 59	%Z= 53	%Z= 13

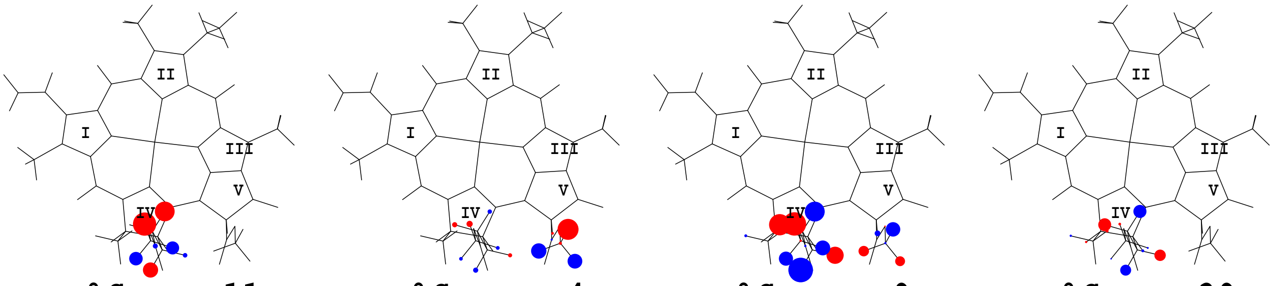

%S= 11	%S= 4	%S= 9	%S= 39

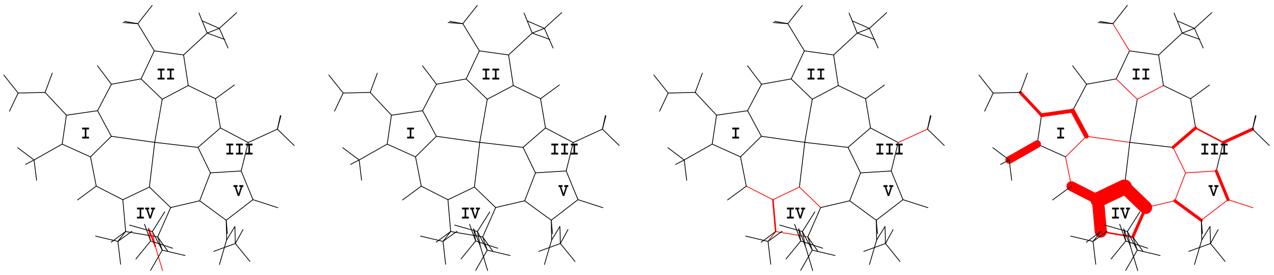

%B= 65	%B= 77	%B= 59	%B= 46

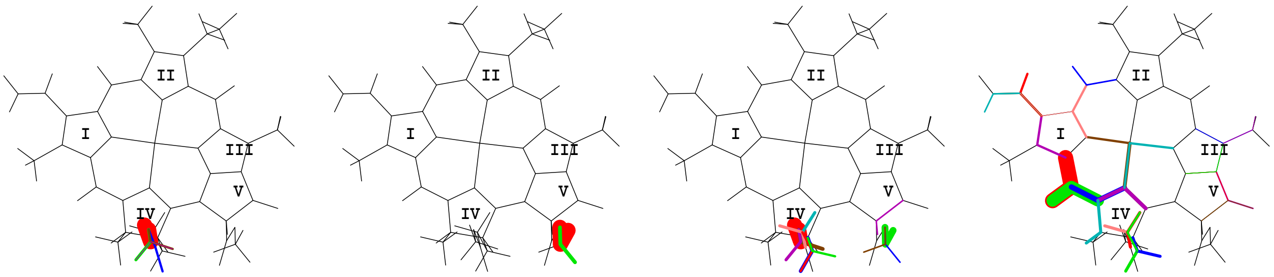

%T= 23	%T= 19	%T= 33	%T= 15

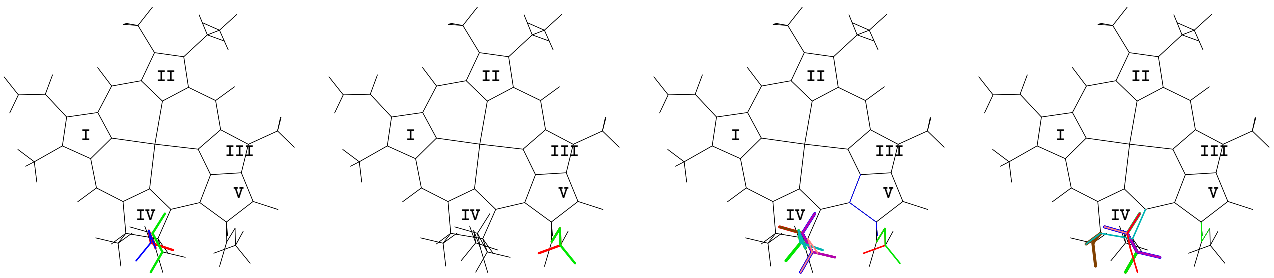

ν= 1193	ν= 1204	ν= 1212	ν= 1224
λ= 9	λ= 0	λ= 5	λ= 47
15N= 1	15N= 1	15N= 0	15N= 1
26Mg= 0	26Mg= 0	26Mg= 0	26Mg= 0

%XY= 93

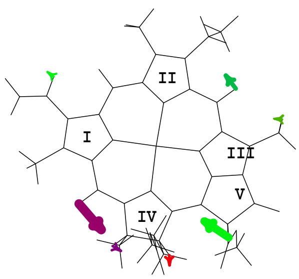

%XY= 93

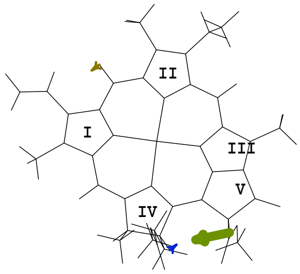

%XY= 94

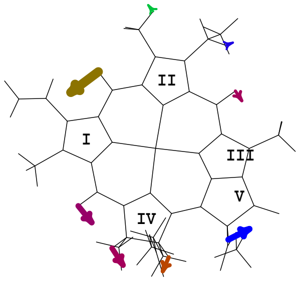

%Z= 87

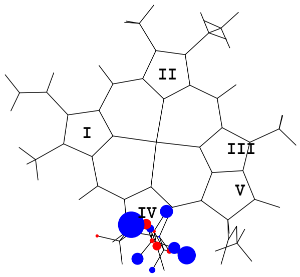

%S= 31

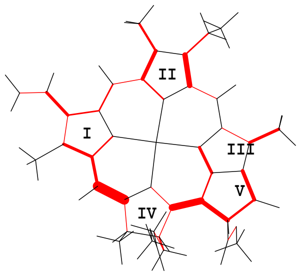

%S= 37

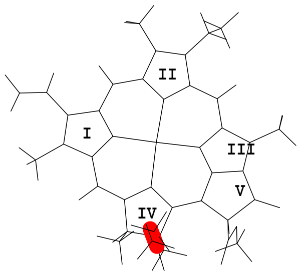

%S= 15

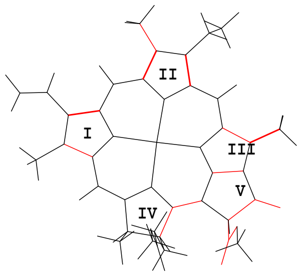

%S= 29

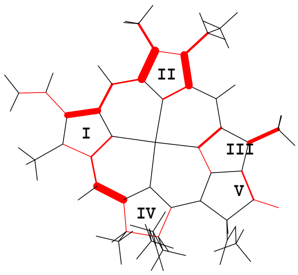

%B= 55

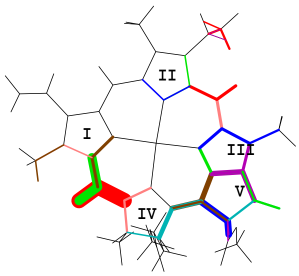

%B= 42

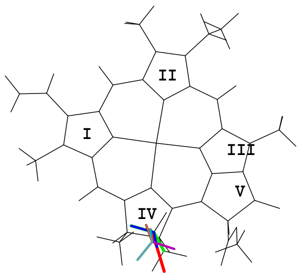

%B= 35

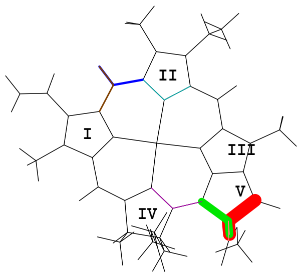

%B= 53

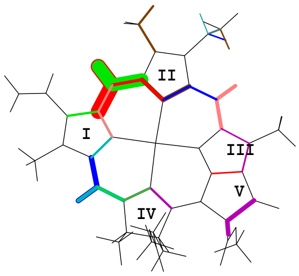

%T= 15

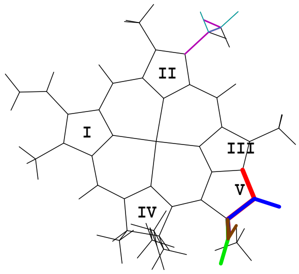

%T= 21

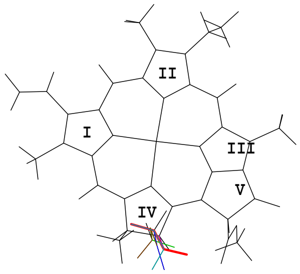

%T= 50

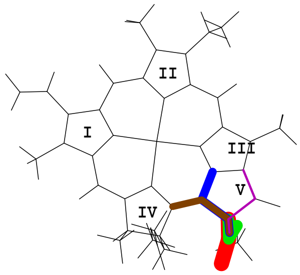

%T= 18

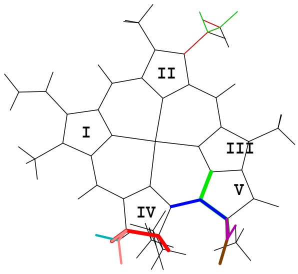

ν= 1248	ν= 1258	ν= 1260	ν= 1271
λ= 1	λ= 1	λ= 15	λ= 0
15N= 2	15N= 1	15N= 0	15N= 0
26Mg= 0	26Mg= 0	26Mg= 0	26Mg= 0

%XY= 74 %XY= 48 %XY= 65 %XY= 39

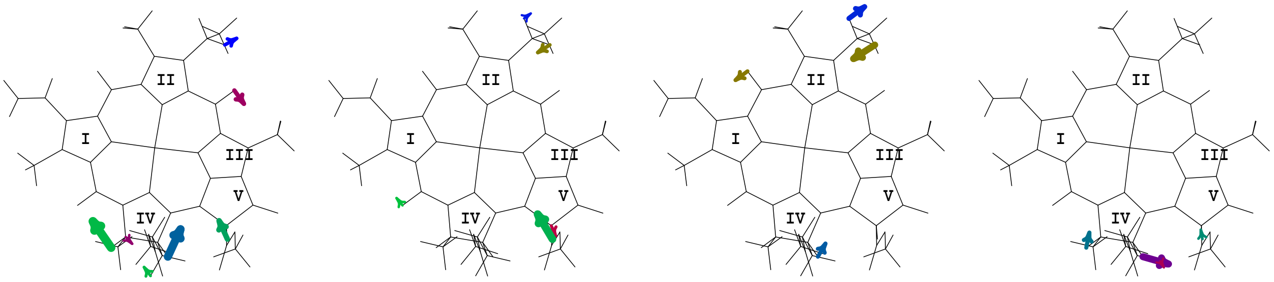

%Z= 26 %Z= 52 %Z= 35 %Z= 61

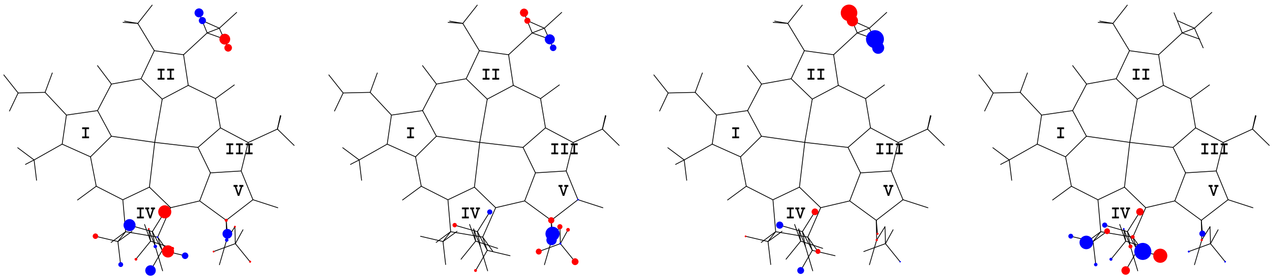

%S= 22 %S= 36 %S= 15 %S= 12

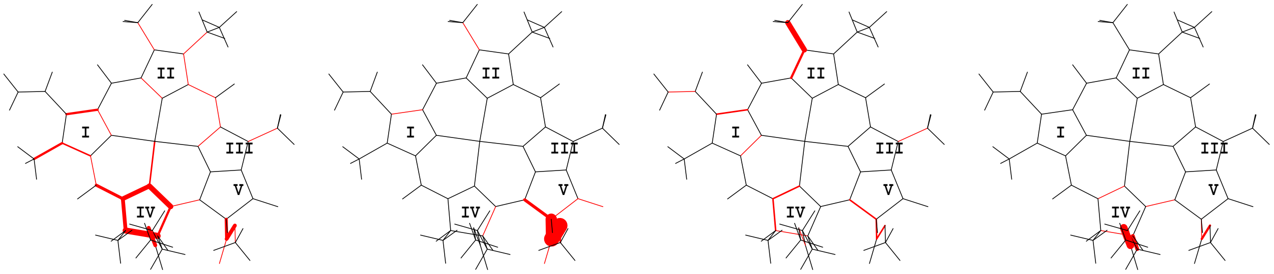

%B= 39 %B= 38 %B= 59 %B= 40

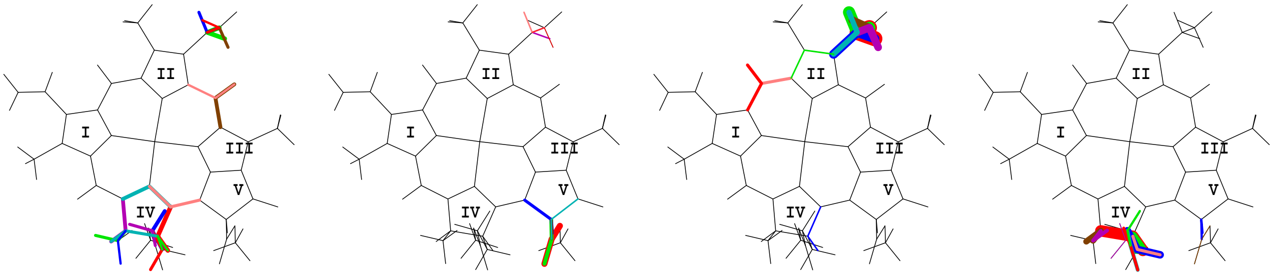

%T= 39 %T= 26 %T= 26 %T= 48

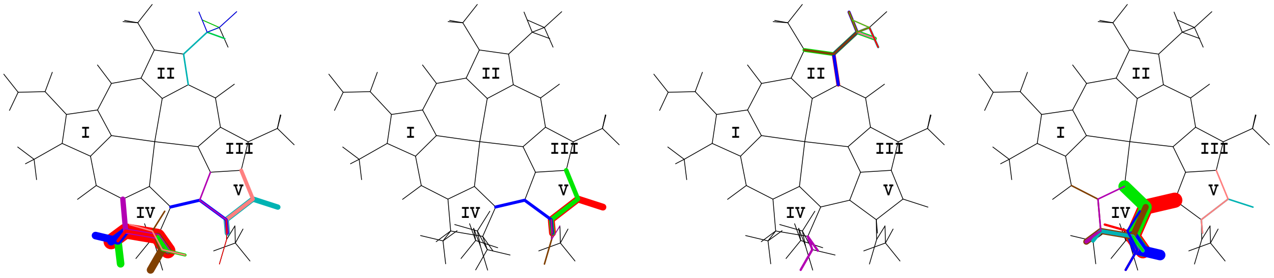

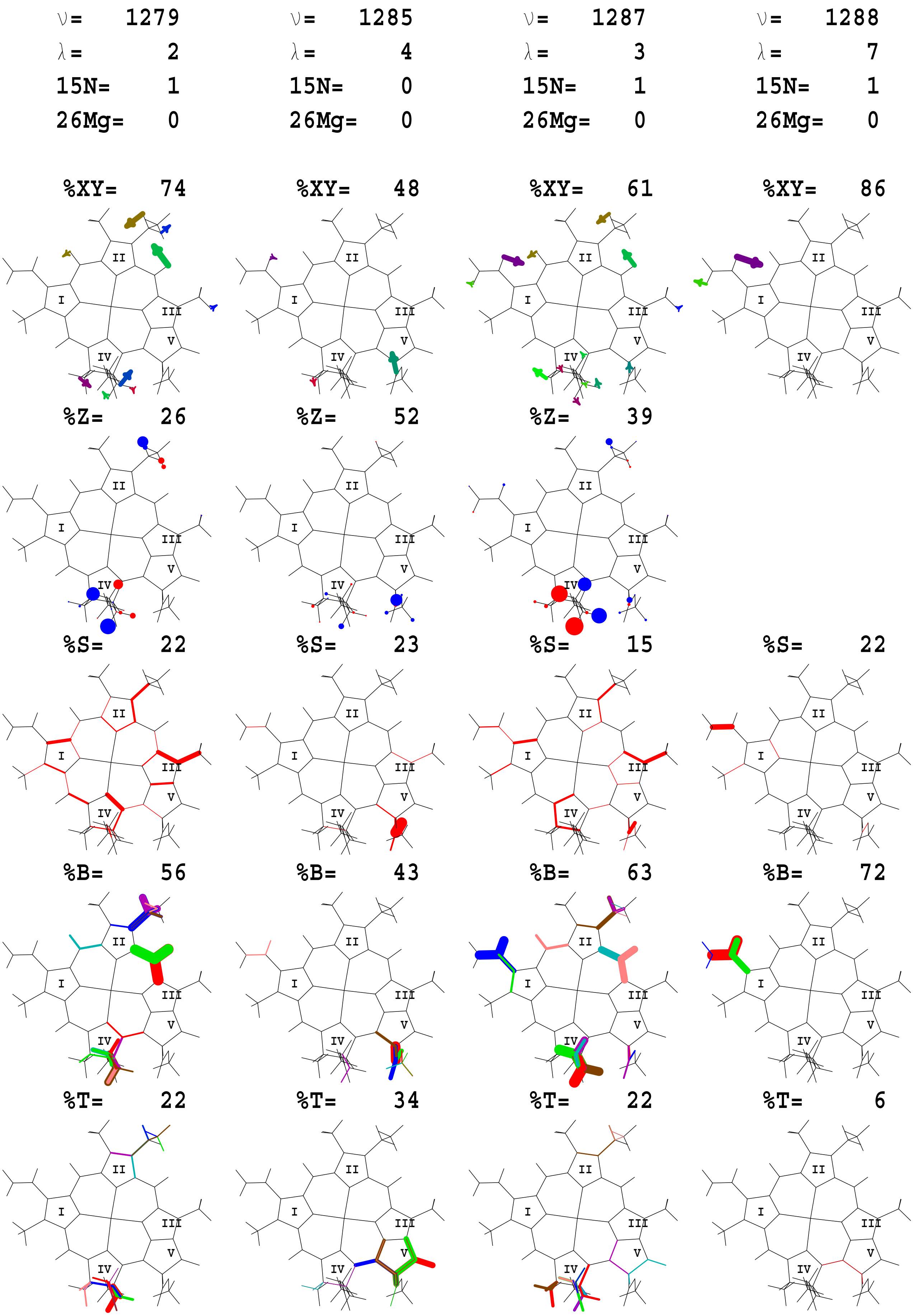

ν= 1279
λ= 2
15N= 1
26Mg= 0
%XY= 74
%Z= 26
%S= 22
%B= 56
%T= 22
ν= 1285
λ= 4
15N= 0
26Mg= 0
%XY= 48
%Z= 52
%S= 23
%B= 43
%T= 34
ν= 1287
λ= 3
15N= 1
26Mg= 0
%XY= 61
%Z= 39
%S= 15
%B= 63
%T= 22
ν= 1288
λ= 7
15N= 1
26Mg= 0
%XY= 86
%S= 22
%B= 72
%T= 6

ν=	1306	1315	1323	1329
λ=	6	5	10	12
15N=	0	1	1	1
26Mg=	0	0	0	0
%XY=	84	47	91	80
%Z=	16	53	9	20
%S=	27	13	30	19
%B=	55	49	61	59
%T=	18	37	9	23

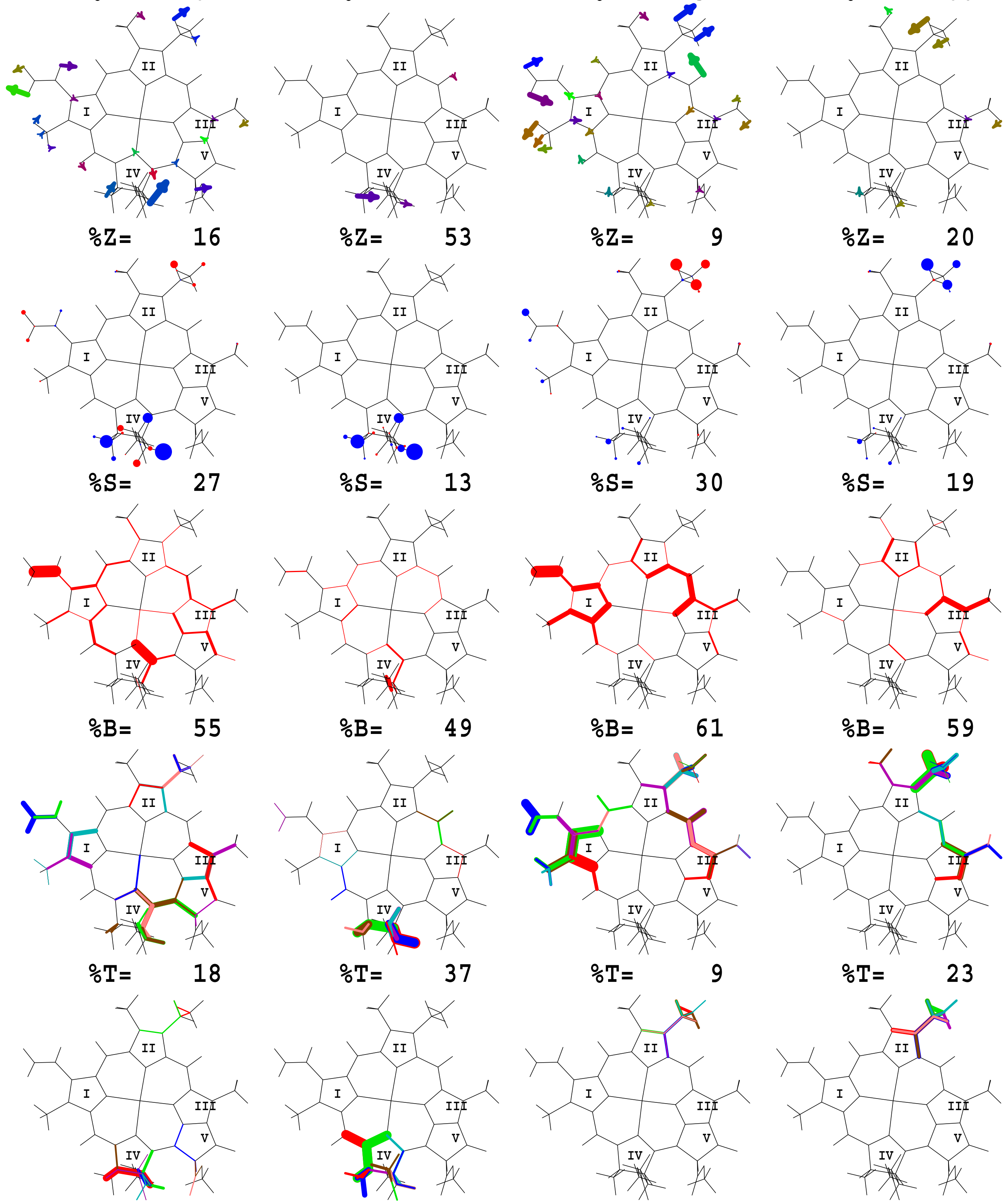

ν= 1332	ν= 1345	ν= 1357	ν= 1359
λ= 17	λ= 3	λ= 7	λ= 0
15N= 2	15N= 1	15N= 3	15N= 1
26Mg= 0	26Mg= 0	26Mg= 0	26Mg= 0

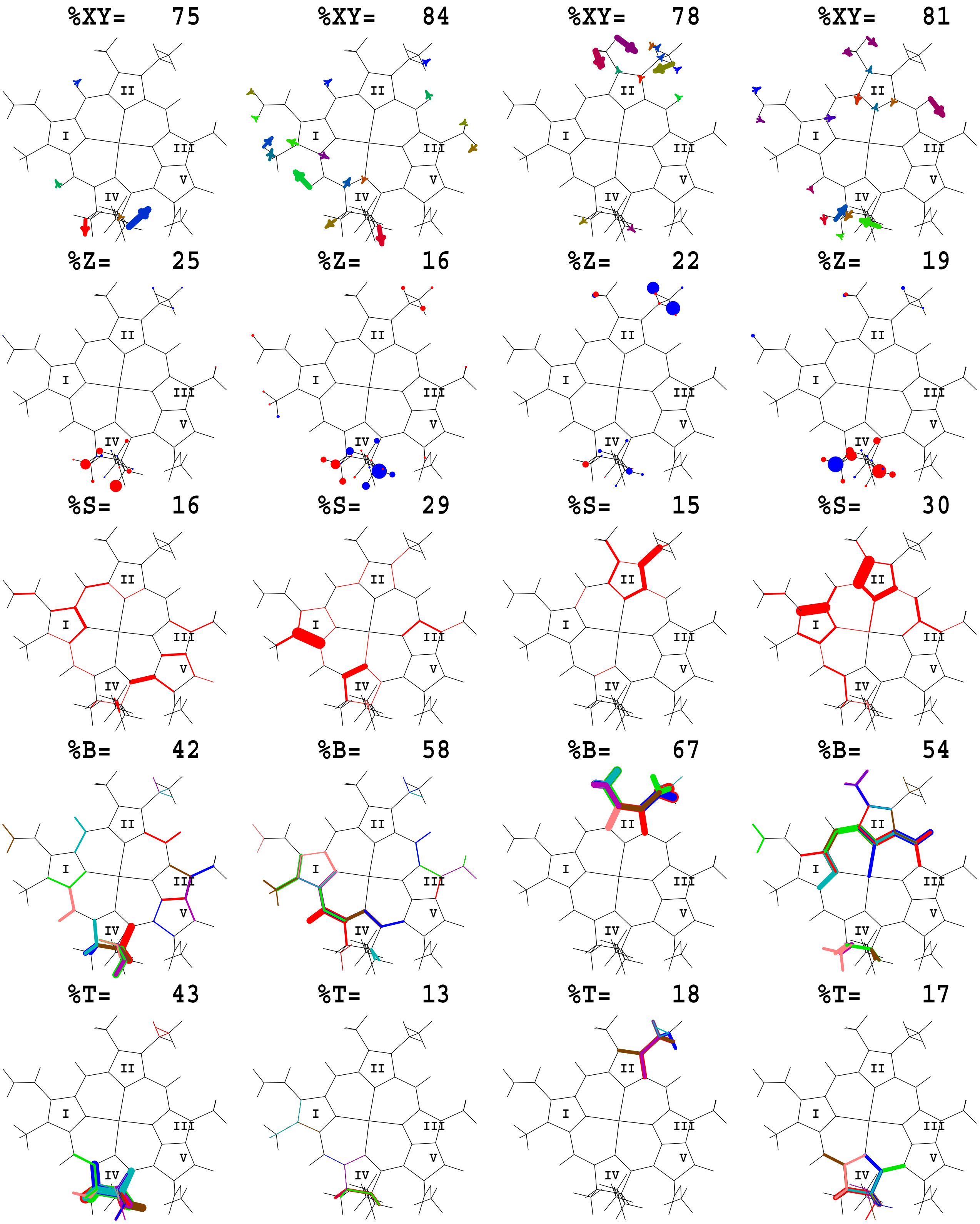

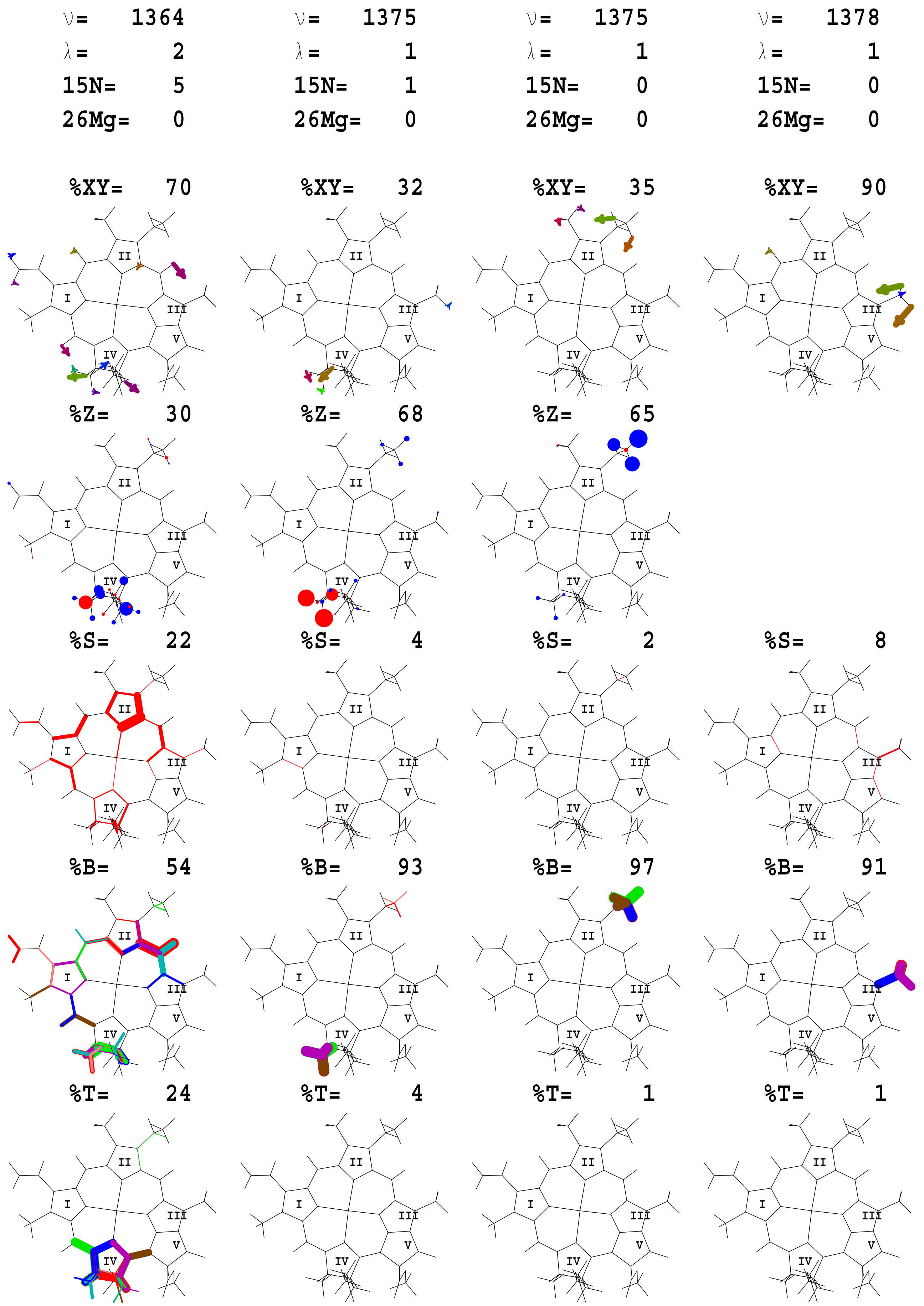
ν= 1364
λ= 2
15N= 5
26Mg= 0
%XY= 70
%Z= 30
%S= 22
%B= 54
%T= 24
ν= 1375
λ= 1
15N= 1
26Mg= 0
%XY= 32
%Z= 68
%S= 4
%B= 93
%T= 4
ν= 1375
λ= 1
15N= 0
26Mg= 0
%XY= 35
%Z= 65
%S= 2
%B= 97
%T= 1
ν= 1378
λ= 1
15N= 0
26Mg= 0
%XY= 90
%S= 8
%B= 91
%T= 1
I
II
III
IV
V

ν= 1381	ν= 1382	ν= 1392	ν= 1399
λ= 4	λ= 1	λ= 7	λ= 0
15N= 1	15N= 1	15N= 0	15N= 0
26Mg= 0	26Mg= 0	26Mg= 0	26Mg= 0

%XY= 97

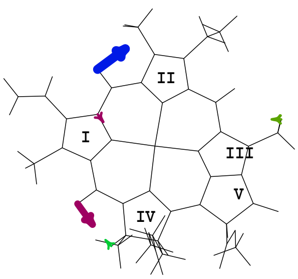

%XY= 90

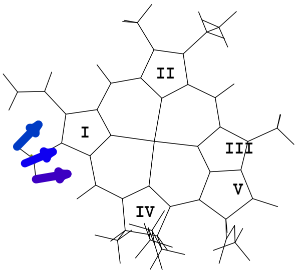

%XY= 91

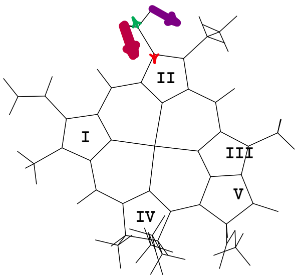

%Z= 10

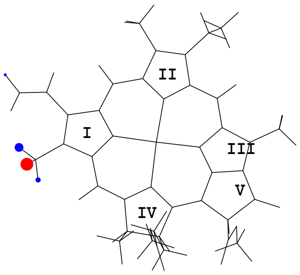

%Z= 78

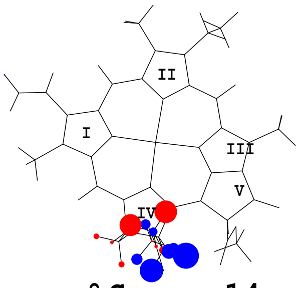

%S= 39

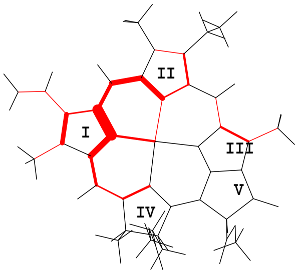

%S= 5

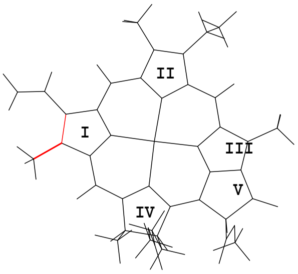

%S= 16

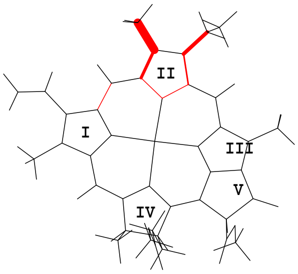

%S= 14

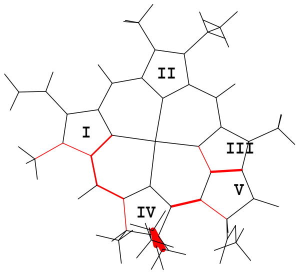

%B= 58

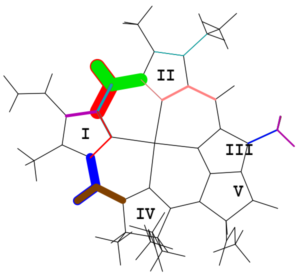

%B= 94

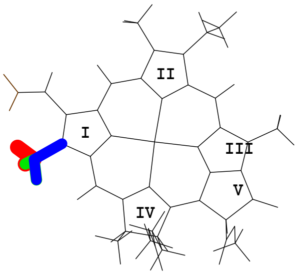

%B= 83

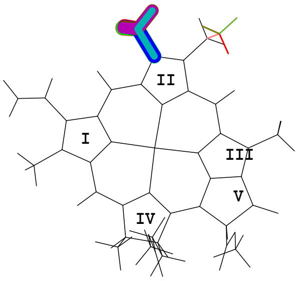

%B= 52

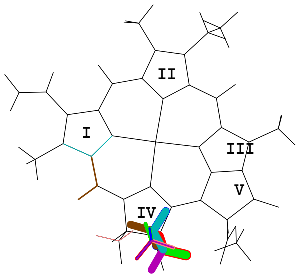

%T= 3

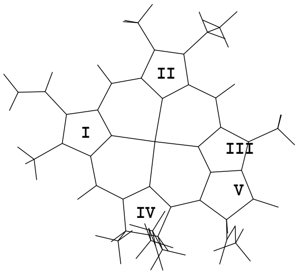

%T= 1

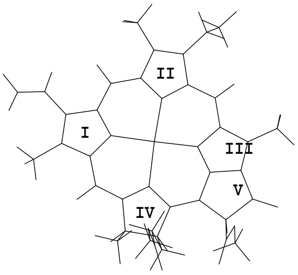

%T= 1

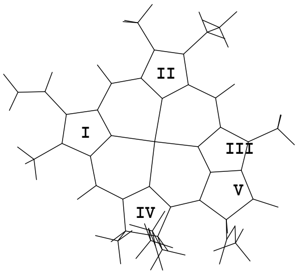

%T= 34

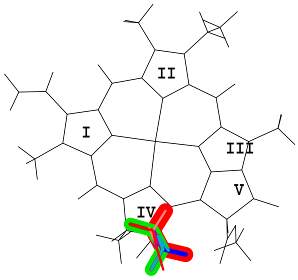

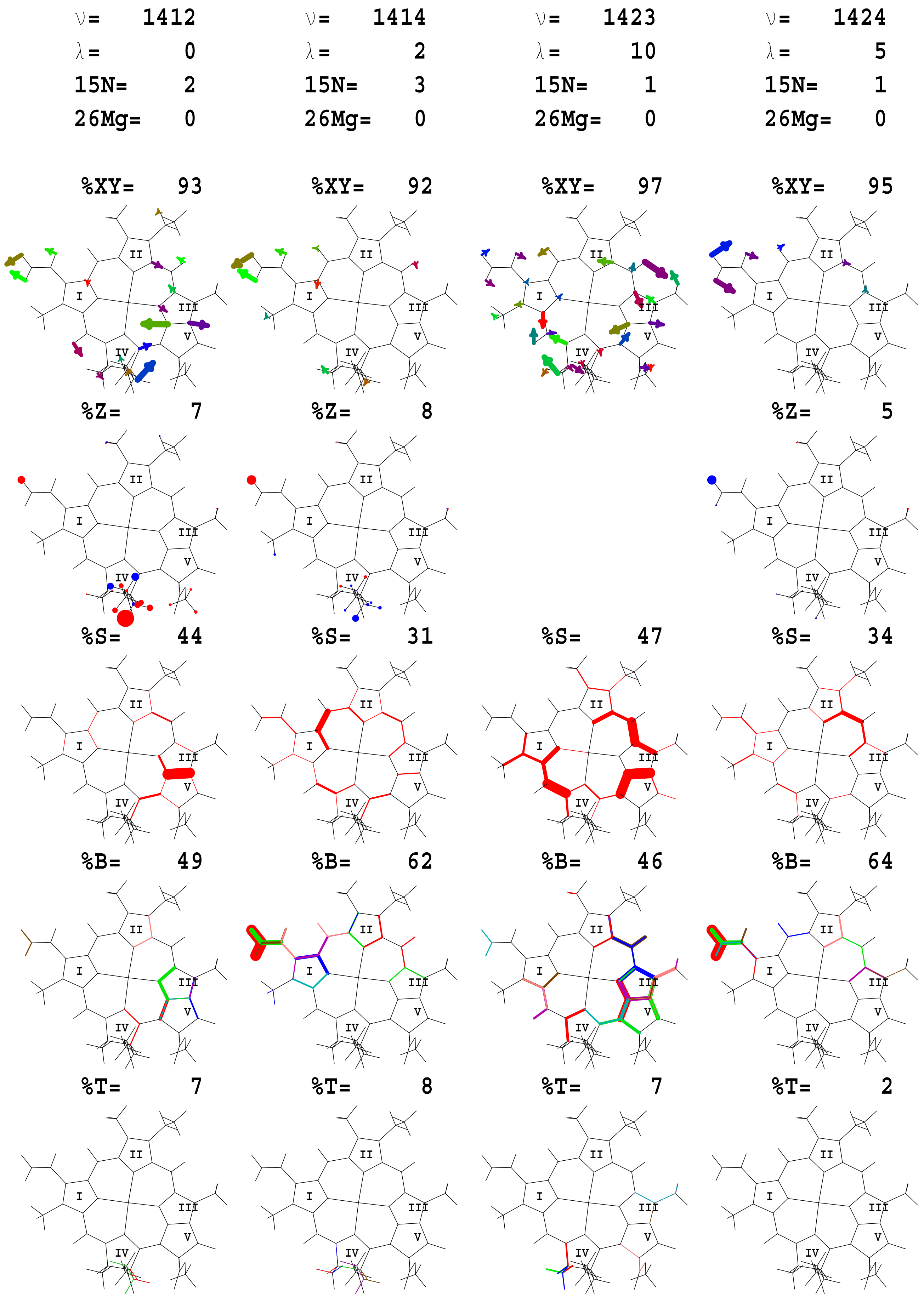

ν= 1412
λ= 0
15N= 2
26Mg= 0
%XY= 93
%Z= 7
%S= 44
%B= 49
%T= 7
ν= 1414
λ= 2
15N= 3
26Mg= 0
%XY= 92
%Z= 8
%S= 31
%B= 62
%T= 8
ν= 1423
λ= 10
15N= 1
26Mg= 0
%XY= 97
%S= 47
%B= 46
%T= 7
ν= 1424
λ= 5
15N= 1
26Mg= 0
%XY= 95
%Z= 5
%S= 34
%B= 64
%T= 2
I
II
III
IV
V

ν= 1431	ν= 1435	ν= 1447	ν= 1459
λ= 0	λ= 0	λ= 0	λ= 0
15N= 0	15N= 0	15N= 0	15N= 1
26Mg= 0	26Mg= 0	26Mg= 0	26Mg= 0

%XY= 29	%XY= 18	%XY= 97	%XY= 77

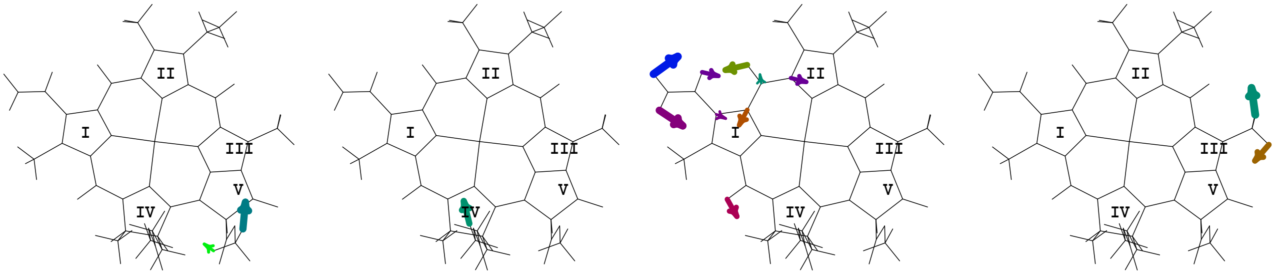

%Z= 71	%Z= 82	%Z= 3	%Z= 23

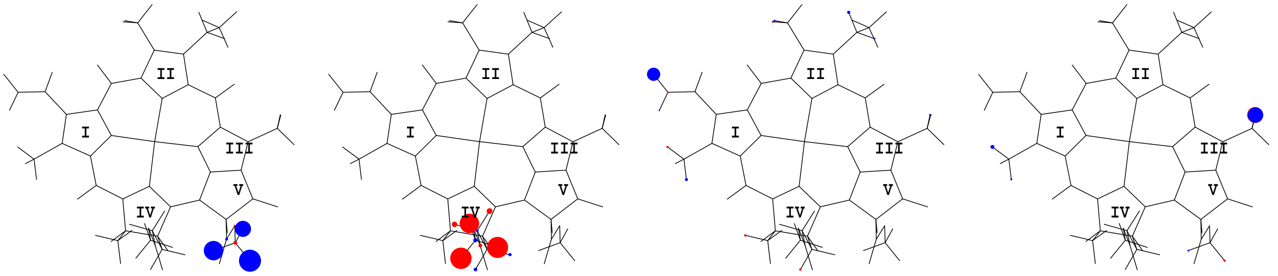

%S= 7	%S= 9	%S= 51	%S= 13

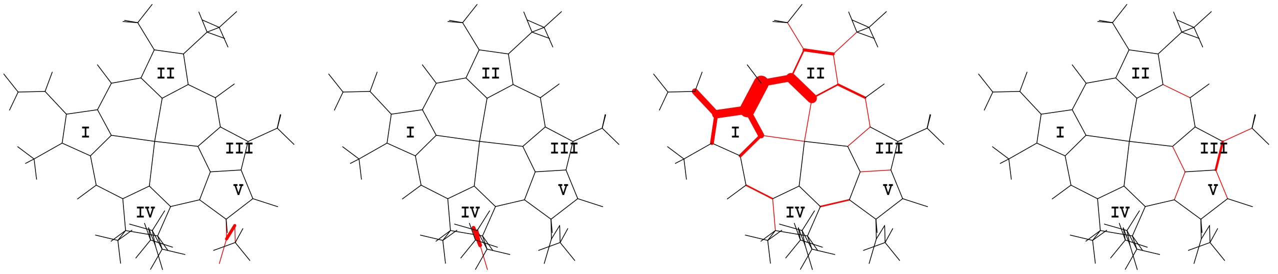

%B= 92	%B= 87	%B= 47	%B= 58

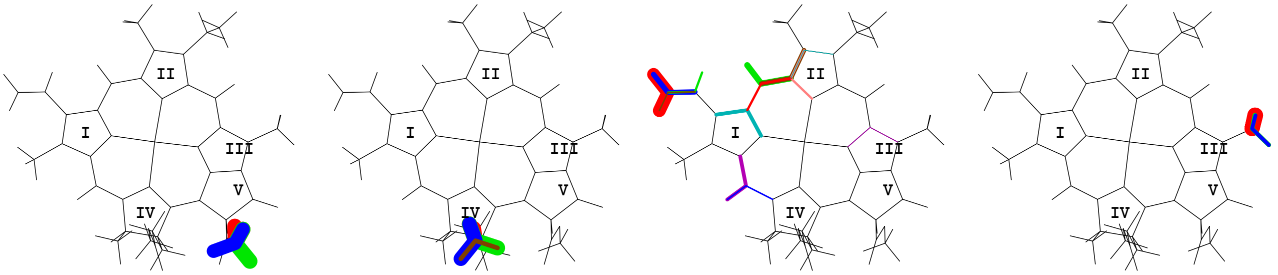

%T= 1	%T= 4	%T= 2	%T= 28

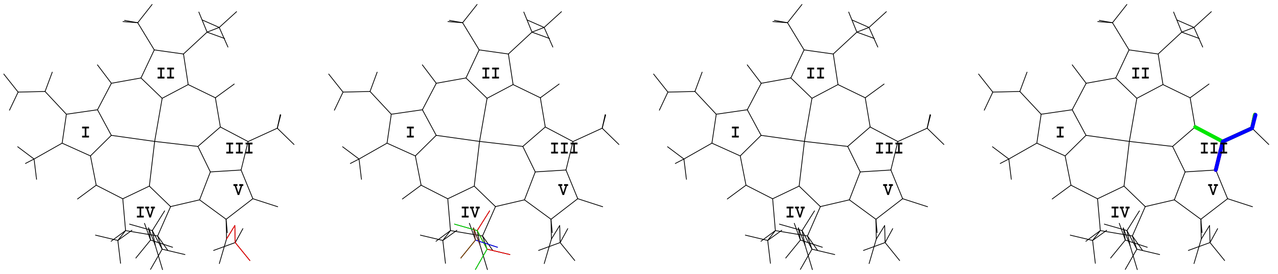

ν= 1459
λ= 0
15N= 0
26Mg= 0

%XY= 63

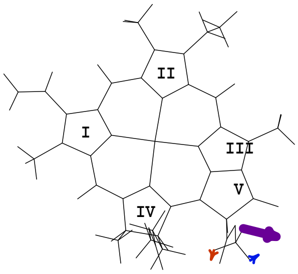

%Z= 37

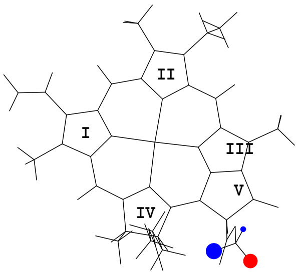

%S= 0

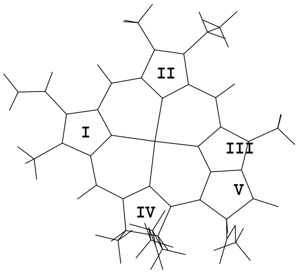

%B= 76

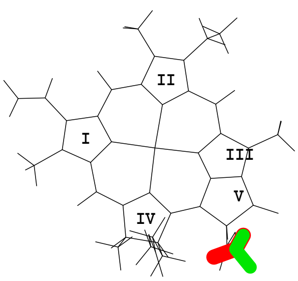

%T= 24

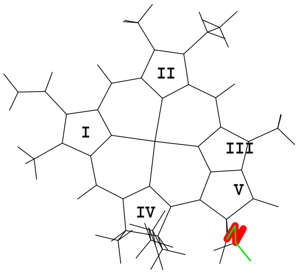

ν= 1459
λ= 0
15N= 1
26Mg= 0

%XY= 69

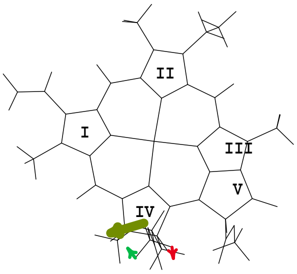

%Z= 31

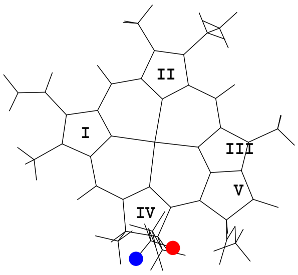

%S= 0

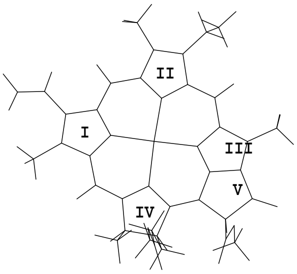

%B= 76

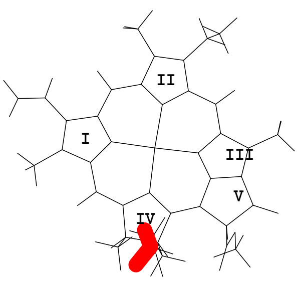

%T= 24

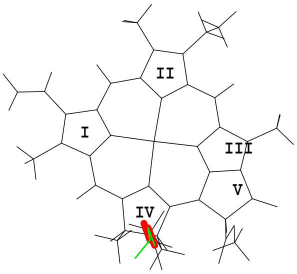

ν= 1460
λ= 0
15N= 0
26Mg= 0

%XY= 98

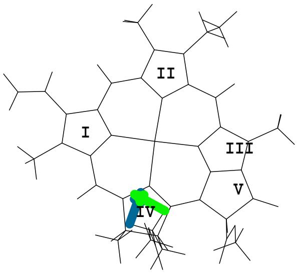

%S= 1

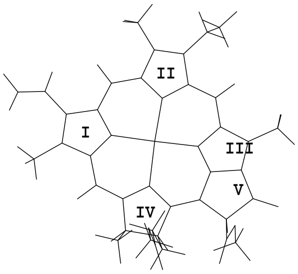

%B= 37

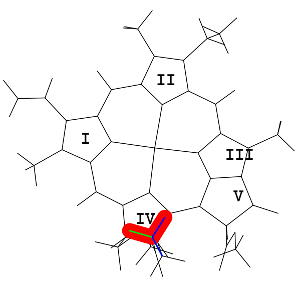

%T= 62

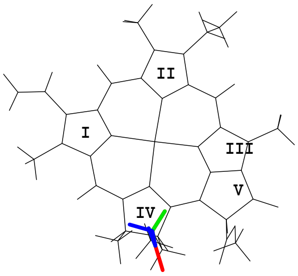

ν= 1462
λ= 0
15N= 0
26Mg= 0

%XY= 45

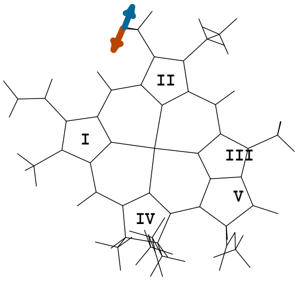

%Z= 55

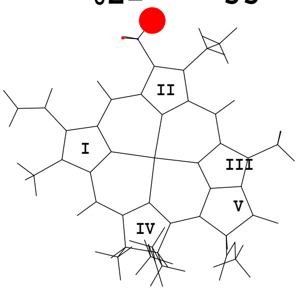

%S= 0

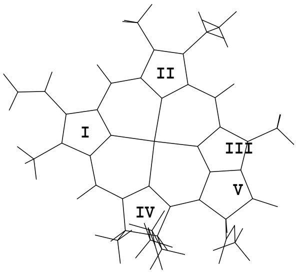

%B= 64

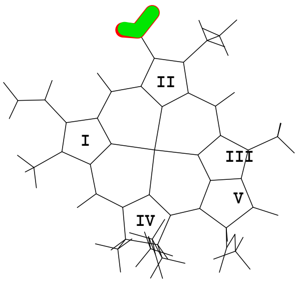

%T= 35

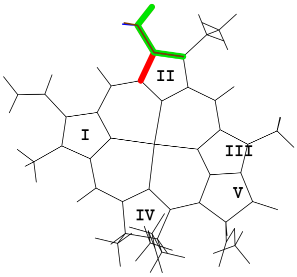

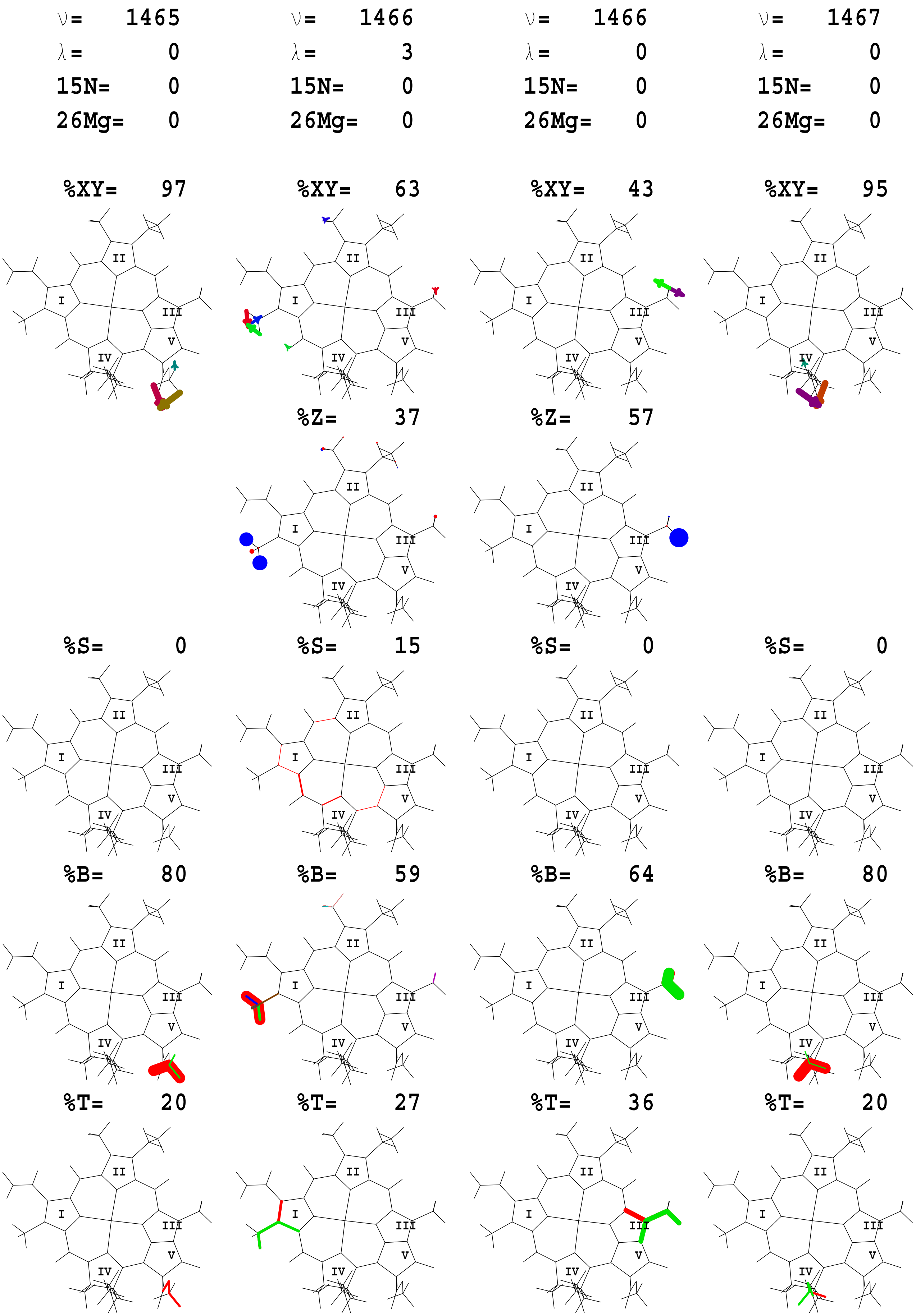
ν= 1465
λ= 0
15N= 0
26Mg= 0
%XY= 97
%S= 0
%B= 80
%T= 20
ν= 1466
λ= 3
15N= 0
26Mg= 0
%XY= 63
%Z= 37
%S= 15
%B= 59
%T= 27
ν= 1466
λ= 0
15N= 0
26Mg= 0
%XY= 43
%Z= 57
%S= 0
%B= 64
%T= 36
ν= 1467
λ= 0
15N= 0
26Mg= 0
%XY= 95
%S= 0
%B= 80
%T= 20

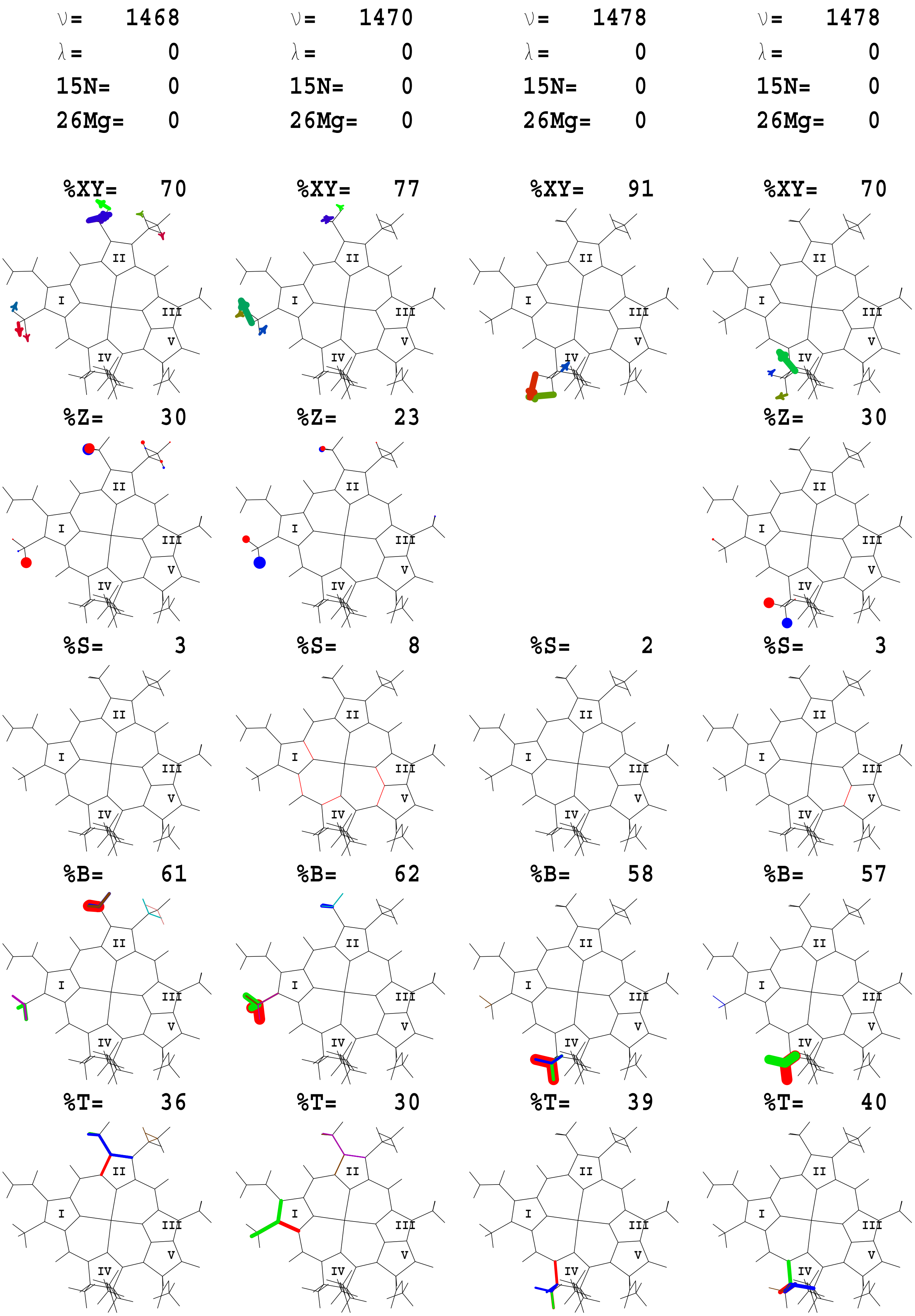
ν= 1468
λ= 0
15N= 0
26Mg= 0
%XY= 70
%Z= 30
%S= 3
%B= 61
%T= 36
ν= 1470
λ= 0
15N= 0
26Mg= 0
%XY= 77
%Z= 23
%S= 8
%B= 62
%T= 30
ν= 1478
λ= 0
15N= 0
26Mg= 0
%XY= 91
%S= 2
%B= 58
%T= 39
ν= 1478
λ= 0
15N= 0
26Mg= 0
%XY= 70
%Z= 30
%S= 3
%B= 57
%T= 40

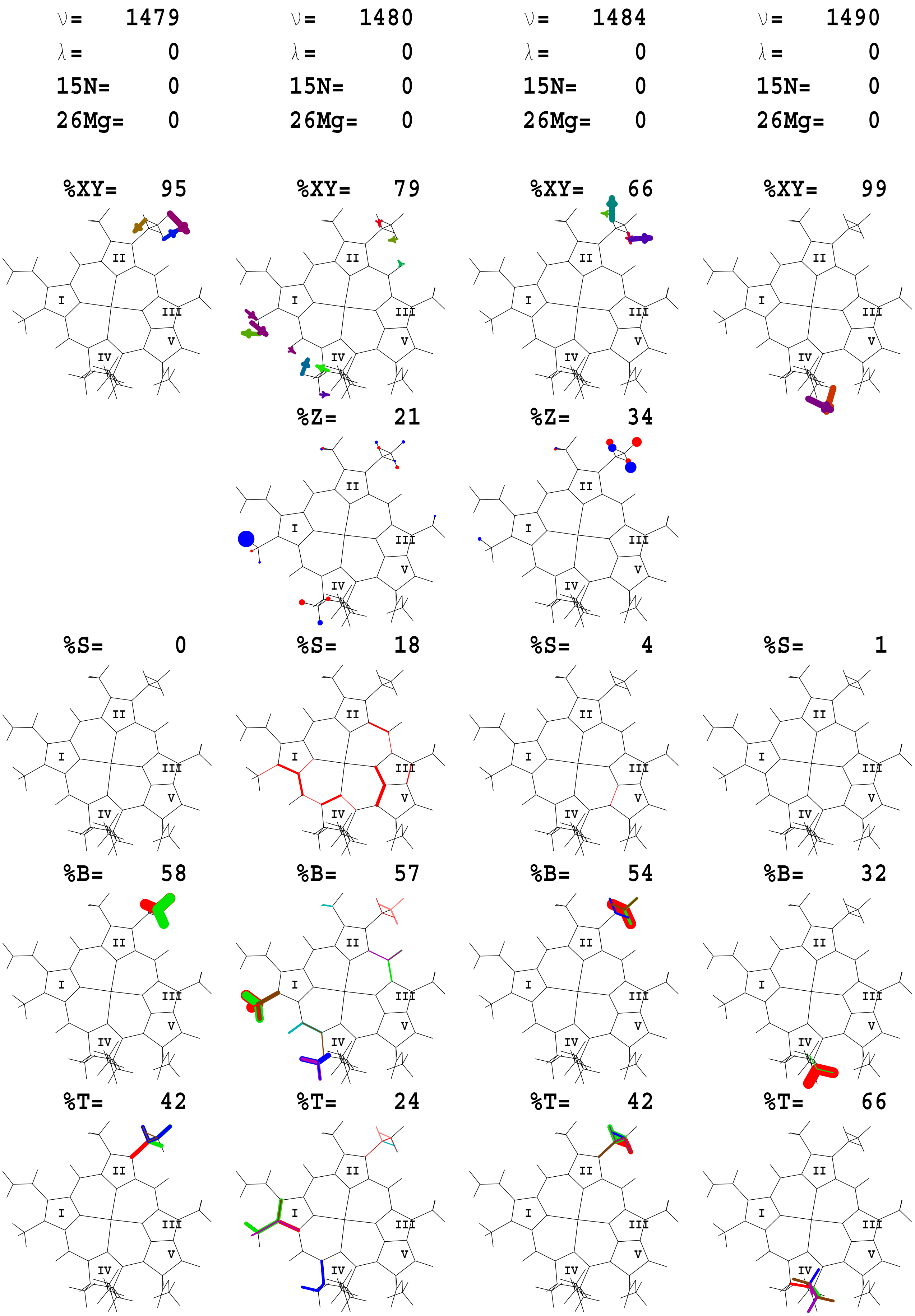
ν= 1479
λ= 0
15N= 0
26Mg= 0
%XY= 95
%S= 0
%B= 58
%T= 42
ν= 1480
λ= 0
15N= 0
26Mg= 0
%XY= 79
%Z= 21
%S= 18
%B= 57
%T= 24
ν= 1484
λ= 0
15N= 0
26Mg= 0
%XY= 66
%Z= 34
%S= 4
%B= 54
%T= 42
ν= 1490
λ= 0
15N= 0
26Mg= 0
%XY= 99
%S= 1
%B= 32
%T= 66

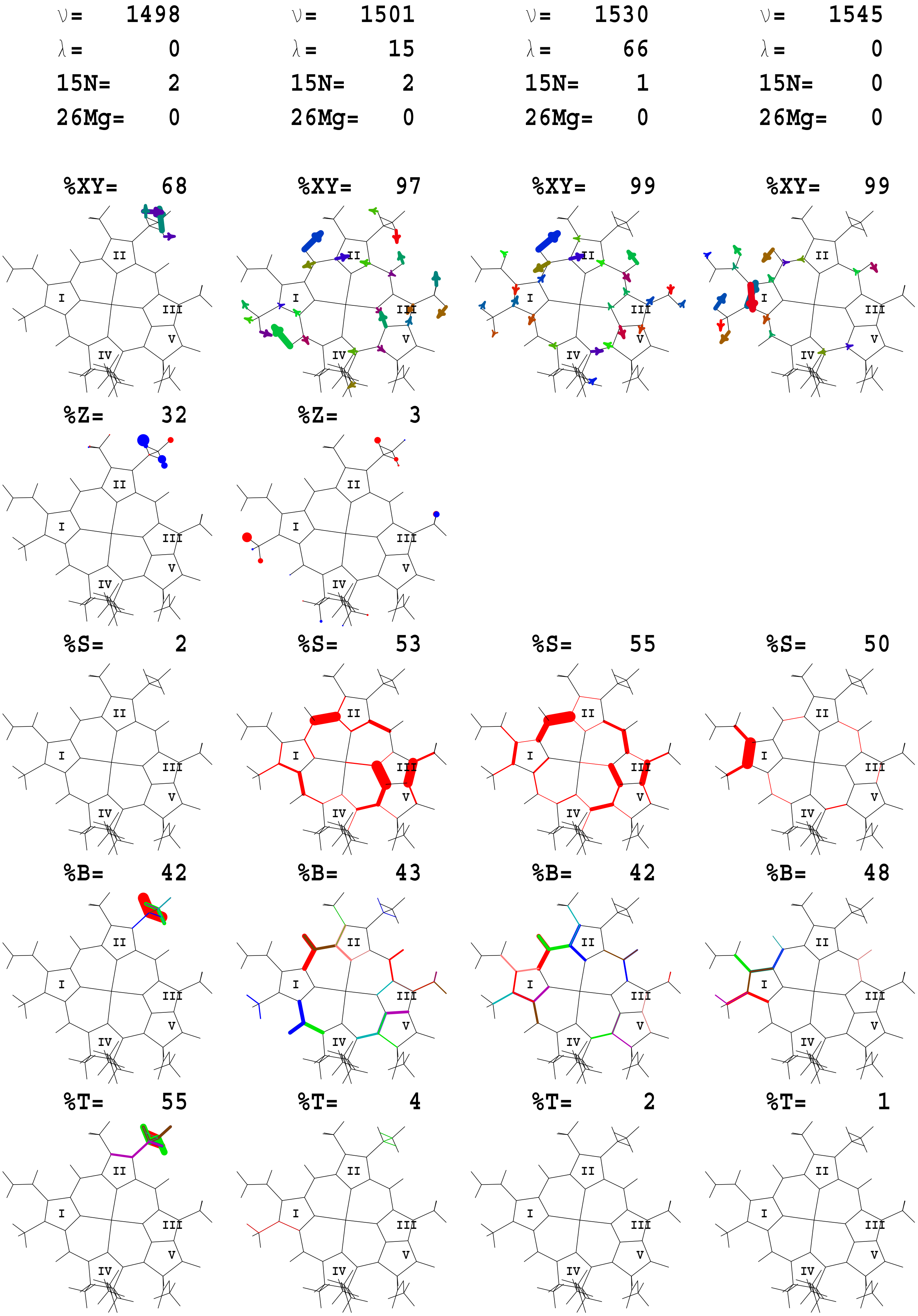

ν= 1498
λ= 0
15N= 2
26Mg= 0
ν= 1501
λ= 15
15N= 2
26Mg= 0
ν= 1530
λ= 66
15N= 1
26Mg= 0
ν= 1545
λ= 0
15N= 0
26Mg= 0
%XY= 68
%XY= 97
%XY= 99
%XY= 99
%Z= 32
%Z= 3
%S= 2
%S= 53
%S= 55
%S= 50
%B= 42
%B= 43
%B= 42
%B= 48
%T= 55
%T= 4
%T= 2
%T= 1
I
II
III
IV
V

ν= 1563	ν= 1570	ν= 1595	ν= 1608
λ= 7	λ= 42	λ= 29	λ= 3
15N= 1	15N= 0	15N= 0	15N= 0
26Mg= 0	26Mg= 0	26Mg= 0	26Mg= 0

%XY= 99 %XY= 100 %XY= 100 %XY= 99

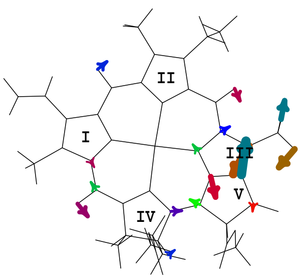
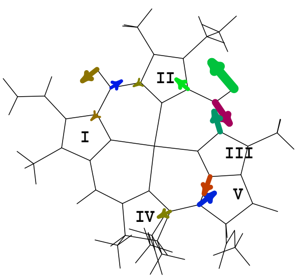
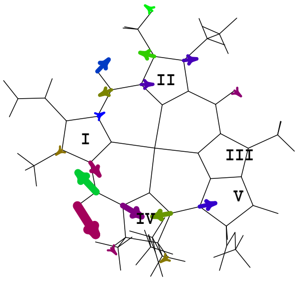
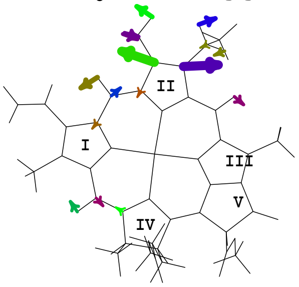

%S= 46 %S= 60 %S= 56 %S= 59

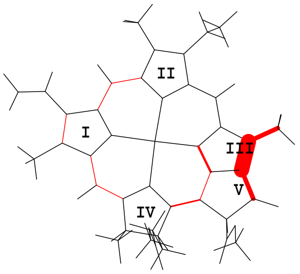
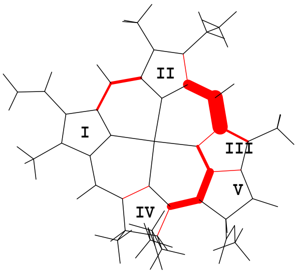
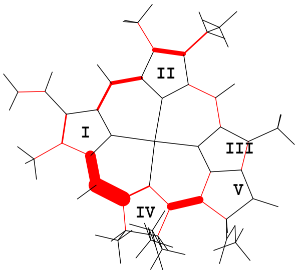
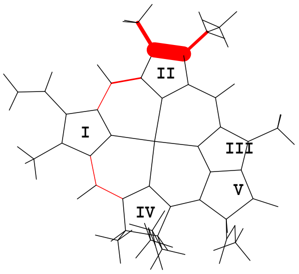

%B= 53 %B= 38 %B= 41 %B= 39

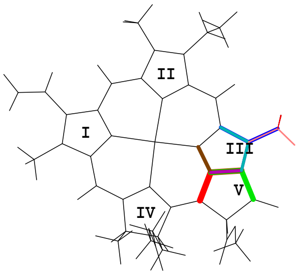
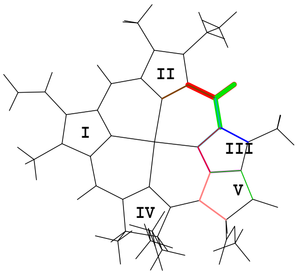
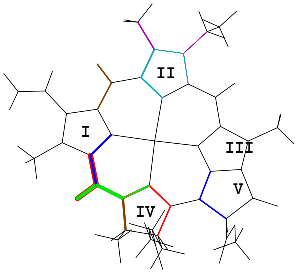
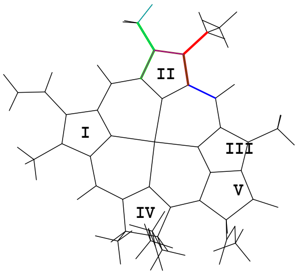

%T= 1 %T= 2 %T= 3 %T= 2

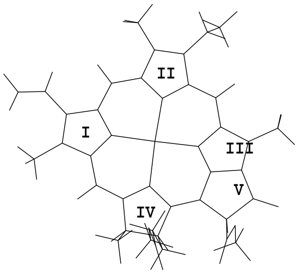
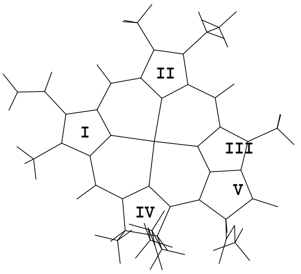
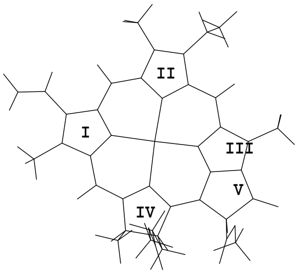
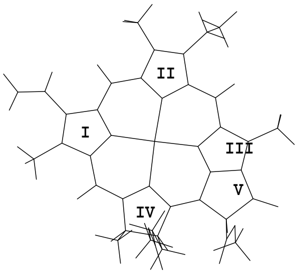

ν= 1648	ν= 1749	ν= 1759	ν= 1774
λ= 0	λ= 1	λ= 0	λ= 0
15N= 0	15N= 0	15N= 0	15N= 0
26Mg= 0	26Mg= 0	26Mg= 0	26Mg= 0

%XY= 91

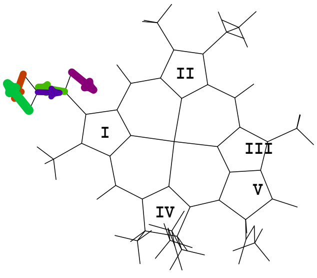

%XY= 100

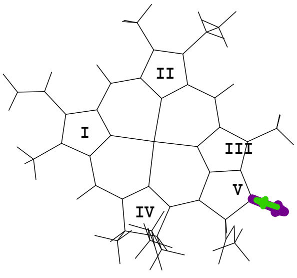

%XY= 99

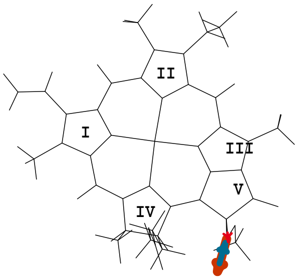

%XY= 96

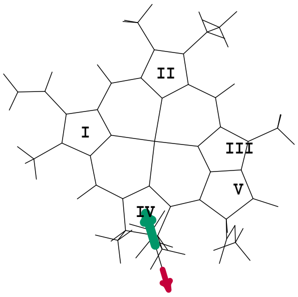

%S= 62

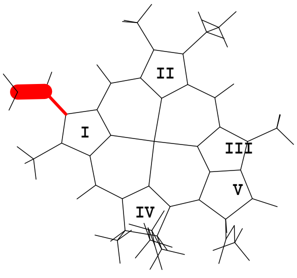

%S= 78

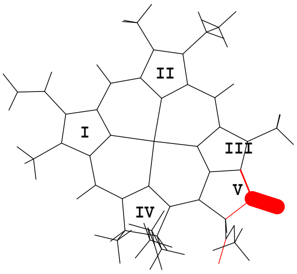

%S= 77

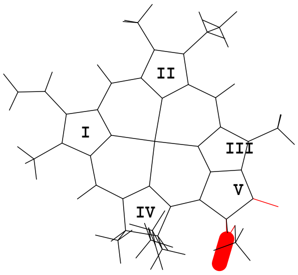

%S= 79

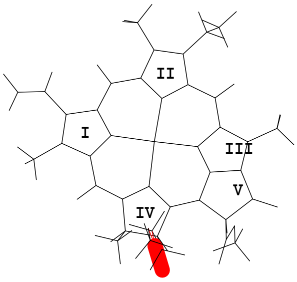

%B= 37

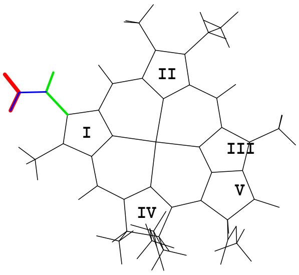

%B= 19

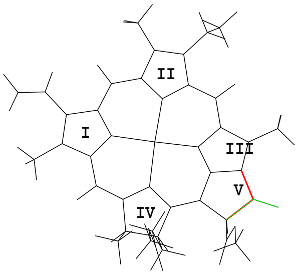

%B= 19

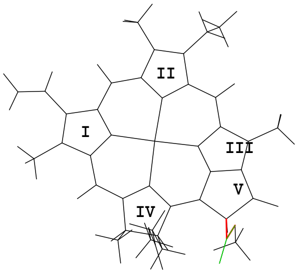

%B= 21

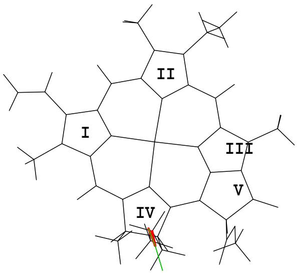

%T= 1

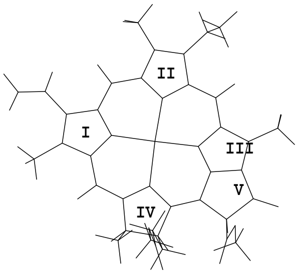

%T= 3

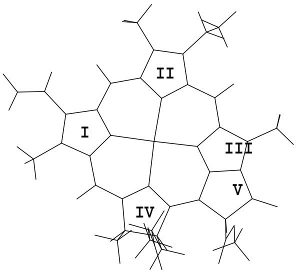

%T= 5

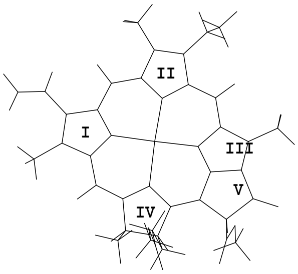

%T= 0

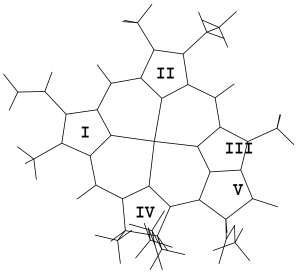

ν= 2910	ν= 2912	ν= 2918	ν= 2923
λ= 0	λ= 0	λ= 0	λ= 0
15N= 0	15N= 0	15N= 0	15N= 0
26Mg= 0	26Mg= 0	26Mg= 0	26Mg= 0

%XY= 44

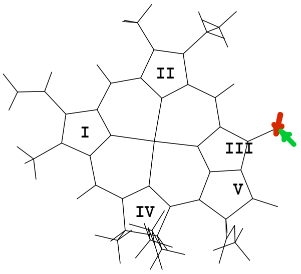

%XY= 47

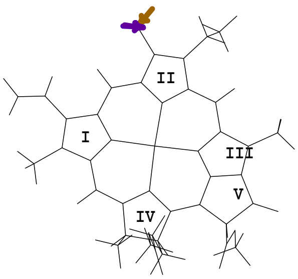

%XY= 73

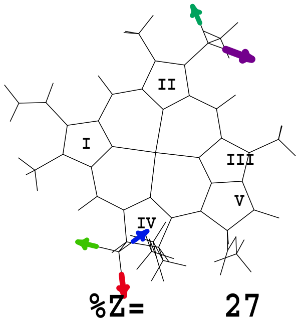

%Z= 64

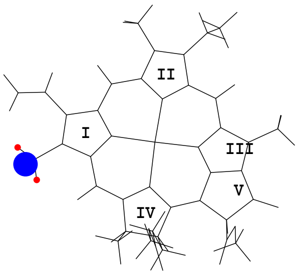

%Z= 56

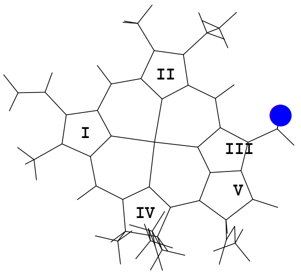

%Z= 53

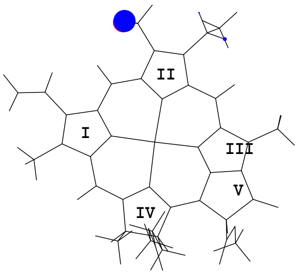

%Z= 27

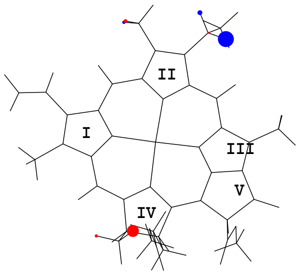

%S= 100

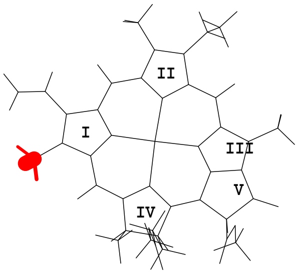

%S= 100

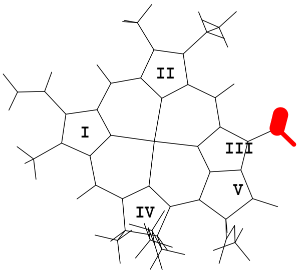

%S= 100

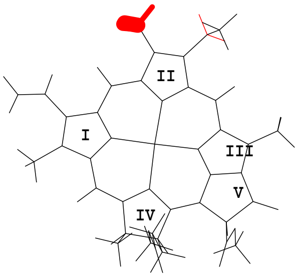

%S= 100

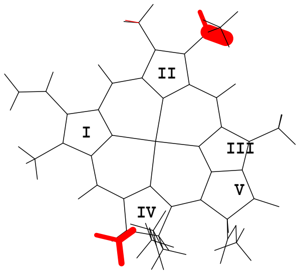

%B= 0

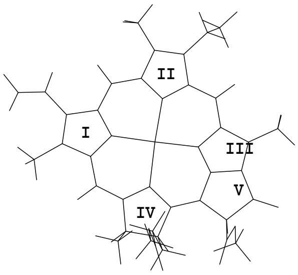

%B= 0

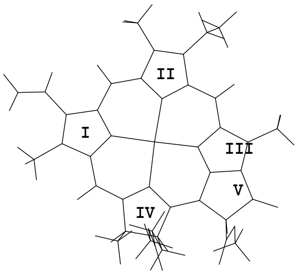

%B= 0

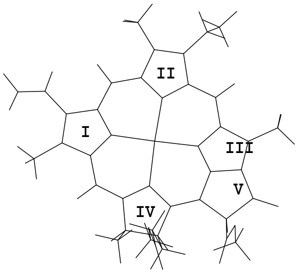

%B= 0

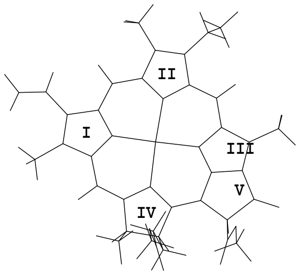

%T= 0

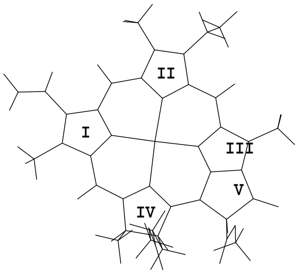

%T= 0

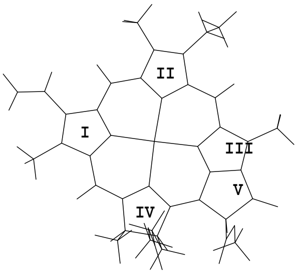

%T= 0

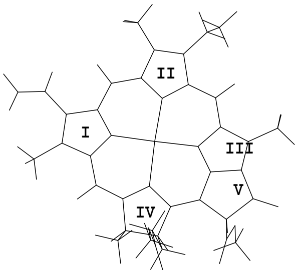

%T= 0

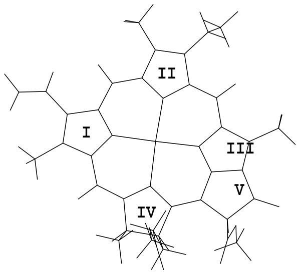

ν= 2923
λ= 0
15N= 0
26Mg= 0

%XY= 74

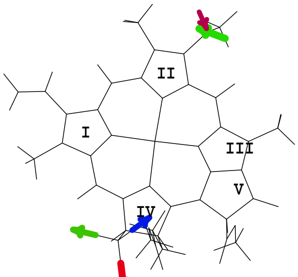

%Z= 26

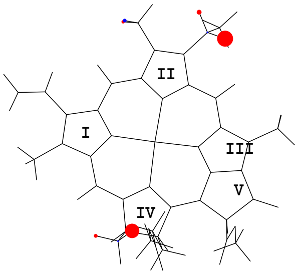

%S= 100

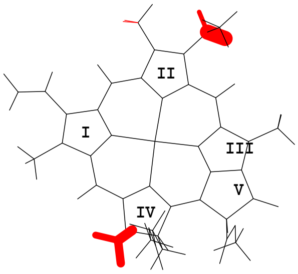

%B= 0

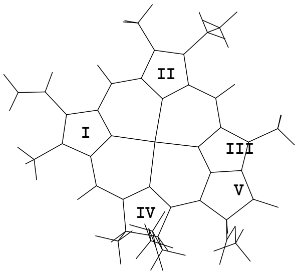

%T= 0

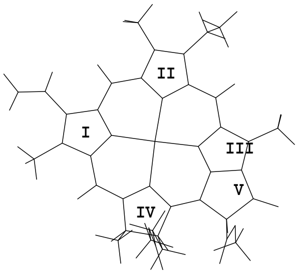

ν= 2927
λ= 0
15N= 0
26Mg= 0

%XY= 82

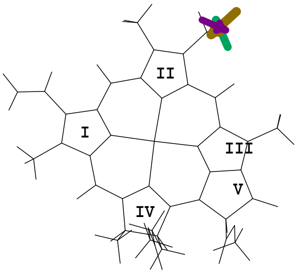

%Z= 18

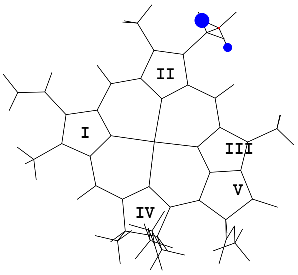

%S= 100

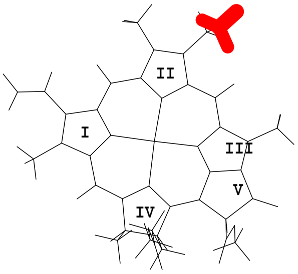

%B= 0

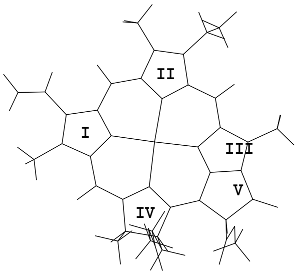

%T= 0

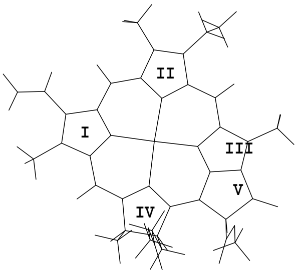

ν= 2929
λ= 0
15N= 0
26Mg= 0

%XY= 79

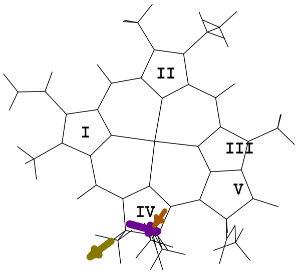

%Z= 21

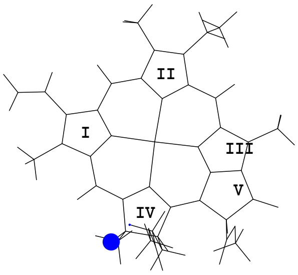

%S= 99

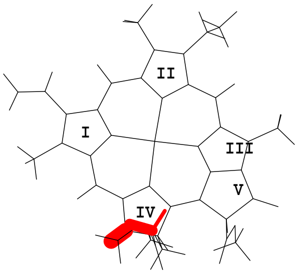

%B= 0

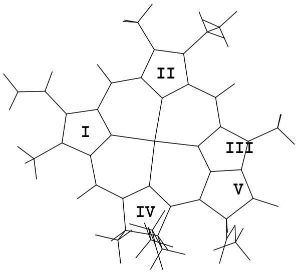

%T= 1

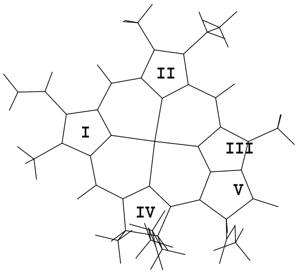

ν= 2931
λ= 0
15N= 0
26Mg= 0

%XY= 80

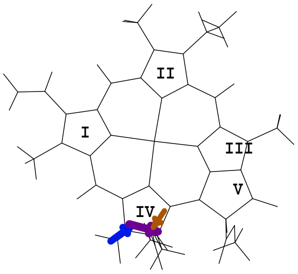

%Z= 20

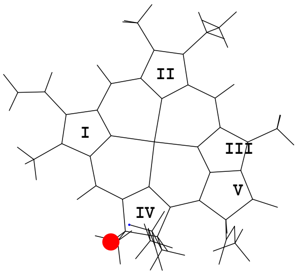

%S= 99

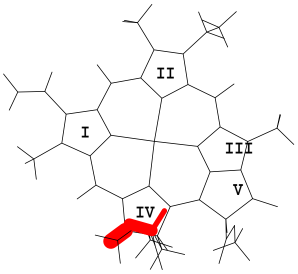

%B= 0

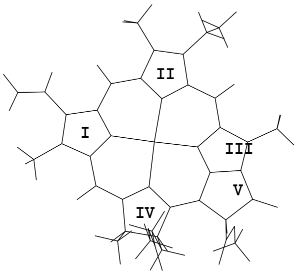

%T= 1

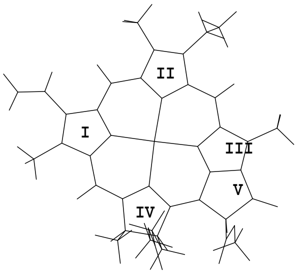

ν= 2947	ν= 2949	ν= 2949	ν= 2960
λ= 0	λ= 0	λ= 0	λ= 0
15N= 0	15N= 0	15N= 0	15N= 0
26Mg= 0	26Mg= 0	26Mg= 0	26Mg= 0

%XY= 89

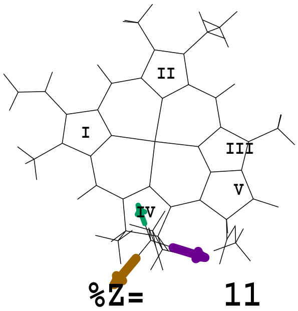

%XY= 96

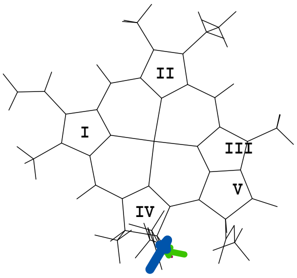

%XY= 87

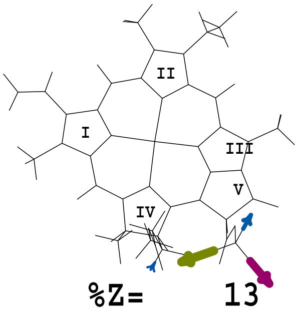

%XY= 83

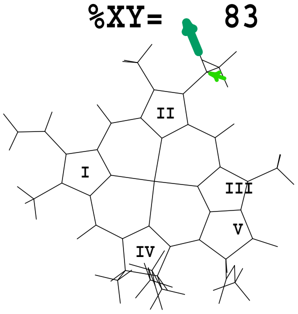

%Z= 11

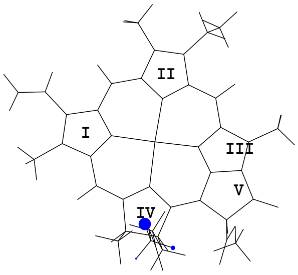

%Z= 13

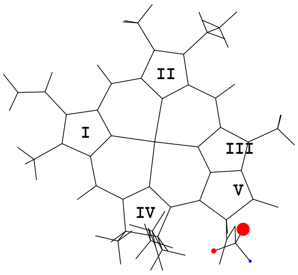

%S= 100

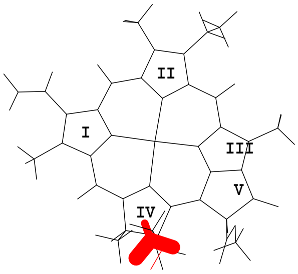

%S= 99

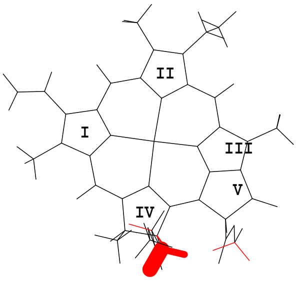

%S= 100

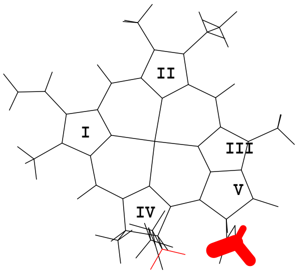

%S= 98

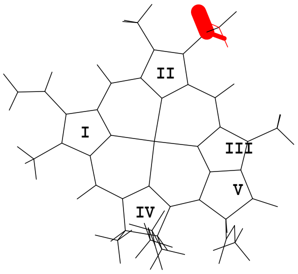

%B= 0

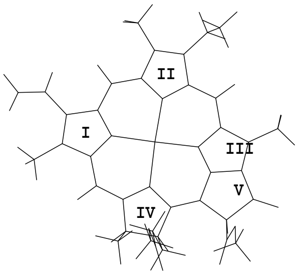

%B= 0

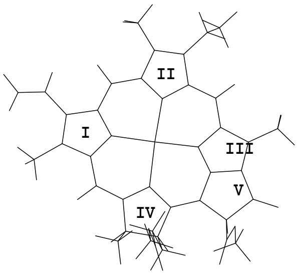

%B= 0

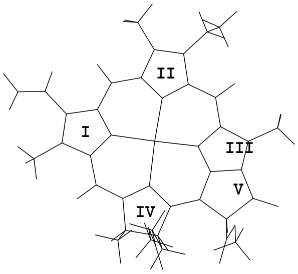

%B= 1

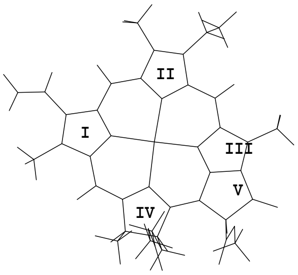

%T= 0

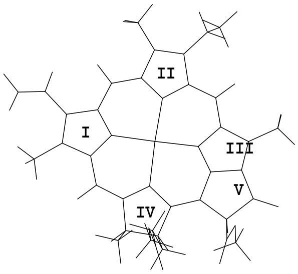

%T= 1

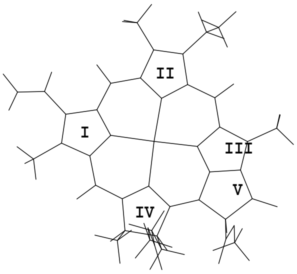

%T= 0

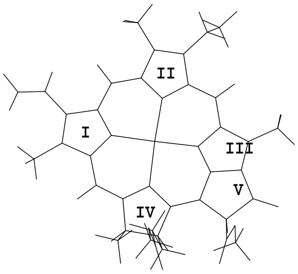

%T= 1

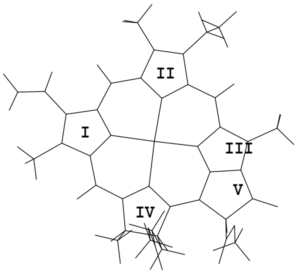

ν= 2961
λ= 0
15N= 0
26Mg= 0

ν= 2964
λ= 0
15N= 0
26Mg= 0

ν= 2966
λ= 0
15N= 0
26Mg= 0

ν= 2969
λ= 0
15N= 0
26Mg= 0

%XY= 33

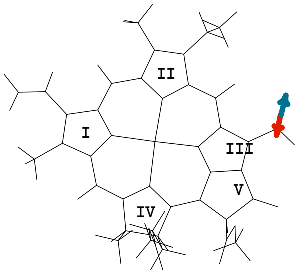

%XY= 62

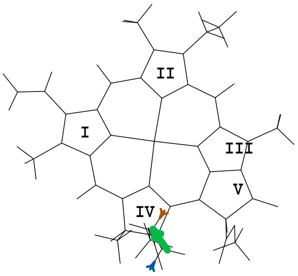

%XY= 89

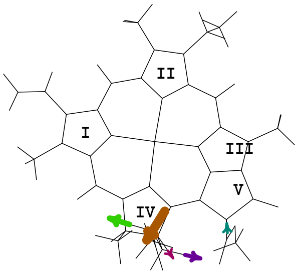

%XY= 42

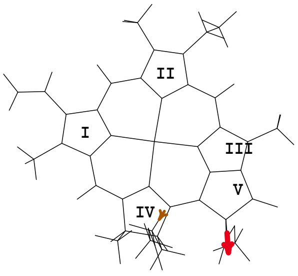

%Z= 67

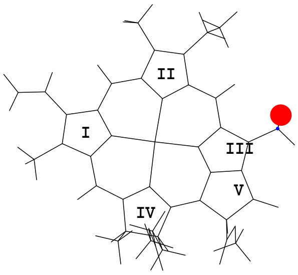

%Z= 38

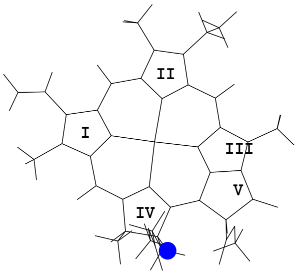

%Z= 58

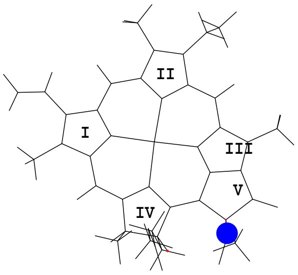

%S= 99

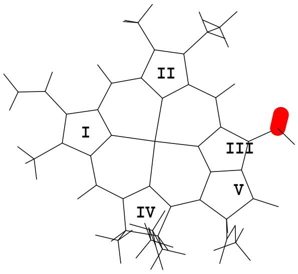

%S= 98

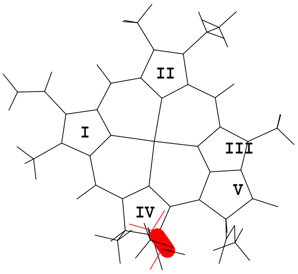

%S= 98

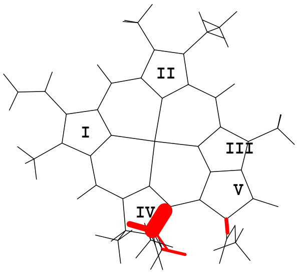

%S= 98

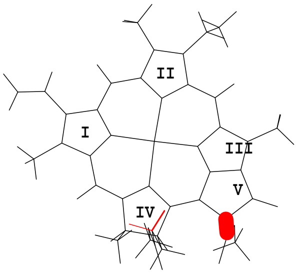

%B= 0

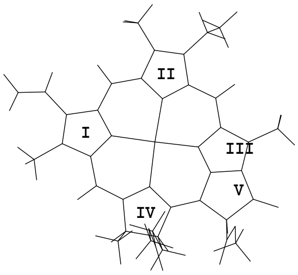

%B= 0

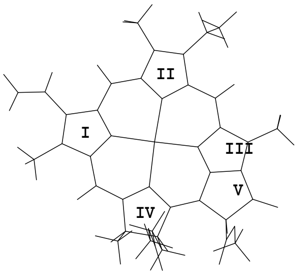

%B= 0

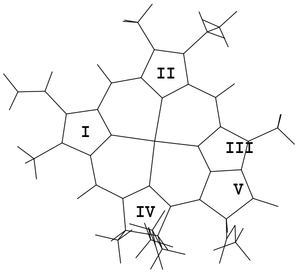

%B= 0

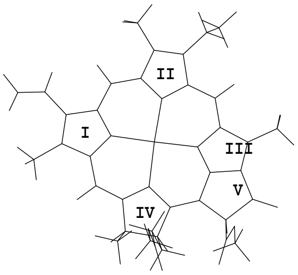

%T= 1

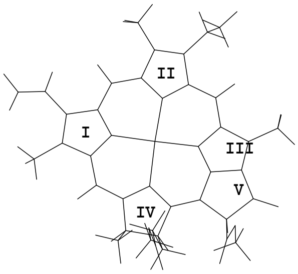

%T= 1

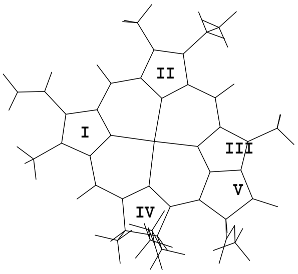

%T= 1

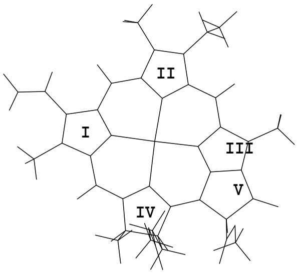

%T= 1

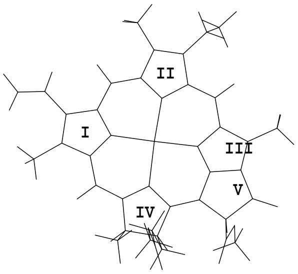

ν=	2969	2969	2992	2994
λ=	0	0	0	0
15N=	0	0	0	0
26Mg=	0	0	0	0

%XY=	39	50	98	77
%Z=	61	50		23
%S=	99	99	99	99
%B=	0	0	0	0
%T=	1	1	1	1

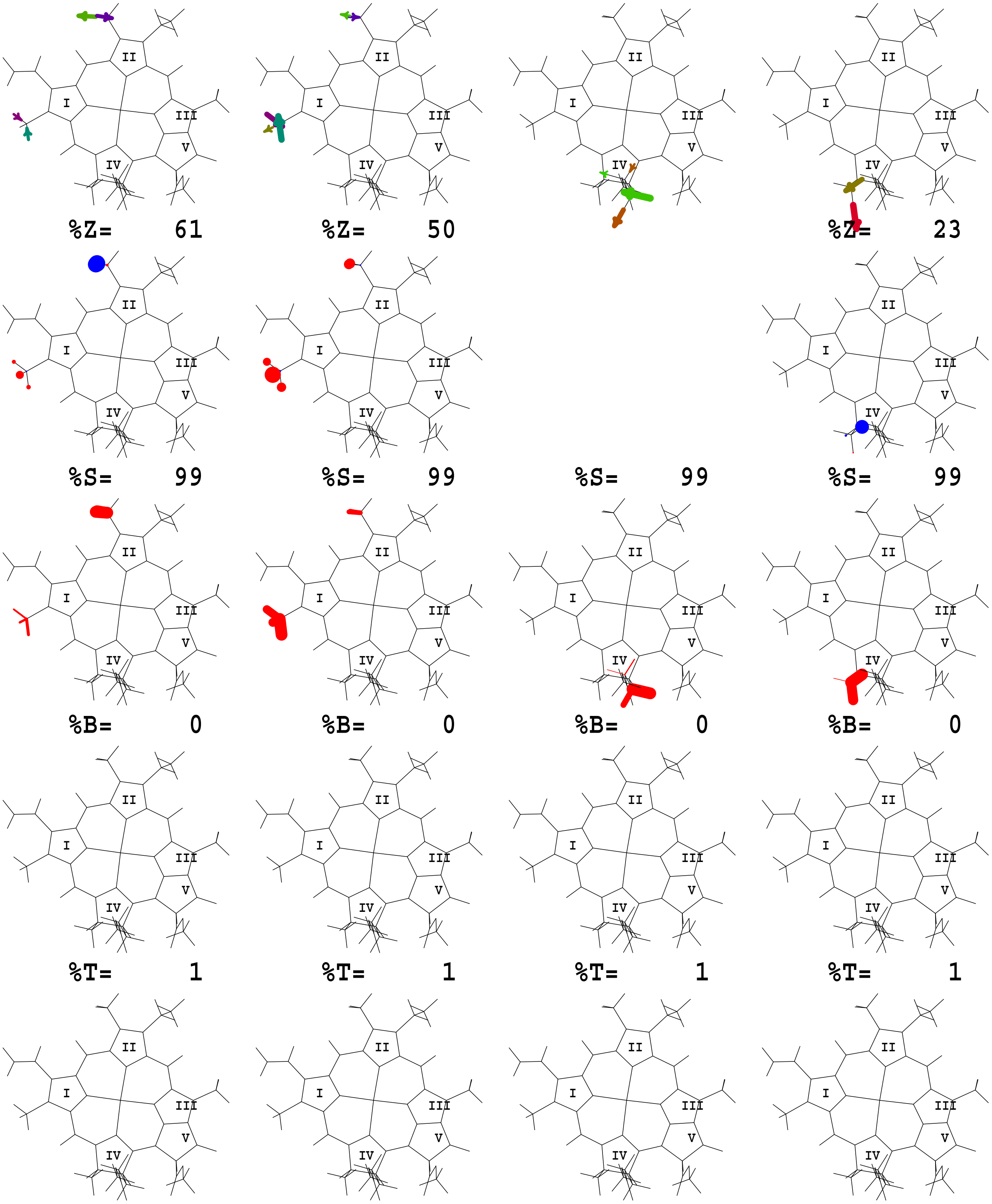

ν= 2997

λ= 0

15N= 0

26Mg= 0

%XY= 90

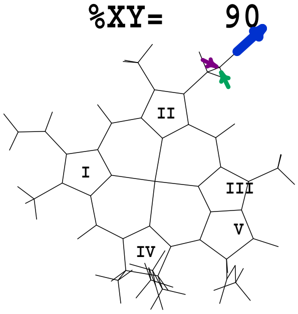

%S= 99

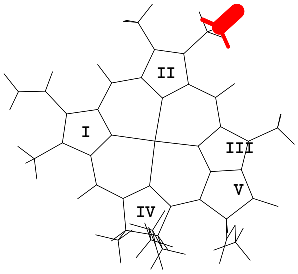

%B= 0

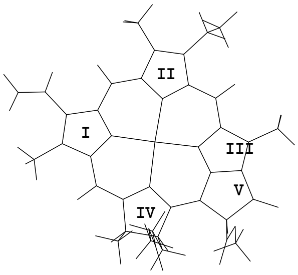

%T= 0

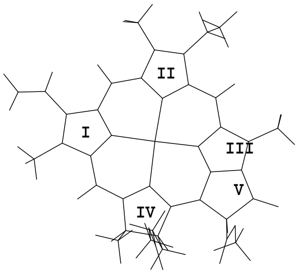

ν= 2998

λ= 0

15N= 0

26Mg= 0

%XY= 78

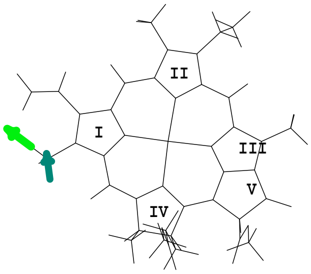

%Z= 22

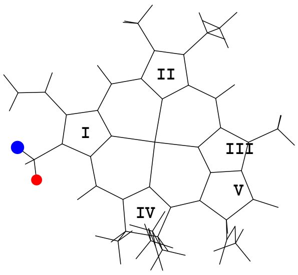

%S= 99

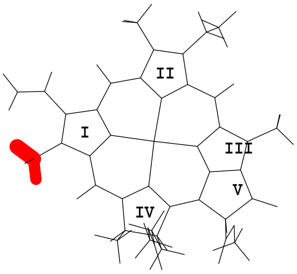

%B= 1

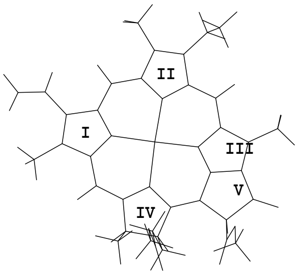

%T= 0

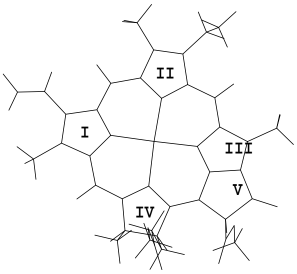

ν= 3000

λ= 0

15N= 0

26Mg= 0

%XY= 77

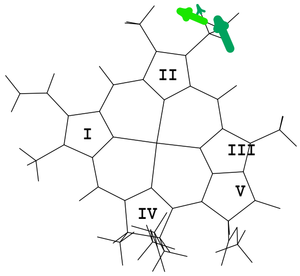

%Z= 23

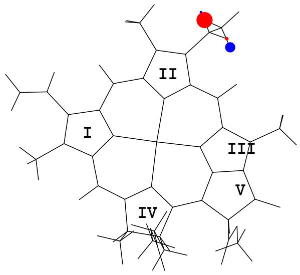

%S= 99

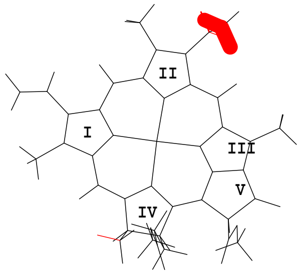

%B= 0

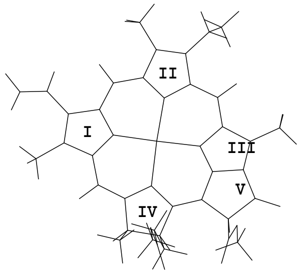

%T= 0

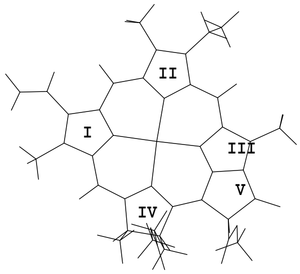

ν= 3000

λ= 0

15N= 0

26Mg= 0

%XY= 93

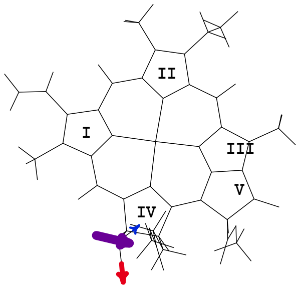

%S= 99

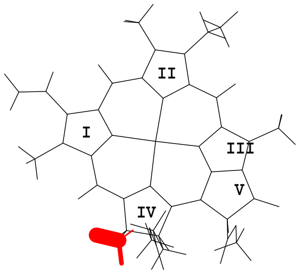

%B= 0

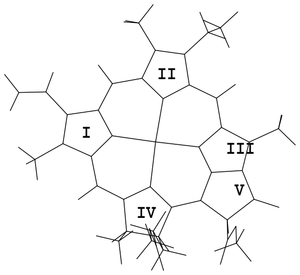

%T= 0

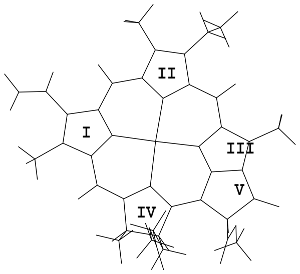

ν= 3007
λ= 0
15N= 0
26Mg= 0

%XY= 91

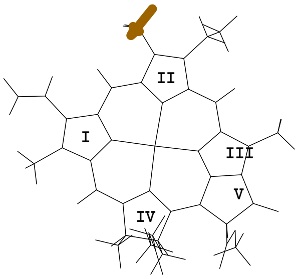

ν= 3017
λ= 0
15N= 0
26Mg= 0

%XY= 92

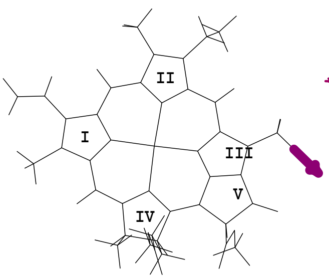

ν= 3020
λ= 0
15N= 0
26Mg= 0

%XY= 81

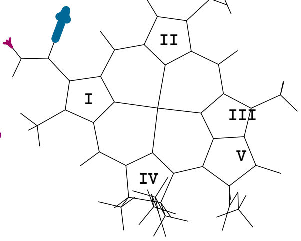

ν= 3021
λ= 0
15N= 0
26Mg= 0

%XY= 99

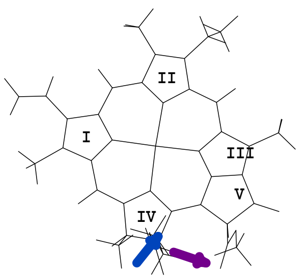

%S= 99

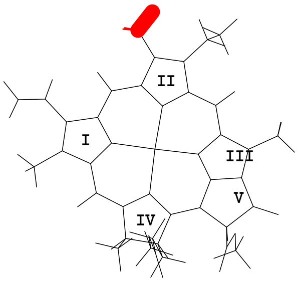

%S= 99

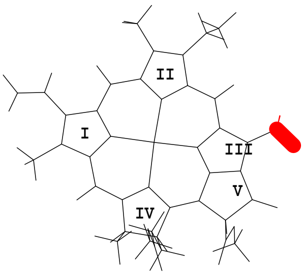

%S= 99

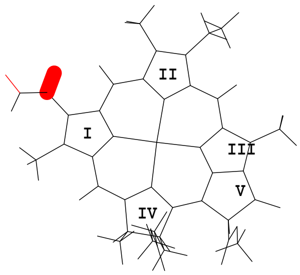

%S= 99

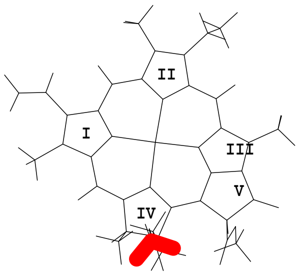

%B= 1

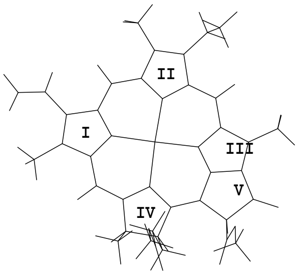

%B= 1

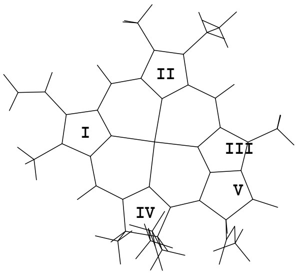

%B= 1

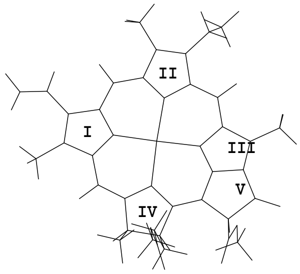

%B= 0

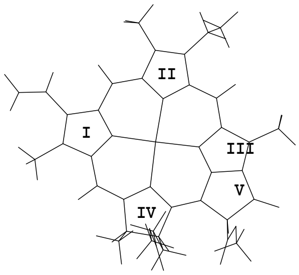

%T= 0

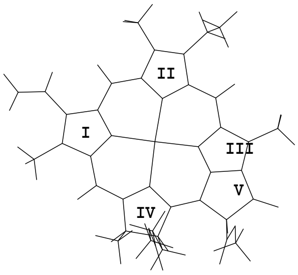

%T= 0

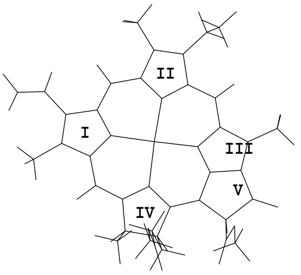

%T= 0

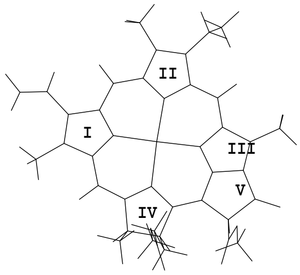

%T= 1

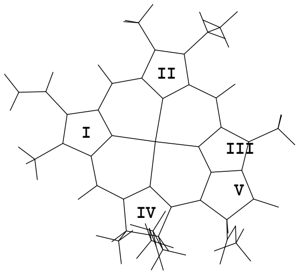

ν=	3024	3044	3050	3055
λ=	0	0	0	0
15N=	0	0	0	0
26Mg=	0	0	0	0

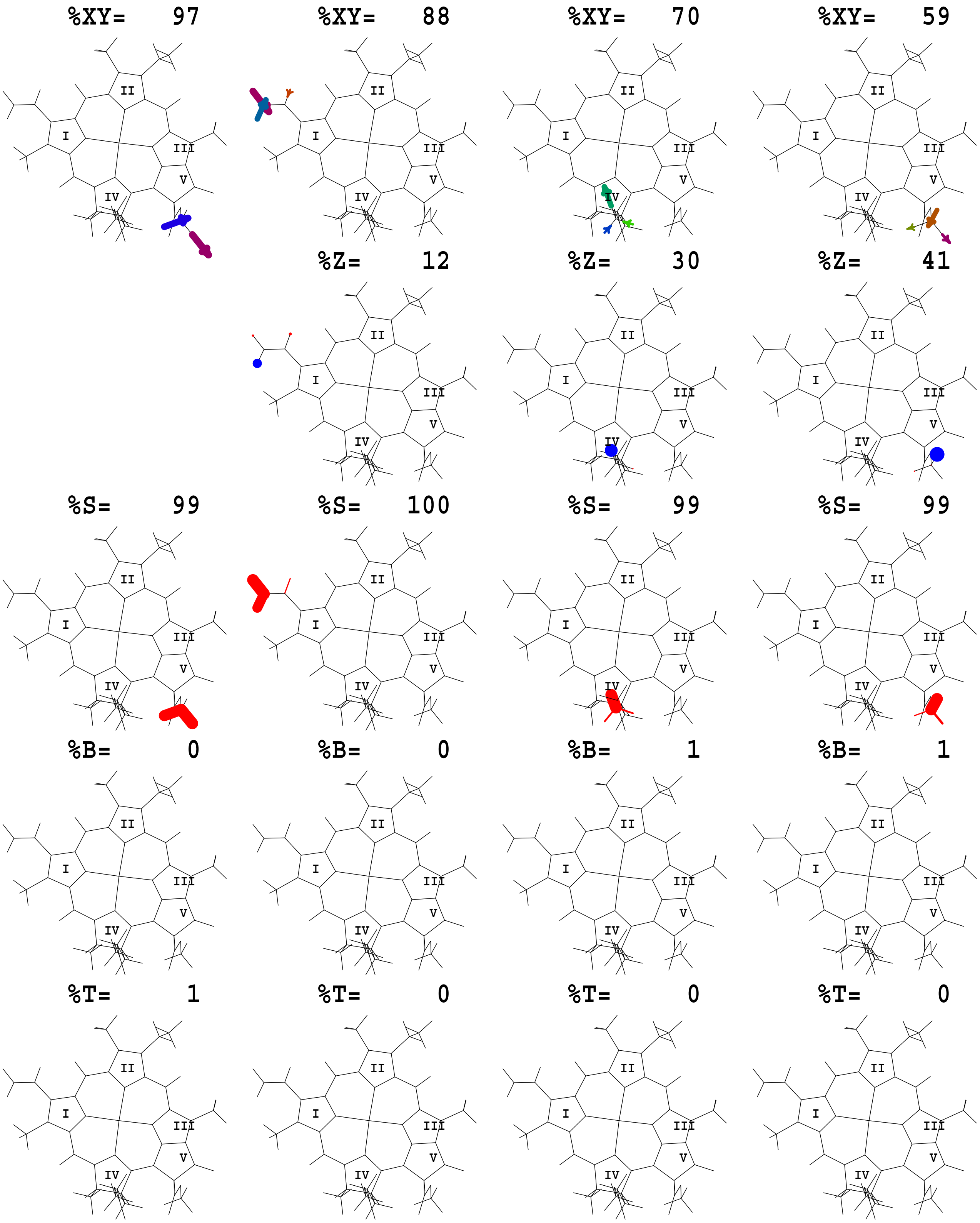

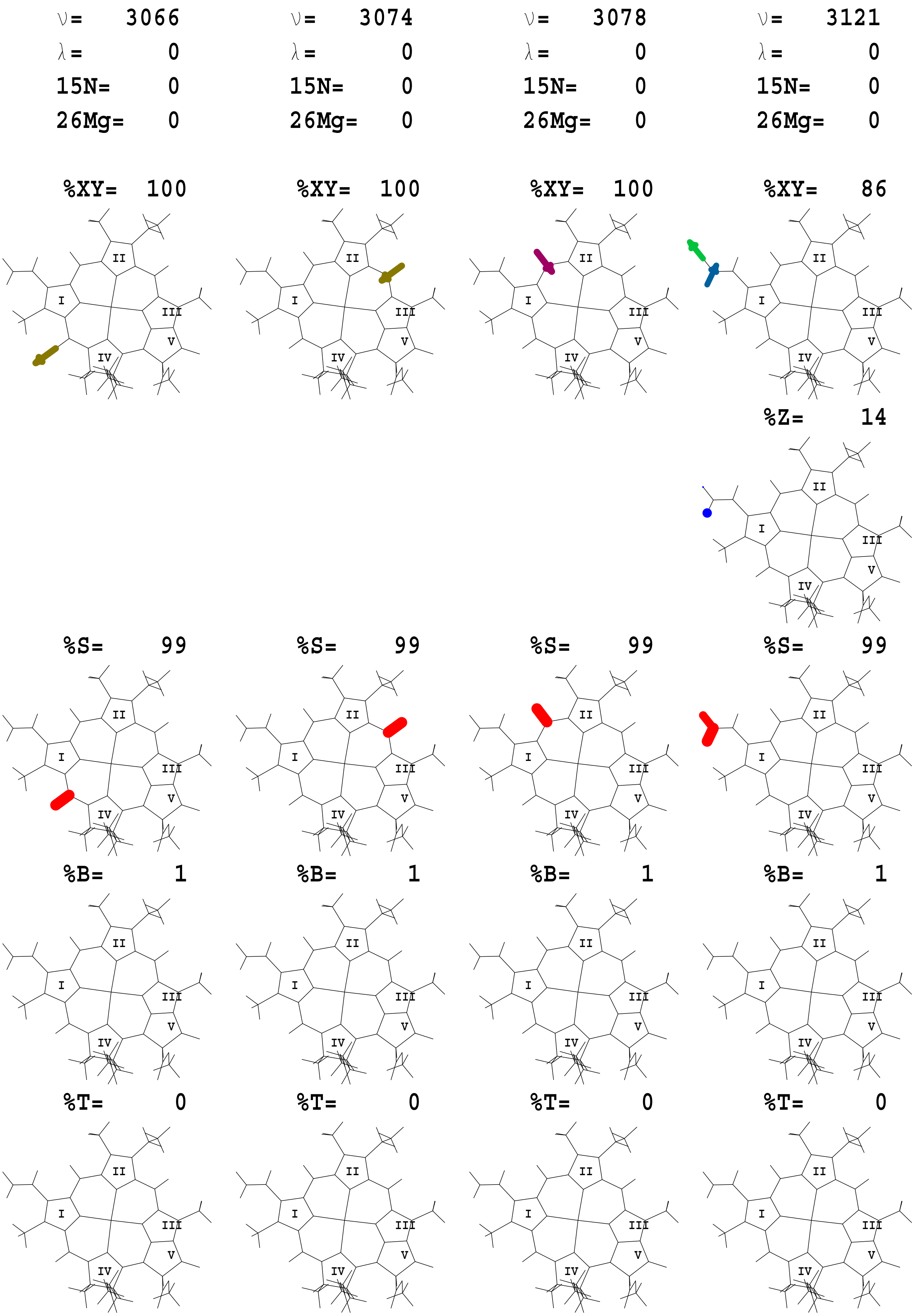
ν= 3066
λ= 0
15N= 0
26Mg= 0
%XY= 100
%S= 99
%B= 1
%T= 0
ν= 3074
λ= 0
15N= 0
26Mg= 0
%XY= 100
%S= 99
%B= 1
%T= 0
ν= 3078
λ= 0
15N= 0
26Mg= 0
%XY= 100
%S= 99
%B= 1
%T= 0
ν= 3121
λ= 0
15N= 0
26Mg= 0
%XY= 86
%Z= 14
%S= 99
%B= 1
%T= 0
I
II
III
IV
V
