# Supplementary material for: Asymmetry in the Qy Fluorescence and Absorption Spectra of Chlorophyll a Pertaining to Exciton Dynamics
Source: Front Chem. 2020 Dec 2;8:588289. doi: 10.3389/fchem.2020.588289 (PMC7738624; doi:10.3389/fchem.2020.588289)
Supplement: Supplementary file 3 [file Data_Sheet_3.pdf]

Supplementary Material

Non-orthogonal character of Duschinsky matrix in curvilinear coordinates.

The DUSHIN programme (Reimers, 2001) implements a basic scheme for determining the Duschinsky matrix **D** in curvilinear coordinates (curvilinear option “1”) that returns an orthonormal matrix. Unfortunately, this derivation is based on assumptions of alignment between the ground and excited states that typically only hold for small molecules. In addition, there is a general, approximate, scheme (curvilinear option “2”) implemented, which is what is applied herein. Two file names must be provided to DUSHIN containing the normal coordinates to be analysed. The Duschinsky matrix that results gives the mapping of the second state in terms of the first state. Hence to obtain the results presented in Fig. 8 etc, one must supply the Q_y file first and the S_0 file second.

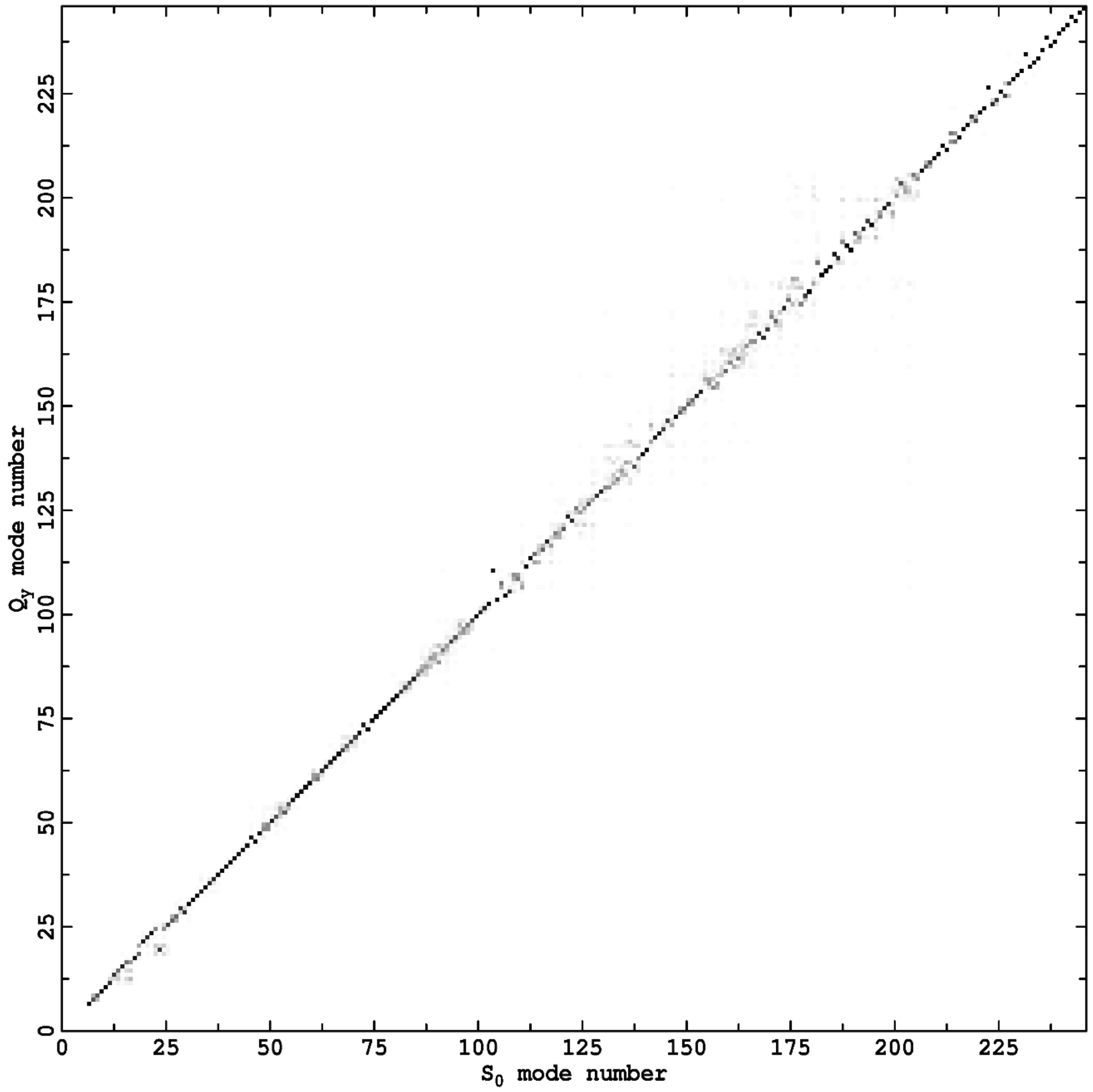

Supplementary Figure S1. The vibrational density D_{ik}^2 linking modes on the ground state to those on the excited state, in order of increasing frequency.

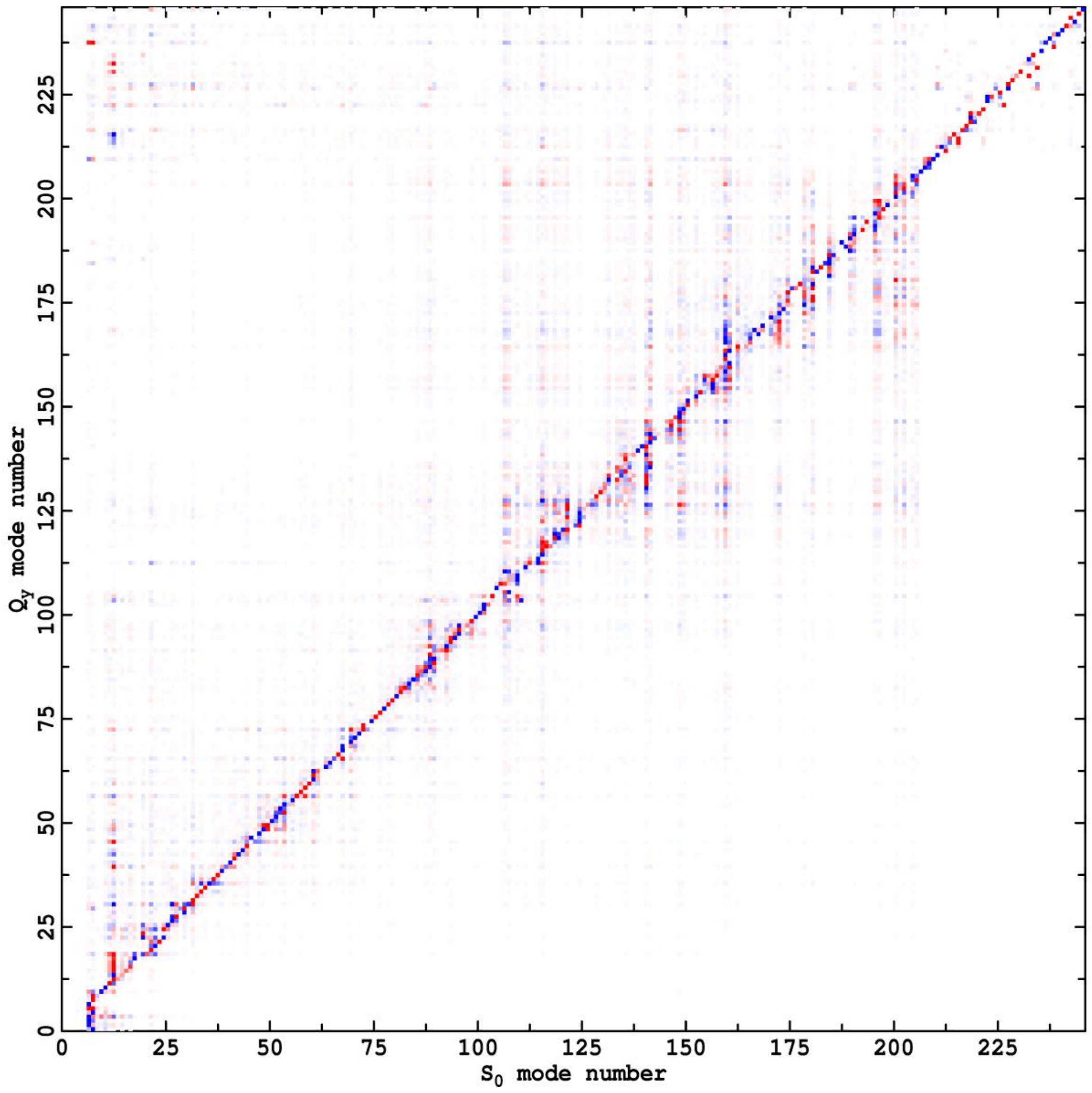

Supplementary Figure S2. Contributions to the displacement of each excited-state mode from each ground-state mode (Eqn. (13)): red- positive sign, blue- negative sign.

Supplementary Table S1. Variations of CAM-B3LYP/6-31G* reorganization energies from total energy differences, in cm^{-1}, for transitions between S_0 and Q_y with treatment of dispersion and solvent; some observed data from Table 3 is added for comparison.

Dispersion	explicit solvent	implicit solvent	λ^A	λ^E
none	none	none	607	517
D3	none	none	606	519
D3	1-propanol	none	598	491
D3	$(1\text{-propanol})_2$	none	589	509
D3	ether	none	628	630
D3	none	1-propanol	1795	934
D3	$(1\text{-propanol})_2$	1-propanol	1721	1006
D3	ether	ether	1254	787
observed 1-propanol low res. 5-coordinate			~490	492
observed 1-propanol low res. 6-coordinate			~420	~650
observed ether low res. 5-coordinate			264	438

Supplementary Table S2. Tentative relationships between observed Chl-a ^1Q_y vibrational modes (Reimers et al., 2013) from fluorescence excitation (Avarmaa and Rebane, 1985) in wet (Reimers et al., 2014) ether at 4.2 K and observed S_0 modes from ΔFLN in 1-propanol or TEA at 4.5 K, see Tables 1-2. These are obtained by assigning the observed lines to modes calculated for methyl Chl-a. 1-propanol using CAM-B3LYP, using the calculated Duschinsky matrix elements to map the calculated modes of ^1Q_y onto those of S_0.

^1Q_y FE wet ether		^1Q_y calc.		^1Q_y assignment in terms of S_0 modes	S_0 calc.		S_0 ΔFLN TEA		S_0 ΔFLN 1-propanol	
ν_i	λ_i	ν_i	λ_i		ν_i	λ_i	ν_i	λ_i	ν_i	λ_i
--	-						22	0.3	-	-
		63	11	73% 63, 20% 64	63	11				
110	1	95	7	53% 91, 41% 93	91	5	99	2	92[b]	4[b]
190	1	152	2	88% 153	153	3	170-210	1	194	3
263	2	263	2	91% 269	269	1	263	4	267	9
344	3	345	2	98% 348	348	2	349	6	353	11
370-390	3	378,384	4	96% 388, 69% 392, 24% 402	348,388,392	5	370-390	5	370-390	7
435-470	1	463	3	77% 470	470	3	430-450	1	430-450	5
		500	2	68% 504	504	4				
515	0.3	505	3	67% 512	512	0.3	520	4	519	7
563	3	591	3	99% 595	595	3	570	2	573	7
739	11	736	15	75% 740	740	15	742	12	745	36
788	3	840	3	89% 843	843	2	798	3	798	9
925	3	918	7	50% 894, 15% 957	894,908	15	915	13	914	25
966	23	979	25	82% 977	977	39	986	25	985	31
1034	6						1046	7	1043	25[c]
1070	8	1078	7	12% 1059, 35% 1073	1073	0.2	1071	5	1064	18[c]
1107	4	1110	1	42% 1102, 22% 1117, 12% 1137	1102,1117	7	1109	5	1107	13[c]
1132	5	1133	15	54% 1132, 20% 1152	1132	7	1117	5	1120	18
					1152[a]	15	1144	18	1146	42
1165	8	1163	13	37% 1171, 33% 1185	1171	13	1183	18	1183	55
1196	5	1192	5	39% 1185, 16% 1152	1185	1	1209	17		
		1210	2	25% 1219, 72% 1212	1212	23	1236	9		
1228	15	1223	45	20% 1212, 69% 1219	1219	32	1223	17	1224	69
1253	18	1260	14	91% 1262	1262	10	1263	8	1261	10
1286	16	1289	13	39% 1285, 25% 1292	1282,1285,1292	8	1288	7	1288	18
		1307	7	23% 1292, 17% 1310, 13% 1320	1292	3				
		1324	6	39% 1323, 21% 1339	1323	6	1306	4	1306	24
		1328	11	27% 1330, 18% 1339, 25% 1348	1320	43	1329	23	1324	39
1332	18	1332	14	60% 1330	1330	0				
					1339[a]	3	1354	4	1352	12
1369	10	1365	3	53% 1367, 15% 1390	1367	2	1374	2	1374	10
1393	7	1392	8	16% 1380, 10% 1390, 11% 1397	1390,1397	7	1390	6	1388	12
		1423	4	24% 1423, 19% 1460	1423	4				
1415	6	1426	11	33% 1423, 39% 1439	1439	5	1435	15	1436	23
					1460[a]	5	1467	1	1488	6
1446	8	1467	3	68% 1470	1470	2				
1510[d]	18	1505	12	45% 1488,10% 1502	1488	15	1519	5	1517	18
1530	9	1535	54	32% 1542, 23% 1565, 20% 1585	1542	24	1537	22	1531	18
1587	11	1579	57	15% 1565, 18% 1566, 17% 1624	1565,1566	12	1552	15	1552	17
		1601	30	20% 1594, 54% 1624	1624	6	1686	6	1654	10
1665	4	1612	4	54% 1594, 22% 1624	1594	8	1610	4	1596	2
	188		428	Total, listed modes		370		301		500
	262		549	Total, all modes		469		370		650

[a]: no clear assignment, distributed over many Q_y modes.

[b]: it is unclear as to whether all or part of this emission should be attributed to intramolecular vibrations, as reported in this table and elsewhere, or else to intermolecular phonons; modes of lower frequency are not easily identifiable in spectra.

[c]: no plausible assignment.

[d]: broad band, originally listed at 1493 cm^{-1} but the peak in this region is at 1510 cm^{-1}.

Supplementary Table S3. Comparison of absorption line frequencies and reorganization energies, both in cm^{-1}, from both calculation (after rescaling of the force constants) and experiment; lines with large intensity changes are highlighted in red.

S_0 calc. gas phase		S_0 calc. 1-propanol		S_0 ΔFLN TEA		S_0 ΔFLN 1-propanol	
ν_i	λ_i	ν_i	λ_i	ν_i	λ_i	ν_i	λ_i
				22	0.3	-	-
41, 64	12	63	11				
93,99	7	91	5	99	2	92[b]	4[b]
154	5	153	3	170-210	1	194	3
244,267	3	269	1	263	4	267	9
345	2	348	2	349	6	353	11
388,391	3	348,388,392	5	370-390	5	370-390	7
470	2	470	3	430-450	1	430-450	5
501	3	504	4				
513	1	512	0.3	520	4	519	7
593	2	595	3	570	2	573	7
737,739	16	740	15	742	12	745	36
839	2	843	2	798	3	798	9
895,897	15	894,908	15	915	13	914	25
977	40	977	39	986	25	985	31
				1046	7	1043	25[c]
1093	1	1073	0.2	1071	5	1064	18[c]
1102,1122	3	1102,1117	7	1109	5	1107	13[c]
1131	10	1132	7	1117	5	1120	18
1149[a]	12	1152[a]	15	1144	18	1146	42
1167	12	1171	13	1183	18	1183	55
1192	5	1185	1	1209	17		
1211	26	1212	23	1236	9		
1221	35	1219	32	1223	17	1224	69
1262	11	1262	10	1263	8	1261	10
1286,1287,1288	9	1282,1285,1292	8	1288	7	1288	18
1288[a]	3	1292	3				
1324	17	1323	6	1306	4	1306	24
1321[a]	37	1320	43	1329	23	1324	39
1331	0	1330	0				
1338[a]	4	1339[a]	3	1354	4	1352	12
1366	2	1367	2	1374	2	1374	10
1396	5	1390,1397	7	1390	6	1388	12
		1423	4				
1422	5	1439	5				
1441	5	1460[a]	5	1435	15	1436	23
1458	5	1470	2	1467	1	1488	6
1470	0	1488	15				
1481,1486	24	1542	24	1519	5	1517	18
1538	26	1565,1566	12	1537	22	1531	18
1568	11	1624	6	1552	15	1552	17
1617	8	1594	8	1686	6	1654	10
1590	11		355	1610	4	1596	2
total in listed lines	355		370		301		500
total all lines	492		469		370		650

Supplementary Table S4. The observed ΔFLN spectrum of Chl-a in 1-propanol at 4.5 K is partitioned into blocks, showing the raw Huang-Rhys factors S_i and associated reorganization energies λ_i (in cm^{-1}) as a function of frequency ν_i (in cm^{-1}), the frequency range used in each block, the signal peak within each block ν_j (in cm^{-1}), and the reorganization energy summed over each block λ_j (in cm^{-1}).

ν_i	S_i	λ_i	λ_j (block sum)	ν_j (peak)	range
66.0	0.0016	0.10			
73.0	0.0043	0.32			
80.0	0.0047	0.37			
85.0	0.0037	0.32			
91.0	0.0057	0.52			
98.0	0.0049	0.48	3.79	92	66-151
105.0	0.0044	0.46			
112.0	0.0032	0.35			
119.0	0.0028	0.33			
126.0	0.0016	0.20			
133.0	0.0011	0.14			
139.0	0.0005	0.08			
146.0	0.0004	0.05			
151.0	0.0005	0.08			
157.0	0.0003	0.05			
162.5	0.0016	0.26			
174.0	0.0021	0.37			
180.0	0.0012	0.21			
188.0	0.0024	0.45	3.20	194	157-210
194.0	0.0045	0.87			
202.0	0.0026	0.53			
210.0	0.0023	0.47			
218.0	0.0012	0.25			
232.0	0.0008	0.18			
245.0	0.0015	0.37			
253.5	0.0028	0.70			
259.5	0.0042	1.08			
265.0	0.0044	1.15	8.84	267	218-315
270.0	0.0039	1.05			
275.0	0.0029	0.79			
281.0	0.0028	0.79			
287.0	0.0022	0.62			
294.0	0.0021	0.62			
301.0	0.0018	0.54			
308.5	0.0012	0.38			
315.0	0.0010	0.33			
322.5	0.0012	0.40			
332.0	0.0013	0.42			
339.0	0.0028	0.94			
346.0	0.0057	1.98			

353.2	0.0115	4.07	10.80	353	322-366
359.5	0.0062	2.21			
366.0	0.0021	0.78			
372.0	0.0029	1.08			
379.0	0.0043	1.62	2.70	380	372-379
385.0	0.0042	1.60	4.35	392	385-392
392.0	0.0070	2.75			
400.0	0.0023	0.90			
407.5	0.0030	1.22	2.86	408	400-414
414.0	0.0018	0.75			
422.0	0.0008	0.33			
428.0	0.0014	0.61	2.57	434	422-435
434.5	0.0037	1.62			
442.0	0.0019	0.83			
448.0	0.0022	0.99	2.31	448	442-456
455.5	0.0011	0.50			
463.5	0.0007	0.34			
472.0	0.0022	1.05	1.58	472	463-479
479.0	0.0004	0.19			
487.0	0.0009	0.42			
494.0	0.0028	1.39	2.96	495	487-502
501.5	0.0023	1.15			
510.0	0.0012	0.63			
519.0	0.0087	4.51			
526.0	0.0024	1.26	7.40	519	510-541
533.0	0.0011	0.58			
541.0	0.0008	0.43			
549.5	0.0015	0.81	1.23	549	549-560
560.0	0.0007	0.41			
572.5	0.0096	5.48	6.76	573	573-584
584.0	0.0022	1.28			
591.5	0.0025	1.45	1.45	591	592
599.0	0.0009	0.53			
605.5	0.0020	1.19	2.60	606	599-613
613.0	0.0014	0.88			
620.5	0.0005	0.32			
628.5	0.0009	0.57			
639.0	0.0005	0.29			
648.0	0.0004	0.24			
658.0	0.0005	0.34	3.78	664	620-697
669.0	0.0007	0.47			
677.0	0.0004	0.24			
687.0	0.0009	0.59			
697.0	0.0010	0.72			
703.0	0.0022	1.55	1.55	703	703
712.0	0.0019	1.36			
720.0	0.0027	1.94			
729.0	0.0044	3.19			

735.0	0.0037	2.71			
742.0	0.0185	13.72	35.47	743	712-755
749.0	0.0120	8.96			
755.0	0.0048	3.59			
763.0	0.0033	2.51			
769.0	0.0023	1.75	6.10	763	763-775
775.0	0.0024	1.84			
782.0	0.0018	1.38			
789.0	0.0024	1.86			
798.0	0.0054	4.30	8.94	798	782-805
804.5	0.0017	1.40			
811.5	0.0013	1.08			
819.0	0.0006	0.48			
825.0	0.0011	0.92			
831.0	0.0007	0.57			
838.5	0.0015	1.25	7.06	838	810-882
847.0	0.0012	1.02			
855.0	0.0005	0.45			
861.0	0.0002	0.19			
871.0	0.0006	0.48			
882.0	0.0007	0.62			
903.0	0.0010	0.87			
896.5	0.0022	1.97			
906.5	0.0021	1.94			
913.5	0.0139	12.65	25.10	914	903-933
918.8	0.0037	3.40			
925.0	0.0036	3.34			
933.0	0.0010	0.93			
942.5	0.0019	1.81			
951.0	0.0008	0.78	2.59	942	942-951
958.0	0.0016	1.54			
965.0	0.0020	1.88			
973.0	0.0028	2.72			
984.0	0.0126	12.39	31.22	985	958-1008
989.0	0.0028	2.81			
995.0	0.0060	5.94			
1000.5	0.0028	2.75			
1008.0	0.0012	1.18			
1014.5	0.0030	3.01	3.01	1016	1015
1023.0	0.0025	2.56			
1030.5	0.0031	3.22			
1037.0	0.0054	5.60	24.84	1043	1023-1049
1043.0	0.0072	7.54			
1048.5	0.0057	5.92			
1055.0	0.0033	3.50			
1062.0	0.0026	2.78			
1067.5	0.0047	4.96	17.51	1064	1055-1080
1074.0	0.0042	4.52			

1080.0	0.0016	1.74			
1086.0	0.0011	1.22			
1092.0	0.0016	1.79			
1100.0	0.0032	3.55			
1107.0	0.0055	6.12	12.68	1107	1086-1107
1113.0	0.0012	1.32			
1119.5	0.0097	10.90	18.31	1120	1113-1135
1127.0	0.0026	2.92			
1135.0	0.0028	3.17			
1145.5	0.0192	21.94	41.58	1146	1145-1167
1153.5	0.0074	8.51			
1161.0	0.0048	5.56			
1167.0	0.0048	5.57			
1174.0	0.0051	6.00			
1180.0	0.0168	19.84			
1185.5	0.0201	23.86	55.38	1183	1174-1192
1192.0	0.0048	5.69			
1198.0	0.0043	5.20			
1206.5	0.0062	7.53			
1212.5	0.0076	9.17			
1218.5	0.0086	10.52			
1224.5	0.0135	16.47	68.46	1224	1198-1250
1231.5	0.0048	5.90			
1237.0	0.0037	4.61			
1243.0	0.0033	4.10			
1250.0	0.0040	4.96			
1257.5	0.0033	4.15			
1264.0	0.0033	4.20	9.88	1261	1258-1271
1271.0	0.0012	1.54			
1277.0	0.0027	3.45			
1282.5	0.0022	2.85			
1288.5	0.0087	11.16	17.45	1288	1277-1289
1296.0	0.0032	4.20			
1304.0	0.0094	12.23	23.60	1306	1296-1311
1311.0	0.0055	7.17			
1317.0	0.0018	2.32			
1324.0	0.0224	29.70	39.08	1324	1317-1339
1332.0	0.0033	4.41			
1339.0	0.0020	2.65			
1347.0	0.0035	4.69			
1353.0	0.0038	5.18	11.47	1352	1347-1359
1359.0	0.0012	1.60			
1368.0	0.0027	3.69			
1375.0	0.0021	2.85	10.10	1374	1368-1381
1381.0	0.0026	3.56			
1388.0	0.0071	9.81	11.98	1388	1388-1395
1395.0	0.0016	2.16			
1402.5	0.0028	3.96	3.96	1402	1403

1414.0	0.0009	1.27			
1425.0	0.0023	3.33			
1433.0	0.0031	4.46			
1438.0	0.0034	4.95	22.58	1436	1414-1465
1444.5	0.0020	2.86			
1451.5	0.0020	2.83			
1458.5	0.0013	1.93			
1465.0	0.0007	0.95			
1471.0	0.0005	0.66			
1481.0	0.0011	1.66			
1488.0	0.0015	2.25	5.58	1488	1471-1495
1495.0	0.0007	1.02			
1502.0	0.0004	0.53			
1506.5	0.0006	0.86			
1511.5	0.0028	4.20			
1516.5	0.0047	7.10			
1521.0	0.0037	5.58	18.27	1517.0	1502-1521
1525.0	0.0003	0.44			
1530.0	0.0075	11.46	17.52	1531	1525-1536
1535.5	0.0037	5.62			
1542.0	0.0016	2.48			
1549.0	0.0028	4.26			
1554.0	0.0036	5.58	17.02	1552	1542-1585
1561.0	0.0017	2.65			
1568.0	0.0005	0.78			
1575.0	0.0006	0.96			
1585.0	0.0002	0.30			
1596.0	0.0012	1.93	1.93	1596	1596
1647.5	0.0018	2.98			
1654.0	0.0022	3.67	10.41	1654	1648-1661
1661.0	0.0023	3.75			
**Total**	0.7132	649.82	649.82		

Supplementary Table S5. The observed ΔFLN spectrum of Chl-a in TEA at 4.5 K is partitioned into blocks, showing the raw Huang-Rhys factors S_i and associated reorganization energies λ_i (in cm^{-1}) as a function of frequency ν_i (in cm^{-1}), the frequency range used in each block, the signal peak within each block ν_j, and the reorganization energy summed over each block (all in cm^{-1}).

ν_i	S_i	λ_i	λ_j (block sum)	ν_j (peak)	range
21.5	10.1	0.2	0.3	22	10-41
27.88	2.3	0.1			
34.25	0	0			
40.62	0	0			
47	0	0			
53.38	0.5	0			
59.75	1.9	0.1			
66.12	3.7	0.2	1.3	66	41-87
72.5	3.3	0.2			
77	3	0.2			
81.5	3.4	0.3			
86	4.5	0.4			
92.25	4.5	0.4			
98.5	4.9	0.5	2	99	87-115
104.58	4	0.4			
110.67	3.3	0.4			
116.75	2.1	0.2			
122.83	1.4	0.2	0.5	123	115-155
128.92	0.4	0.1			
135	0	0			
139	0.4	0.1			
144.5	0.2	0			
149	0.5	0.1			
155.17	0	0			
161.33	0.4	0.1			
167.5	0.6	0.1			
173.5	1	0.2	0.6	174	155-190
179.5	0.3	0.1			
185.5	0.7	0.1			
192	0.5	0.1			
195	0.6	0.1			
200.25	0.5	0.1			
205.5	0.7	0.1	0.5	206	190-220
212	0.5	0.1			
218.5	0	0			
225	0	0			
231.5	0	0			
237.7	0.2	0.1			
243.9	1	0.2			
250.1	2.2	0.5			
256.3	3.5	0.9			
262.5	3.7	1	3.8	263	220-280
267.8	2.5	0.7			
273.1	1.3	0.4			
278.4	0.6	0.2			
283.7	0.8	0.2			
289	1.6	0.4	1.3	289	280-312
294.75	0.7	0.2			
300.5	0.4	0.1			
306.25	0.5	0.2			
312	0.2	0.1			
317.75	0.1	0			

323.5	0.1	0			
329.88	0.4	0.1	0.4	330	312-335
336.25	1	0.3			
342.62	4	1.4			
349	8.4	2.9	6	349	335-362
355.38	3.5	1.2			
361.75	1.9	0.7			
368.12	2.3	0.8			
374.5	2.9	1.1	2.6	375	362-380
380.25	2	0.8			
386	2.9	1.1	2.6	386	380-412
392.5	1.5	0.6			
399	0.8	0.3			
405.5	0.3	0.1			
412	0	0			
418.5	0.2	0.1			
425	1.2	0.5	0.7	425	412-432
430.67	0.6	0.3			
436.33	0.6	0.2	0.5	436	432-458
442	0.2	0.1			
447.67	0	0			
453.33	0	0			
459	0	0			
464.75	0.6	0.3			
470.5	1.4	0.6	1.1	471	458-477
476.5	0.6	0.3			
482.5	0.6	0.3			
488.5	1.1	0.5	1.5	489	477-500
494.7	0.7	0.3			
500.9	0.7	0.4			
507.1	0.3	0.2			
513.3	1.4	0.7			
519.5	3.7	1.9	3.6	520	500-530
525.75	1.3	0.7			
532	0	0			
538.25	0.3	0.2			
544.5	0.8	0.4	0.8	545	530-558
550.88	0.3	0.2			
557.25	0.2	0.1			
563.62	0.8	0.5			
570	3.3	1.9	2.4	570	558-575
575.5	0.1	0.1			
581	0.4	0.2			
586.5	0.4	0.2	0.5	587	575-593
593	0	0			
599.5	0.4	0.2			
606	1	0.6	0.9	606	593-612
611.5	0.3	0.2			
617	0	0			
622.5	0	0			
628	0.4	0.3	0.4	628	612-632
632.5	0.1	0.1			
637	0.1	0.1			
641.5	0.4	0.3	0.4	642	632-648
646.75	0.1	0.1			
652	0.1	0			
657	0.1	0.1			
662	0.1	0.1			
667.7	0.1	0.1			
673.4	0.2	0.1			

679.1	0.7	0.5			
684.8	0.7	0.5			
690.5	1.3	0.9	2.4	691	648-695
696	0.5	0.3			
701.5	1.6	1.1	2.3	702	695-718
707.25	0.4	0.3			
713	0.6	0.4			
718.75	0.9	0.6			
724.5	1.4	1	1.6	725	718-730
730.17	0.9	0.6			
735.83	3.5	2.6			
741.5	10.8	8	12	742	730-745
746.75	3	2.3			
752	5.8	4.4	6.4	752	745-756
757	2.3	1.8			
762	0.9	0.7			
767	1.8	1.4	3.8	767	756-775
772	0.9	0.7			
777	0.6	0.5			
782	0.5	0.4			
787	0.8	0.6			
792.5	0.5	0.4			
798	0.9	0.7	2.6	798	775-803
804.5	0.7	0.5			
811	0.2	0.2	0.5	811	803-820
817.5	0.1	0.1			
824	0.1	0			
830.5	0.2	0.2			
835.5	0.2	0.1			
840.5	0.5	0.4			
844.75	0.3	0.2			
849	0.4	0.3			
853.5	0.6	0.5	2	854	820-860
859.7	0.4	0.3			
865.9	0.2	0.2			
872.1	0.1	0.1			
878.3	0.3	0.3			
884.5	0.7	0.6	1.5	885	860-890
890.5	0.5	0.4			
896.5	1.2	1			
902.5	1.6	1.4			
908.5	2.2	2			
914.5	5.1	4.6	12.9	915	890-930
918.5	0	0			
922.5	3.4	3.2			
928.25	0.9	0.8			
934	0.1	0.1			
939.75	0.2	0.2			
945.5	0.3	0.3	1.1	946	930-955
950	0.1	0.1			
954.5	0.1	0.1			
960.7	0.2	0.2			
966.9	0.7	0.7			
973.1	0.6	0.5			
979.3	4.2	4.1			
985.5	12.5	12.4	24.9	986	955-1018
992.07	3.8	3.8			
998.64	2.2	2.2			
1005.21	0.4	0.4			
1011.79	0.5	0.5			

1018.36	0.3	0.3			
1024.93	1.1	1.1			
1031.5	1.1	1.1	2.9	1032	1018-1036
1036.17	1	1			
1040.83	1.5	1.5			
1045.5	2.1	2.2	6.5	1046	1036-1060
1051.75	1.8	1.9			
1058	0.8	0.8			
1064.25	1	1			
1070.5	1.7	1.8	4.8	1071	1060-1090
1076.92	1	1.1			
1083.33	0.2	0.2			
1089.75	0.4	0.4			
1096.17	0.5	0.5			
1102.58	1.5	1.7			
1109	2.6	2.9	5.4	1109	1090-1113
1113	0.1	0.1			
1117	2.9	3.2	4.7	1117	1113-1127
1122.3	0.9	1			
1127.6	0.7	0.8			
1132.9	1.1	1.3			
1138.2	2.5	2.8			
1143.5	10.6	12.2	18.2	1144	1127-1150
1150.08	2.8	3.2			
1156.67	2.3	2.7	7.3	1157	1150-1170
1163.25	1.8	2.1			
1169.83	1.7	1.9			
1176.42	5.2	6.1			
1183	6.9	8.1	18	1183	1170-1192
1189.38	4.7	5.6			
1195.75	3.3	4			
1202.12	3.6	4.3			
1208.5	3.9	4.7	16.9	1209	1192-1213
1213.33	1.8	2.2			
1218.17	3.4	4.2			
1223	7.2	8.8	16.6	1223	1213-1229
1229.58	4.1	5			
1236.17	4.3	5.4	9.3	1236	1229-1242
1242.75	2.3	2.8			
1249.33	1	1.2			
1255.92	0.9	1.2			
1262.5	2.4	3	7.5	1263	1242-1272
1268.88	1.1	1.3			
1275.25	1.4	1.8	3.7	1275	1272-1283
1281.62	2	2.5			
1288	3	3.8	7.4	1288	1283-1301
1294	1.2	1.6			
1300	1.1	1.4			
1306	2	2.6	3.6	1306	1301-1311
1310.25	0.5	0.6			
1314.5	1.6	2.1	2.7	1315	1311-1320
1319.33	0.4	0.5			
1324.17	4.2	5.6			
1329	11.8	15.6	22.5	1329	1320-1335
1335.33	1.5	2.1			
1341.67	1.4	1.9	4.1	1342	1335-1347
1348	1.8	2.4			
1354.38	1.1	1.5	3.7	1354	1347-1367
1360.75	0.4	0.6			
1367.12	0.5	0.7			

1373.5	1.1	1.5	2.3	1374	1367-1378
1378.83	0.5	0.7			
1384.17	0.9	1.3			
1389.5	1.5	2.1	5.6	1390	1378-1419
1395.62	0.6	0.8			
1401.75	0.3	0.4			
1407.88	0	0			
1414	0.2	0.3			
1419.12	0.3	0.5			
1424.25	1	1.4			
1429.38	1.7	2.5			
1434.5	3.1	4.4	15.2	1435	1419-1460
1440	1.9	2.7			
1445.5	1.1	1.5			
1451	0.8	1.2			
1456.5	0.7	1			
1462	0.3	0.4			
1467	0.5	0.7	1.1	1467	1460-1472
1472.6	0.2	0.4			
1478.2	0.6	0.8	1.7	1478	1472-1485
1483.8	0.9	1.4			
1489.4	1.5	2.2			
1495	2.6	3.9	7.7	1495	1485-1502
1501	1.1	1.6			
1507	0.3	0.5	2.2	1507	1502-1513
1513	1.1	1.7			
1519	1.1	1.6	4.5	1519	1513-1525
1525	2.6	4			
1531	4.7	7.3			
1537	7	10.7	21.8	1537	1525-1542
1541.83	2.4	3.7			
1546.67	2.4	3.7			
1551.5	5.2	8.1	15.4	1552	1542-1557
1556.88	2.3	3.6			
1562.25	0.8	1.3	4.2	1562	1557-1579
1567.62	0.3	0.4			
1573	0.4	0.6			
1578.67	0.1	0.1			
1584.33	0.1	0.2			
1590	0.5	0.9	1.4	1590	1579-1597
1596.5	0.3	0.4			
1603	0.5	0.8			
1609.5	1.1	1.7	4.4	1610	1597-1634
1615.67	0.7	1.1			
1621.83	0.2	0.3			
1628	0.1	0.2			
1633	0	0			
1638	0	0			
1643.25	0	0			
1648.5	0	0	0.1	1649	1634-1667
1655	0	0			
1661.5	0	0			
1667.62	0.1	0.2			
1673.75	0.3	0.5			
1679.88	1	1.7			
1686	1.6	2.8	5.7	1686	1667-1693
1692.07	0.7	1.2			
1698.14	0.7	1.2	2	1698	1693-1705
1704.21	0.2	0.4			
1710.29	0.1	0.1	0.3	1710	1705-1723

1716.36	0	0
1722.43	0	0
1728.5	0	0
1735	0.1	0.1

Supplementary Table S6. Taken from (Reimers et al., 2013) Table S2, summarizing the *y*-polarized Franck-Condon allowed component of the ^1Q_y absorption spectrum of Chl-a in wet ether at 4.2 K, from fluorescence-excitation data: peak frequencies ν_i and band frequency ranges (cm^{-1}), effective Huang-Rhys factors S_i, and associated conserved band reorganization energies $\lambda_i = h\nu_i S_i$ (cm^{-1}).

ν_i peak	S_i *1000	λ_i	ν min	ν max	ν_i peak	S_i *1000	λ_i	ν min	ν max
74.0	1.3	0.1	63.0	85.0	812.0	5.1	4.1	798.5	825.5
110.0	8.3	0.9	93.0	126.5	846.5	3.2	2.7	832.5	859.5
150.0	9.3	1.4	133.0	166.5	878.0	2.9	2.6	866.0	890.0
190.0	2.9	0.5	173.5	206.0	903.5	3.7	3.3	896.5	910.0
263.0	7.7	2.0	249.5	276.0	925.0	3.6	3.3	917.5	932.5
290.5	3.2	0.9	284.0	297.0	965.5	23.3	22.5	940.0	991.0
314.5	3.0	0.9	304.0	325.0	1008.0	7.7	7.7	998.0	1017.5
344.0	7.8	2.7	332.5	355.5	1034.0	5.7	5.9	1024.0	1045.0
370.0	4.2	1.5	363.0	376.5	1069.5	7.1	7.6	1051.0	1089.5
390.0	4.0	1.5	383.5	397.0	1106.5	3.5	3.9	1096.0	1116.5
410.5	2.1	0.9	403.5	417.5	1132.0	4.1	4.6	1122.5	1141.5
434.5	0.8	0.4	424.0	444.5	1165.0	7.0	8.1	1148.0	1181.5
465.0	0.3	0.2	465.0	465.0	1196.0	3.9	4.6	1188.5	1203.5
476.0	0.7	0.3	472.0	479.5	1228.0	11.4	14.5	1209.0	1544.0
493.5	0.5	0.3	486.5	500.5	1253.0	14.5	18.2	1242.0	1262.5
515.0	0.6	0.3	508.0	522.0	1286.0	12.0	15.5	1269.0	1303.5
535.5	0.6	0.3	529.0	542.5	1331.5	13.5	18.0	1310.5	1352.0
563.0	4.8	2.7	549.5	576.0	1369.0	7.1	9.7	1358.5	1379.5
586.0	2.7	1.6	581.0	591.0	1393.0	5.0	6.9	1385.0	1401.5
610.5	1.6	1.0	600.0	621.0	1415.0	4.1	5.8	1408.5	1422.0
639.0	1.5	1.0	628.0	649.0	1445.5	5.7	8.3	1428.5	1462.5
669.5	1.3	0.9	656.0	682.0	1493.0	11.7	17.5	1469.5	1516.5
703.0	4.0	2.8	688.0	717.5	1530.0	6.1	9.3	1523.5	1536.5
739.0	14.2	10.5	725.0	752.5	1586.5	6.9	11.0	1551.5	1622.0
770.0	6.2	4.8	760.0	778.0	1664.5	2.2	3.7	1629.0	1700.0
788.0	3.3	2.6	783.0	793.0	**total**	**278**	**262**		

References

Avarmaa R.A., Rebane K.K. (1985). High-resolution optical spectra of chlorophyll molecules. *Spectrochim. Acta A* 41, 1365–1380.

Reimers J.R. (2001). A practical method for the use of curvilinear coordinates in calculations of normal-mode projected displacements and Duschinsky rotation matrices for large molecules. *J. Chem. Phys.* 115, 9103-9109. doi:10.1063/1.1412875

Reimers J.R., Cai Z.-L., Kobayashi R., Rätsep M., Freiberg A., Krausz E. (2013). Assignment of the Q-bands of the chlorophylls: coherence loss via $Q_x - Q_y$ mixing. *Sci. Rep.* 3, 2761. doi: 10.1038/srep02761

Reimers J.R., Cai Z.-L., Kobayashi R., Rätsep M., Freiberg A., Krausz E. (2014). Formation of water-chlorophyll clusters in dilute samples of chlorophyll-a in ether at low temperature. *Phys. Chem. Chem. Phys.* 16, 2323-2330. doi: 10.1039/C3CP53729C
