# Supplementary material for: Asymmetry in the Qy Fluorescence and Absorption Spectra of Chlorophyll a Pertaining to Exciton Dynamics
Source: Front Chem. 2020 Dec 2;8:588289. doi: 10.3389/fchem.2020.588289 (PMC7738624; doi:10.3389/fchem.2020.588289)
Supplement: Supplementary file 4 [file Data_Sheet_4.pdf]

In this file, the forms of the normal modes are depicted graphically. For each mode is listed:

- Vibration frequency ν, in cm^{-1}.
- Reorganization energy in absorption, λ^A, in cm^{-1}.
- ^{15}N is the sensitivity of this vibration to N isotopic substitution. The isotopically substituted frequency differs by $\nu \times$ the shown sensitivity / 1000.
- ^{26}Mg is the sensitivity of this vibration to Mg isotopic substitution. The isotopically substituted frequency differs by $\nu \times$ the shown sensitivity / 1000.
- The percentage of the motion that is in the *xy* plane (ca. the macrocyclic plane).
- The percentage of the motion that is in the *z* direction (ca. normal to the macrocyclic plane).
- The percentages of the motion describable as bond stretches (S), bond-angle bends (B), and bond-angle torsions (T). Key motions are shown in different colours for clarity.

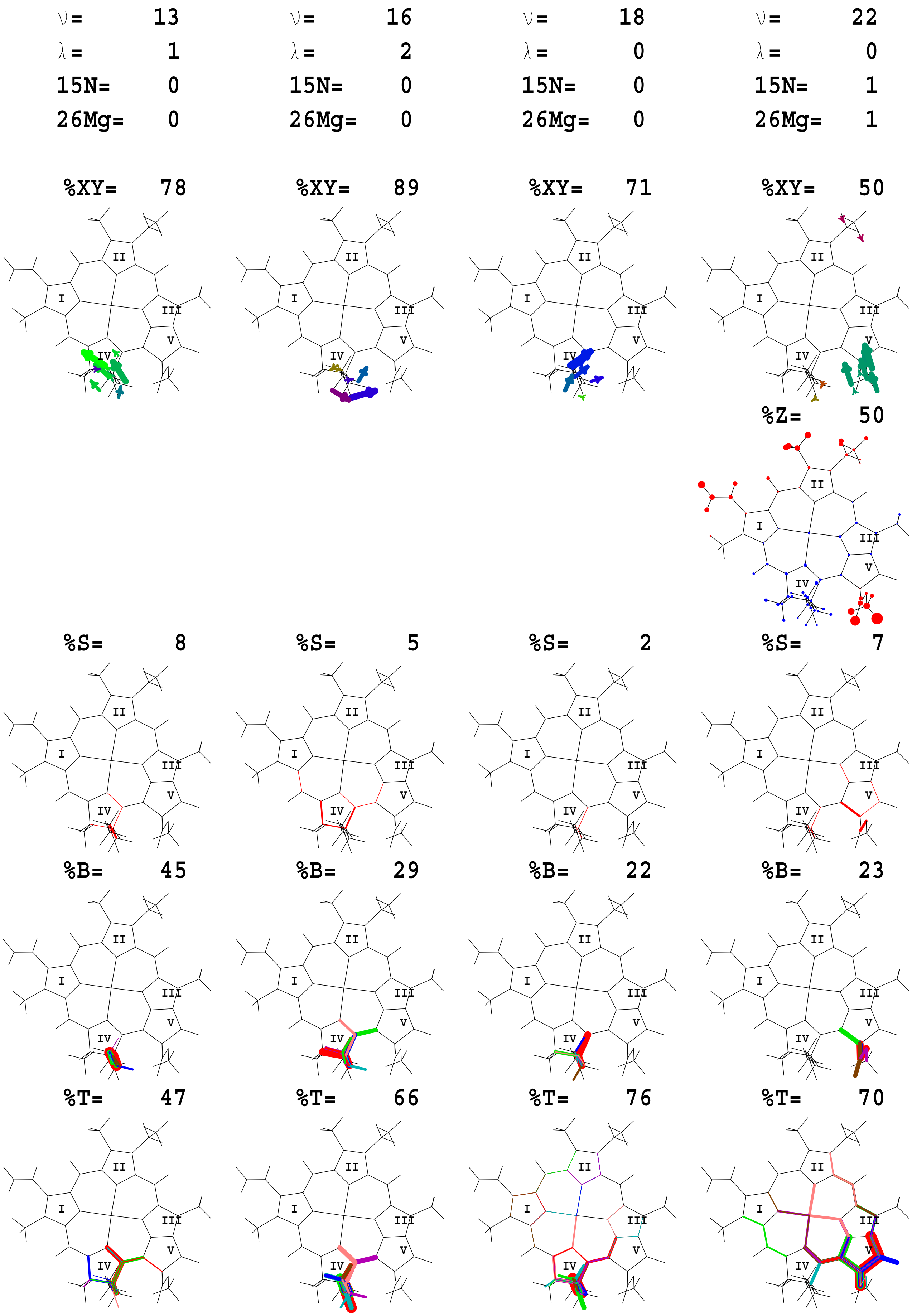

ν= 13
λ= 1
15N= 0
26Mg= 0
%XY= 78
%S= 8
%B= 45
%T= 47
ν= 16
λ= 2
15N= 0
26Mg= 0
%XY= 89
%S= 5
%B= 29
%T= 66
ν= 18
λ= 0
15N= 0
26Mg= 0
%XY= 71
%S= 2
%B= 22
%T= 76
ν= 22
λ= 0
15N= 1
26Mg= 1
%XY= 50
%Z= 50
%S= 7
%B= 23
%T= 70
I
II
III
IV
V

ν=	27	38	41	47
λ=	1	3	8	0
15N=	0	1	0	4
26Mg=	0	0	0	3
%XY=	43	65	74	35
%Z=	57	35	26	65
%S=	3	6	6	3
%B=	12	18	28	12
%T=	86	76	66	85

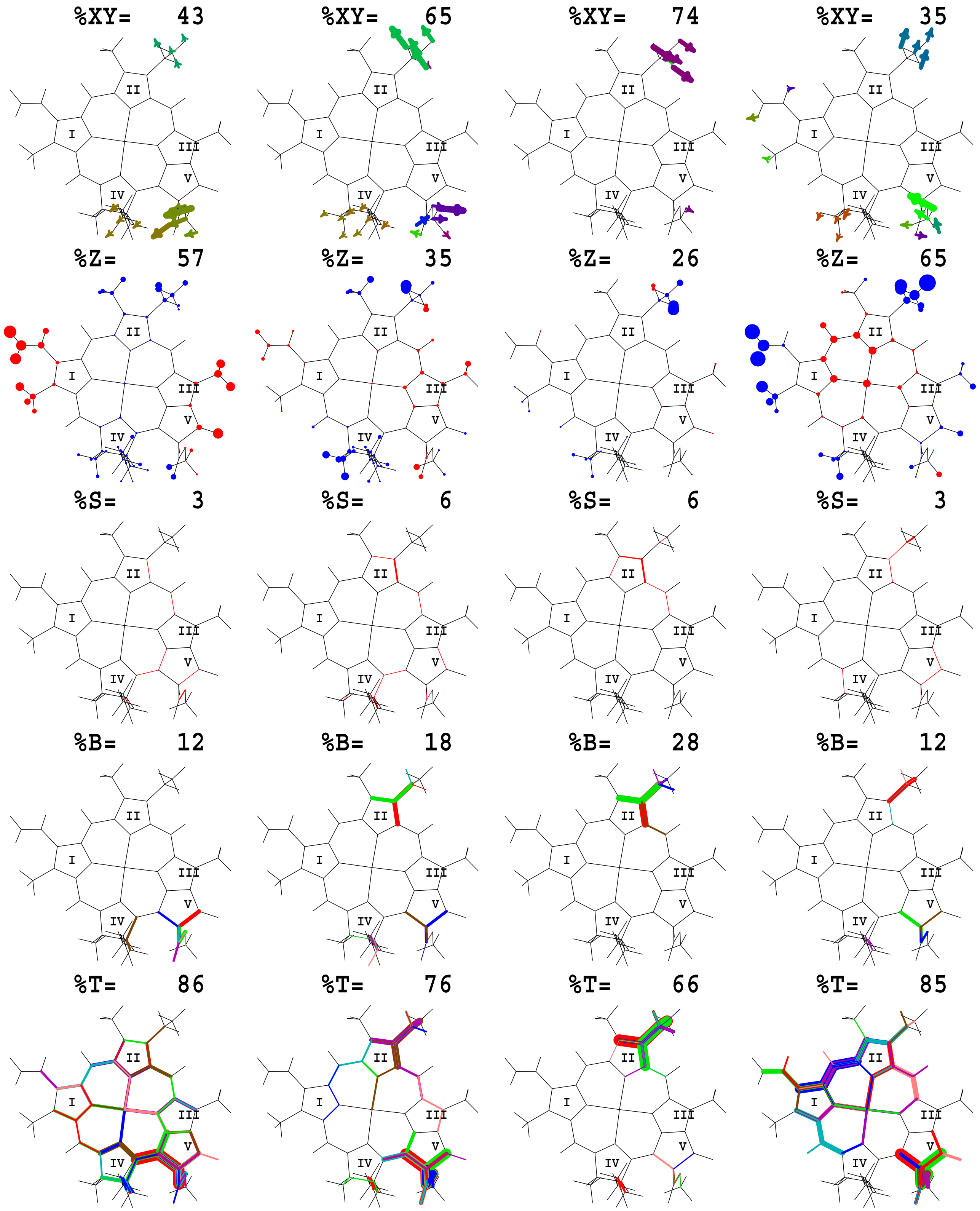

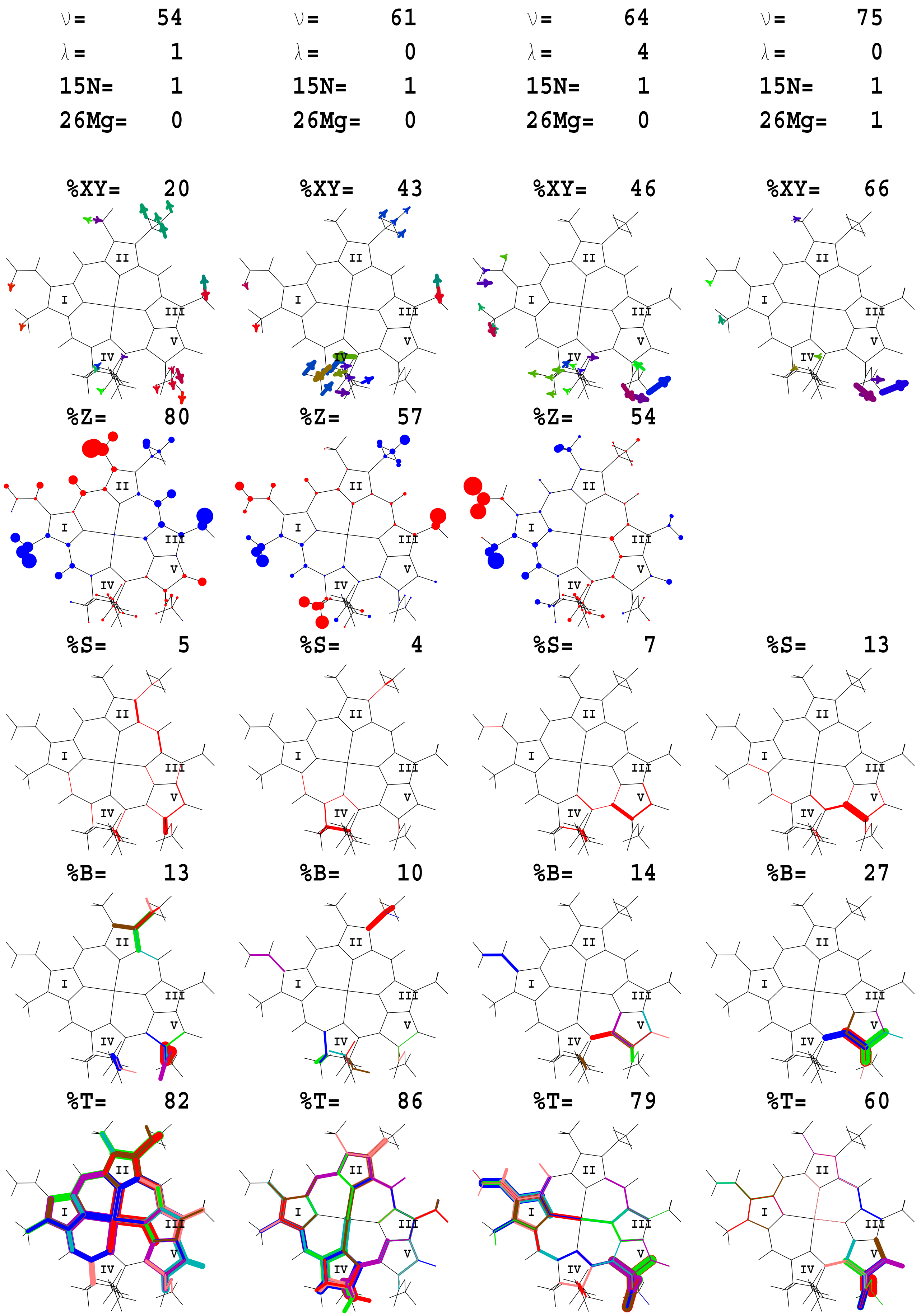

ν= 54
λ= 1
15N= 1
26Mg= 0
ν= 61
λ= 0
15N= 1
26Mg= 0
ν= 64
λ= 4
15N= 1
26Mg= 0
ν= 75
λ= 0
15N= 1
26Mg= 1
%XY= 20
%XY= 43
%XY= 46
%XY= 66
%Z= 80
%Z= 57
%Z= 54
%S= 5
%S= 4
%S= 7
%S= 13
%B= 13
%B= 10
%B= 14
%B= 27
%T= 82
%T= 86
%T= 79
%T= 60

ν=	78	89	90	93
λ=	0	1	0	6
15N=	1	0	1	1
26Mg=	1	0	1	0

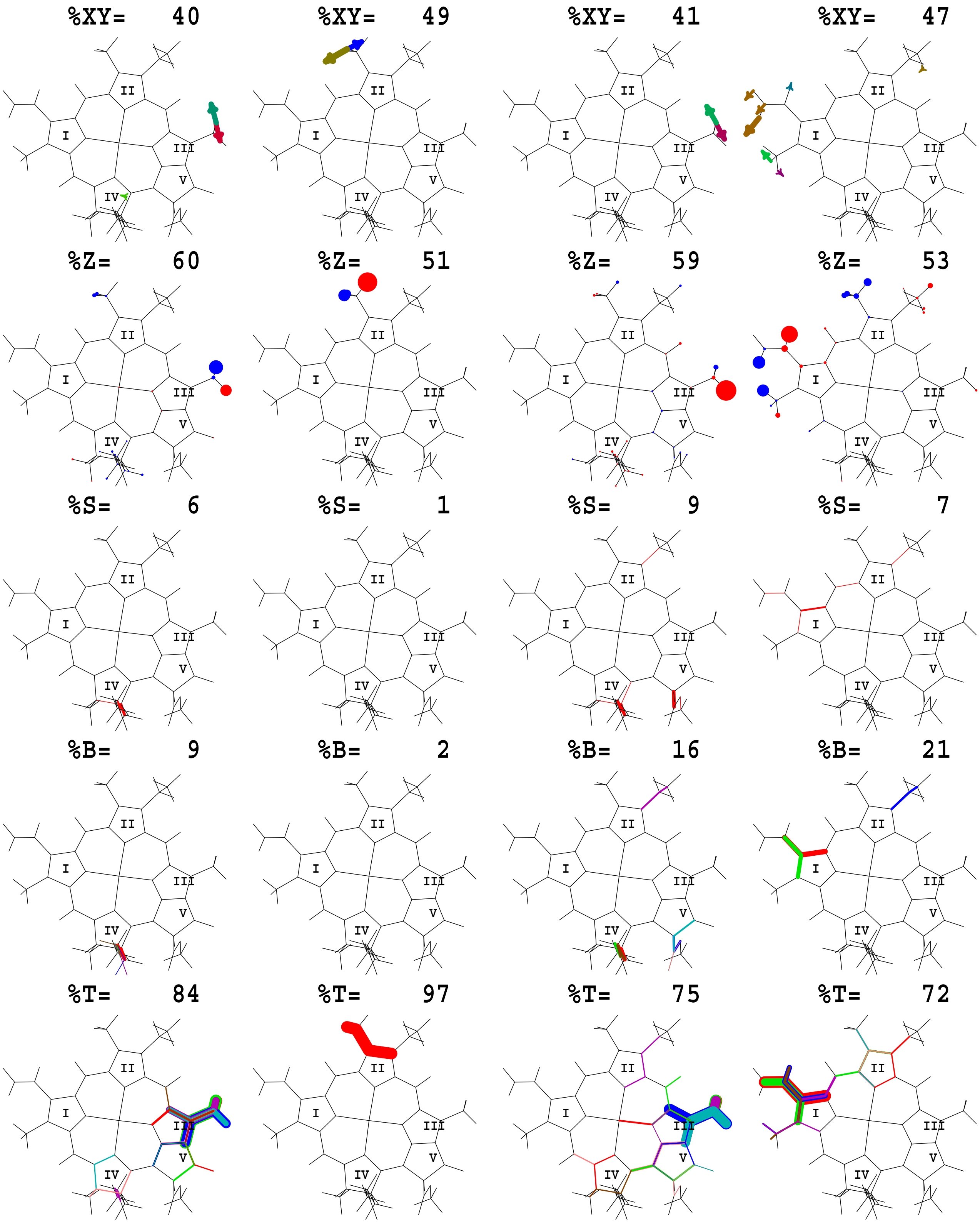

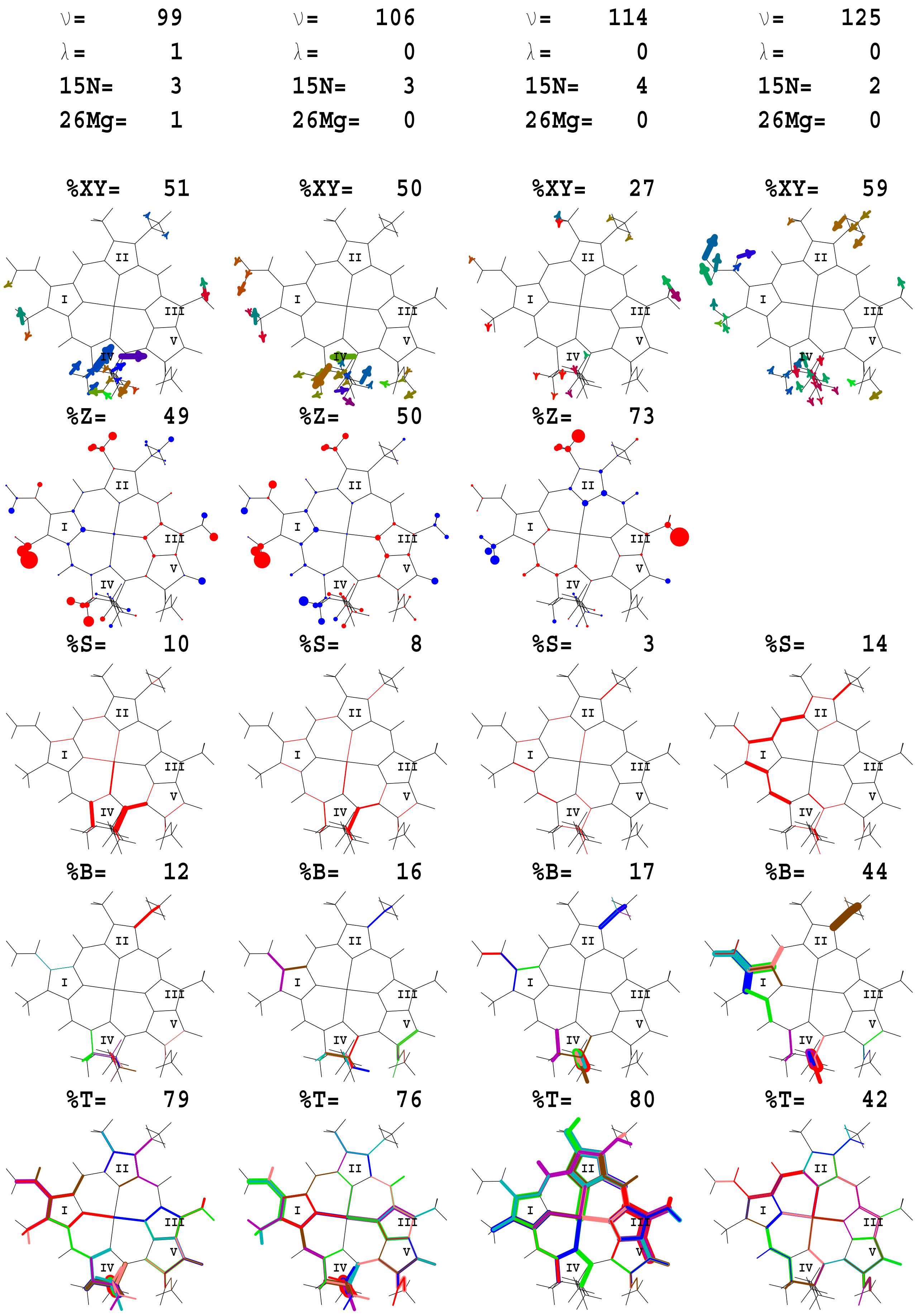
ν= 99
λ= 1
15N= 3
26Mg= 1
%XY= 51
%Z= 49
%S= 10
%B= 12
%T= 79
ν= 106
λ= 0
15N= 3
26Mg= 0
%XY= 50
%Z= 50
%S= 8
%B= 16
%T= 76
ν= 114
λ= 0
15N= 4
26Mg= 0
%XY= 27
%Z= 73
%S= 3
%B= 17
%T= 80
ν= 125
λ= 0
15N= 2
26Mg= 0
%XY= 59
%S= 14
%B= 44
%T= 42

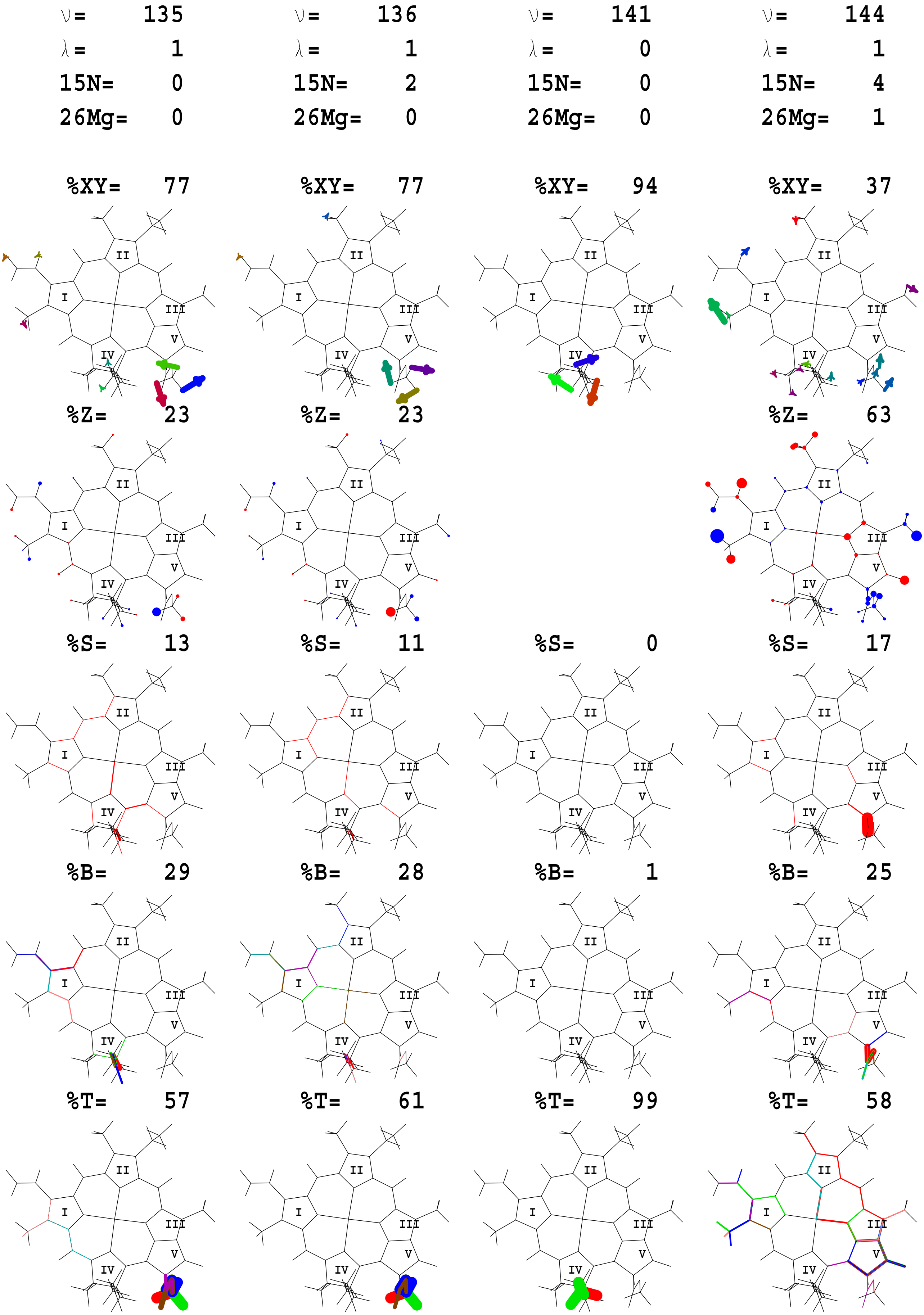

ν= 135
λ= 1
15N= 0
26Mg= 0
%XY= 77
%Z= 23
%S= 13
%B= 29
%T= 57
ν= 136
λ= 1
15N= 2
26Mg= 0
%XY= 77
%Z= 23
%S= 11
%B= 28
%T= 61
ν= 141
λ= 0
15N= 0
26Mg= 0
%XY= 94
%S= 0
%B= 1
%T= 99
ν= 144
λ= 1
15N= 4
26Mg= 1
%XY= 37
%Z= 63
%S= 17
%B= 25
%T= 58
I
II
III
IV
V

ν=	149	154	168	173
λ=	0	5	0	0
15N=	1	2	1	2
26Mg=	0	0	0	9

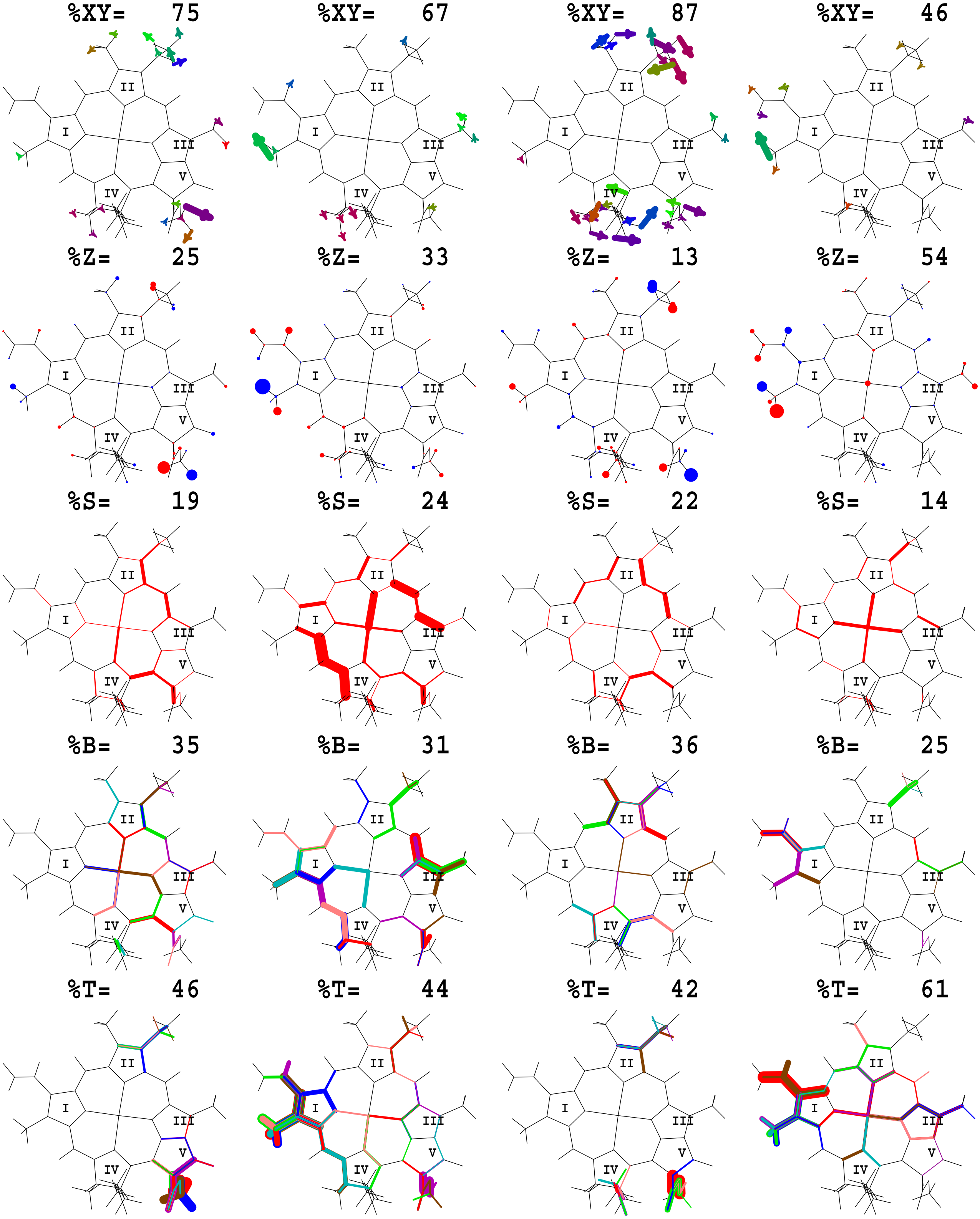

ν= 177	ν= 184	ν= 188	ν= 193
λ= 0	λ= 0	λ= 0	λ= 1
15N= 0	15N= 4	15N= 4	15N= 2
26Mg= 0	26Mg= 4	26Mg= 1	26Mg= 1

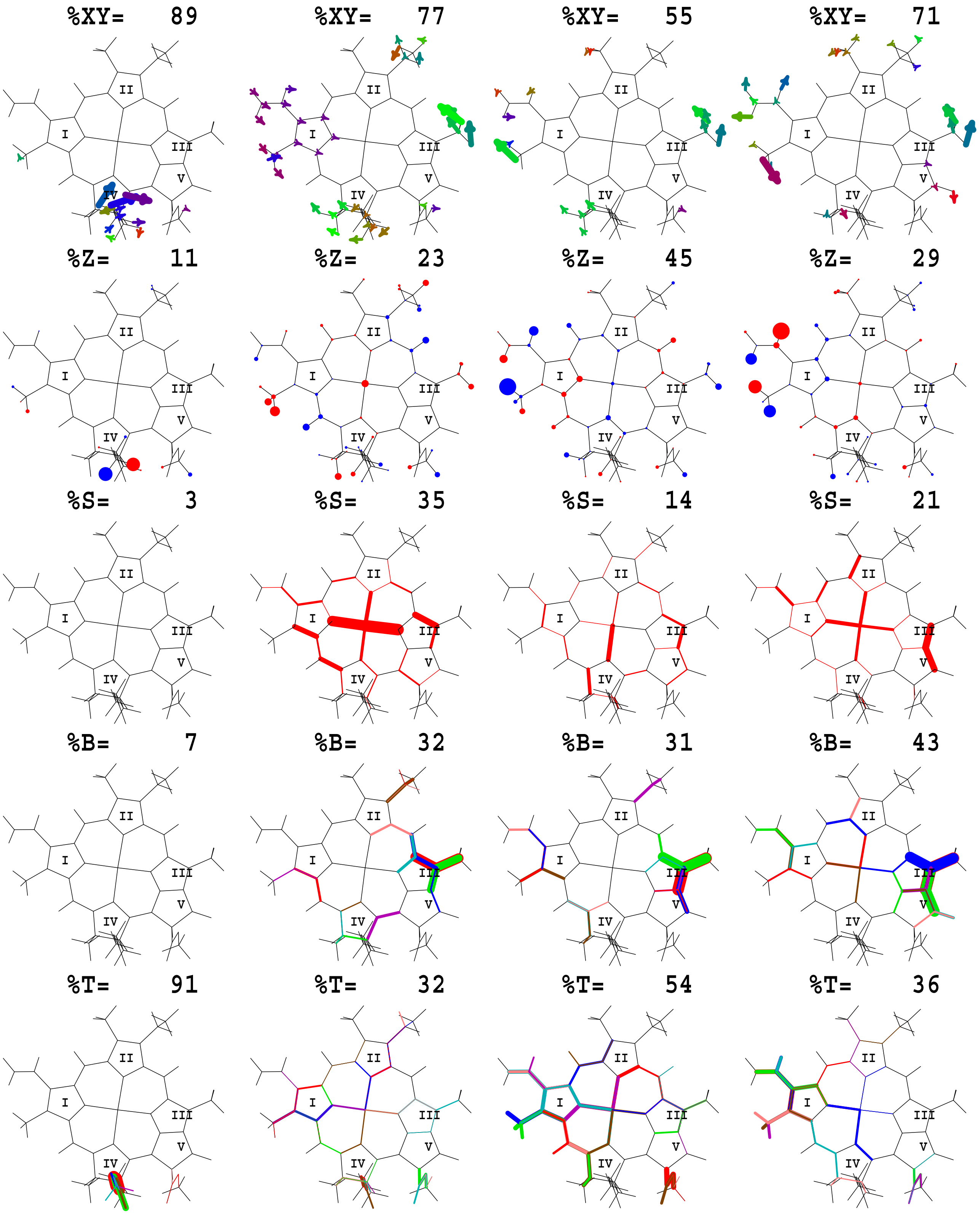

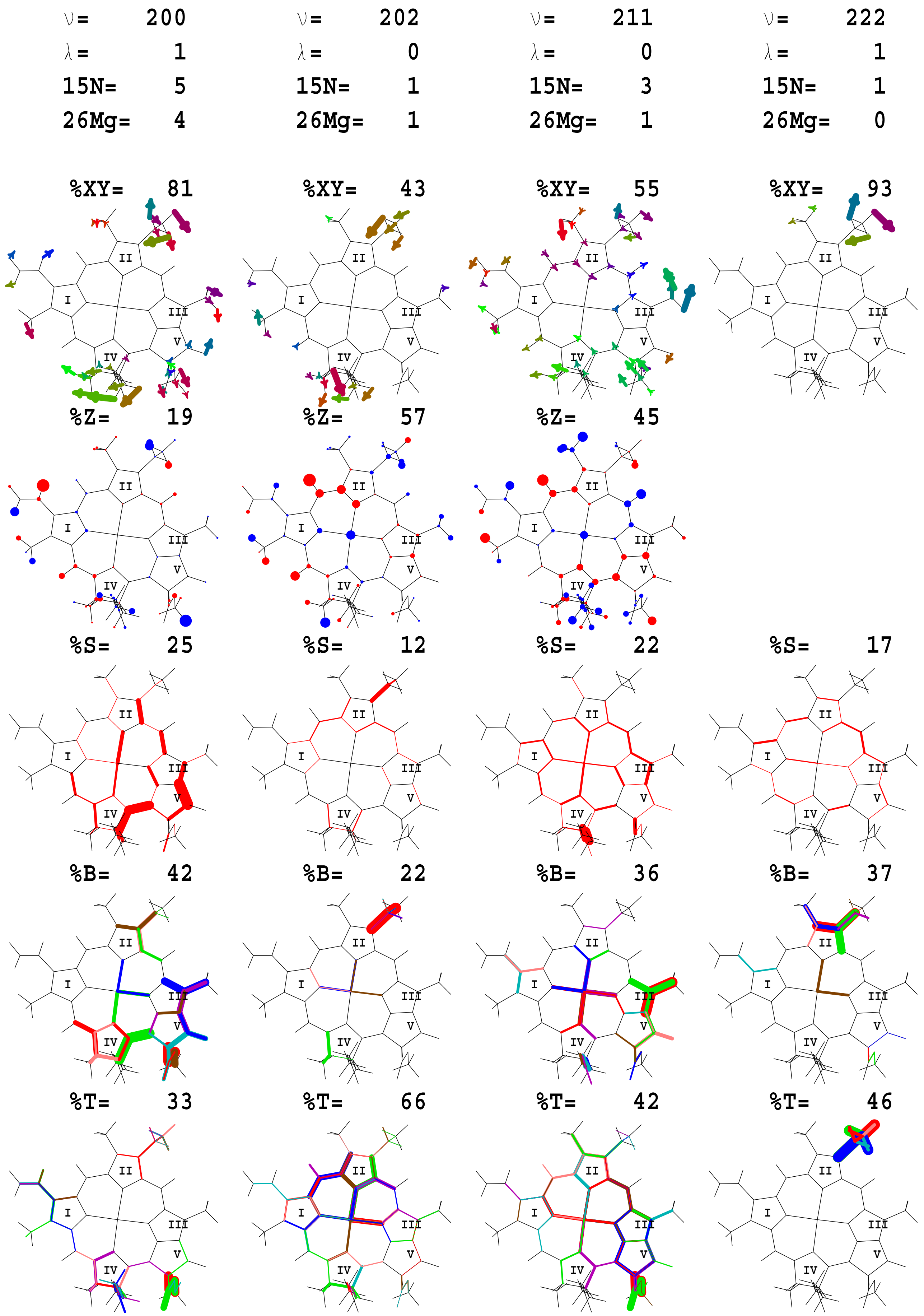

ν= 200
λ= 1
15N= 5
26Mg= 4
%XY= 81
%Z= 19
%S= 25
%B= 42
%T= 33
ν= 202
λ= 0
15N= 1
26Mg= 1
%XY= 43
%Z= 57
%S= 12
%B= 22
%T= 66
ν= 211
λ= 0
15N= 3
26Mg= 1
%XY= 55
%Z= 45
%S= 22
%B= 36
%T= 42
ν= 222
λ= 1
15N= 1
26Mg= 0
%XY= 93
%S= 17
%B= 37
%T= 46
I
II
III
IV
V

ν=	230	236	244	249
λ=	0	0	2	0
15N=	5	1	0	3
26Mg=	0	2	1	1
%XY=	60	73	85	91
%Z=	40	27		
%S=	13	18	32	5
%B=	32	43	35	7
%T=	55	40	33	88

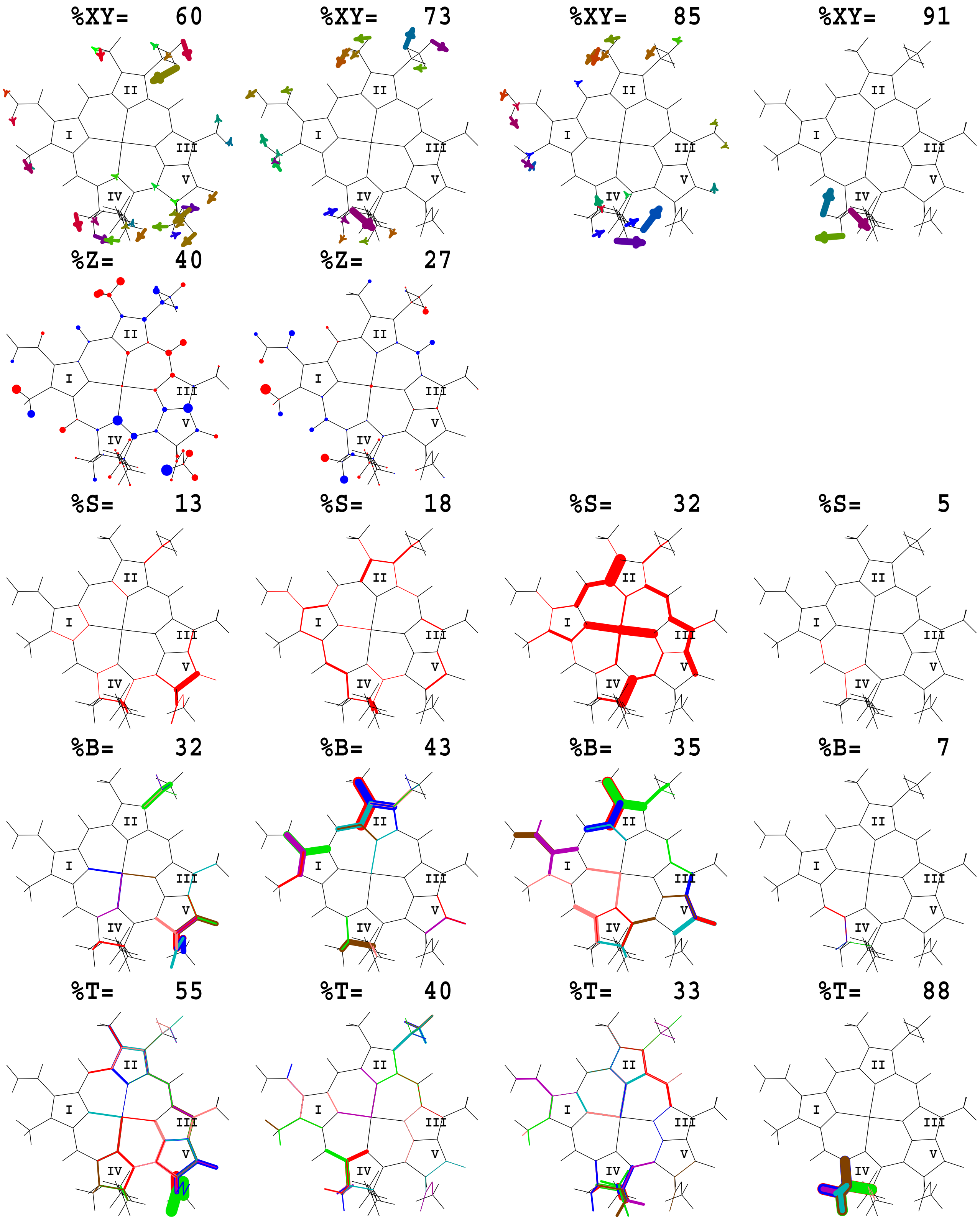

ν= 253	ν= 267	ν= 280	ν= 283
λ= 0	λ= 1	λ= 0	λ= 0
15N= 5	15N= 6	15N= 9	15N= 2
26Mg= 1	26Mg= 0	26Mg= 0	26Mg= 2

%XY= 31	%XY= 59	%XY= 51	%XY= 49

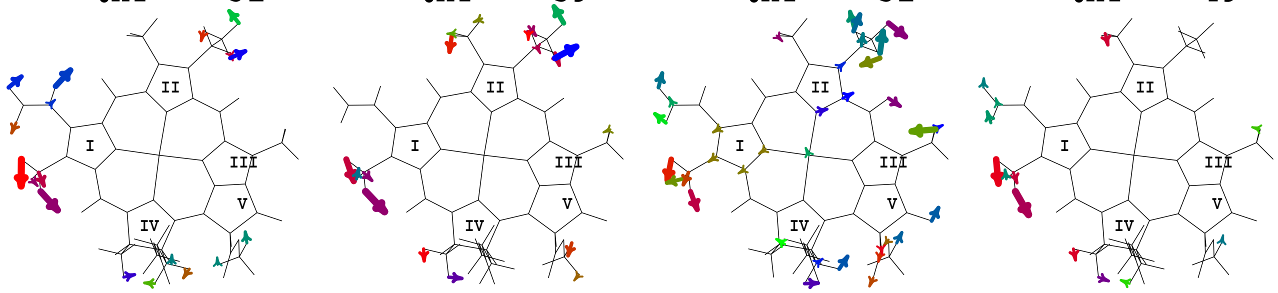

%Z= 69	%Z= 41	%Z= 49	%Z= 51

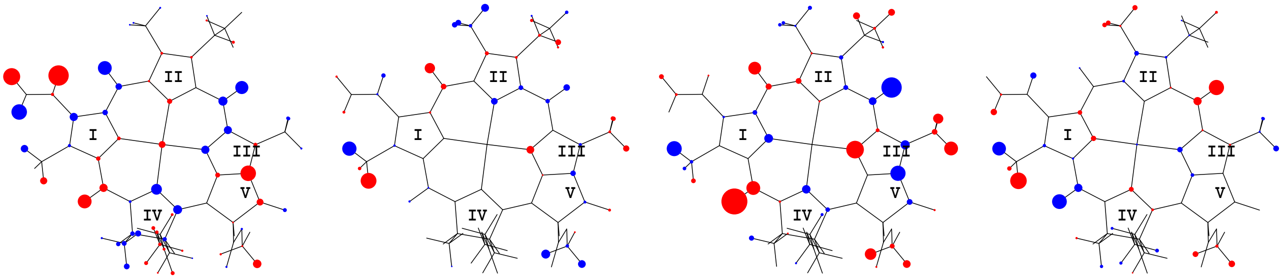

%S= 12	%S= 15	%S= 17	%S= 12

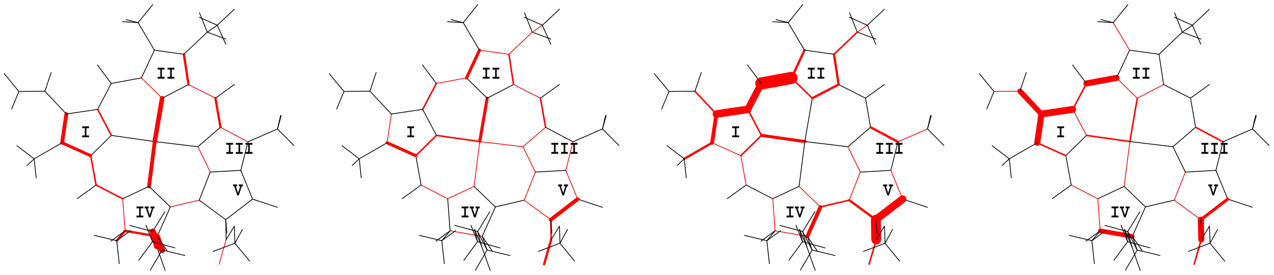

%B= 19	%B= 34	%B= 31	%B= 31

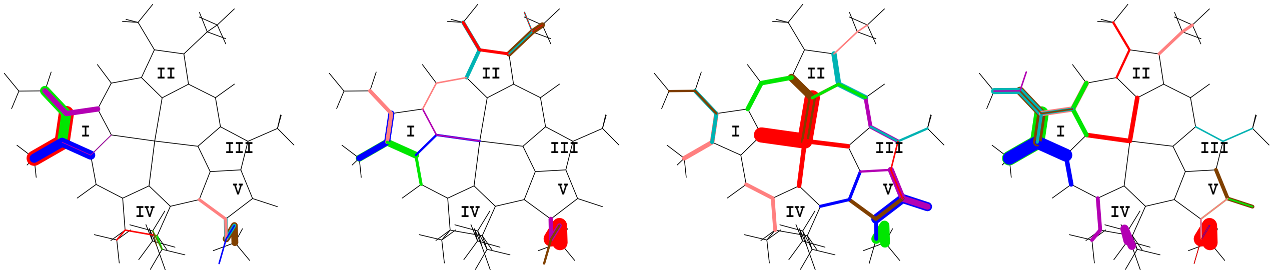

%T= 69	%T= 51	%T= 52	%T= 57

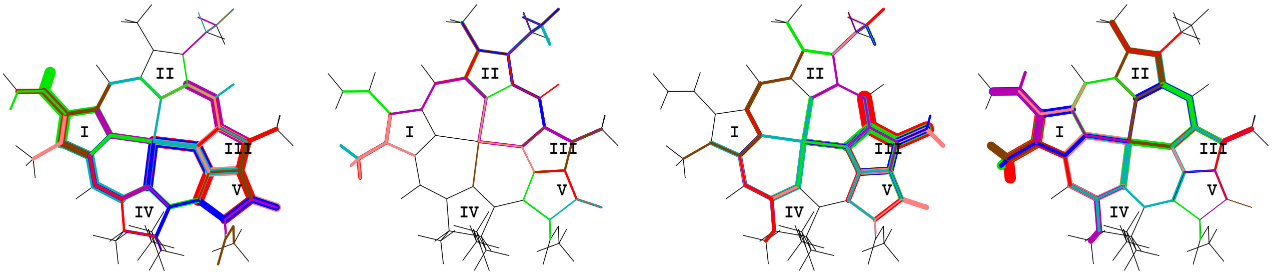

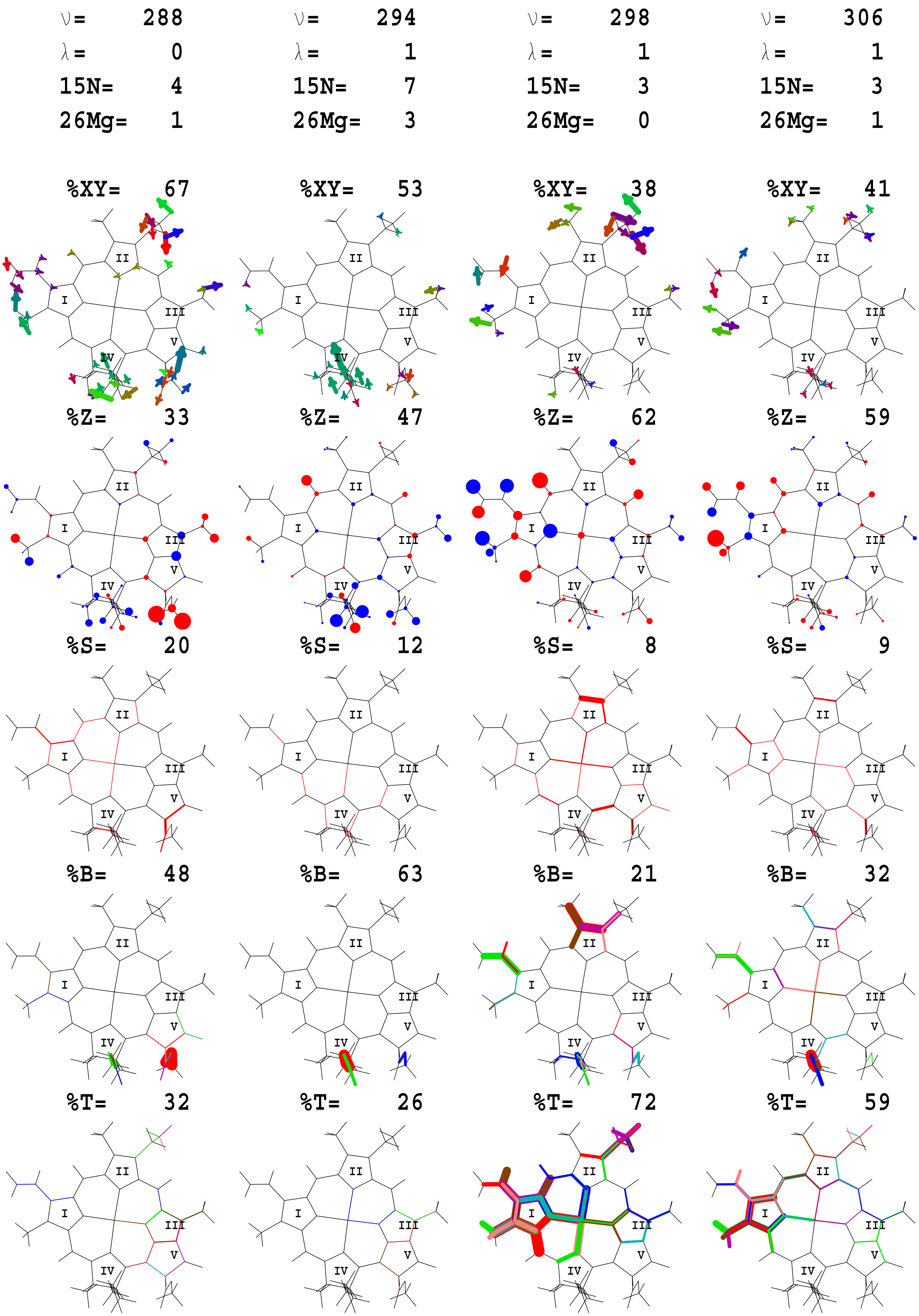

ν= 288
λ= 0
15N= 4
26Mg= 1
%XY= 67
%Z= 33
%S= 20
%B= 48
%T= 32
ν= 294
λ= 1
15N= 7
26Mg= 3
%XY= 53
%Z= 47
%S= 12
%B= 63
%T= 26
ν= 298
λ= 1
15N= 3
26Mg= 0
%XY= 38
%Z= 62
%S= 8
%B= 21
%T= 72
ν= 306
λ= 1
15N= 3
26Mg= 1
%XY= 41
%Z= 59
%S= 9
%B= 32
%T= 59

ν=	312	316	339	345
λ=	0	0	0	2
15N=	5	5	4	4
26Mg=	6	0	2	1

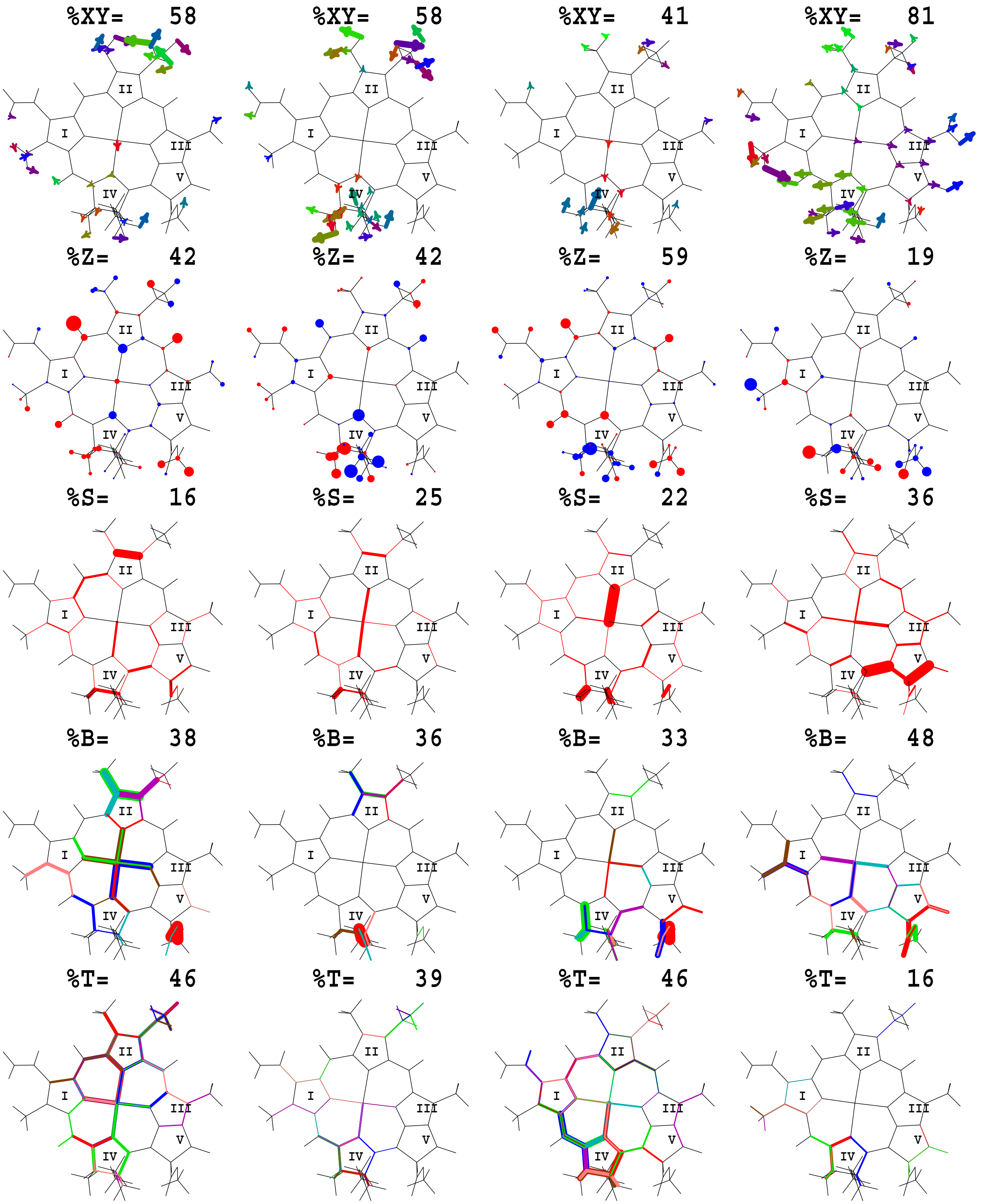

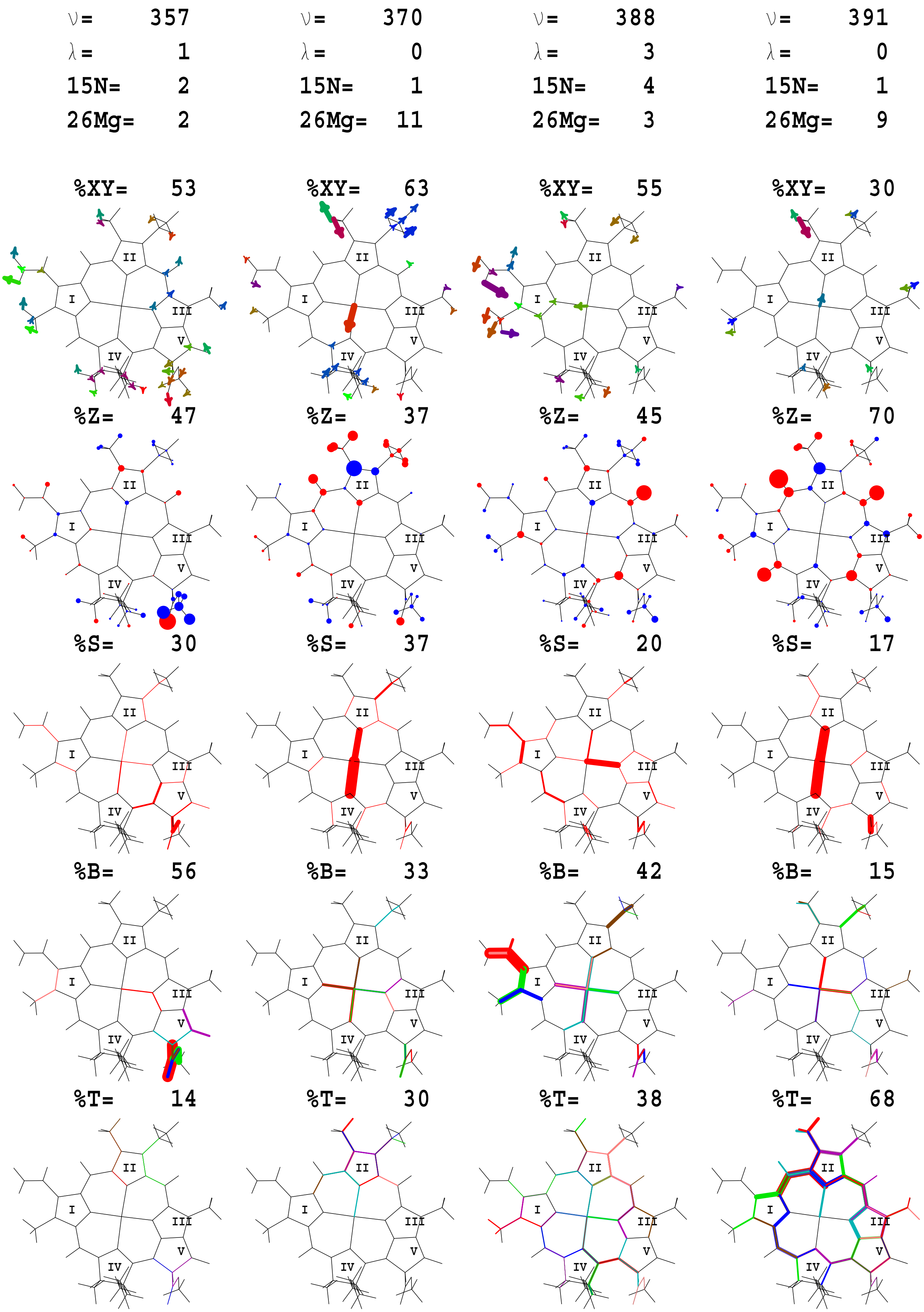

ν= 357
λ= 1
15N= 2
26Mg= 2
%XY= 53
%Z= 47
%S= 30
%B= 56
%T= 14
ν= 370
λ= 0
15N= 1
26Mg= 11
%XY= 63
%Z= 37
%S= 37
%B= 33
%T= 30
ν= 388
λ= 3
15N= 4
26Mg= 3
%XY= 55
%Z= 45
%S= 20
%B= 42
%T= 38
ν= 391
λ= 0
15N= 1
26Mg= 9
%XY= 30
%Z= 70
%S= 17
%B= 15
%T= 68
I
II
III
IV
V

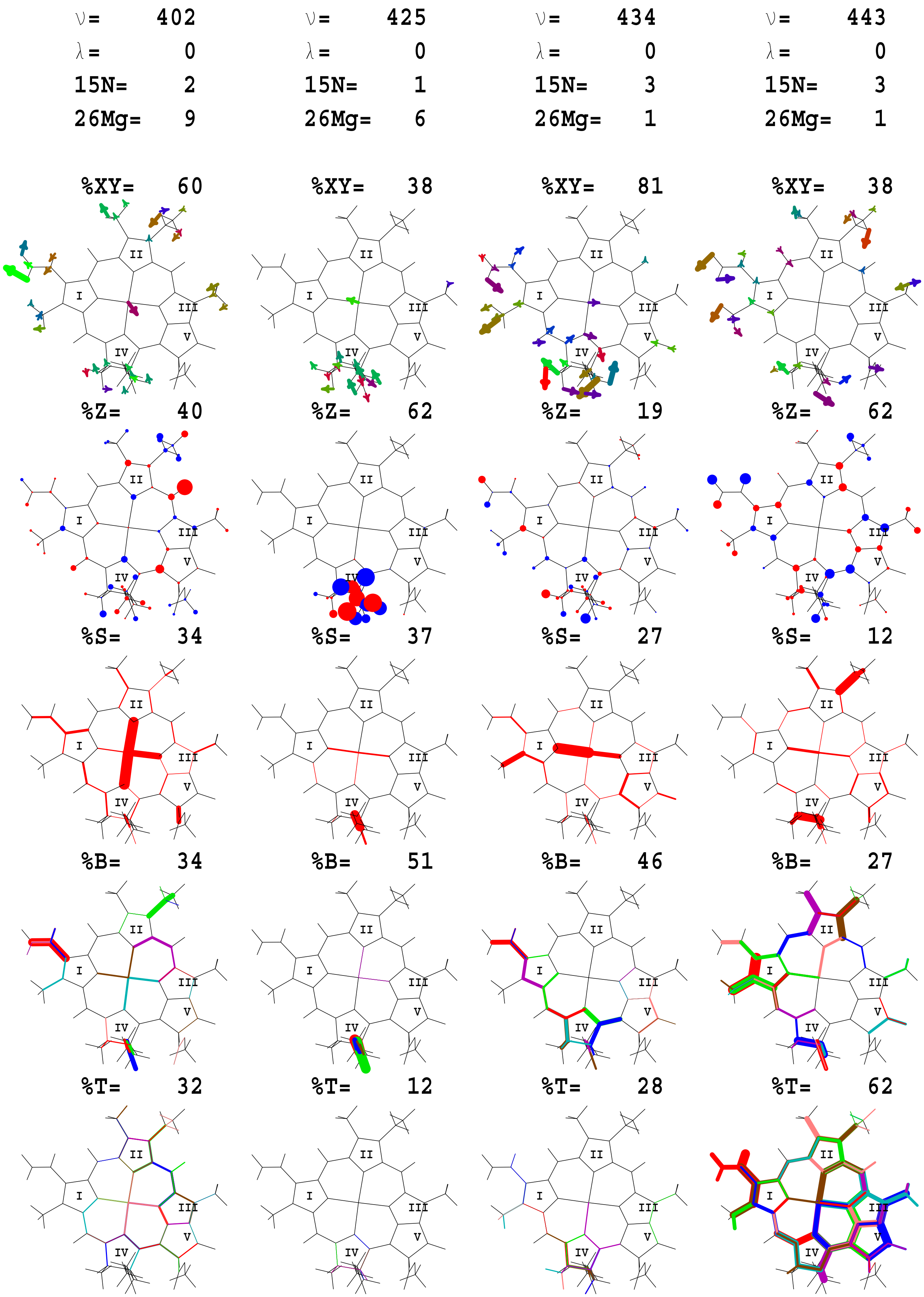

ν= 402
λ= 0
15N= 2
26Mg= 9
%XY= 60
%Z= 40
%S= 34
%B= 34
%T= 32
ν= 425
λ= 0
15N= 1
26Mg= 6
%XY= 38
%Z= 62
%S= 37
%B= 51
%T= 12
ν= 434
λ= 0
15N= 3
26Mg= 1
%XY= 81
%Z= 19
%S= 27
%B= 46
%T= 28
ν= 443
λ= 0
15N= 3
26Mg= 1
%XY= 38
%Z= 62
%S= 12
%B= 27
%T= 62
I
II
III
IV
V

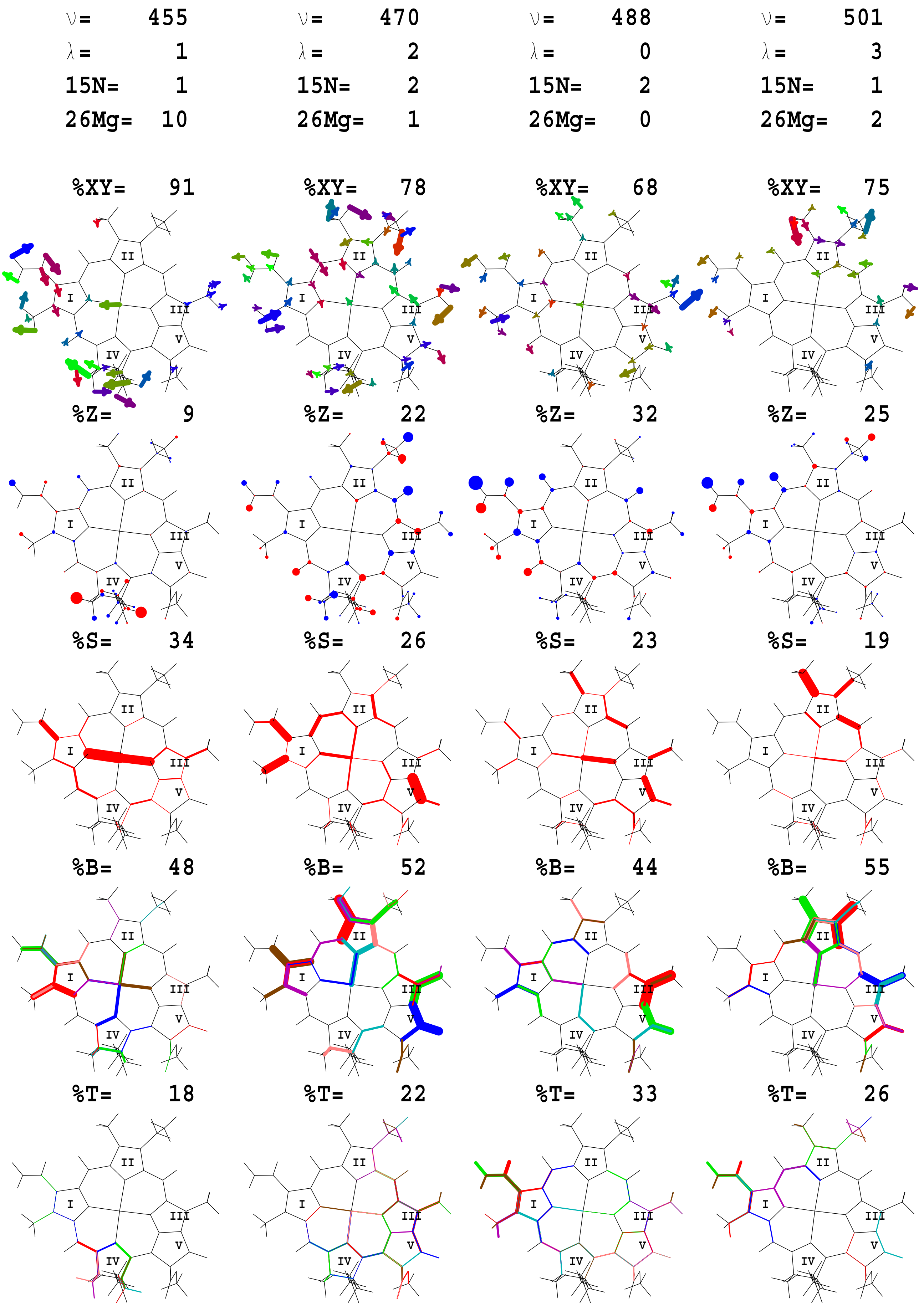

ν= 455
λ= 1
15N= 1
26Mg= 10
%XY= 91
%Z= 9
%S= 34
%B= 48
%T= 18
ν= 470
λ= 2
15N= 2
26Mg= 1
%XY= 78
%Z= 22
%S= 26
%B= 52
%T= 22
ν= 488
λ= 0
15N= 2
26Mg= 0
%XY= 68
%Z= 32
%S= 23
%B= 44
%T= 33
ν= 501
λ= 3
15N= 1
26Mg= 2
%XY= 75
%Z= 25
%S= 19
%B= 55
%T= 26
I
II
III
IV
V

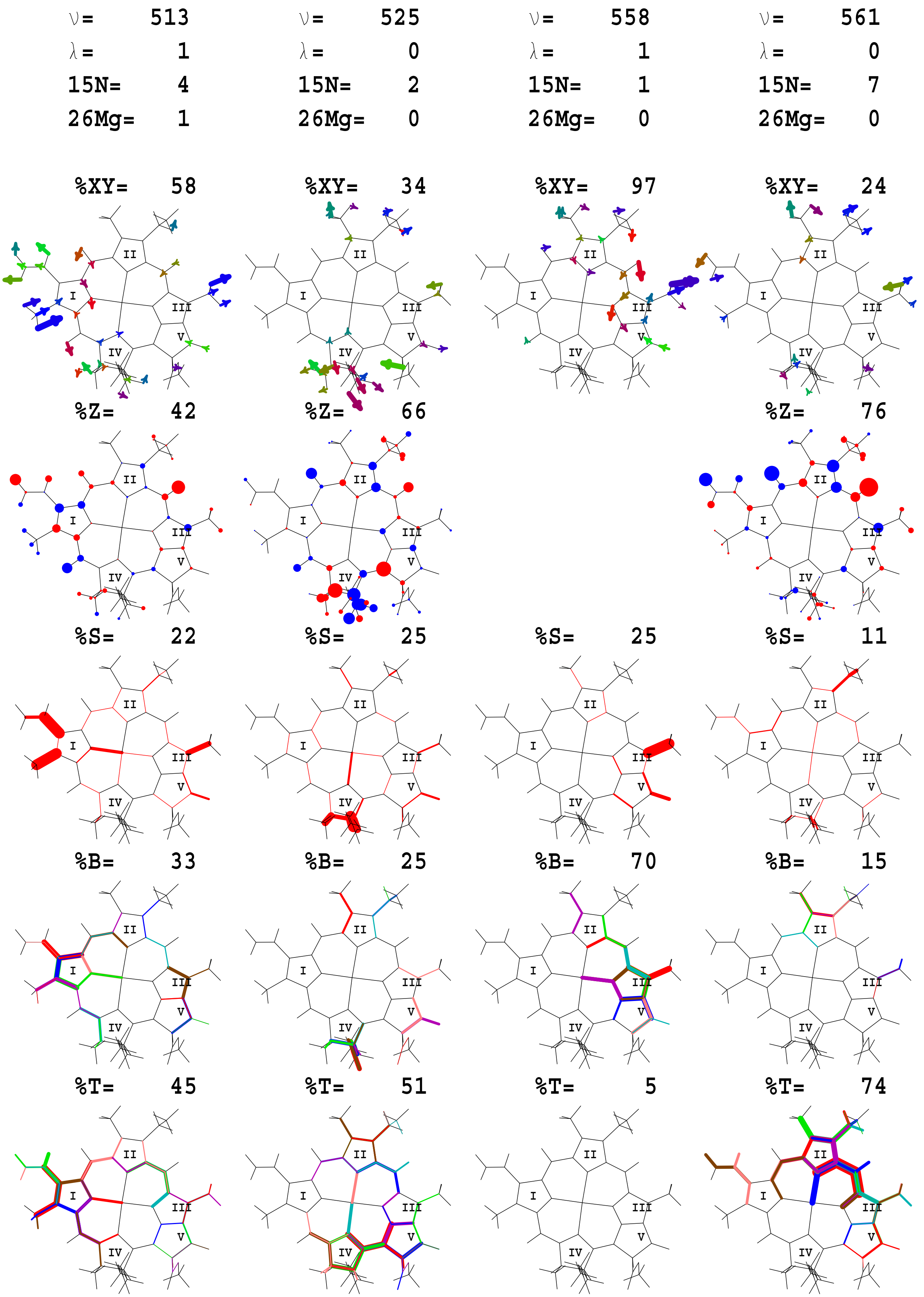
ν= 513
λ= 1
15N= 4
26Mg= 1
%XY= 58
%Z= 42
%S= 22
%B= 33
%T= 45
ν= 525
λ= 0
15N= 2
26Mg= 0
%XY= 34
%Z= 66
%S= 25
%B= 25
%T= 51
ν= 558
λ= 1
15N= 1
26Mg= 0
%XY= 97
%S= 25
%B= 70
%T= 5
ν= 561
λ= 0
15N= 7
26Mg= 0
%XY= 24
%Z= 76
%S= 11
%B= 15
%T= 74

ν= 573	ν= 582	ν= 593	ν= 624
λ= 0	λ= 0	λ= 2	λ= 1
15N= 0	15N= 0	15N= 0	15N= 1
26Mg= 0	26Mg= 0	26Mg= 0	26Mg= 0

%XY= 32 %XY= 92 %XY= 73 %XY= 91

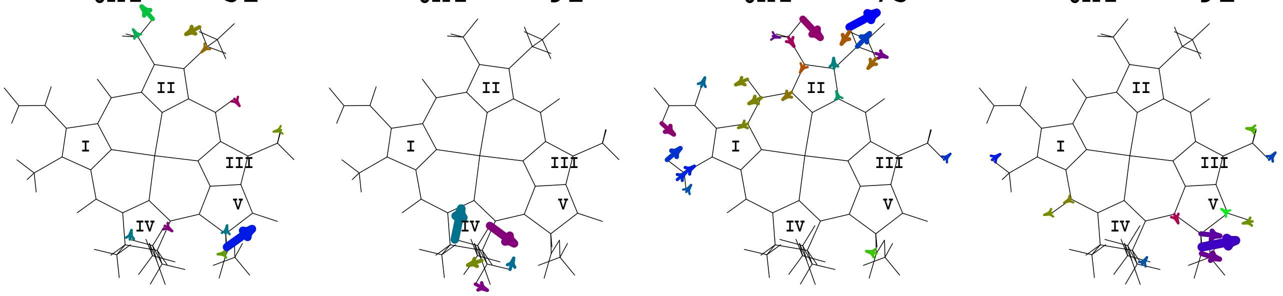

%Z= 68 %Z= 27

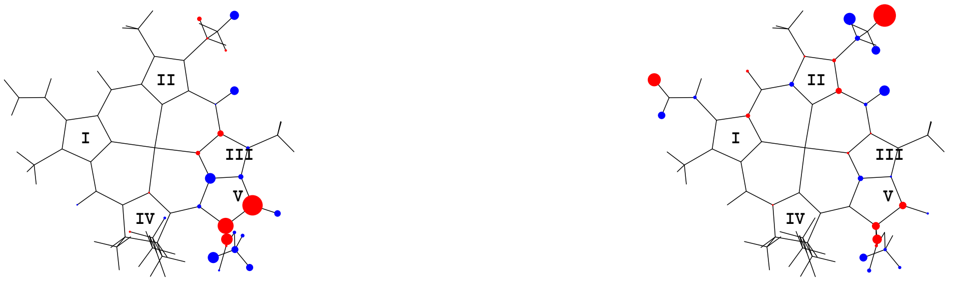

%S= 18 %S= 0 %S= 31 %S= 27

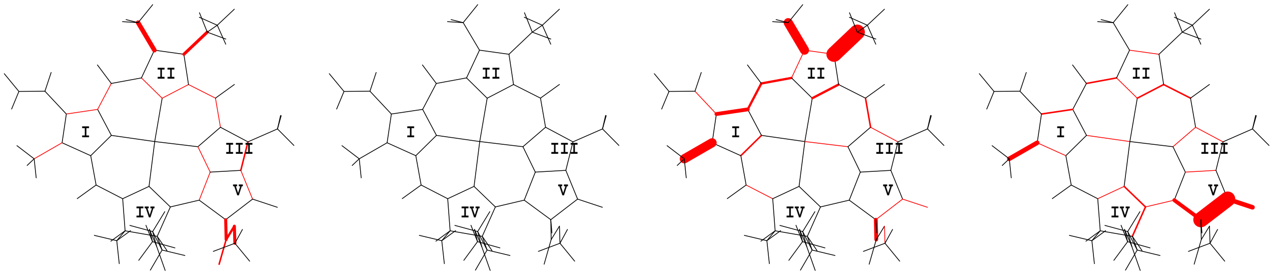

%B= 18 %B= 5 %B= 44 %B= 36

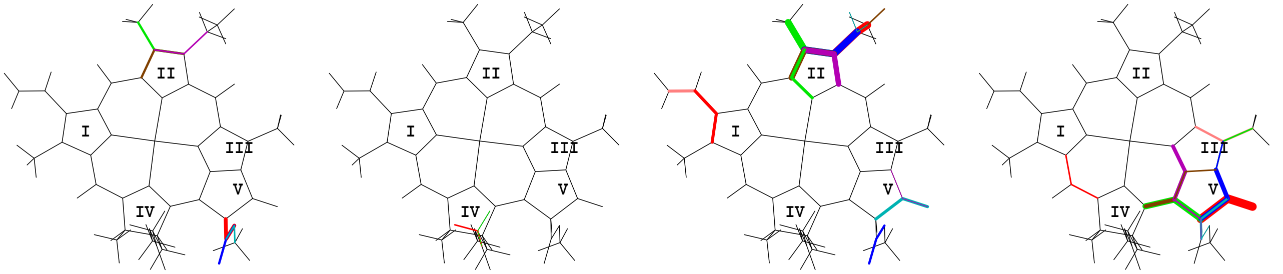

%T= 65 %T= 94 %T= 24 %T= 37

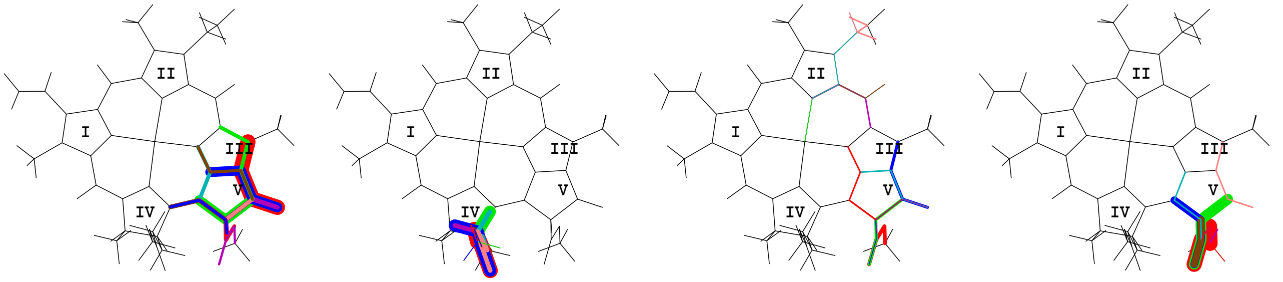

ν=	639	657	668	680
λ=	1	1	1	1
15N=	1	1	1	3
26Mg=	0	0	0	0

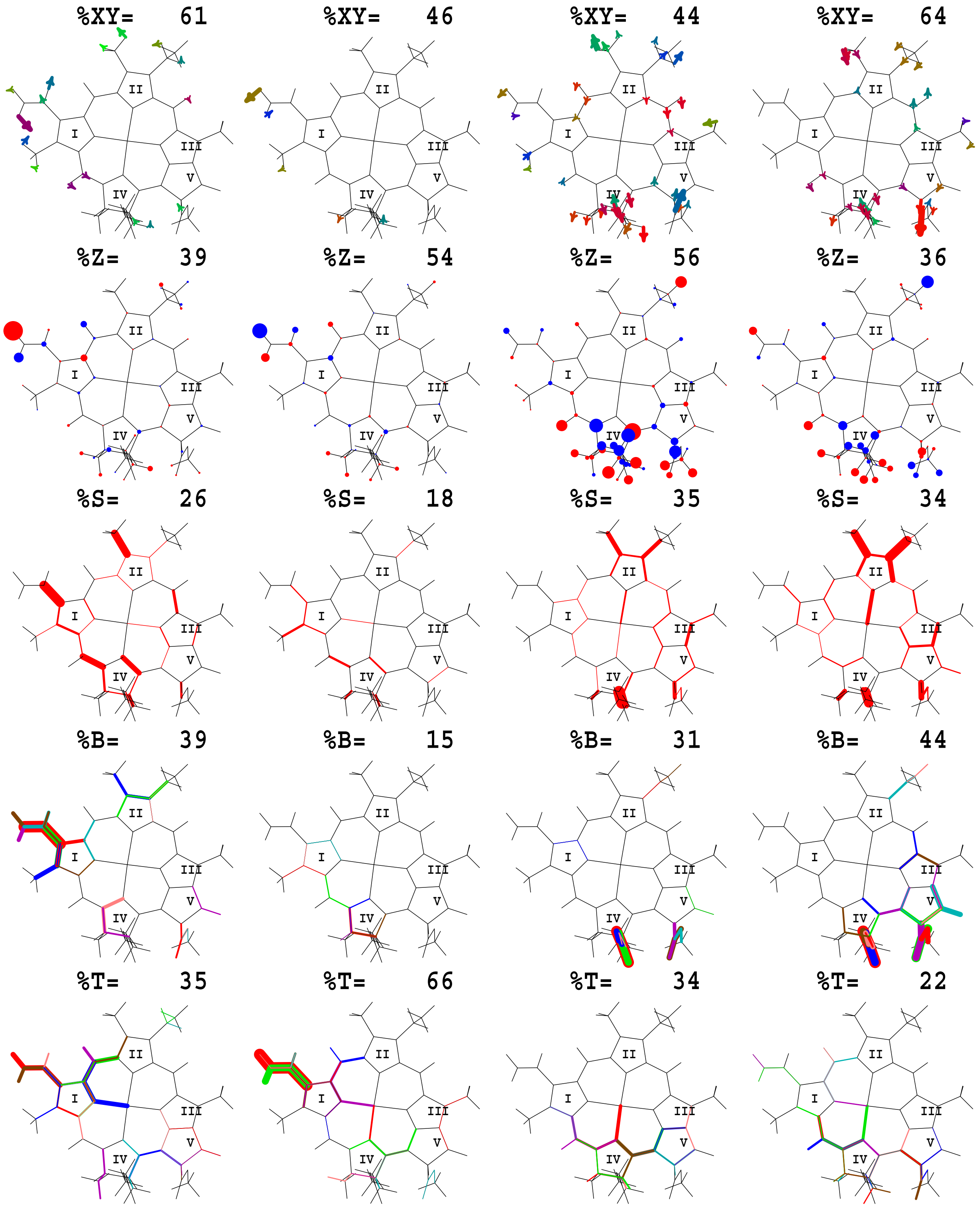

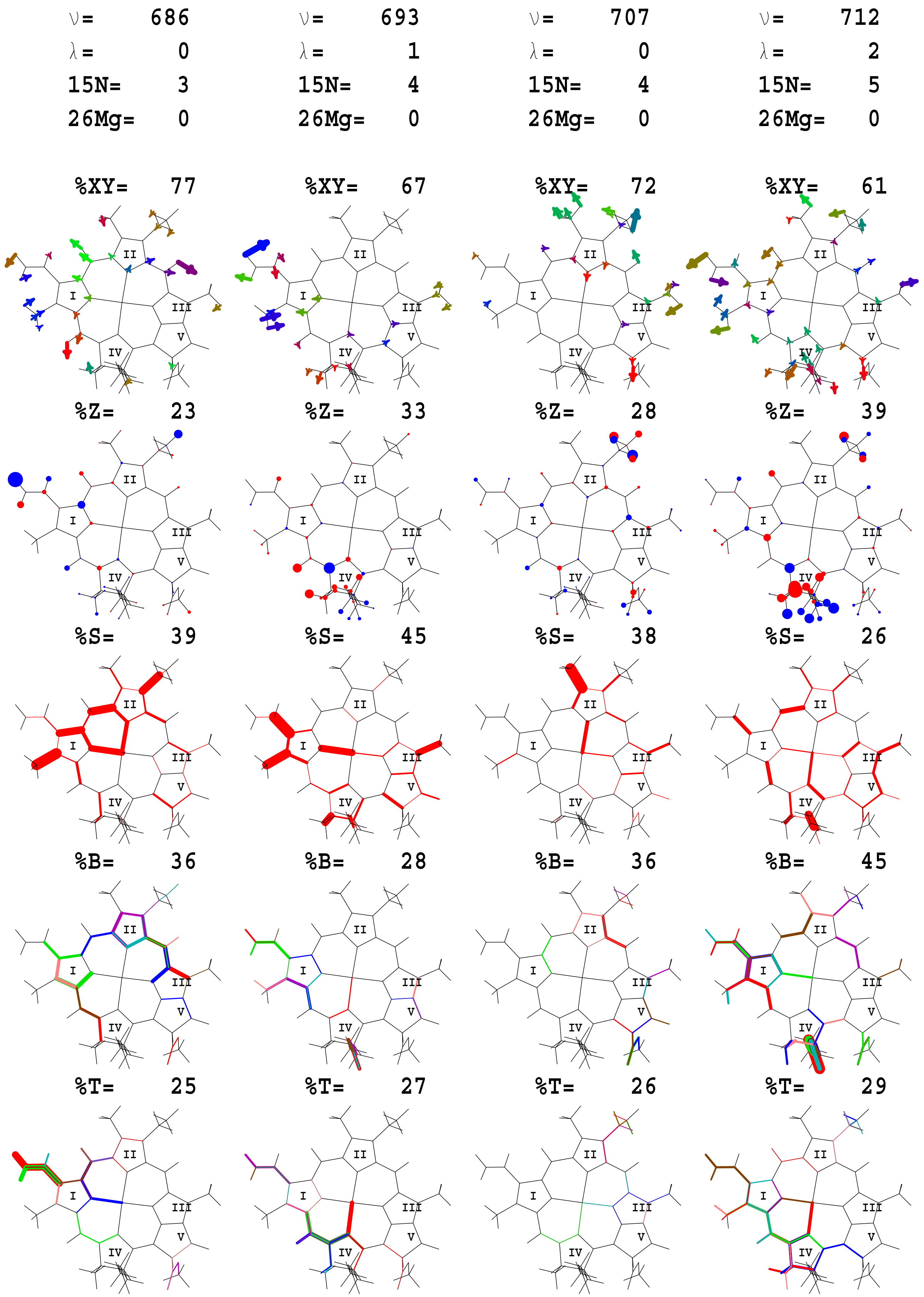

ν= 686
λ= 0
15N= 3
26Mg= 0
%XY= 77
%Z= 23
%S= 39
%B= 36
%T= 25
ν= 693
λ= 1
15N= 4
26Mg= 0
%XY= 67
%Z= 33
%S= 45
%B= 28
%T= 27
ν= 707
λ= 0
15N= 4
26Mg= 0
%XY= 72
%Z= 28
%S= 38
%B= 36
%T= 26
ν= 712
λ= 2
15N= 5
26Mg= 0
%XY= 61
%Z= 39
%S= 26
%B= 45
%T= 29
I
II
III
IV
V

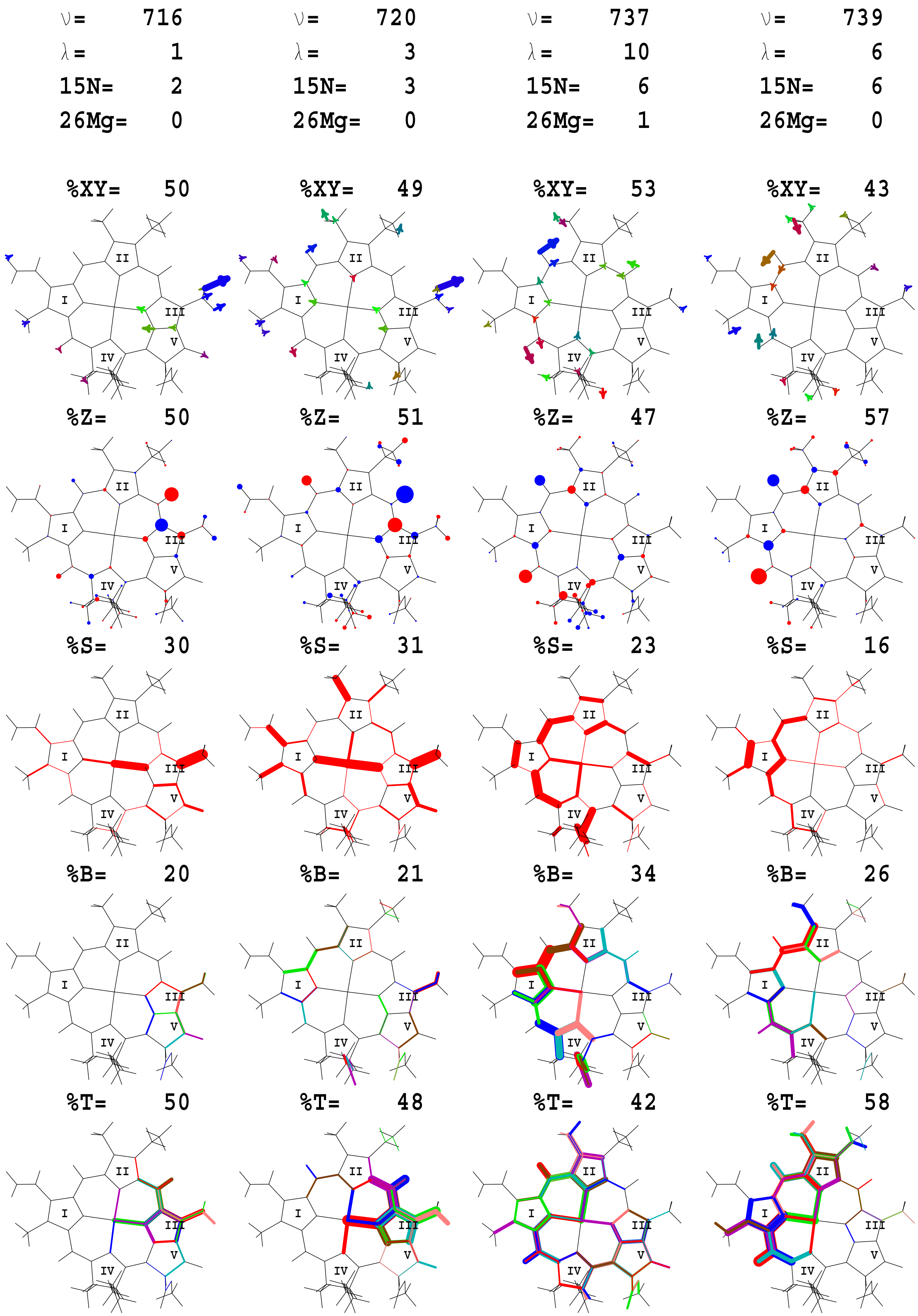
ν= 716
λ= 1
15N= 2
26Mg= 0
%XY= 50
%Z= 50
%S= 30
%B= 20
%T= 50
ν= 720
λ= 3
15N= 3
26Mg= 0
%XY= 49
%Z= 51
%S= 31
%B= 21
%T= 48
ν= 737
λ= 10
15N= 6
26Mg= 1
%XY= 53
%Z= 47
%S= 23
%B= 34
%T= 42
ν= 739
λ= 6
15N= 6
26Mg= 0
%XY= 43
%Z= 57
%S= 16
%B= 26
%T= 58
I
II
III
IV
V

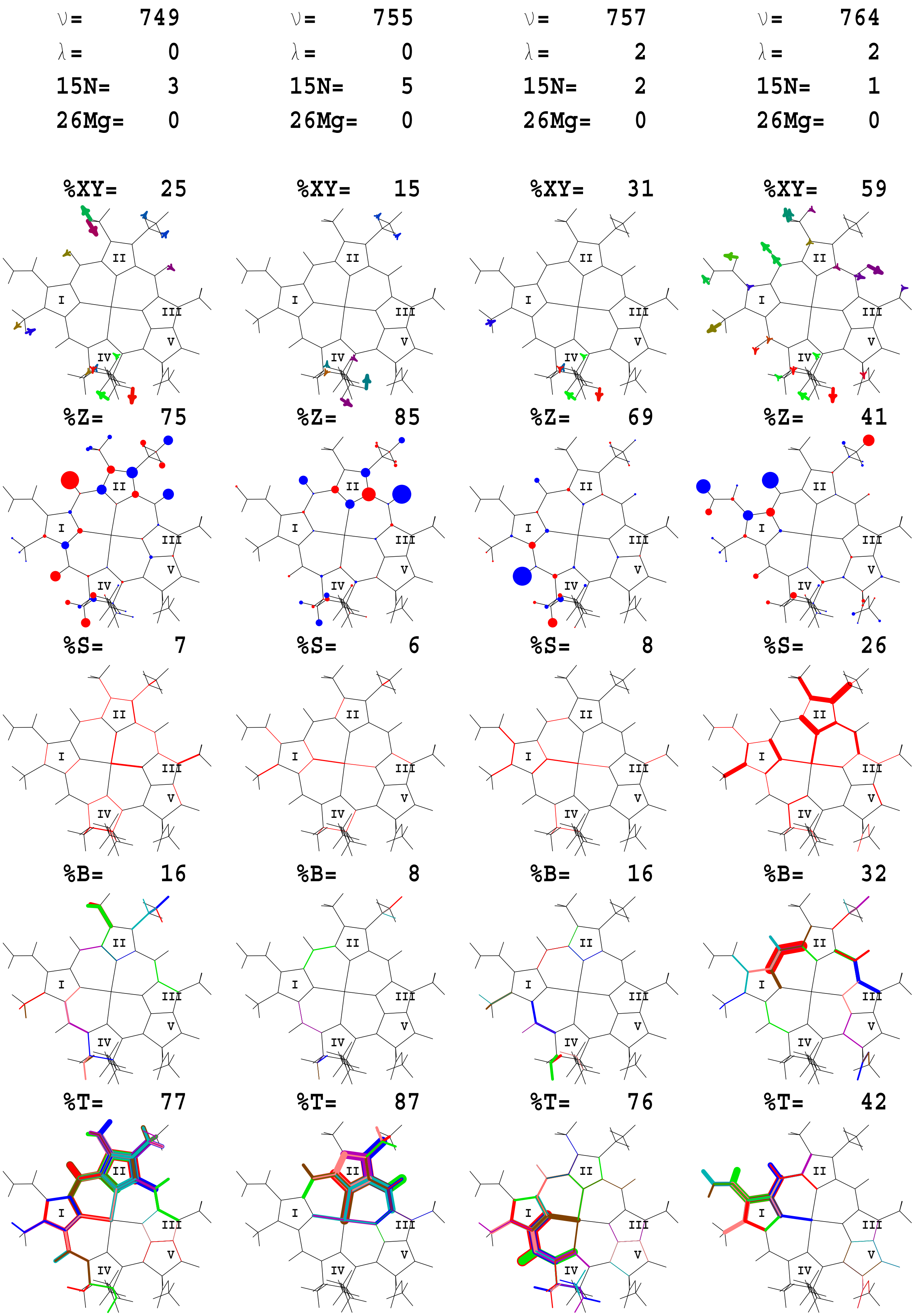

ν= 749
λ= 0
15N= 3
26Mg= 0
%XY= 25
%Z= 75
%S= 7
%B= 16
%T= 77
ν= 755
λ= 0
15N= 5
26Mg= 0
%XY= 15
%Z= 85
%S= 6
%B= 8
%T= 87
ν= 757
λ= 2
15N= 2
26Mg= 0
%XY= 31
%Z= 69
%S= 8
%B= 16
%T= 76
ν= 764
λ= 2
15N= 1
26Mg= 0
%XY= 59
%Z= 41
%S= 26
%B= 32
%T= 42
I
II
III
IV
V

ν=	769	779	783	786
λ=	1	0	0	0
15N=	2	2	4	3
26Mg=	0	0	0	0
%XY=	63	59	60	48
%Z=	37	41	40	52
%S=	18	12	19	17
%B=	36	37	33	31
%T=	46	51	48	52

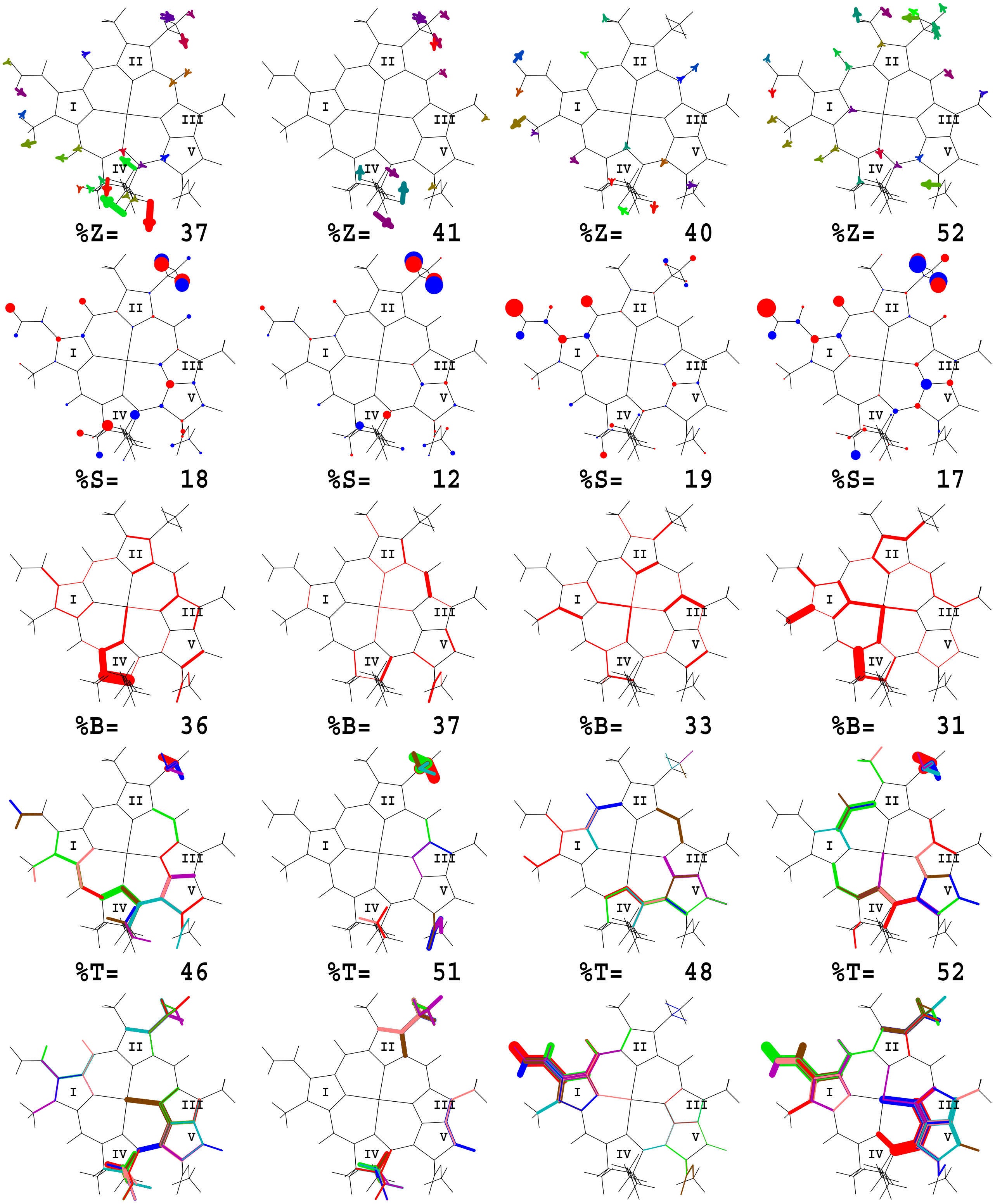

ν=	789	806	831	839
λ=	0	1	1	2
15N=	4	2	0	1
26Mg=	0	0	0	0

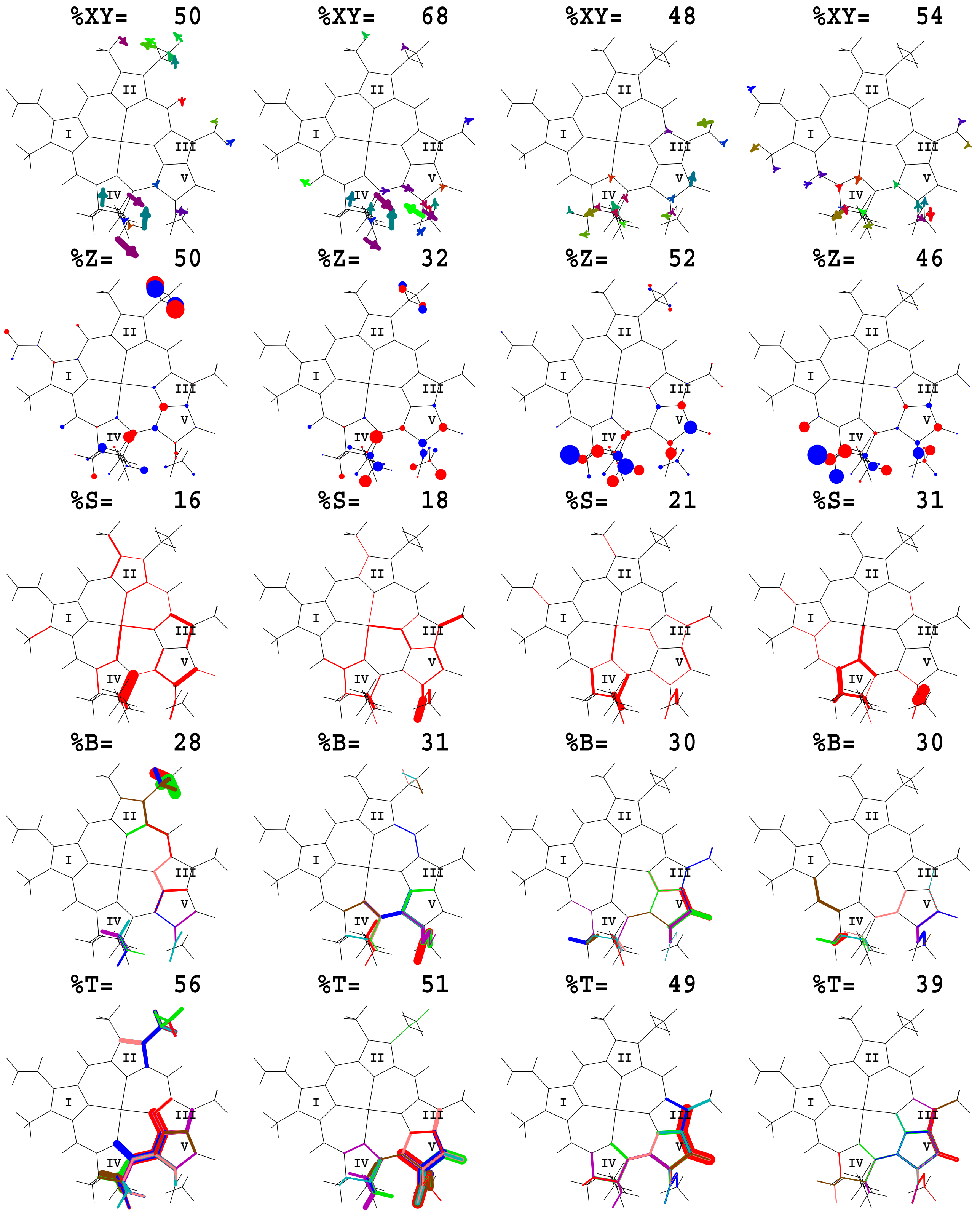

ν=	846	ν=	856	ν=	884	ν=	891
λ=	0	λ=	0	λ=	1	λ=	1
15N=	3	15N=	1	15N=	0	15N=	4
26Mg=	0	26Mg=	0	26Mg=	0	26Mg=	0

%XY= 66

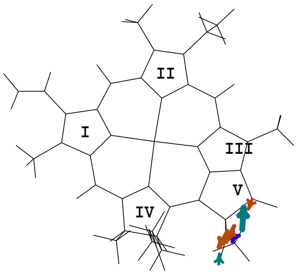

%XY= 75

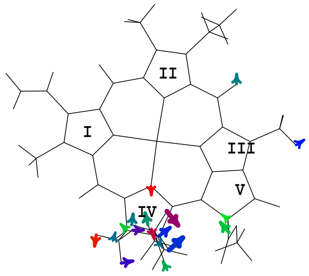

%XY= 60

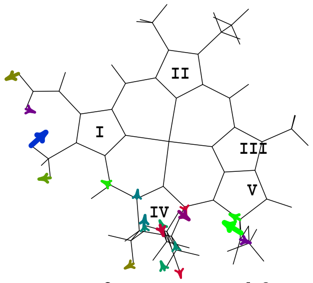

%Z= 97

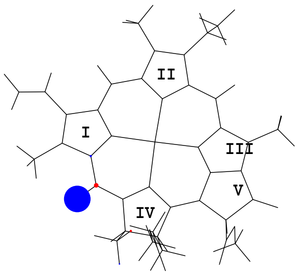

%Z= 34

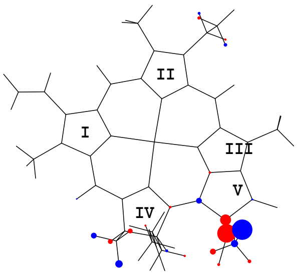

%Z= 25

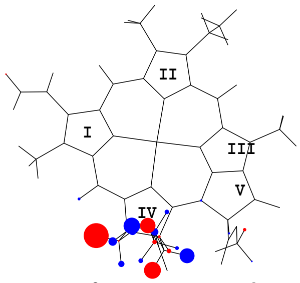

%Z= 40

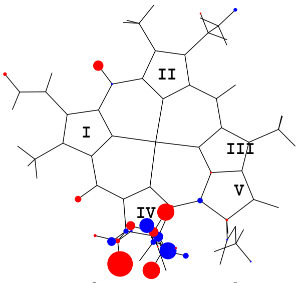

%S= 1

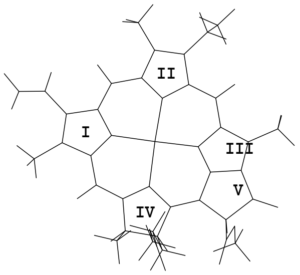

%S= 45

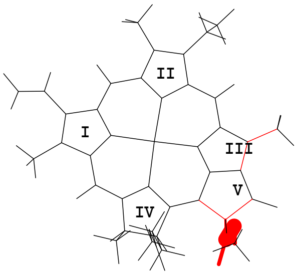

%S= 50

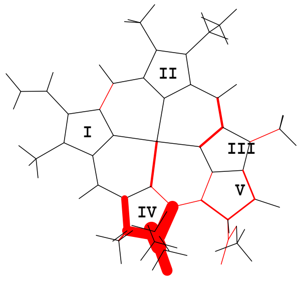

%S= 47

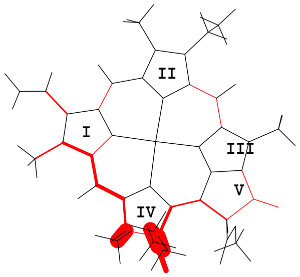

%B= 3

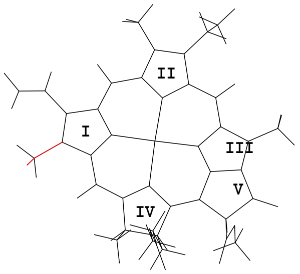

%B= 30

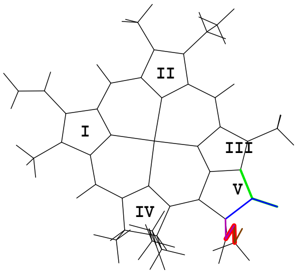

%B= 33

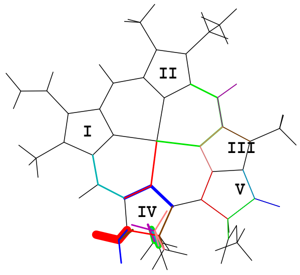

%B= 28

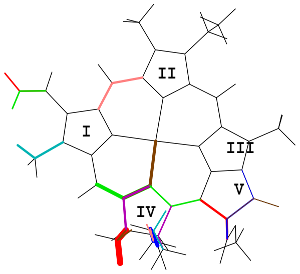

%T= 96

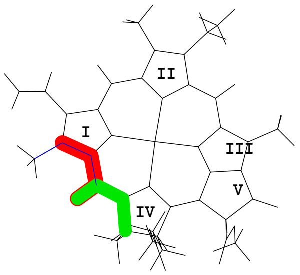

%T= 25

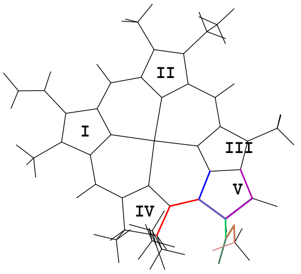

%T= 17

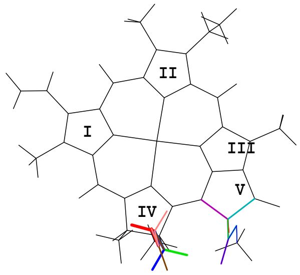

%T= 25

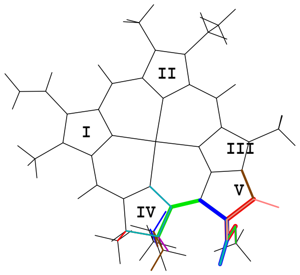

ν=	895	897	898	908
λ=	7	7	1	7
15N=	0	2	4	2
26Mg=	0	0	0	0

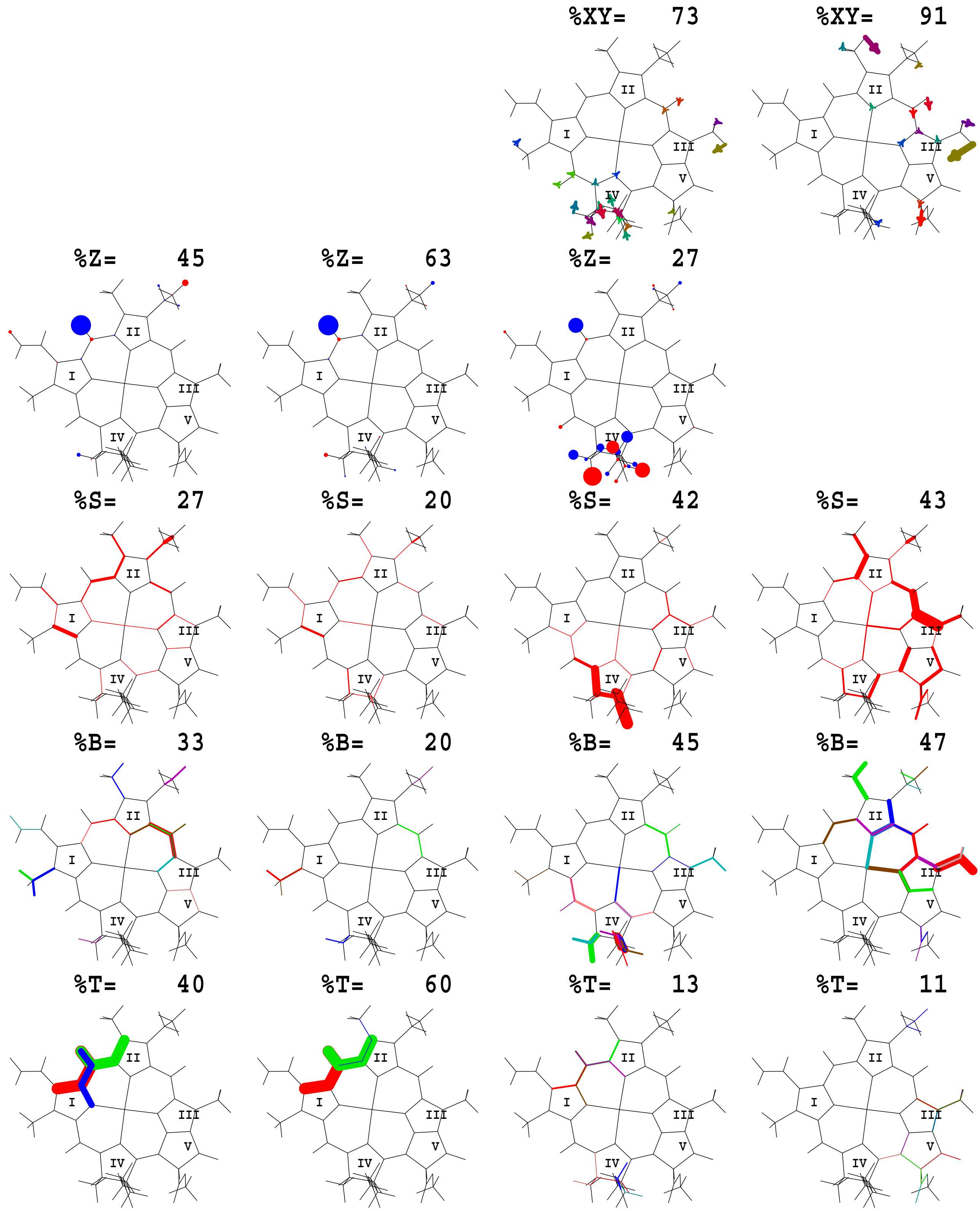

ν=	917	935	957	960
λ=	0	3	0	0
15N=	5	0	6	1
26Mg=	0	0	0	0

%XY= 82 %XY= 68 %XY= 36

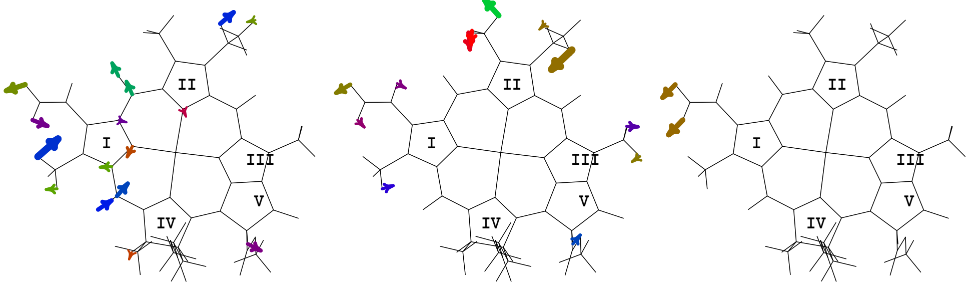

%Z= 98 %Z= 18 %Z= 32 %Z= 64

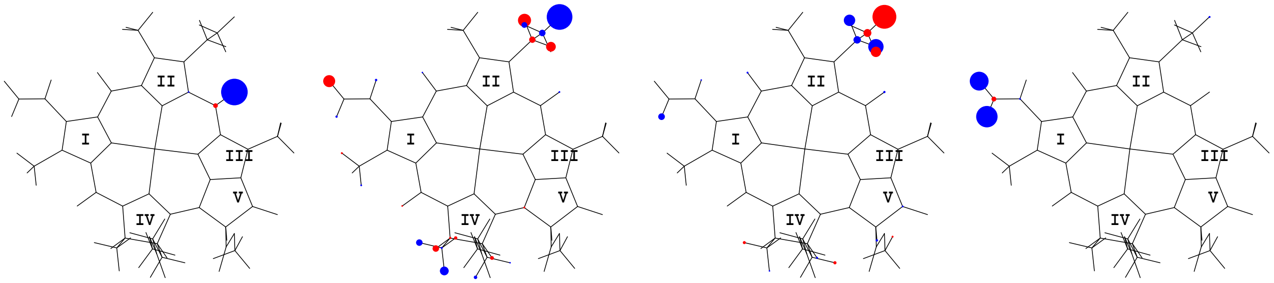

%S= 0 %S= 35 %S= 52 %S= 1

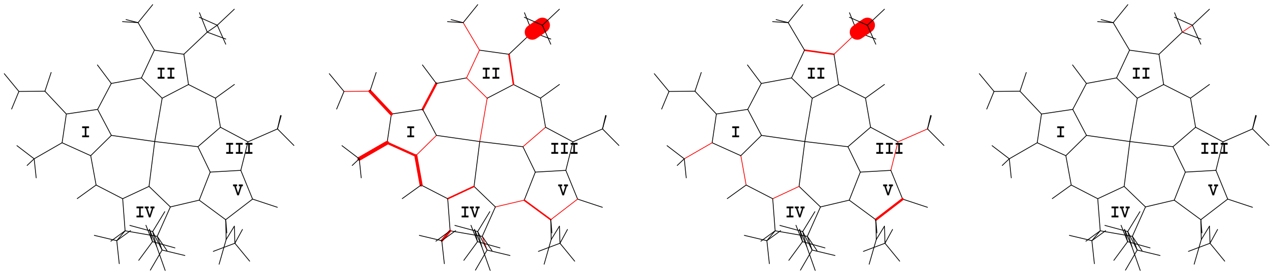

%B= 2 %B= 58 %B= 35 %B= 1

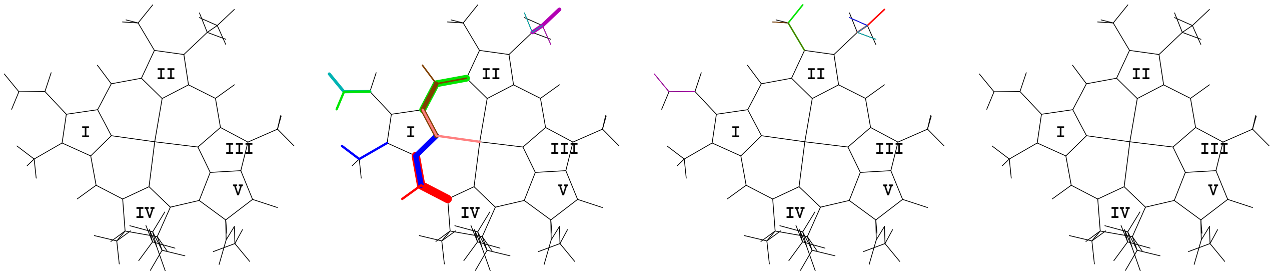

%T= 98 %T= 7 %T= 13 %T= 98

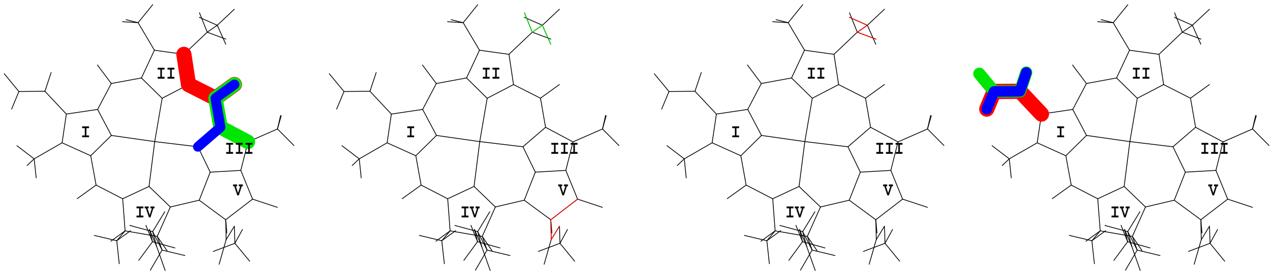

ν=	963	977	986	990
λ=	2	40	4	0
15N=	6	2	1	1
26Mg=	0	0	0	0
%XY=	86	92	74	87
%Z=	14		26	13
%S=	41	34	43	32
%B=	48	49	39	51
%T=	10	17	18	17

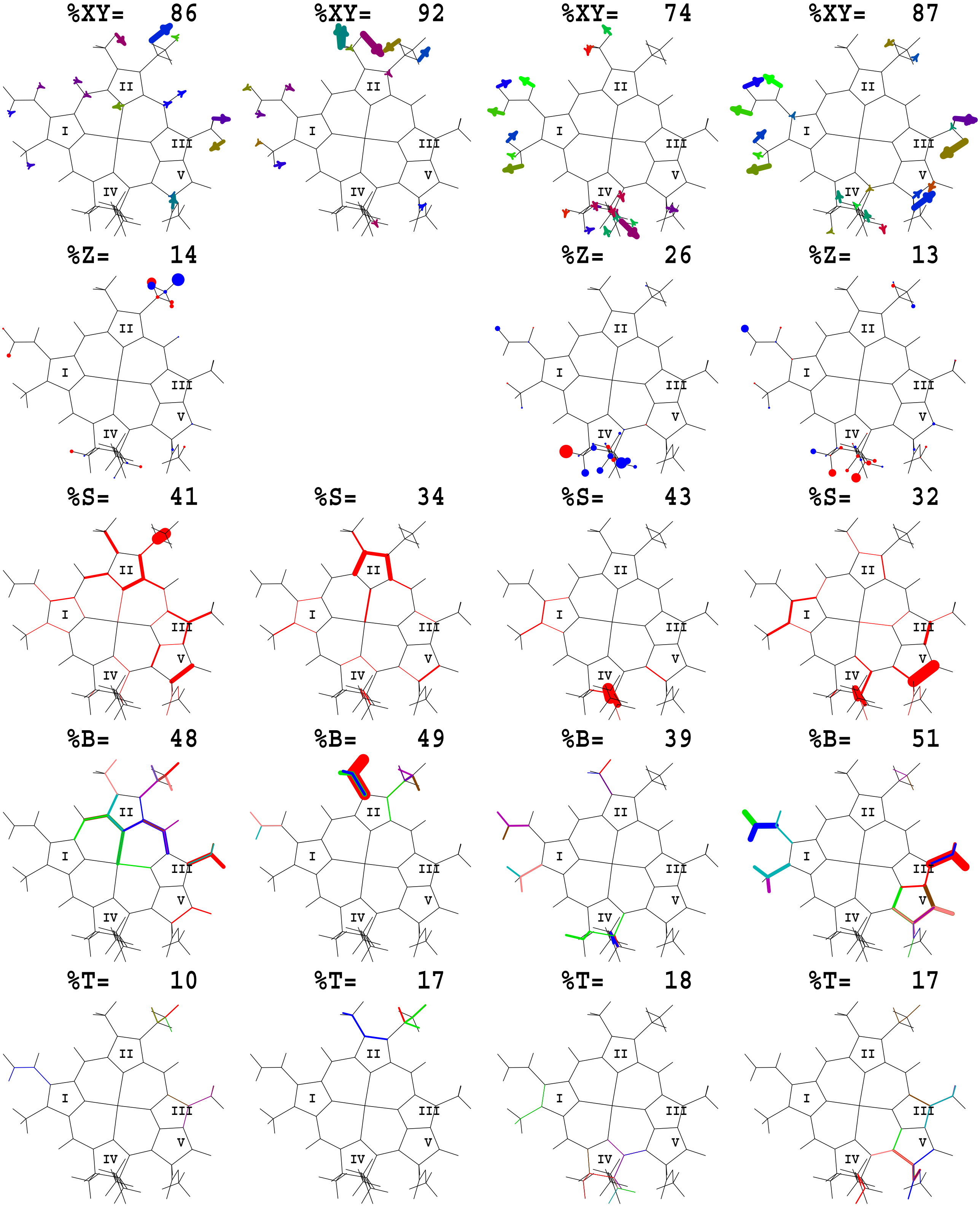

ν=	1015	1024	1028	1035
λ=	3	2	1	2
15N=	0	1	2	3
26Mg=	0	0	0	0

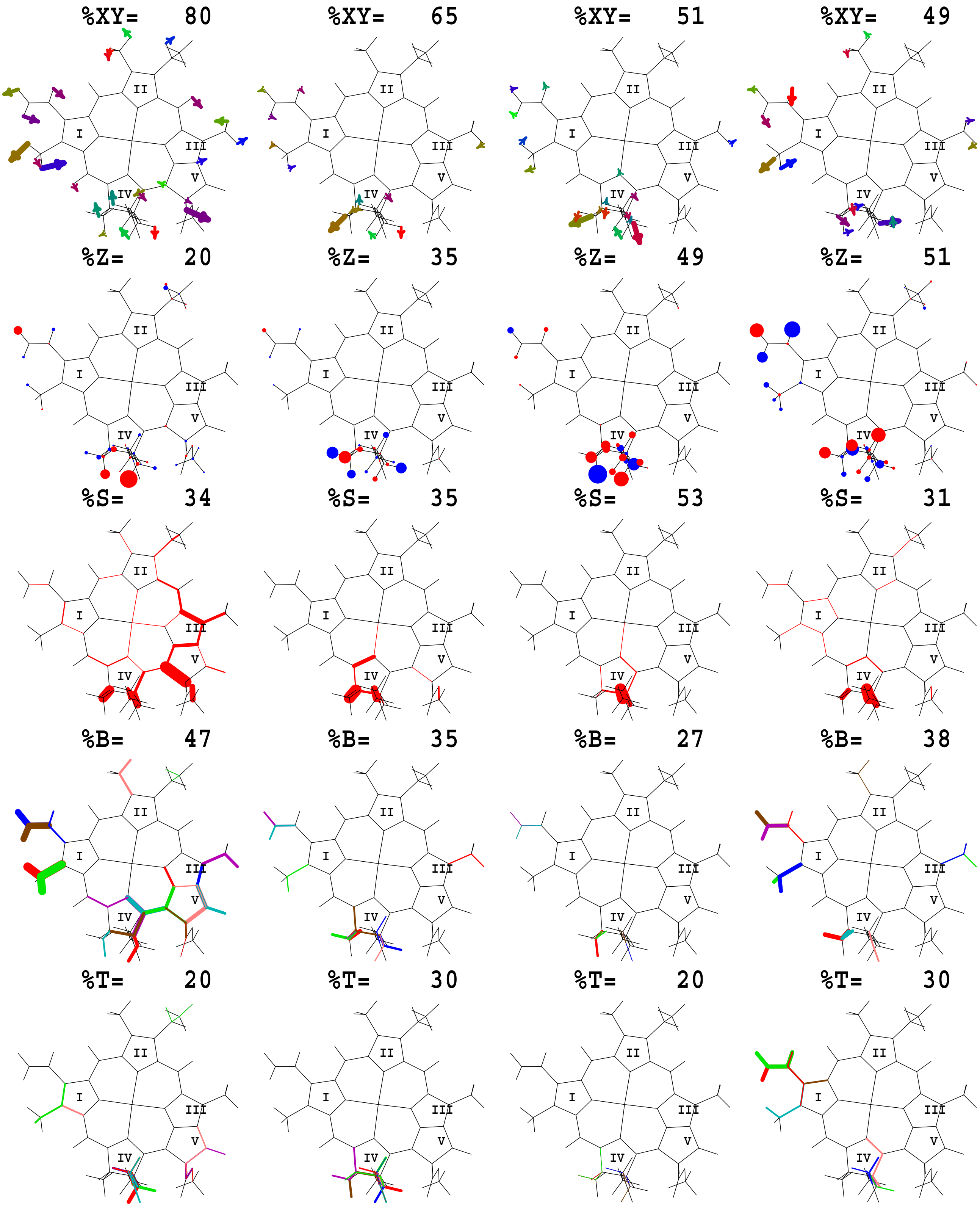

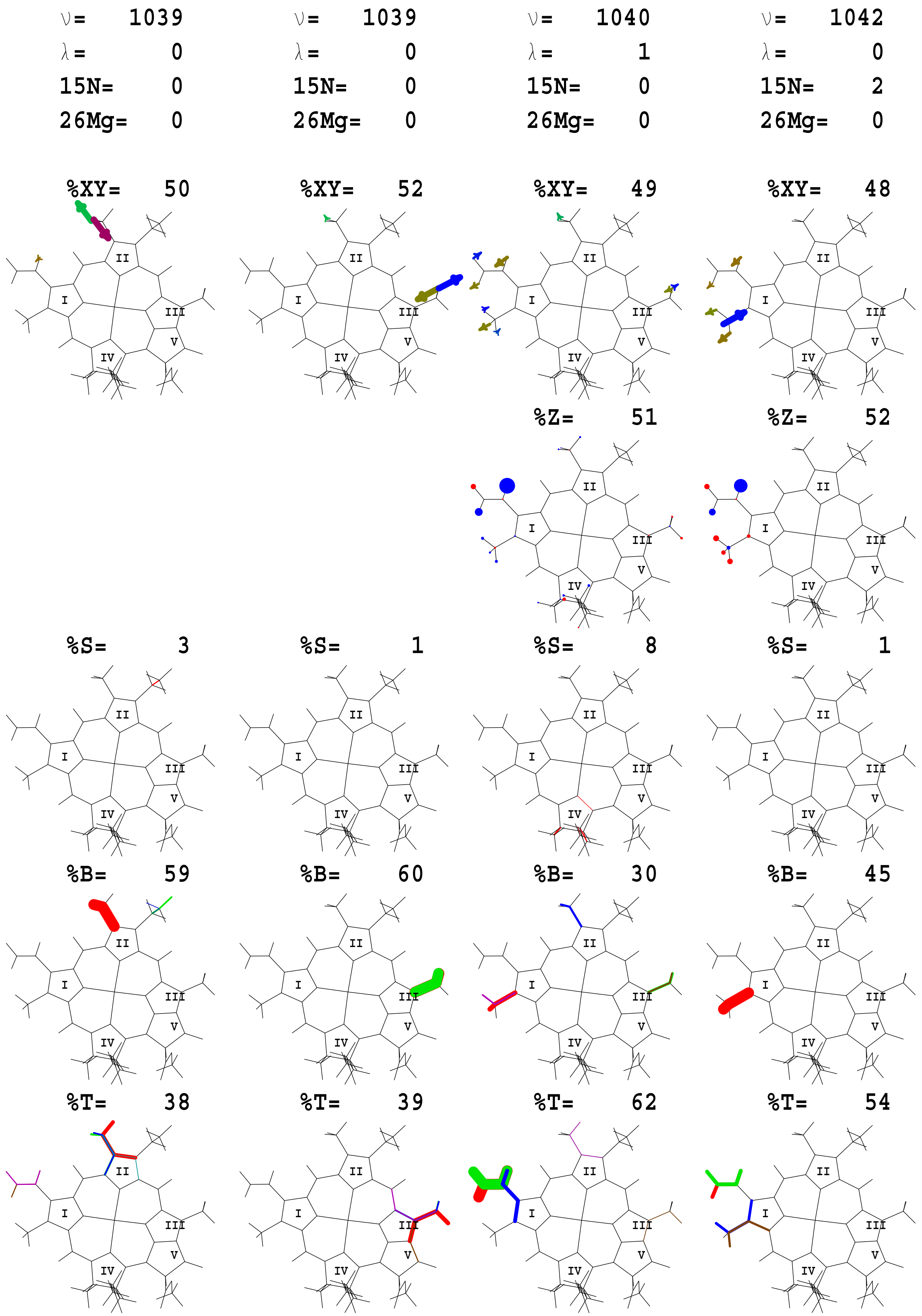
ν= 1039
λ= 0
15N= 0
26Mg= 0
%XY= 50
%S= 3
%B= 59
%T= 38
ν= 1039
λ= 0
15N= 0
26Mg= 0
%XY= 52
%S= 1
%B= 60
%T= 39
ν= 1040
λ= 1
15N= 0
26Mg= 0
%XY= 49
%Z= 51
%S= 8
%B= 30
%T= 62
ν= 1042
λ= 0
15N= 2
26Mg= 0
%XY= 48
%Z= 52
%S= 1
%B= 45
%T= 54
I
II
III
IV
V

ν=	1048	1059	1063	1074
λ=	0	0	1	0
15N=	1	1	3	2
26Mg=	0	0	0	0

%XY=	32	61	48	59

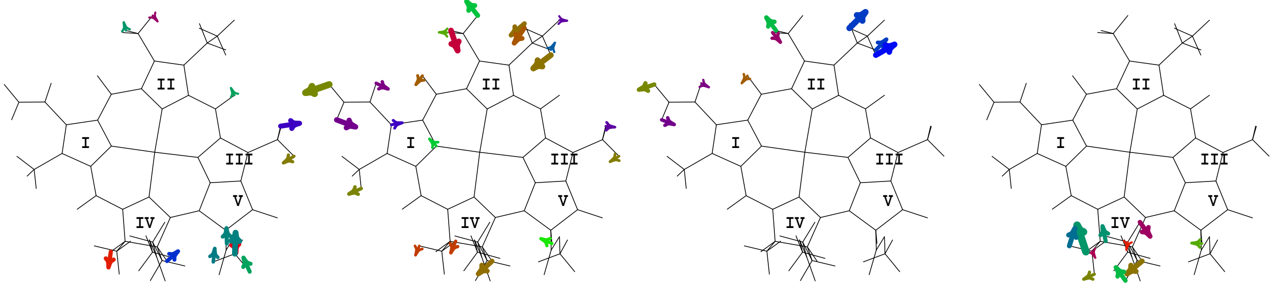

%Z=	68	39	52	41

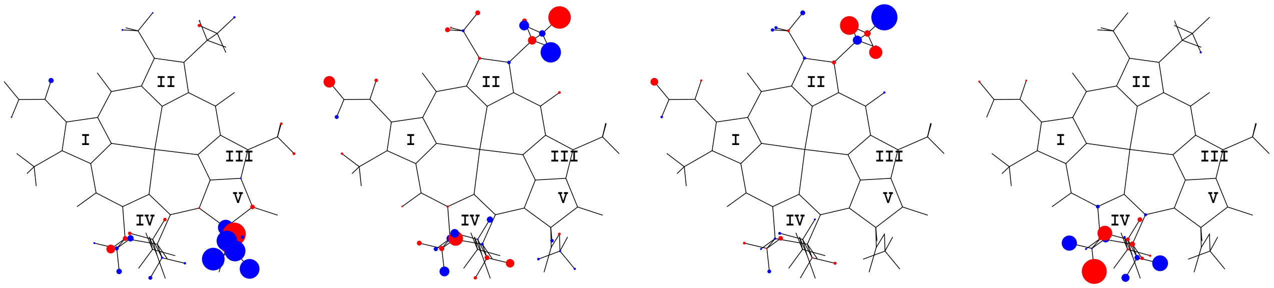

%S=	79	34	24	25

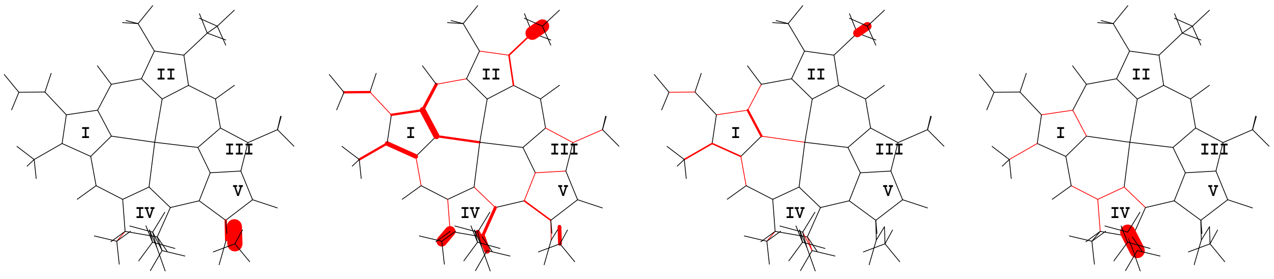

%B=	12	49	55	43

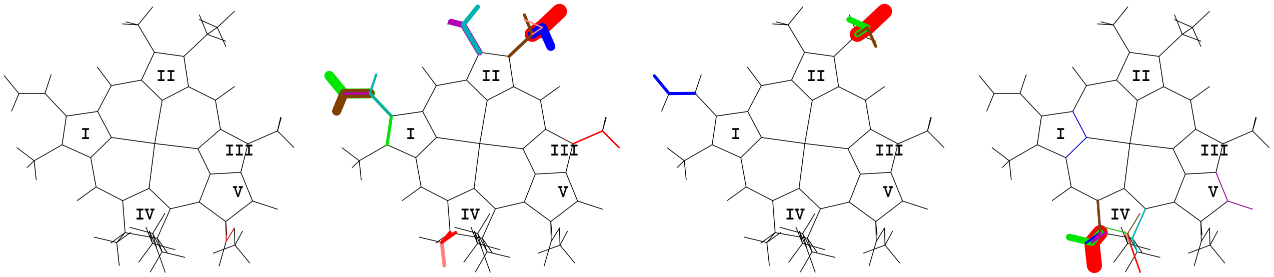

%T=	9	17	22	31

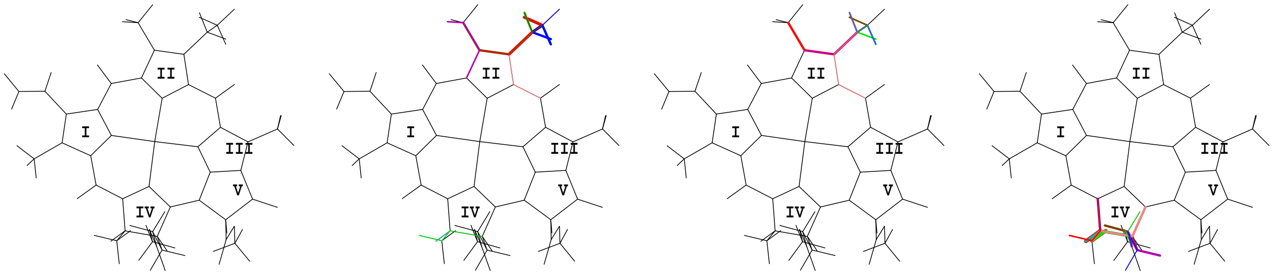

ν= 1093	ν= 1102	ν= 1110	ν= 1114
λ= 0	λ= 2	λ= 0	λ= 2
15N= 0	15N= 2	15N= 2	15N= 9
26Mg= 0	26Mg= 0	26Mg= 0	26Mg= 0
%XY= 40	%XY= 88	%XY= 78	%XY= 93
%Z= 60	%Z= 12	%Z= 22	
%S= 43	%S= 25	%S= 18	%S= 35
%B= 28	%B= 50	%B= 45	%B= 50
%T= 29	%T= 26	%T= 38	%T= 15

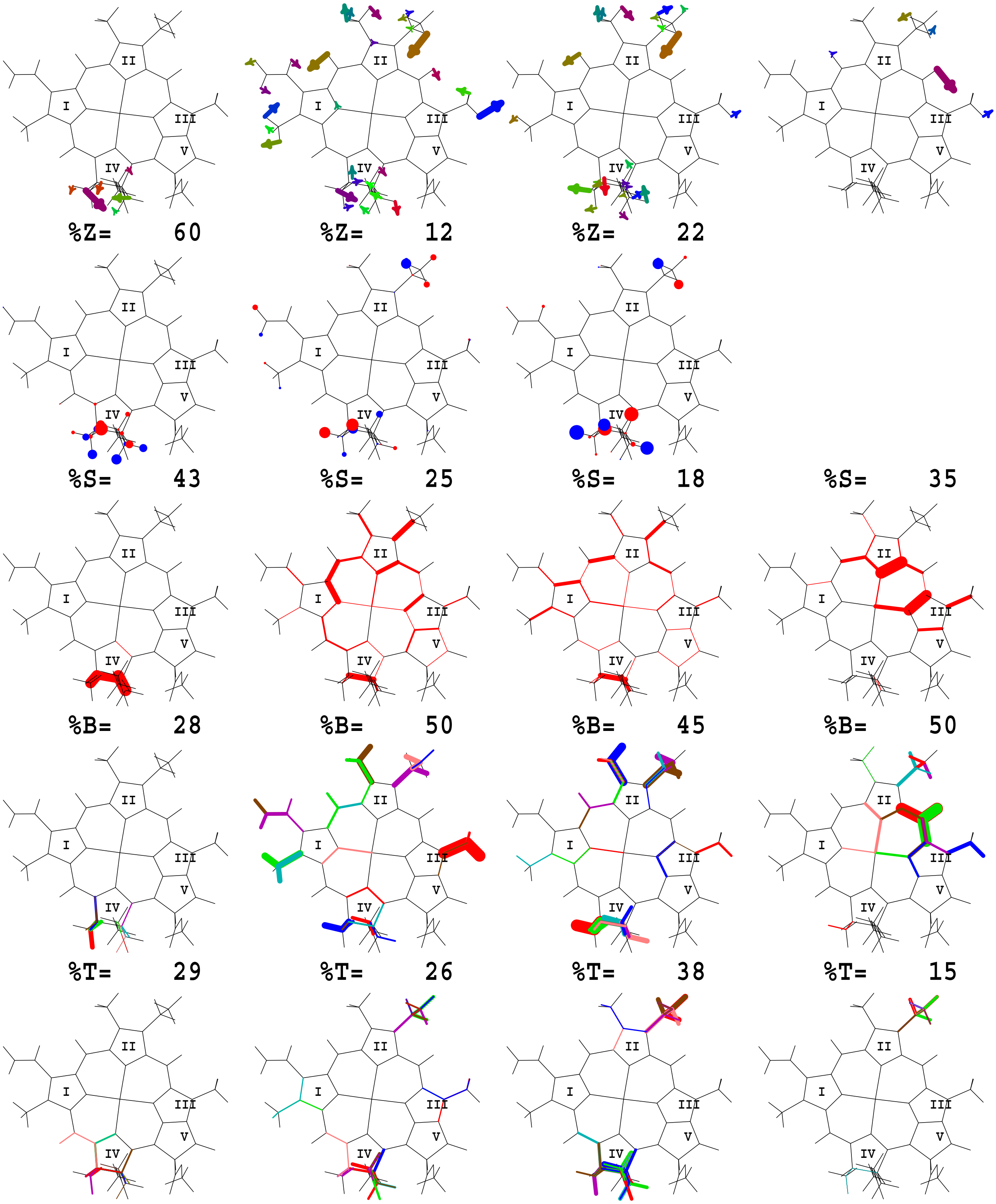

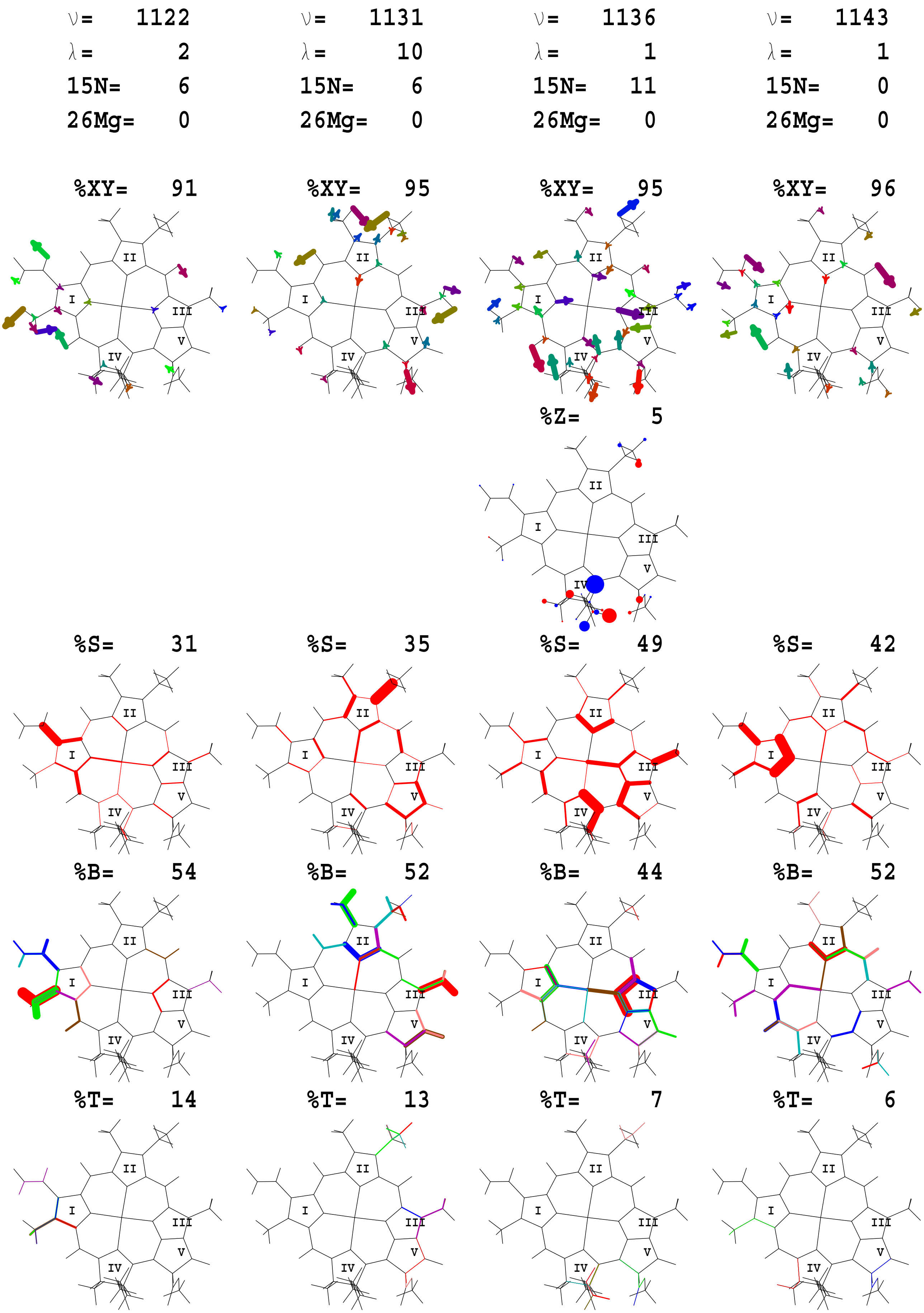
ν= 1122
λ= 2
15N= 6
26Mg= 0
%XY= 91
%S= 31
%B= 54
%T= 14
ν= 1131
λ= 10
15N= 6
26Mg= 0
%XY= 95
%S= 35
%B= 52
%T= 13
ν= 1136
λ= 1
15N= 11
26Mg= 0
%XY= 95
%Z= 5
%S= 49
%B= 44
%T= 7
ν= 1143
λ= 1
15N= 0
26Mg= 0
%XY= 96
%S= 42
%B= 52
%T= 6
I
II
III
IV
V

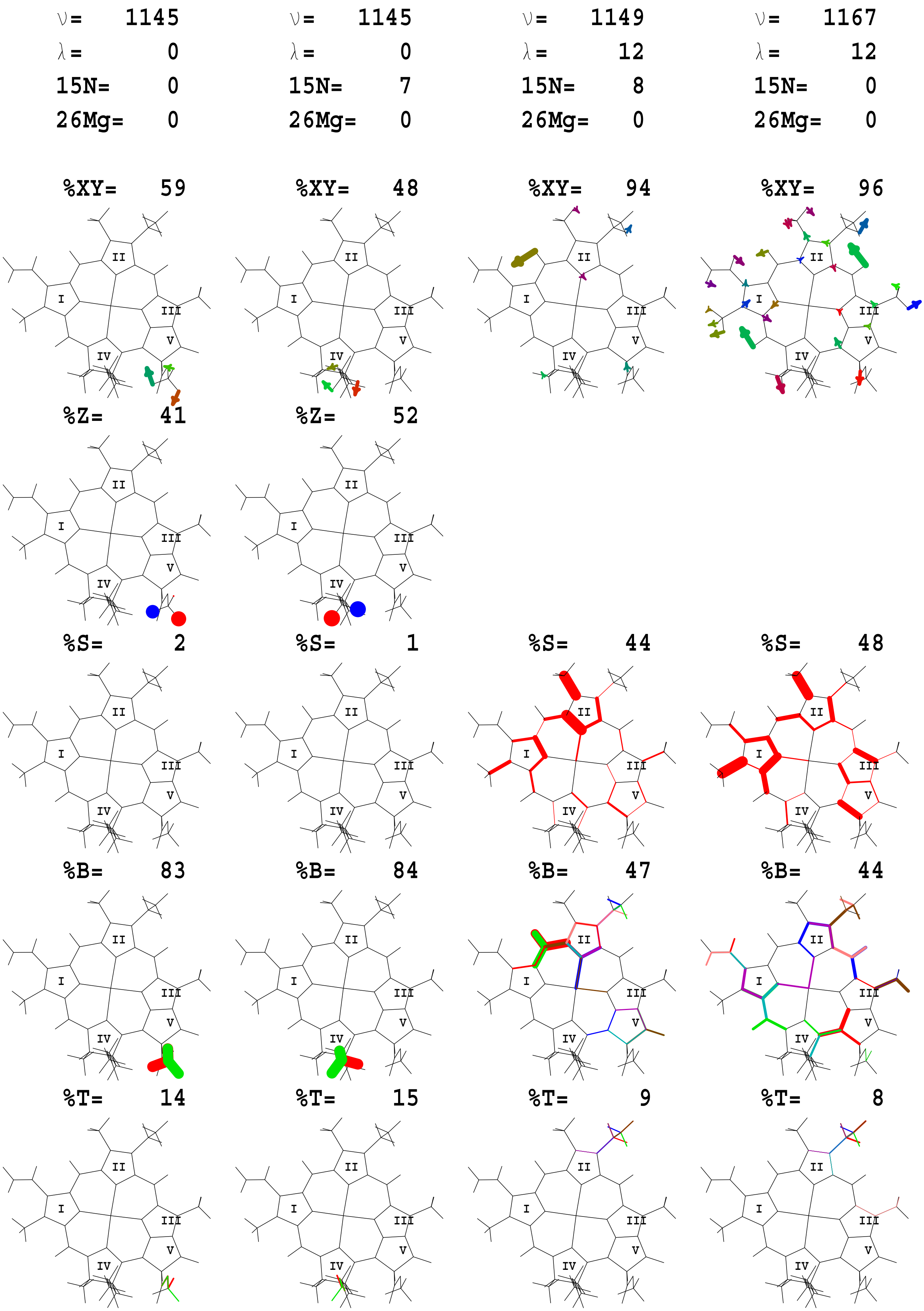
ν= 1145
λ= 0
15N= 0
26Mg= 0
%XY= 59
%Z= 41
%S= 2
%B= 83
%T= 14
ν= 1145
λ= 0
15N= 7
26Mg= 0
%XY= 48
%Z= 52
%S= 1
%B= 84
%T= 15
ν= 1149
λ= 12
15N= 8
26Mg= 0
%XY= 94
%S= 44
%B= 47
%T= 9
ν= 1167
λ= 12
15N= 0
26Mg= 0
%XY= 96
%S= 48
%B= 44
%T= 8

ν=	1174	1178	1179	1184
λ=	0	0	0	1
15N=	4	0	0	3
26Mg=	0	0	0	0

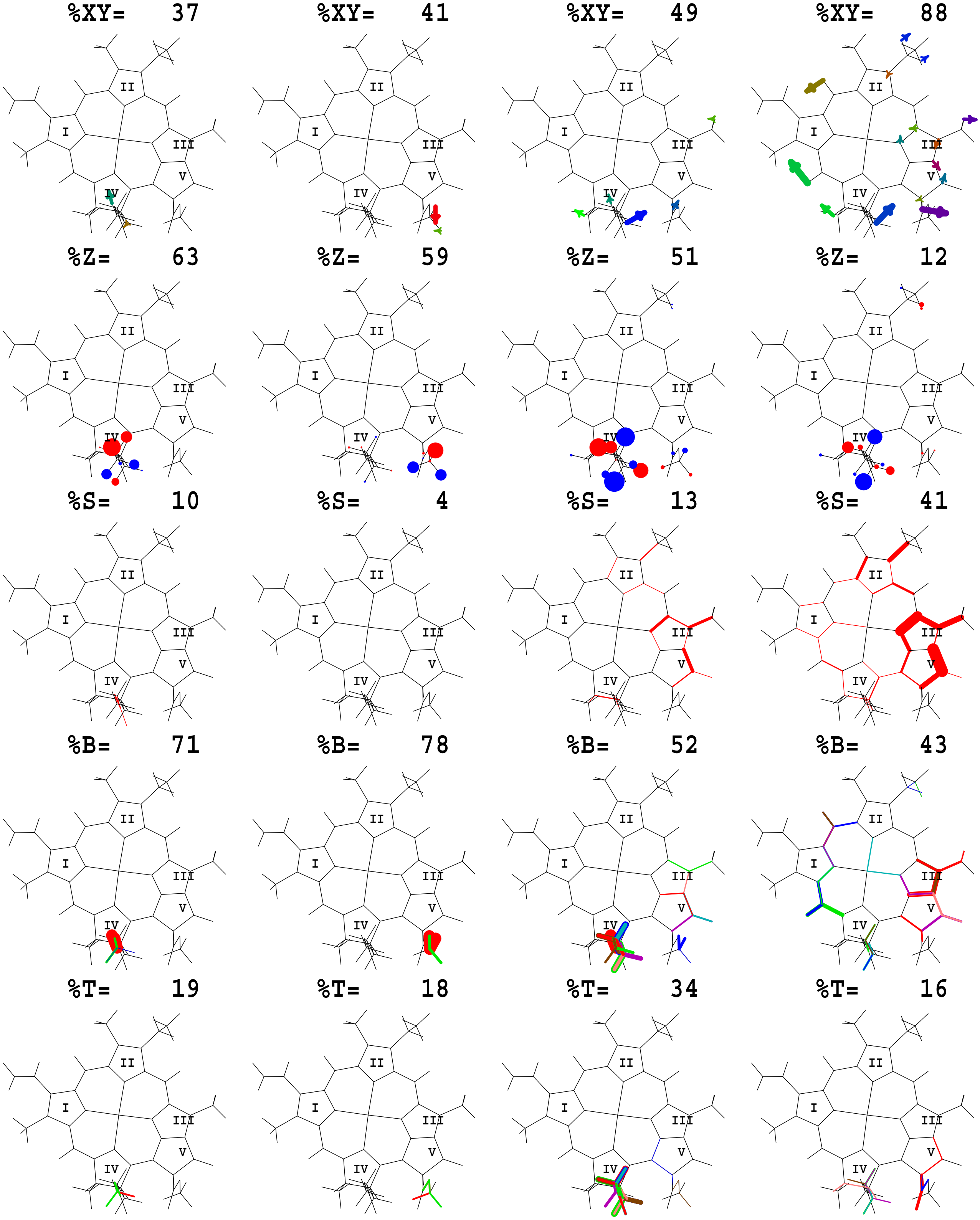

ν=	1192	1203	1211	1221
λ=	5	1	35	26
15N=	5	1	0	3
26Mg=	0	0	0	0

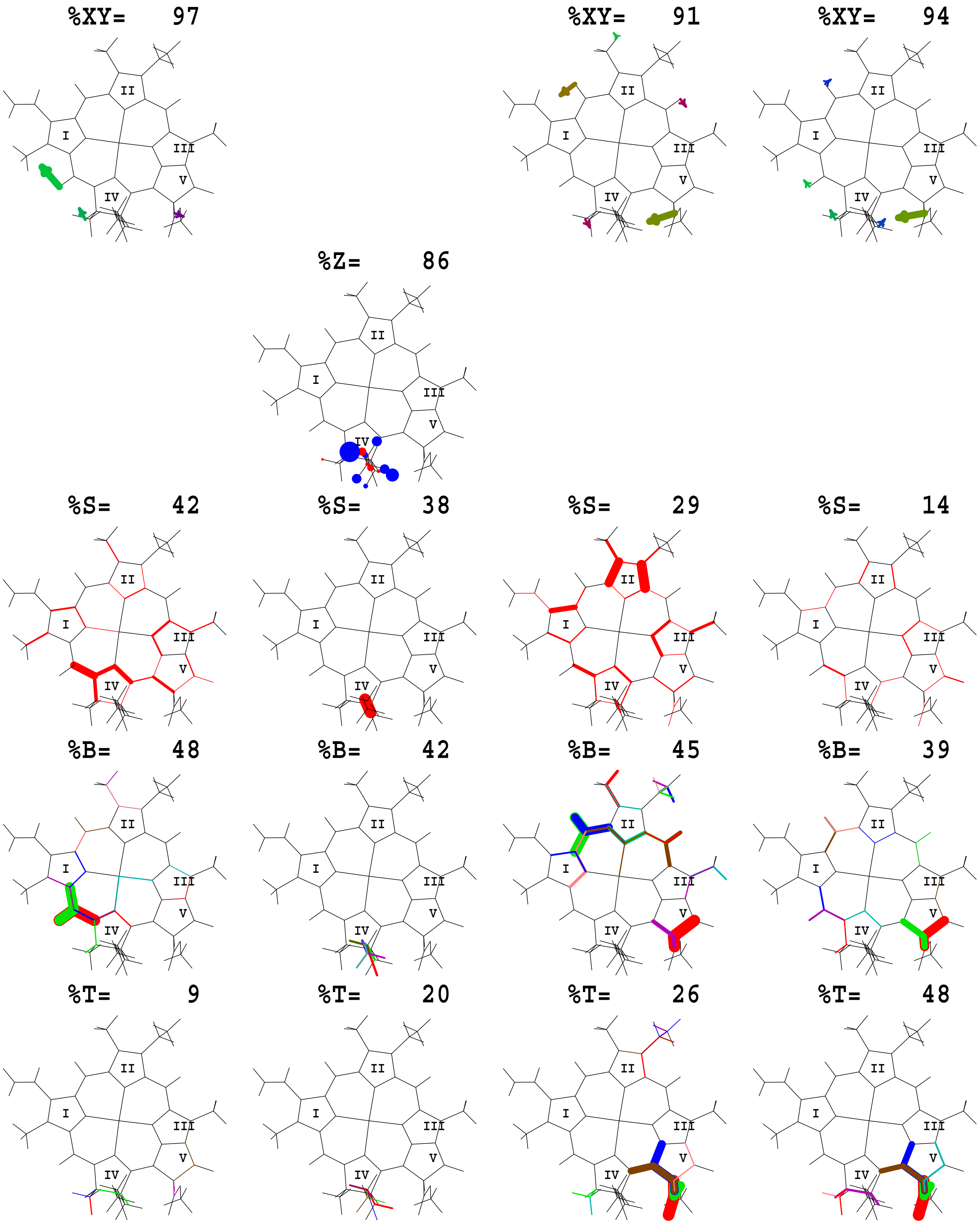

ν=	1255	1259	1262	1273
λ=	6	0	11	0
15N=	1	1	0	0
26Mg=	0	0	0	0

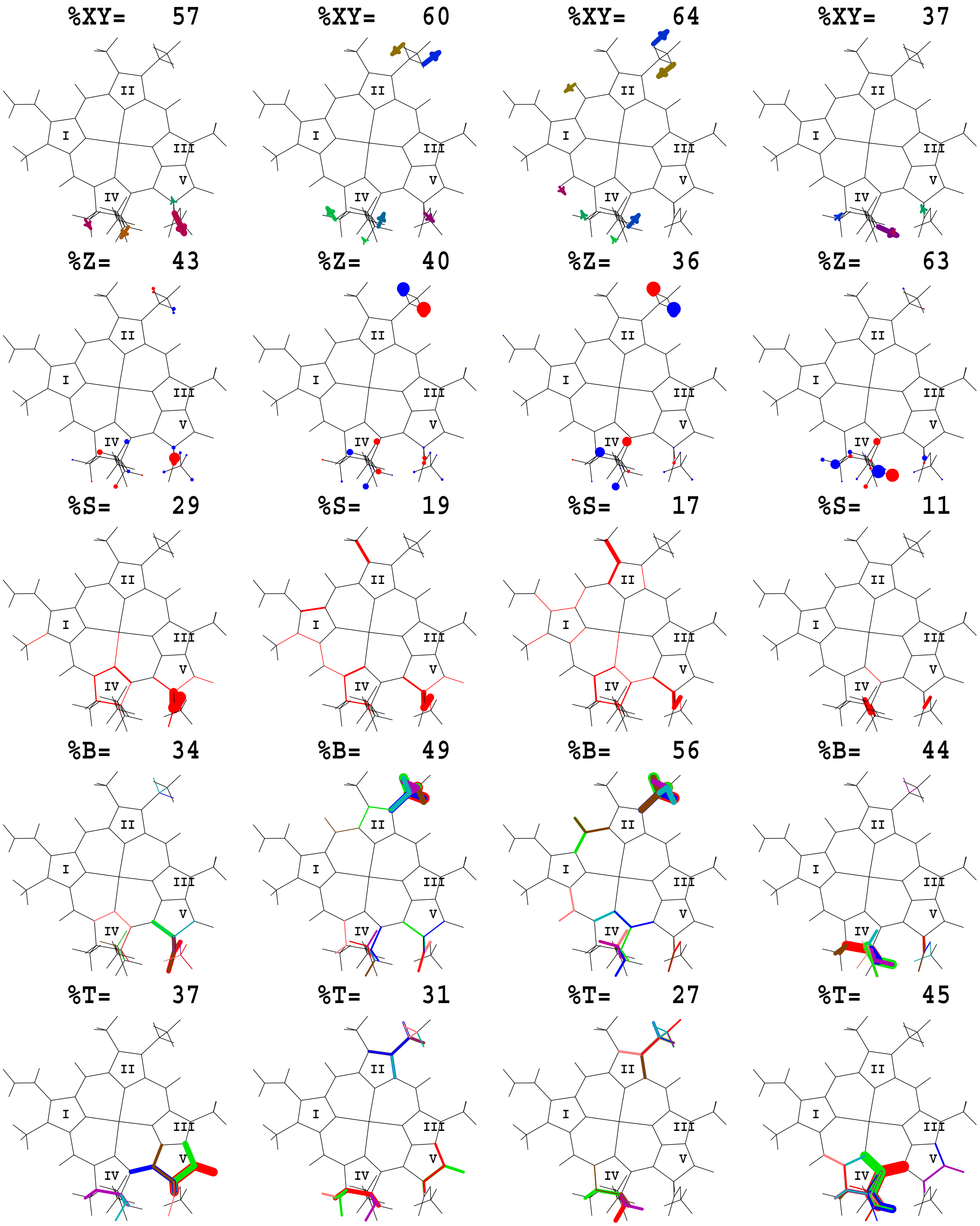

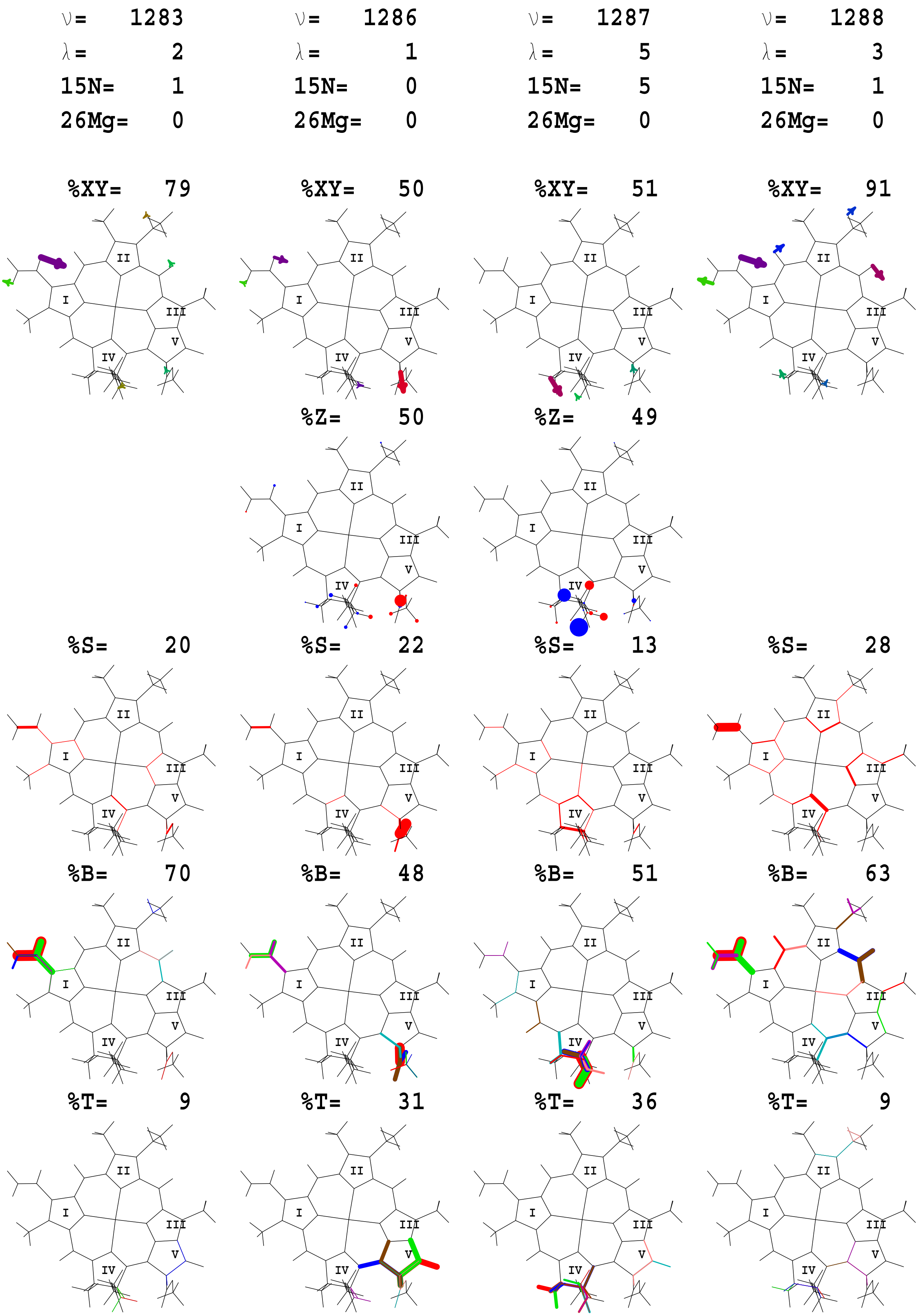

ν= 1283
λ= 2
15N= 1
26Mg= 0
ν= 1286
λ= 1
15N= 0
26Mg= 0
ν= 1287
λ= 5
15N= 5
26Mg= 0
ν= 1288
λ= 3
15N= 1
26Mg= 0
%XY= 79
%XY= 50
%XY= 51
%XY= 91
%Z= 50
%Z= 49
%S= 20
%S= 22
%S= 13
%S= 28
%B= 70
%B= 48
%B= 51
%B= 63
%T= 9
%T= 31
%T= 36
%T= 9

ν= 1312	ν= 1321	ν= 1324	ν= 1331
λ= 1	λ= 37	λ= 17	λ= 0
15N= 0	15N= 1	15N= 1	15N= 2
26Mg= 0	26Mg= 0	26Mg= 0	26Mg= 0

%XY= 50 %XY= 79 %XY= 81 %XY= 80

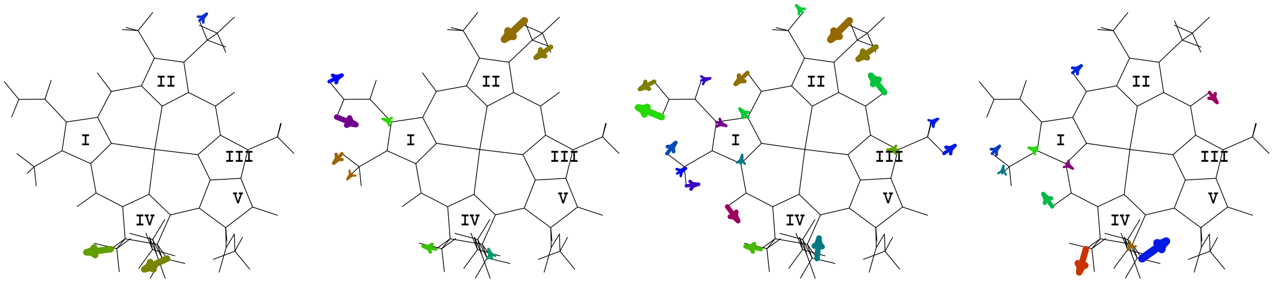

%Z= 50 %Z= 21 %Z= 19 %Z= 20

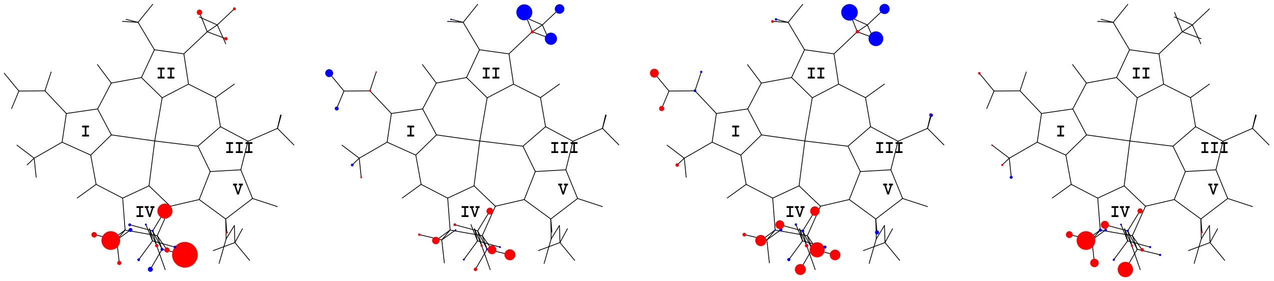

%S= 11 %S= 29 %S= 28 %S= 23

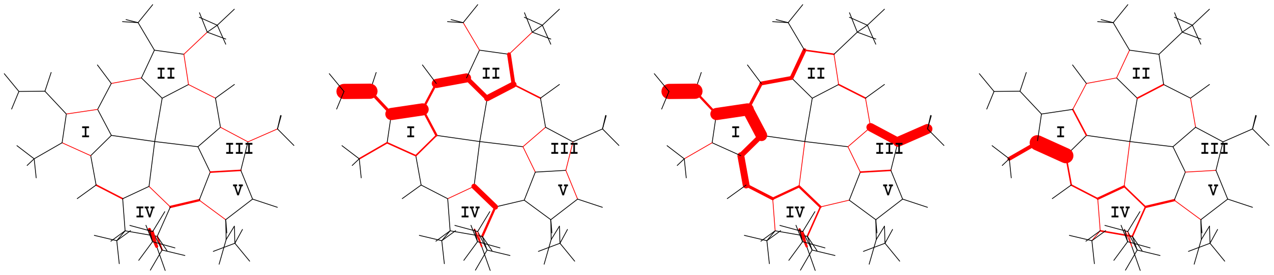

%B= 48 %B= 55 %B= 57 %B= 47

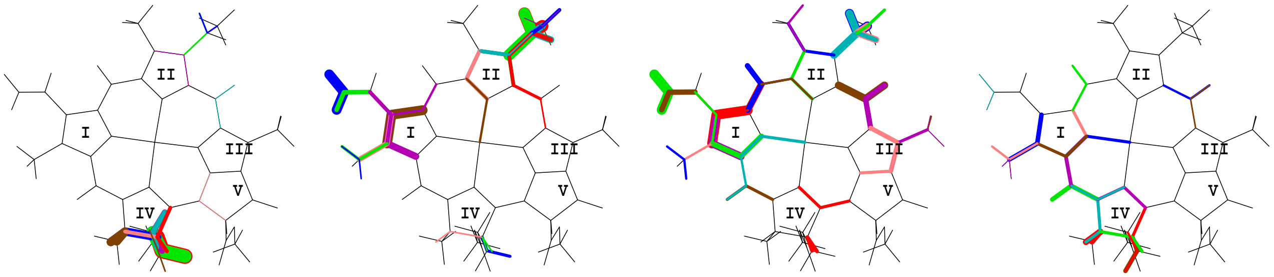

%T= 41 %T= 16 %T= 15 %T= 30

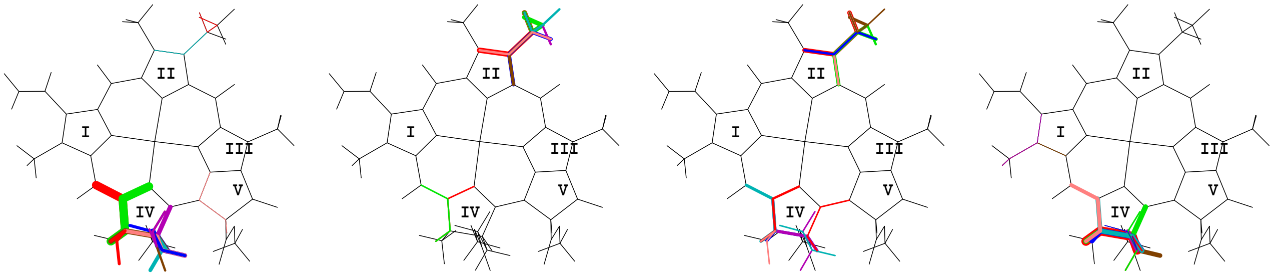

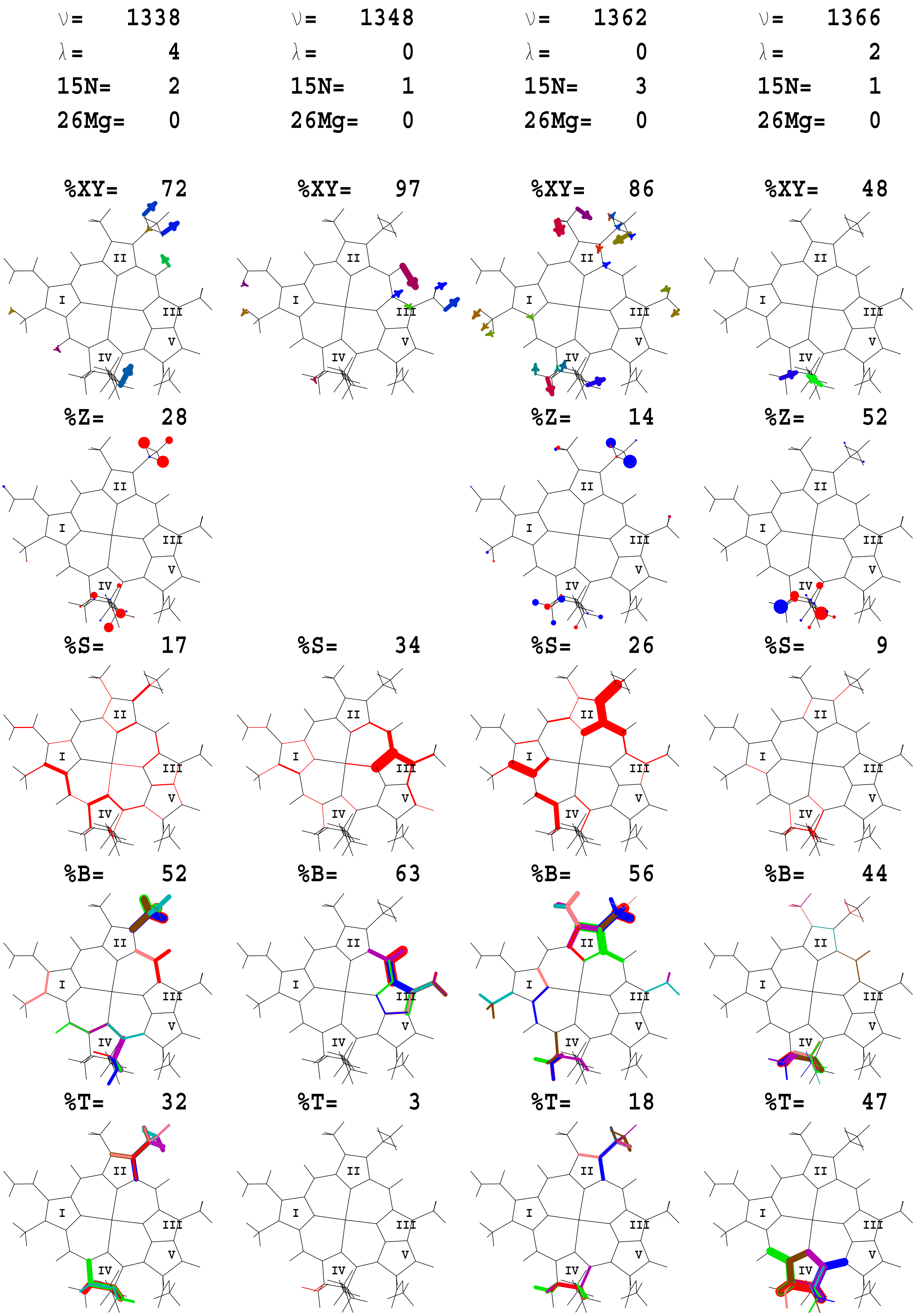

ν= 1338
λ= 4
15N= 2
26Mg= 0
%XY= 72
%Z= 28
%S= 17
%B= 52
%T= 32
ν= 1348
λ= 0
15N= 1
26Mg= 0
%XY= 97
%S= 34
%B= 63
%T= 3
ν= 1362
λ= 0
15N= 3
26Mg= 0
%XY= 86
%Z= 14
%S= 26
%B= 56
%T= 18
ν= 1366
λ= 2
15N= 1
26Mg= 0
%XY= 48
%Z= 52
%S= 9
%B= 44
%T= 47
I
II
III
IV
V

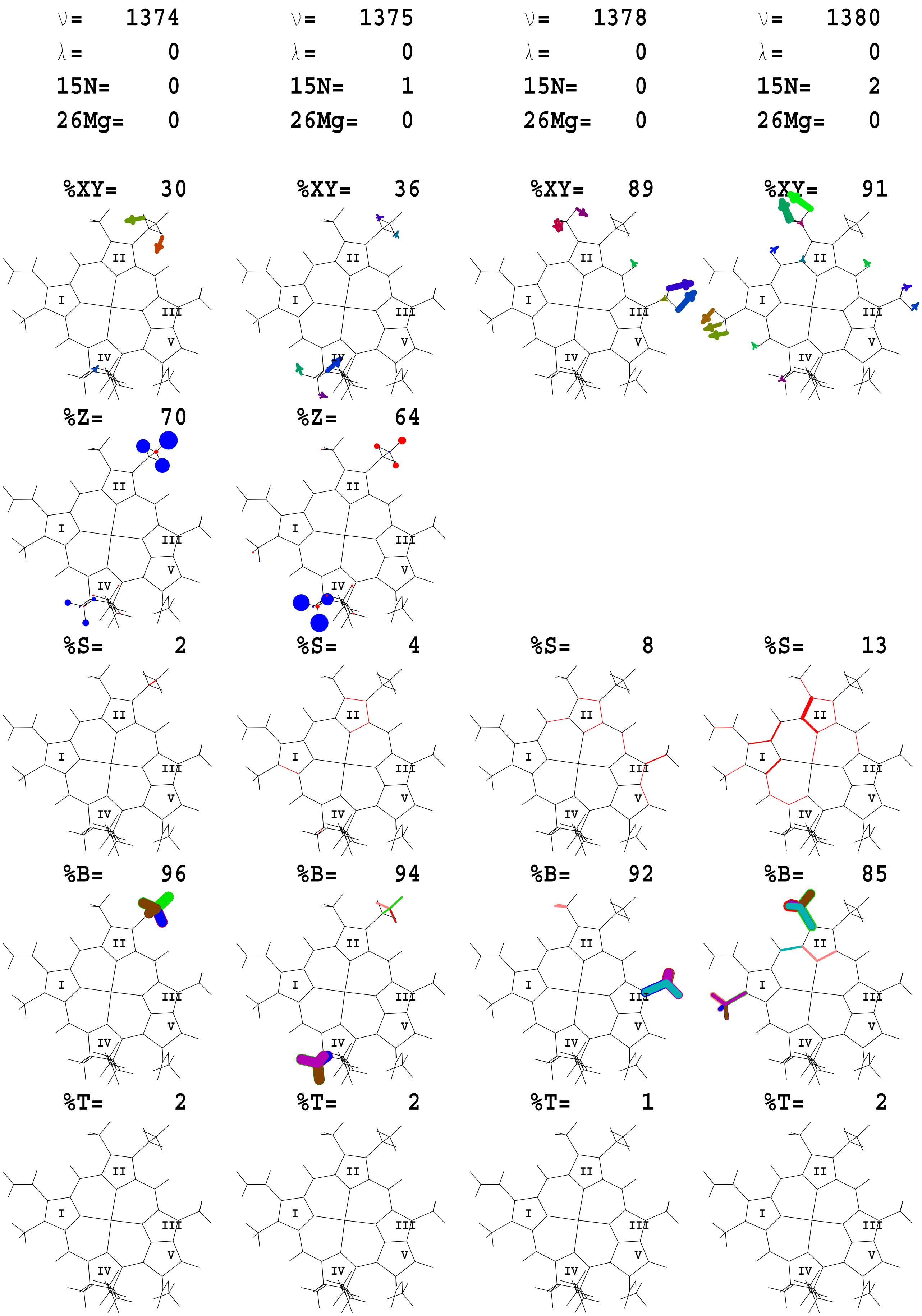
ν= 1374
λ= 0
15N= 0
26Mg= 0
ν= 1375
λ= 0
15N= 1
26Mg= 0
ν= 1378
λ= 0
15N= 0
26Mg= 0
ν= 1380
λ= 0
15N= 2
26Mg= 0
%XY= 30
%XY= 36
%XY= 89
%XY= 91
%Z= 70
%Z= 64
%S= 2
%S= 4
%S= 8
%S= 13
%B= 96
%B= 94
%B= 92
%B= 85
%T= 2
%T= 2
%T= 1
%T= 2
I
II
III
IV
V

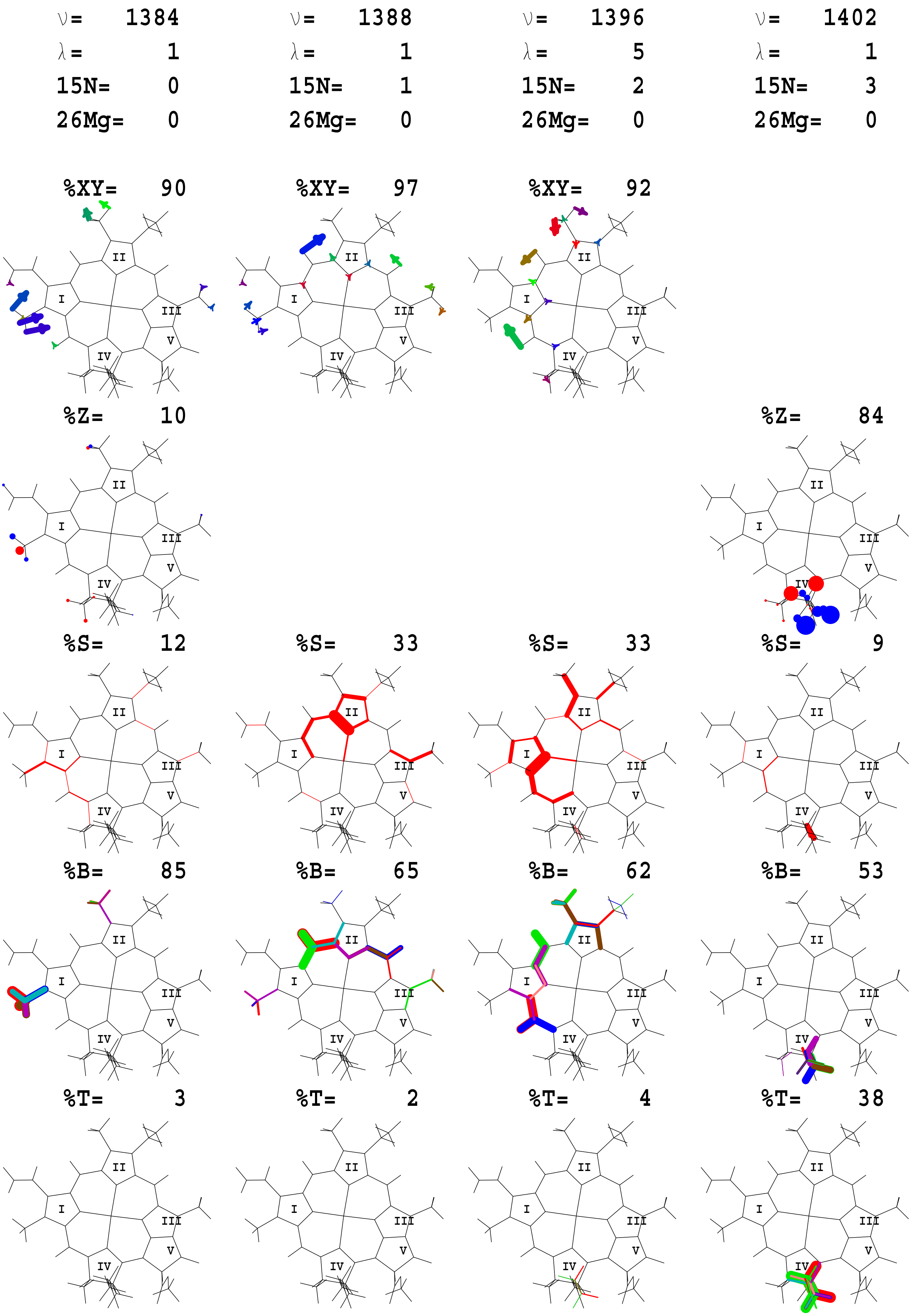

ν= 1384
λ= 1
15N= 0
26Mg= 0
ν= 1388
λ= 1
15N= 1
26Mg= 0
ν= 1396
λ= 5
15N= 2
26Mg= 0
ν= 1402
λ= 1
15N= 3
26Mg= 0
%XY= 90
%XY= 97
%XY= 92
%Z= 10
%Z= 84
%S= 12
%S= 33
%S= 33
%S= 9
%B= 85
%B= 65
%B= 62
%B= 53
%T= 3
%T= 2
%T= 4
%T= 38

ν= 1422	ν= 1429	ν= 1432	ν= 1435
λ= 5	λ= 0	λ= 0	λ= 0
15N= 1	15N= 1	15N= 0	15N= 0
26Mg= 0	26Mg= 0	26Mg= 0	26Mg= 0

%XY= 88	%XY= 78	%XY= 49	%XY= 18

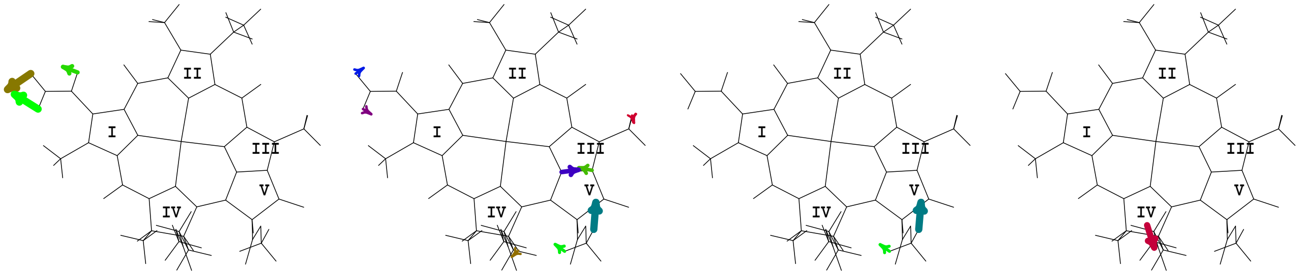

%Z= 12	%Z= 22	%Z= 51	%Z= 82

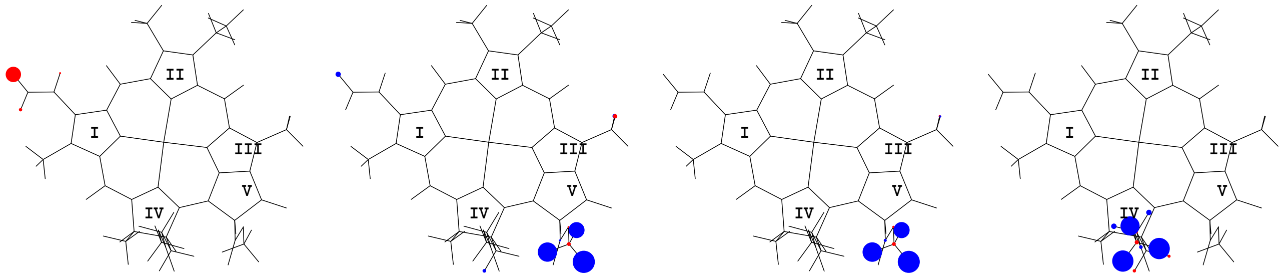

%S= 18	%S= 34	%S= 18	%S= 8

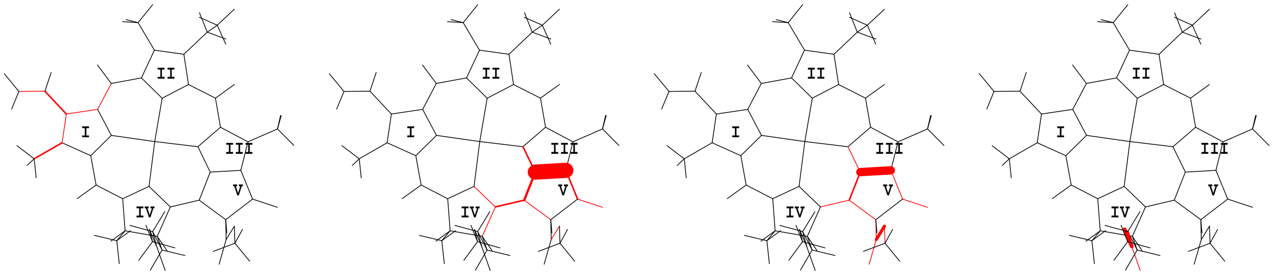

%B= 82	%B= 64	%B= 80	%B= 87

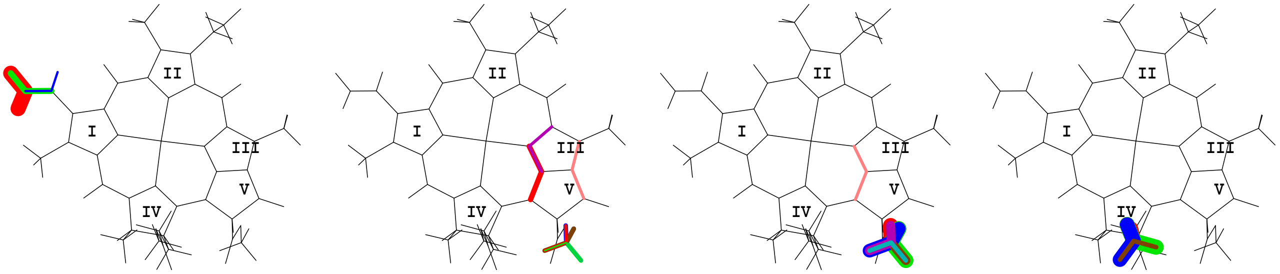

%T= 1	%T= 3	%T= 2	%T= 4

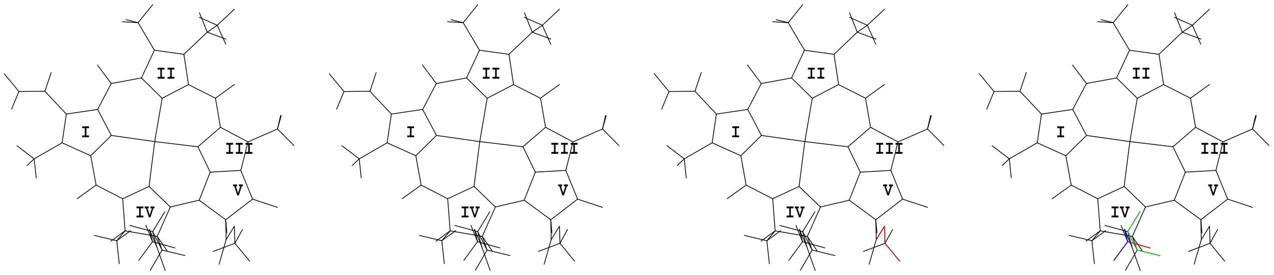

ν=	1441	1444	1458	1459
λ=	5	0	5	0
15N=	0	2	1	0
26Mg=	0	0	0	0

%XY= 97 | %XY= 95 | %XY= 96 | %XY= 63

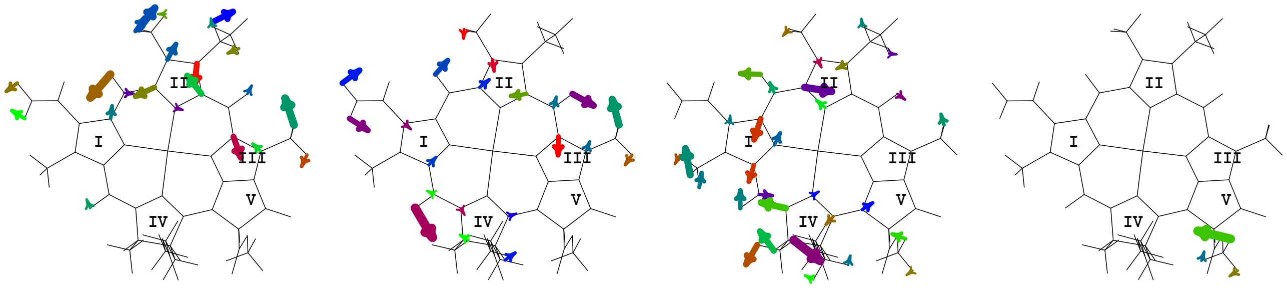

%Z= 3 | %Z= 5 | %Z= 4 | %Z= 37

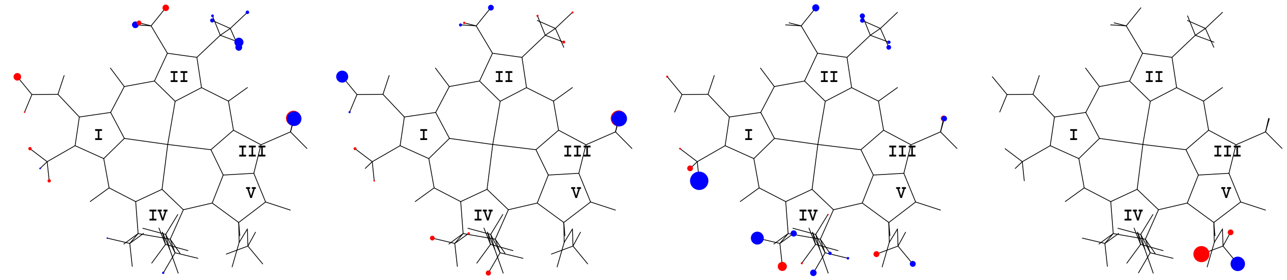

%S= 44 | %S= 44 | %S= 49 | %S= 0

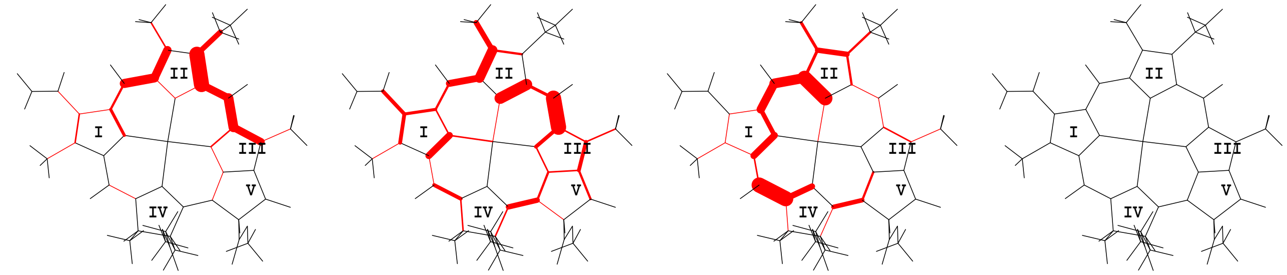

%B= 49 | %B= 50 | %B= 43 | %B= 76

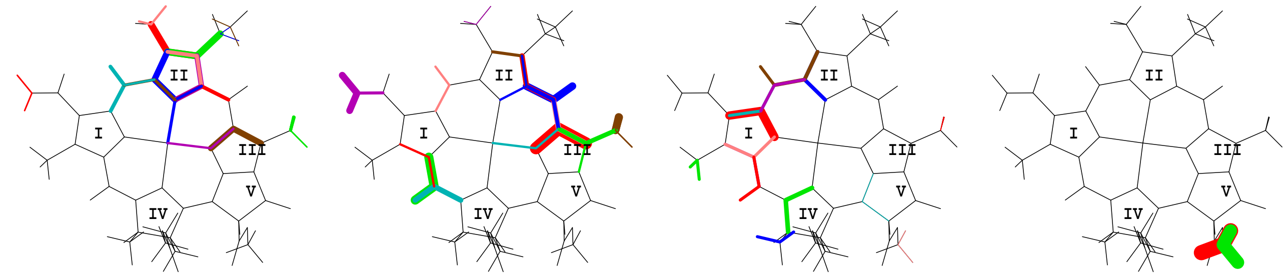

%T= 7 | %T= 6 | %T= 9 | %T= 24

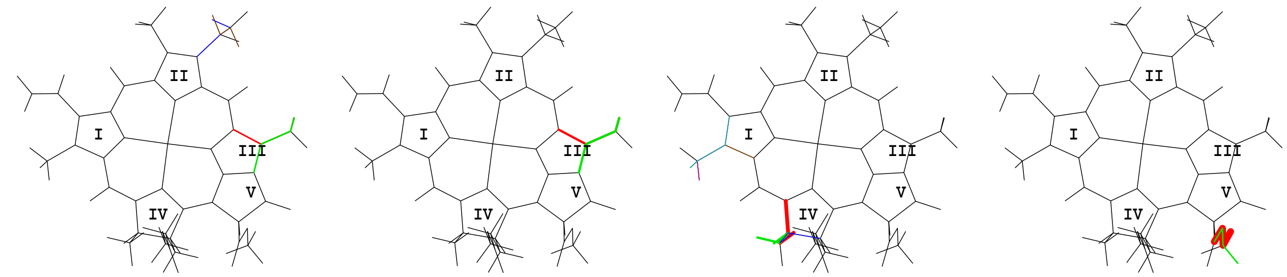

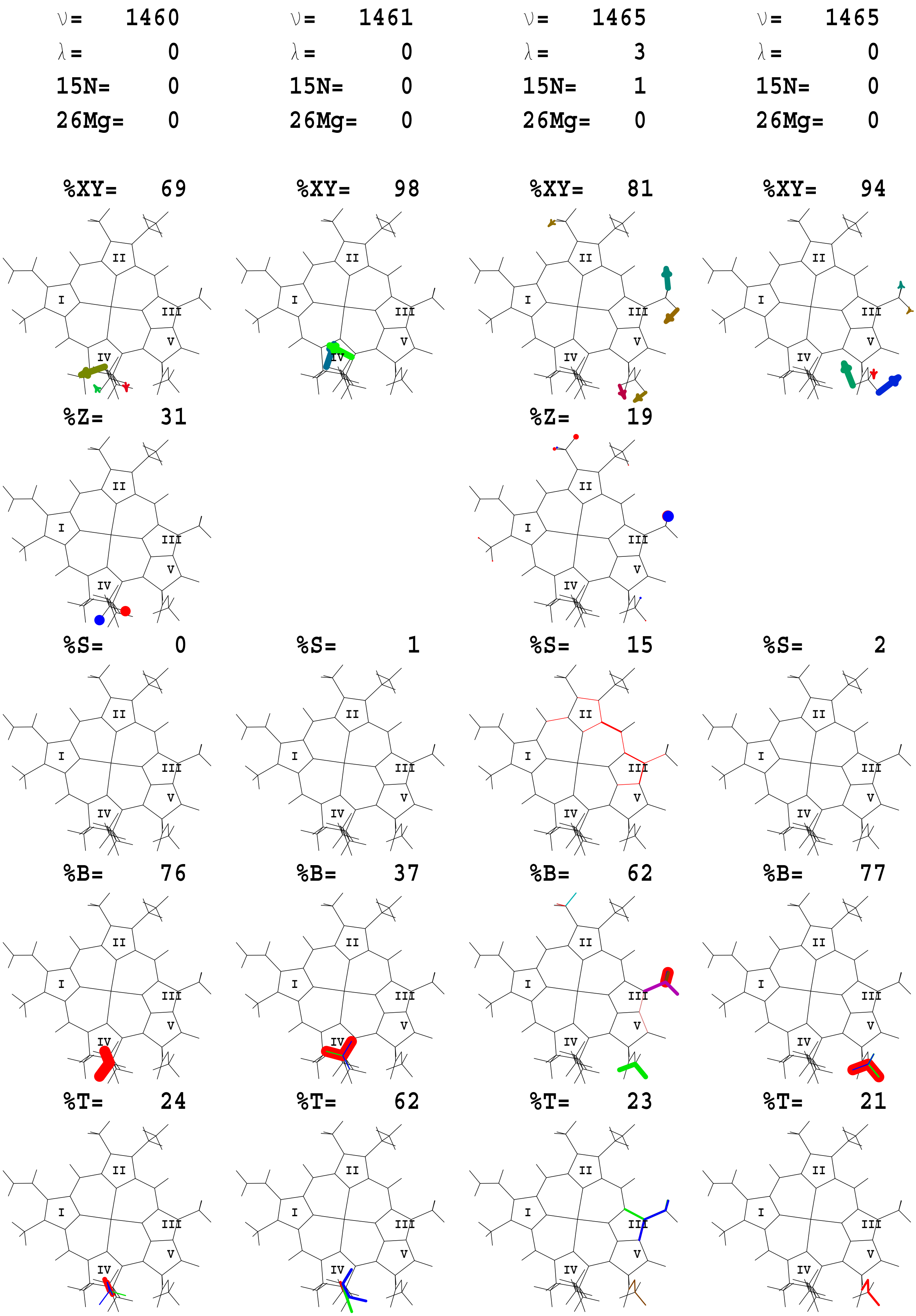

ν= 1460
λ= 0
15N= 0
26Mg= 0
%XY= 69
%Z= 31
%S= 0
%B= 76
%T= 24
ν= 1461
λ= 0
15N= 0
26Mg= 0
%XY= 98
%S= 1
%B= 37
%T= 62
ν= 1465
λ= 3
15N= 1
26Mg= 0
%XY= 81
%Z= 19
%S= 15
%B= 62
%T= 23
ν= 1465
λ= 0
15N= 0
26Mg= 0
%XY= 94
%S= 2
%B= 77
%T= 21
I
II
III
IV
V

ν= 1467
λ= 0
15N= 0
26Mg= 0

%XY= 48

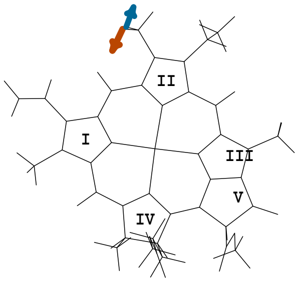

%Z= 52

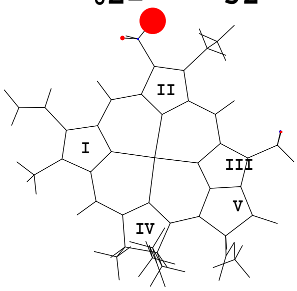

%S= 2

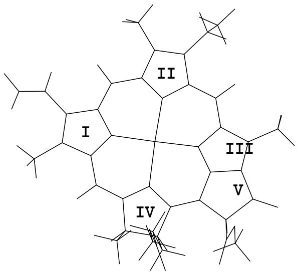

%B= 64

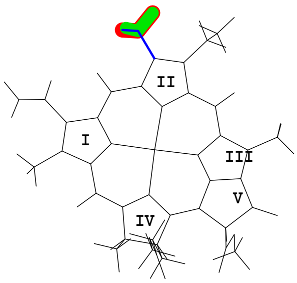

%T= 35

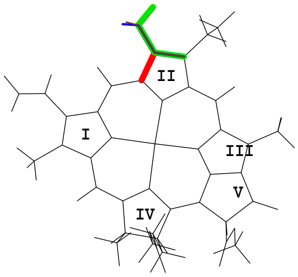

ν= 1468
λ= 0
15N= 0
26Mg= 0

%XY= 95

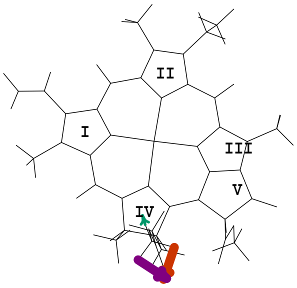

%S= 0

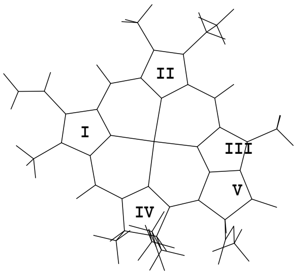

%B= 80

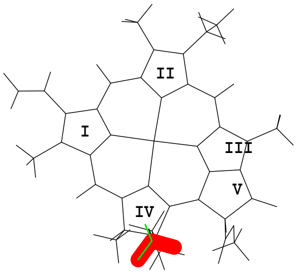

%T= 20

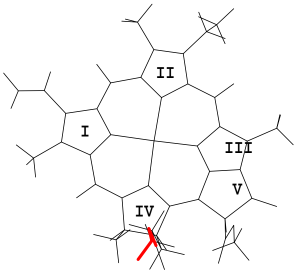

ν= 1470
λ= 0
15N= 1
26Mg= 0

%XY= 43

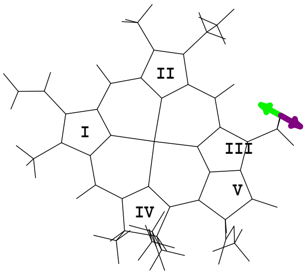

%Z= 57

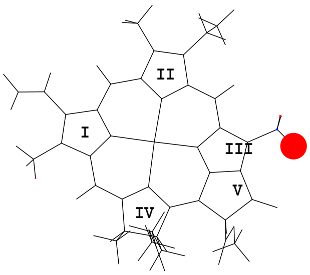

%S= 0

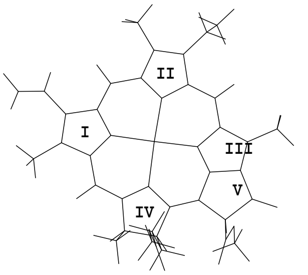

%B= 64

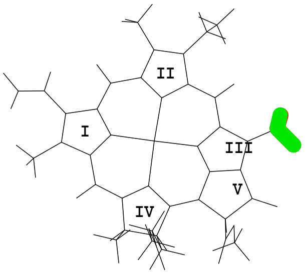

%T= 36

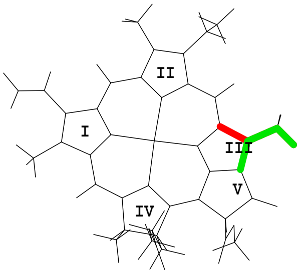

ν= 1470
λ= 2
15N= 0
26Mg= 0

%XY= 56

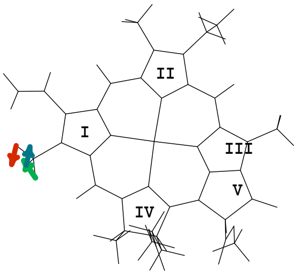

%Z= 44

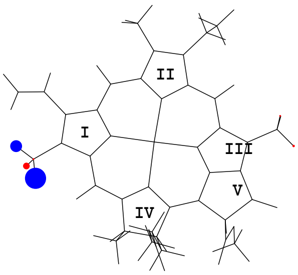

%S= 4

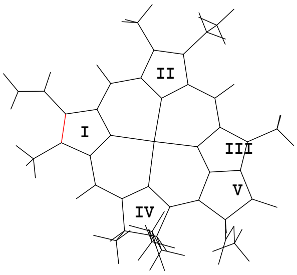

%B= 62

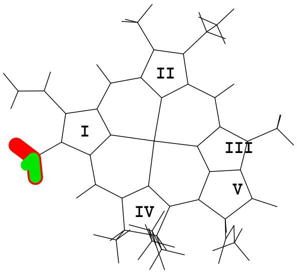

%T= 34

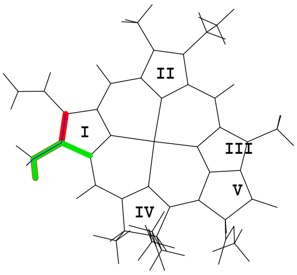

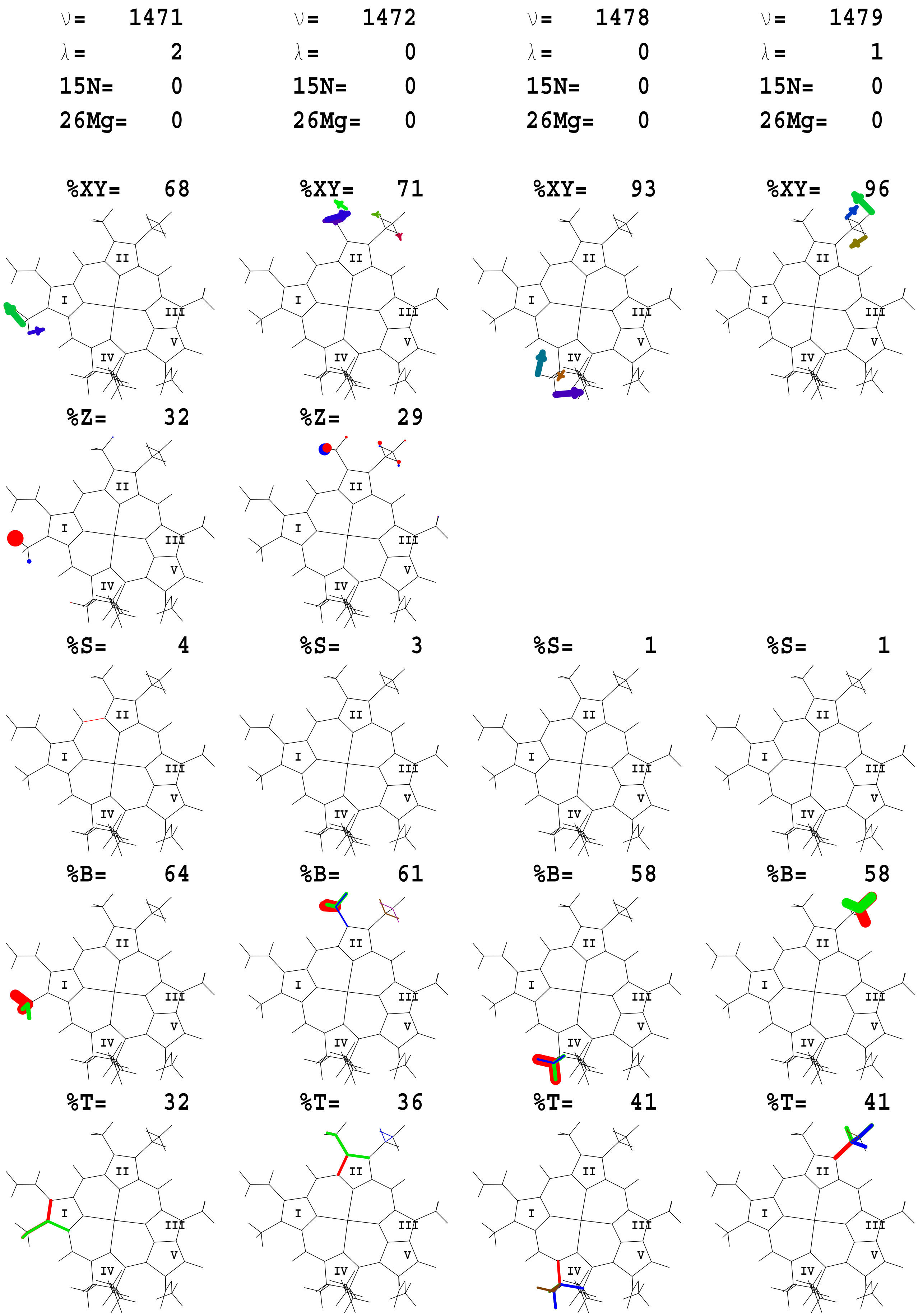
ν= 1471
λ= 2
15N= 0
26Mg= 0
ν= 1472
λ= 0
15N= 0
26Mg= 0
ν= 1478
λ= 0
15N= 0
26Mg= 0
ν= 1479
λ= 1
15N= 0
26Mg= 0
%XY= 68
%XY= 71
%XY= 93
%XY= 96
%Z= 32
%Z= 29
%S= 4
%S= 3
%S= 1
%S= 1
%B= 64
%B= 61
%B= 58
%B= 58
%T= 32
%T= 36
%T= 41
%T= 41
I
II
III
IV
V

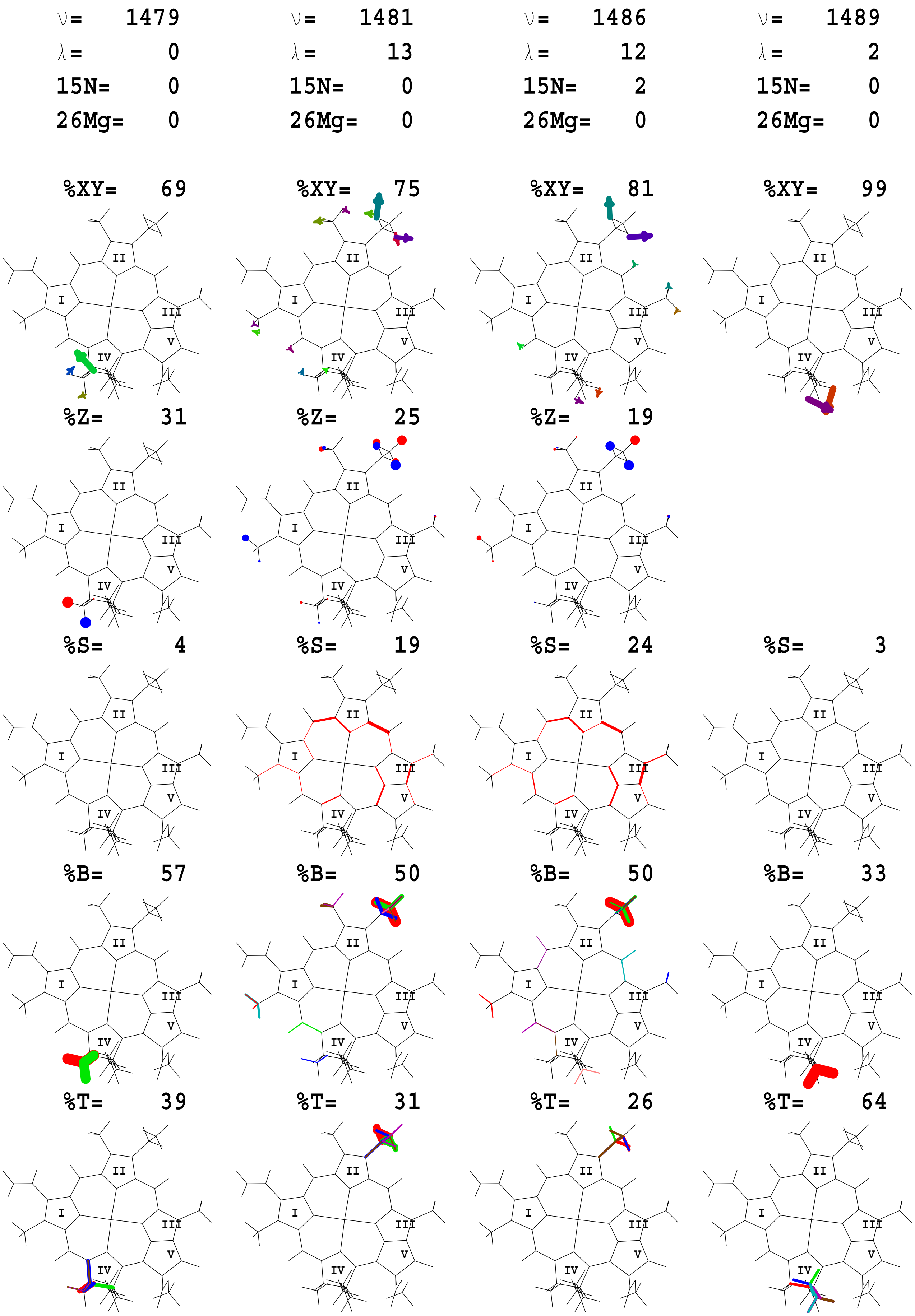

ν= 1479
λ= 0
15N= 0
26Mg= 0
%XY= 69
%Z= 31
%S= 4
%B= 57
%T= 39
ν= 1481
λ= 13
15N= 0
26Mg= 0
%XY= 75
%Z= 25
%S= 19
%B= 50
%T= 31
ν= 1486
λ= 12
15N= 2
26Mg= 0
%XY= 81
%Z= 19
%S= 24
%B= 50
%T= 26
ν= 1489
λ= 2
15N= 0
26Mg= 0
%XY= 99
%S= 3
%B= 33
%T= 64
I
II
III
IV
V

ν= 1501	ν= 1522	ν= 1538	ν= 1562
λ= 2	λ= 0	λ= 26	λ= 1
15N= 2	15N= 3	15N= 1	15N= 3
26Mg= 0	26Mg= 0	26Mg= 0	26Mg= 0

%XY= 70 %XY= 99 %XY= 99 %XY= 100

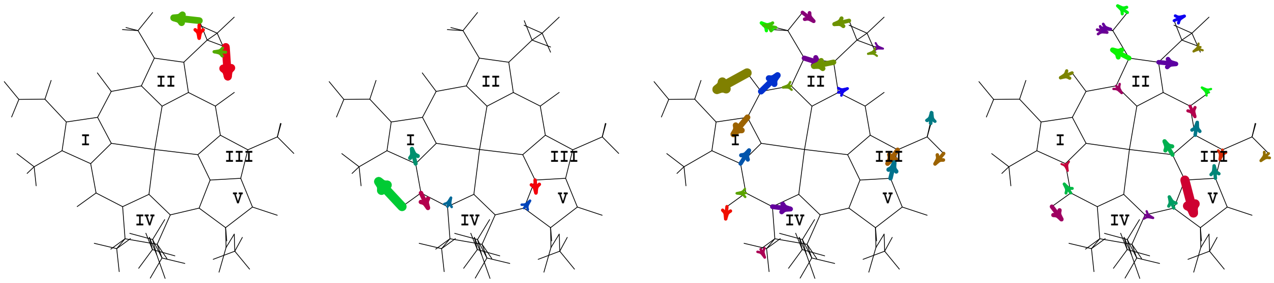

%Z= 30

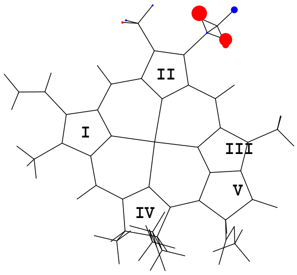

%S= 5 %S= 56 %S= 55 %S= 50

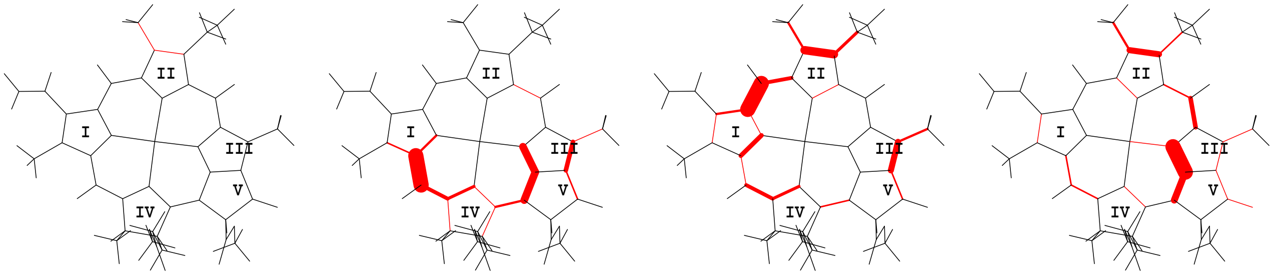

%B= 42 %B= 42 %B= 43 %B= 49

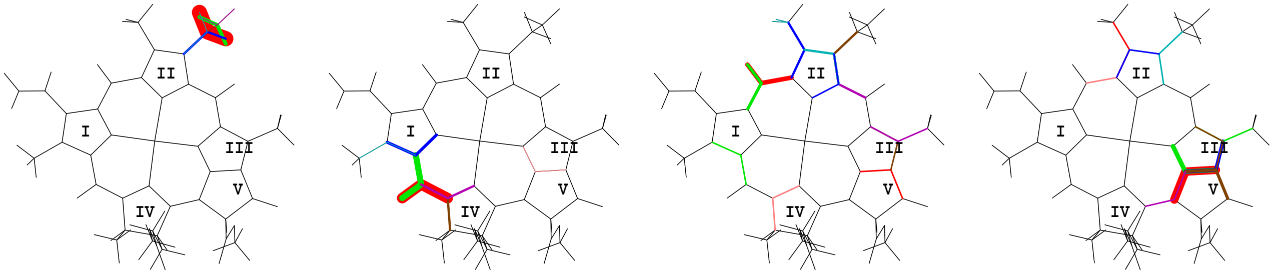

%T= 54 %T= 2 %T= 2 %T= 1

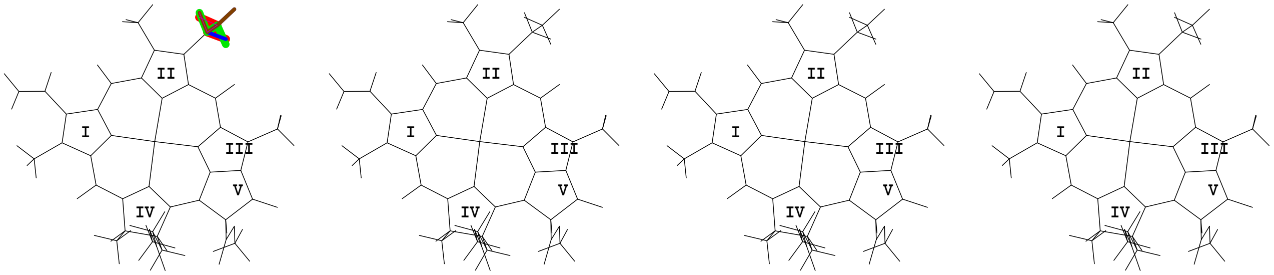

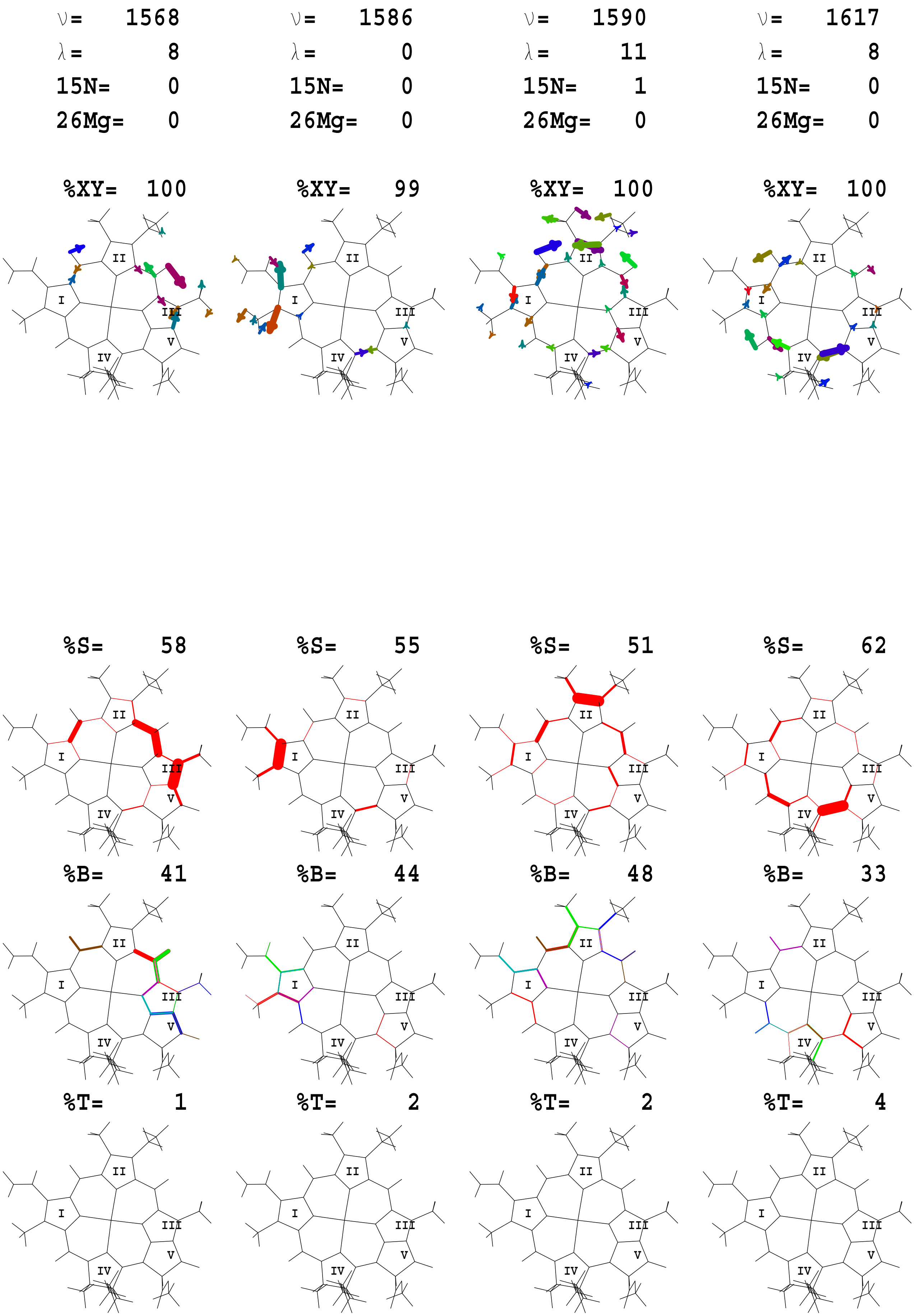

ν= 1568
λ= 8
15N= 0
26Mg= 0
%XY= 100
%S= 58
%B= 41
%T= 1
ν= 1586
λ= 0
15N= 0
26Mg= 0
%XY= 99
%S= 55
%B= 44
%T= 2
ν= 1590
λ= 11
15N= 1
26Mg= 0
%XY= 100
%S= 51
%B= 48
%T= 2
ν= 1617
λ= 8
15N= 0
26Mg= 0
%XY= 100
%S= 62
%B= 33
%T= 4

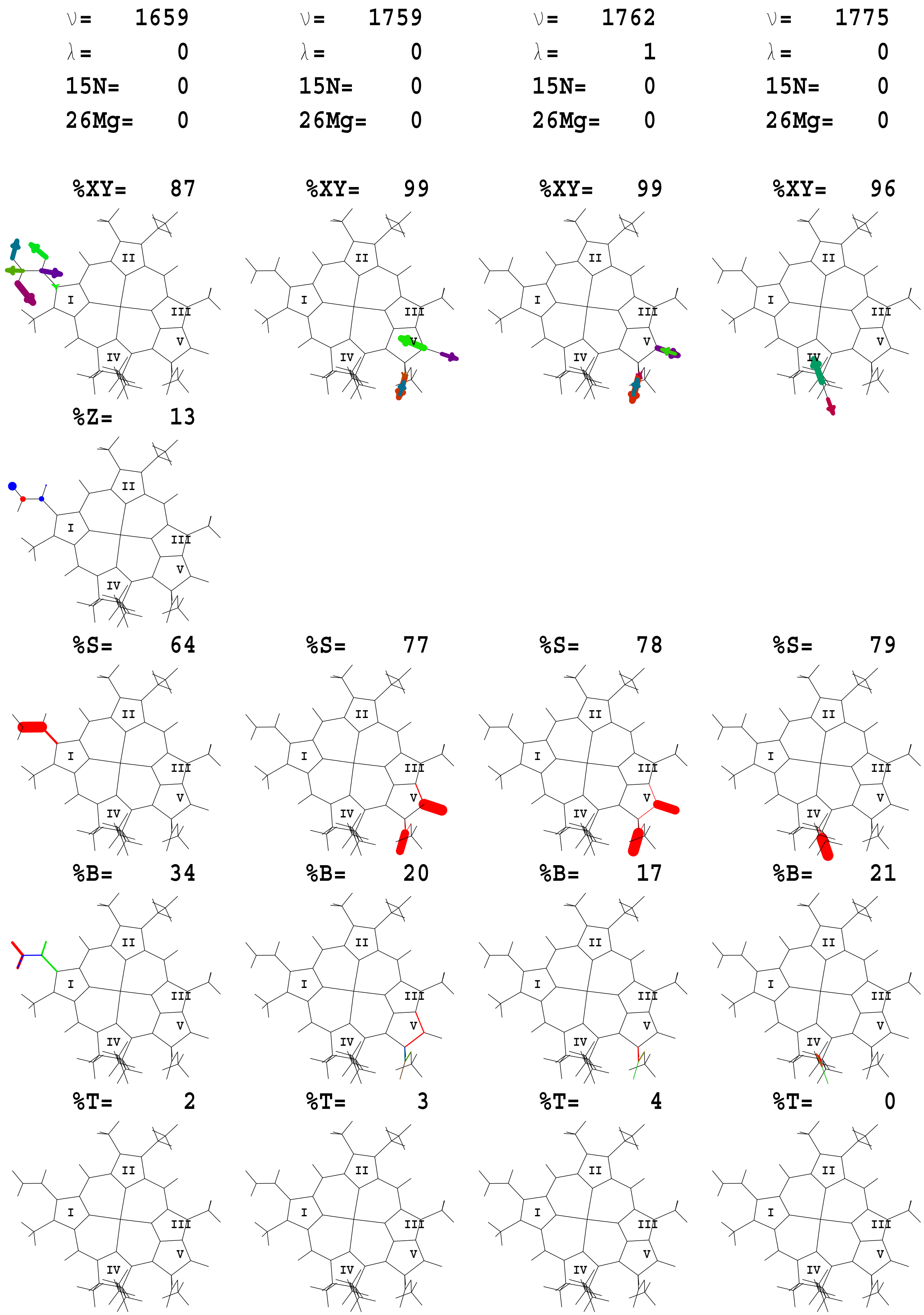

ν= 1659
λ= 0
15N= 0
26Mg= 0
%XY= 87
%Z= 13
%S= 64
%B= 34
%T= 2
ν= 1759
λ= 0
15N= 0
26Mg= 0
%XY= 99
%S= 77
%B= 20
%T= 3
ν= 1762
λ= 1
15N= 0
26Mg= 0
%XY= 99
%S= 78
%B= 17
%T= 4
ν= 1775
λ= 0
15N= 0
26Mg= 0
%XY= 96
%S= 79
%B= 21
%T= 0
I
II
III
IV
V

ν= 2916
λ= 0
15N= 0
26Mg= 0

%XY= 39

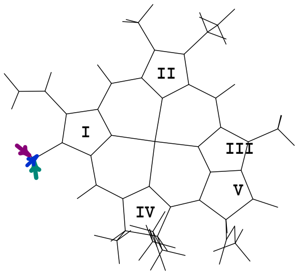

%Z= 61

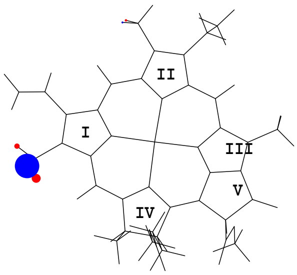

%S= 100

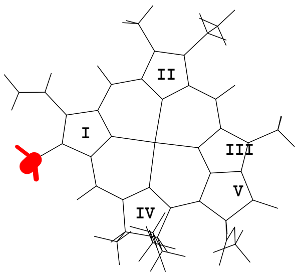

%B= 0

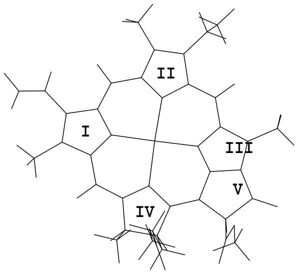

%T= 0

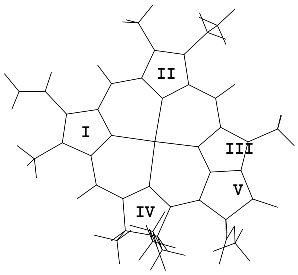

ν= 2916
λ= 0
15N= 0
26Mg= 0

%XY= 46

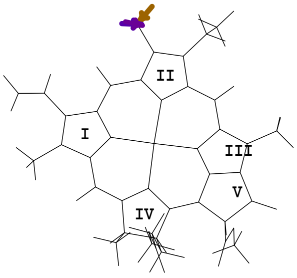

%Z= 54

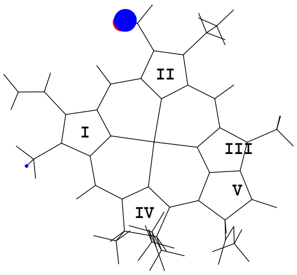

%S= 100

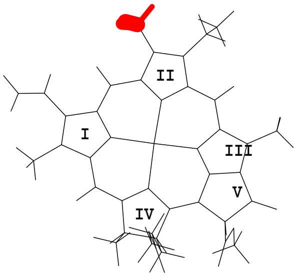

%B= 0

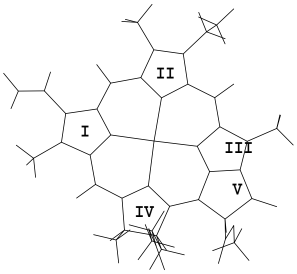

%T= 0

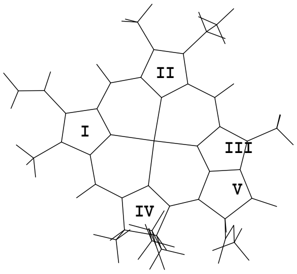

ν= 2921
λ= 0
15N= 0
26Mg= 0

%XY= 45

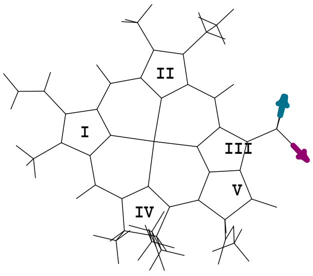

%Z= 55

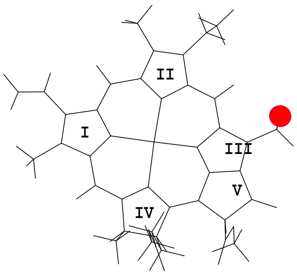

%S= 100

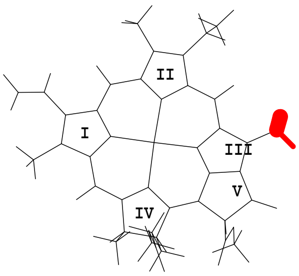

%B= 0

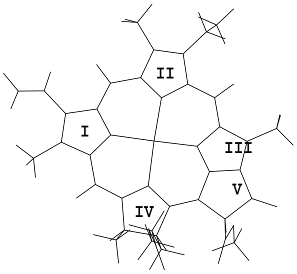

%T= 0

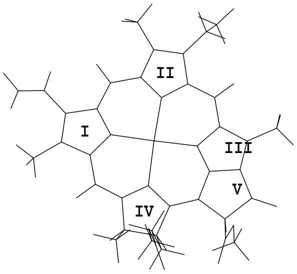

ν= 2923
λ= 0
15N= 0
26Mg= 0

%XY= 79

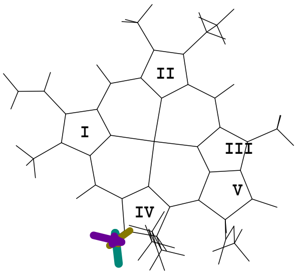

%Z= 21

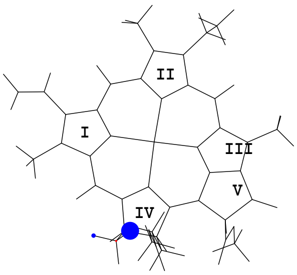

%S= 100

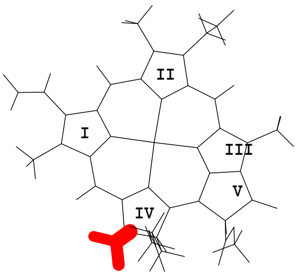

%B= 0

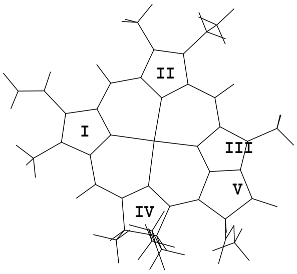

%T= 0

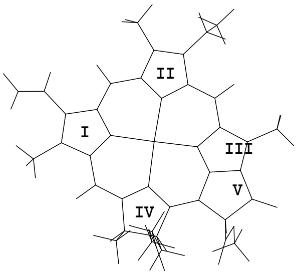

ν=	2925	2925	2930	2932
λ=	0	0	0	0
15N=	0	0	0	0
26Mg=	0	0	0	0
%XY=	83	73	72	85
%Z=	17	27	28	15
%S=	100	99	99	99
%B=	0	0	0	0
%T=	0	0	1	1

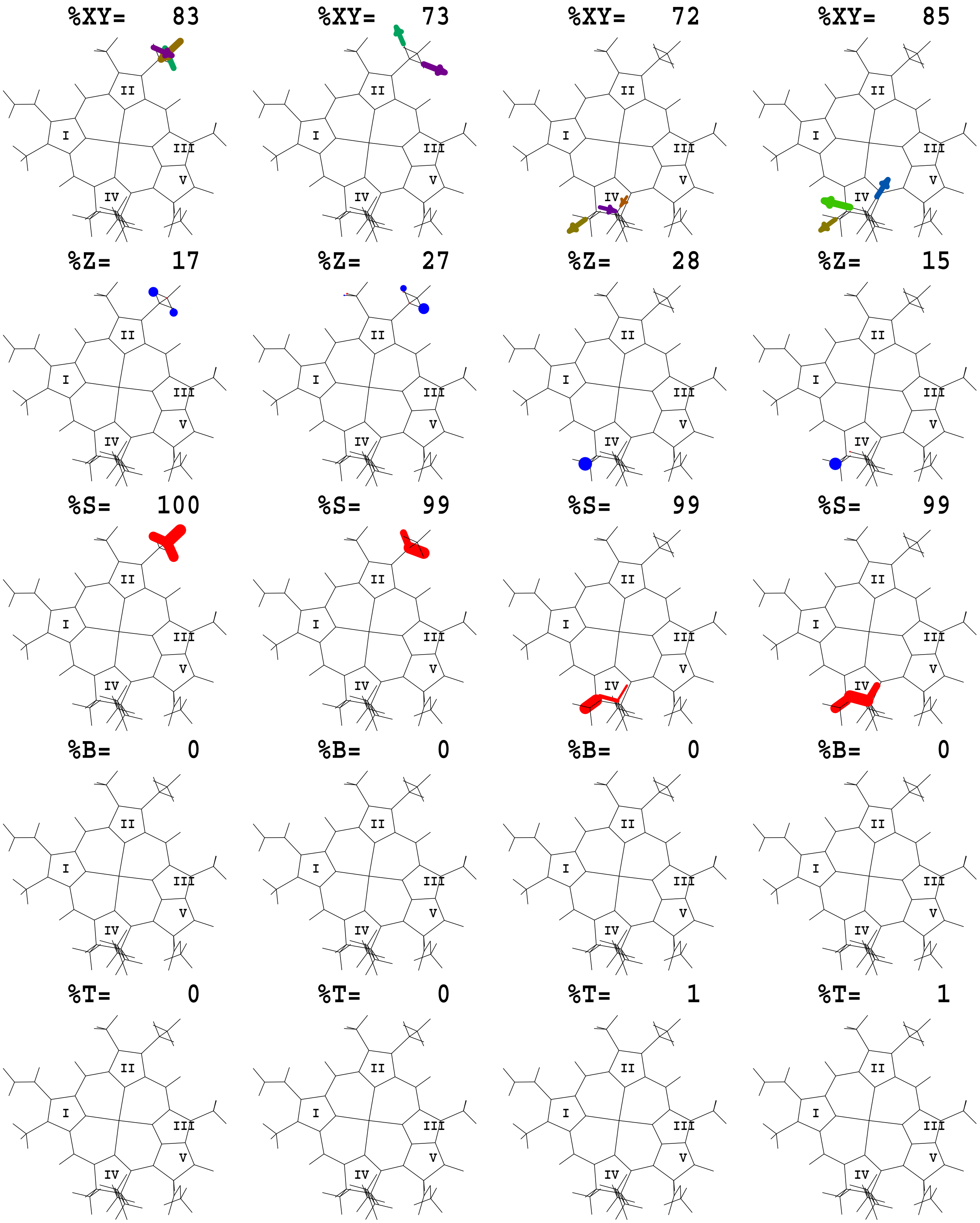

ν=	2946	2947	2949	2957
λ=	0	0	0	0
15N=	0	0	0	0
26Mg=	0	0	0	0
%XY=	96	90	86	79
%Z=		10	14	
%S=	99	100	100	98
%B=	0	0	0	1
%T=	1	0	0	1

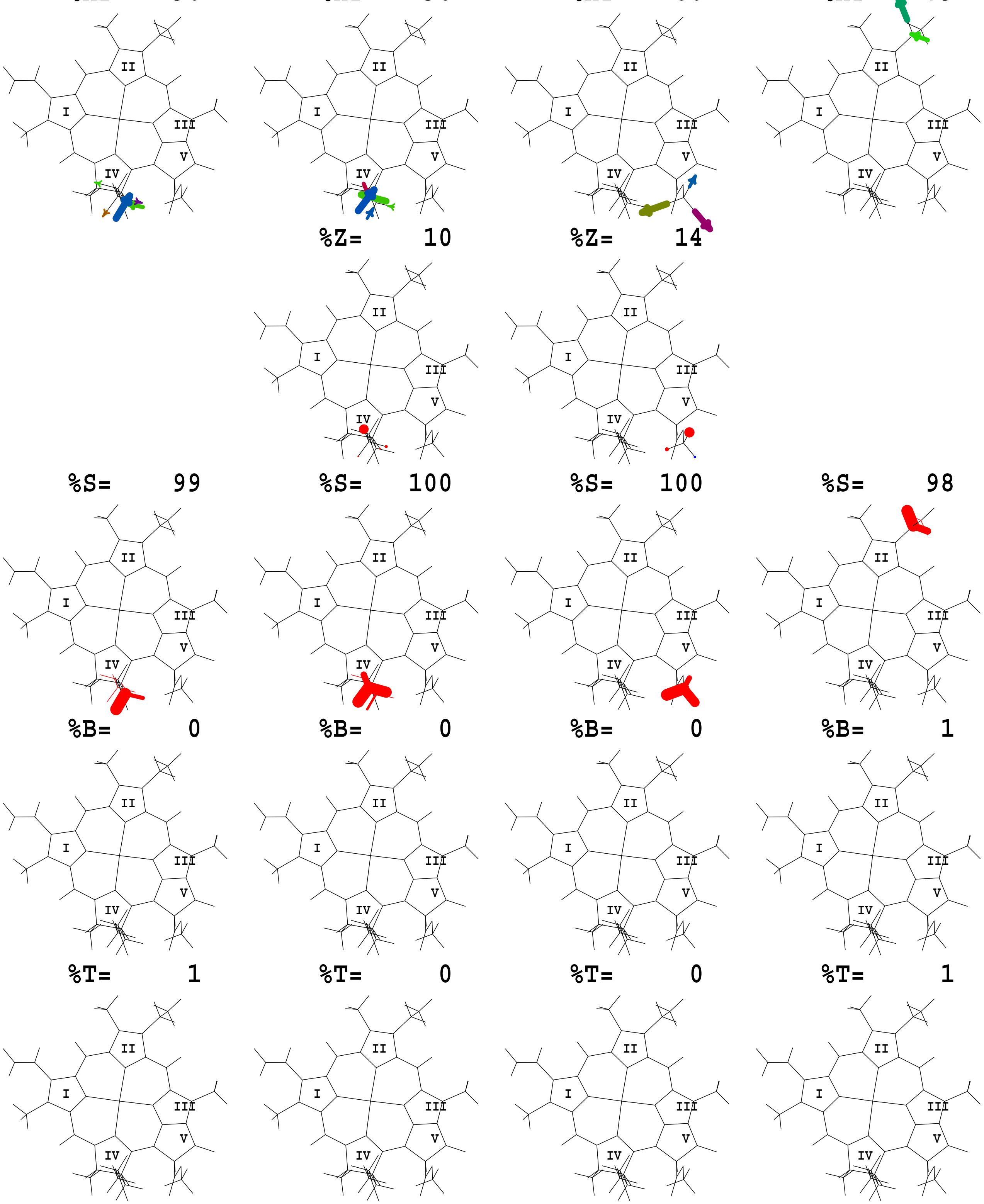

ν= 2964	ν= 2965	ν= 2967	ν= 2971
λ= 0	λ= 0	λ= 0	λ= 0
15N= 0	15N= 0	15N= 0	15N= 0
26Mg= 0	26Mg= 0	26Mg= 0	26Mg= 0

%XY= 74

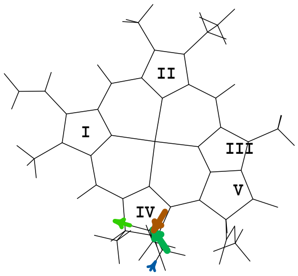

%XY= 80

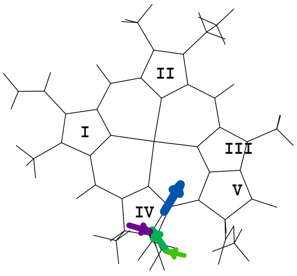

%XY= 34

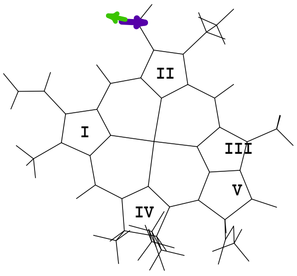

%XY= 37

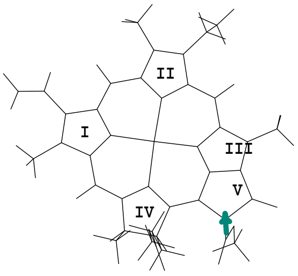

%Z= 26

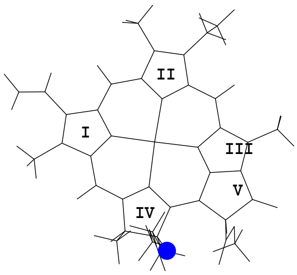

%Z= 20

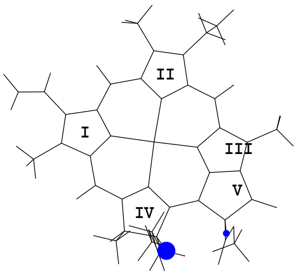

%Z= 66

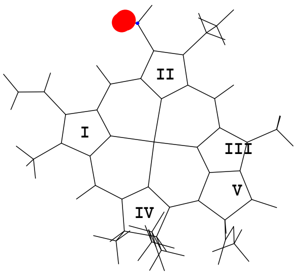

%Z= 63

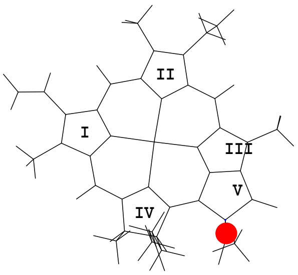

%S= 98

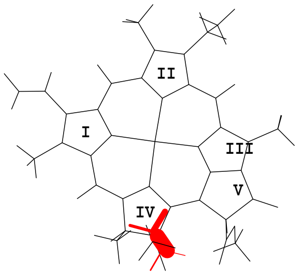

%S= 98

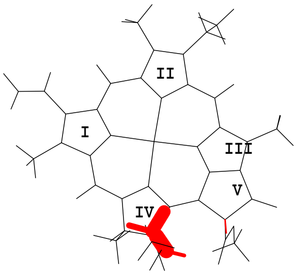

%S= 99

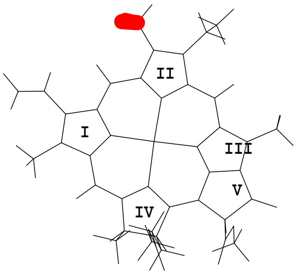

%S= 98

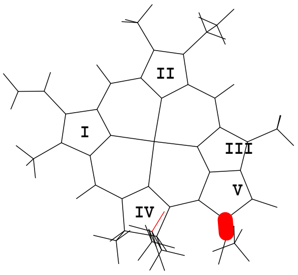

%B= 0

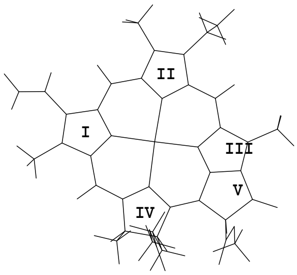

%B= 0

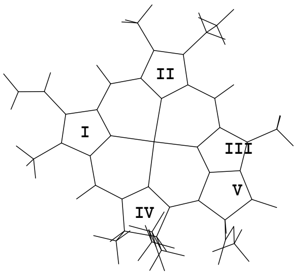

%B= 0

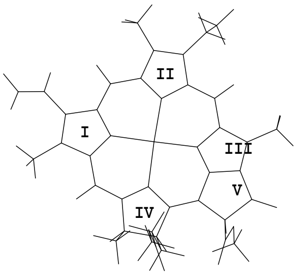

%B= 0

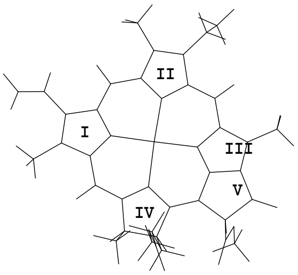

%T= 2

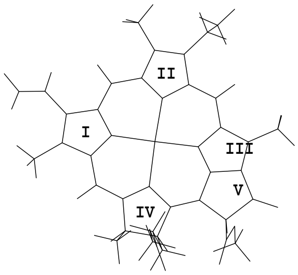

%T= 1

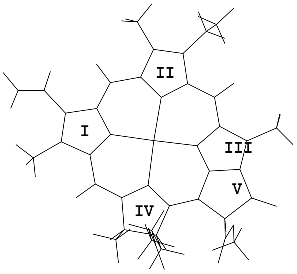

%T= 1

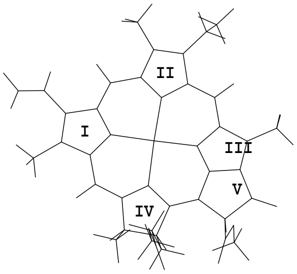

%T= 1

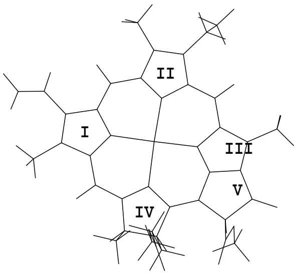

ν= 2973

λ= 0

15N= 0

26Mg= 0

%XY= 33

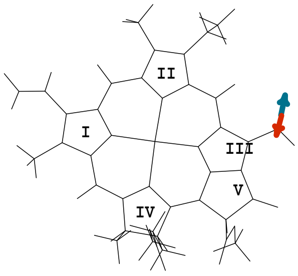

%Z= 67

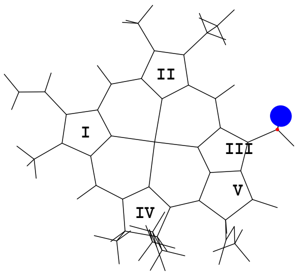

%S= 99

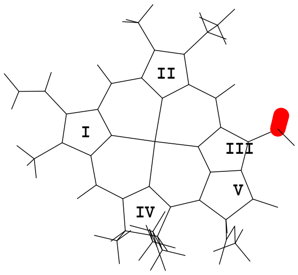

%B= 0

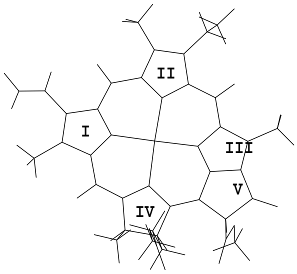

%T= 1

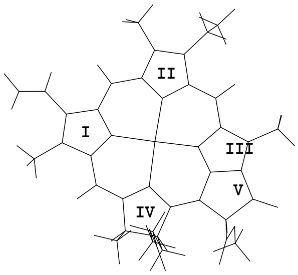

ν= 2974

λ= 0

15N= 0

26Mg= 0

%XY= 50

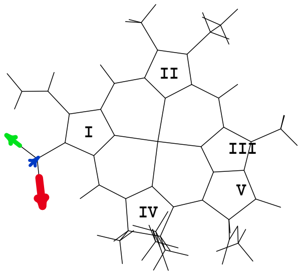

%Z= 50

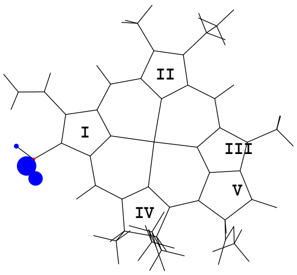

%S= 99

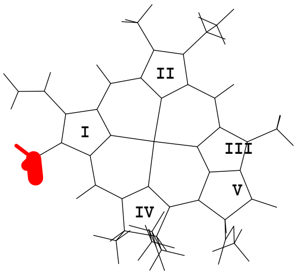

%B= 0

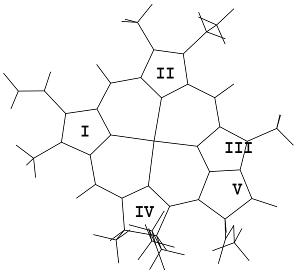

%T= 1

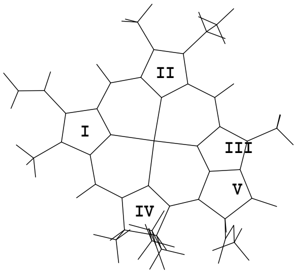

ν= 2991

λ= 0

15N= 0

26Mg= 0

%XY= 98

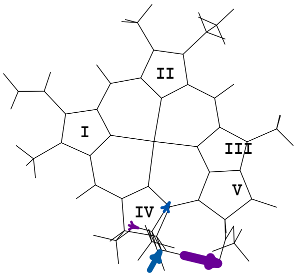

%S= 99

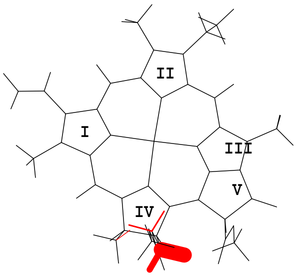

%B= 0

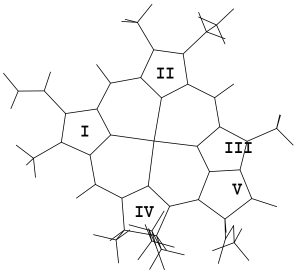

%T= 1

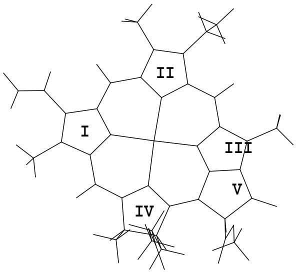

ν= 2993

λ= 0

15N= 0

26Mg= 0

%XY= 74

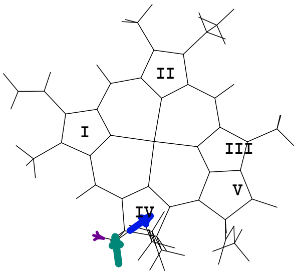

%Z= 26

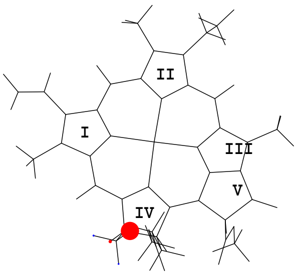

%S= 99

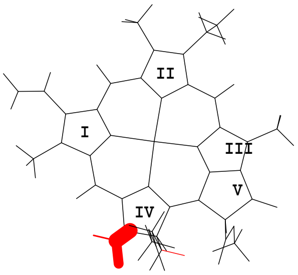

%B= 0

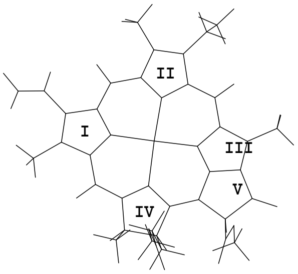

%T= 1

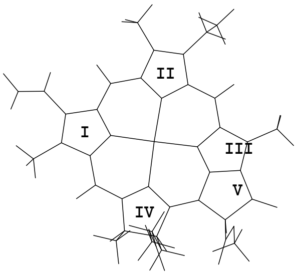

ν=	2995	2998	2998	3001
λ=	0	0	0	0
15N=	0	0	0	0
26Mg=	0	0	0	0

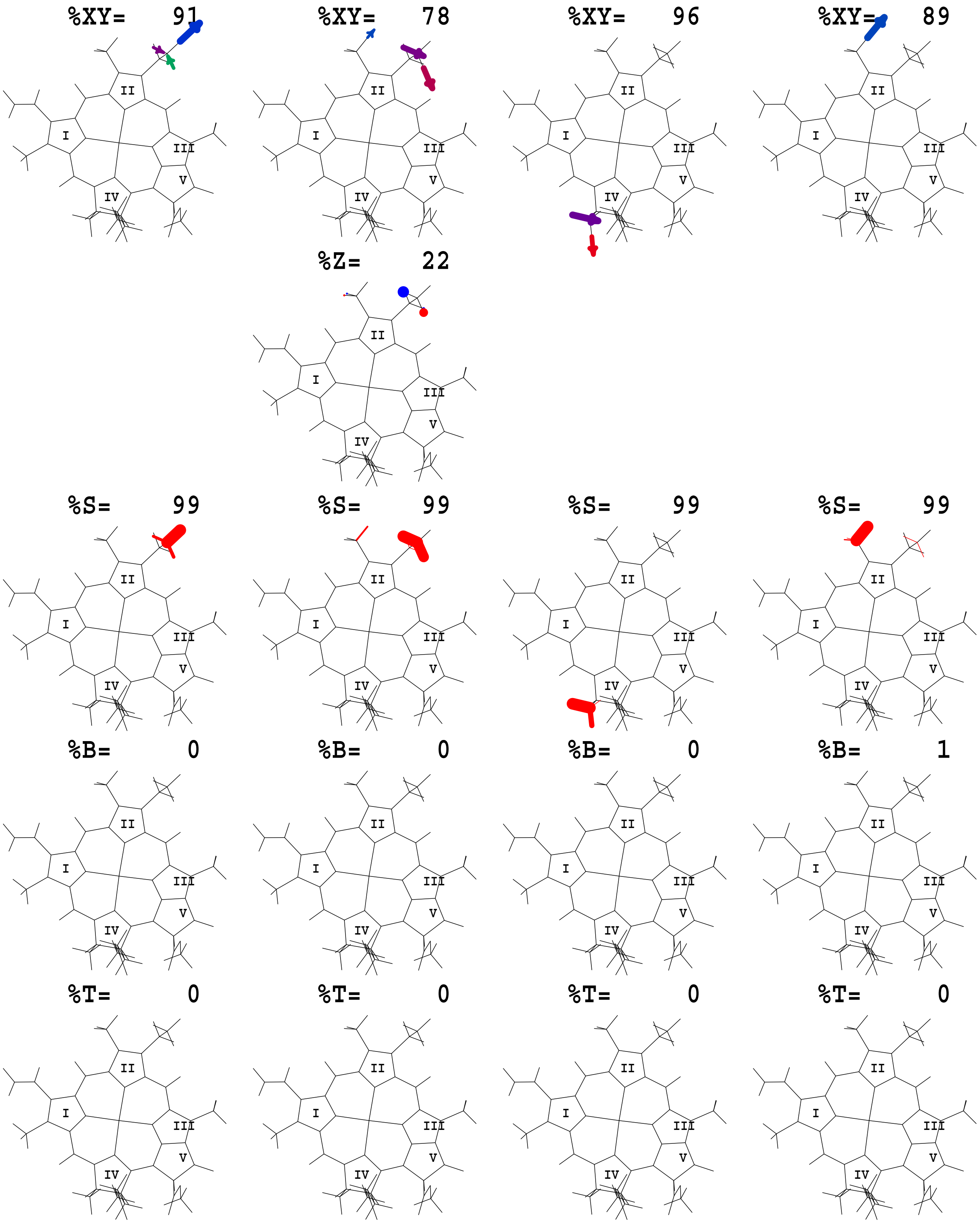

ν= 3005
λ= 0
15N= 0
26Mg= 0

%XY= 81

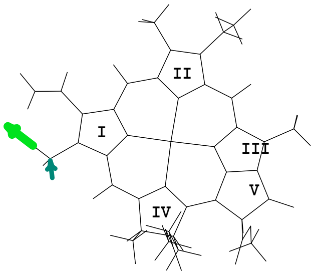

%S= 99

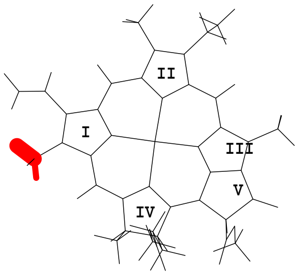

%B= 1

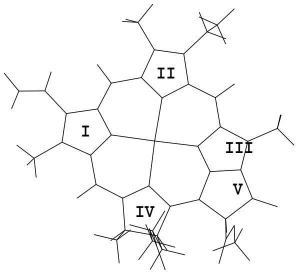

%T= 0

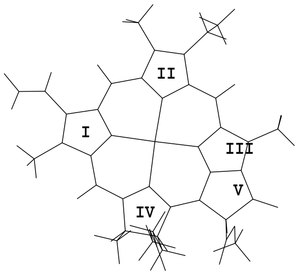

ν= 3018
λ= 0
15N= 0
26Mg= 0

%XY= 91

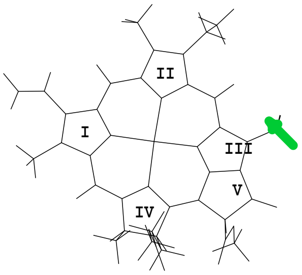

%S= 99

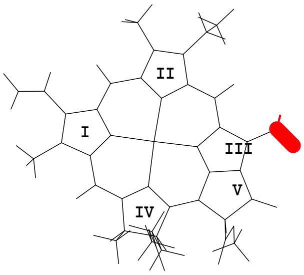

%B= 1

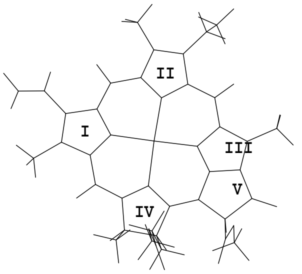

%T= 0

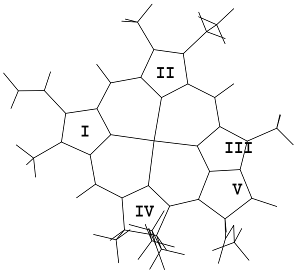

ν= 3021
λ= 0
15N= 0
26Mg= 0

%XY= 99

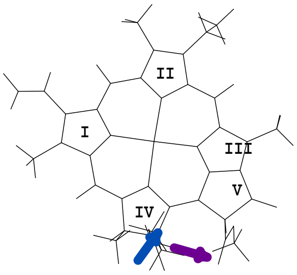

%S= 99

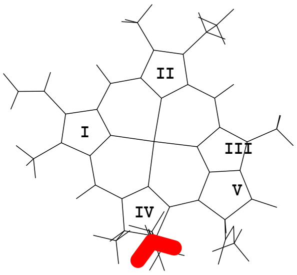

%B= 0

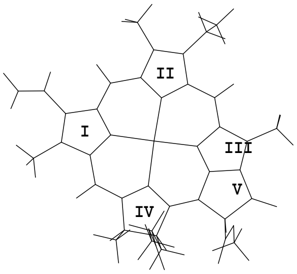

%T= 1

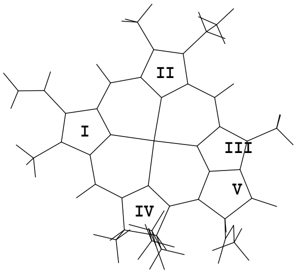

ν= 3024
λ= 0
15N= 0
26Mg= 0

%XY= 97

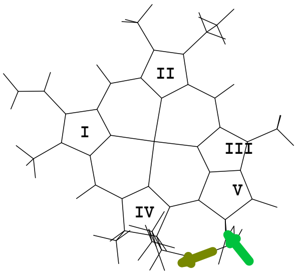

%S= 99

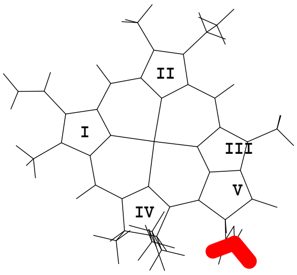

%B= 0

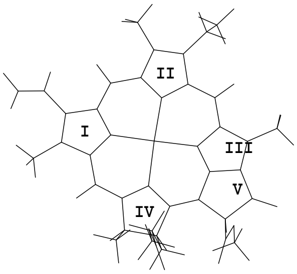

%T= 1

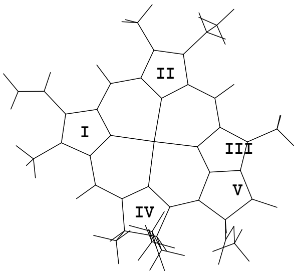

ν= 3026	ν= 3043	ν= 3050	ν= 3054
λ= 0	λ= 0	λ= 0	λ= 0
15N= 0	15N= 0	15N= 0	15N= 0
26Mg= 0	26Mg= 0	26Mg= 0	26Mg= 0

%XY= 76 %XY= 82 %XY= 69 %XY= 59

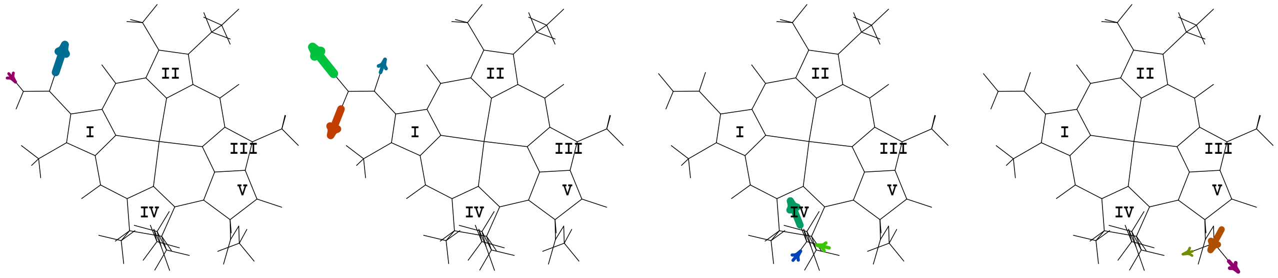

%Z= 24 %Z= 18 %Z= 31 %Z= 41

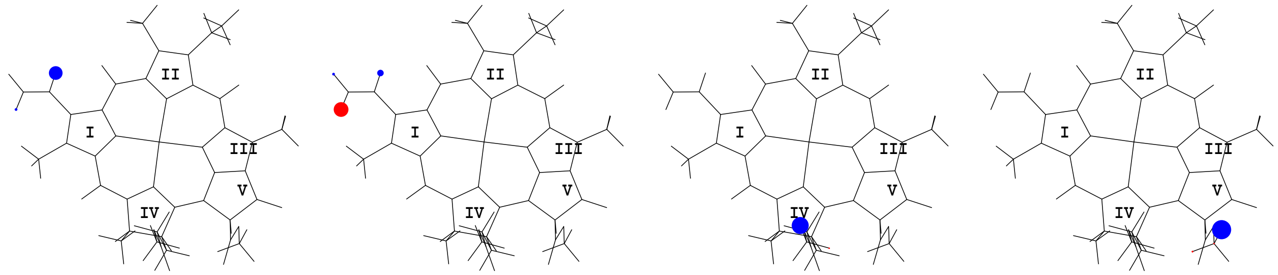

%S= 99 %S= 100 %S= 99 %S= 99

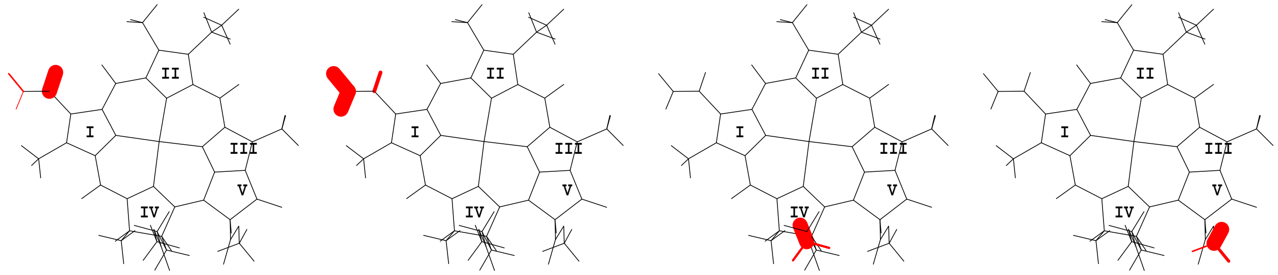

%B= 1 %B= 0 %B= 1 %B= 1

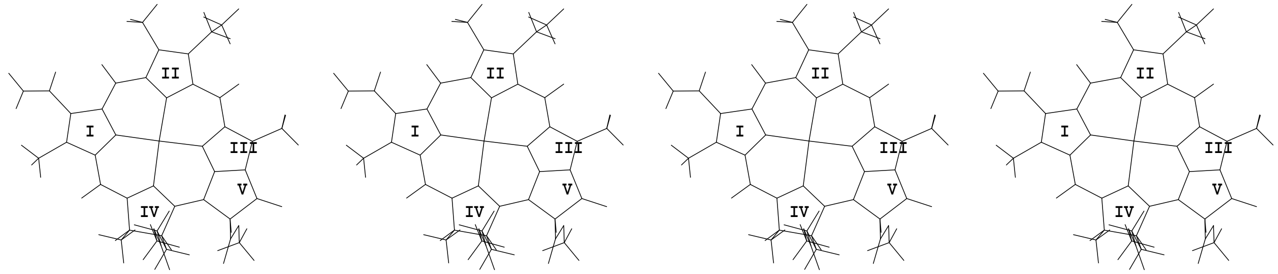

%T= 0 %T= 0 %T= 0 %T= 0

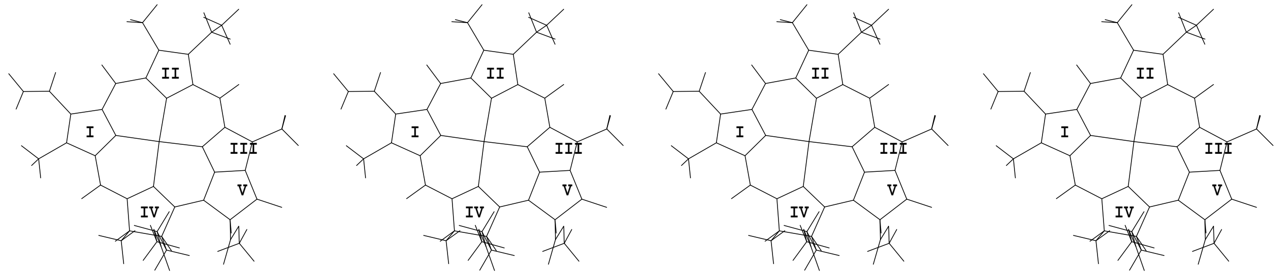

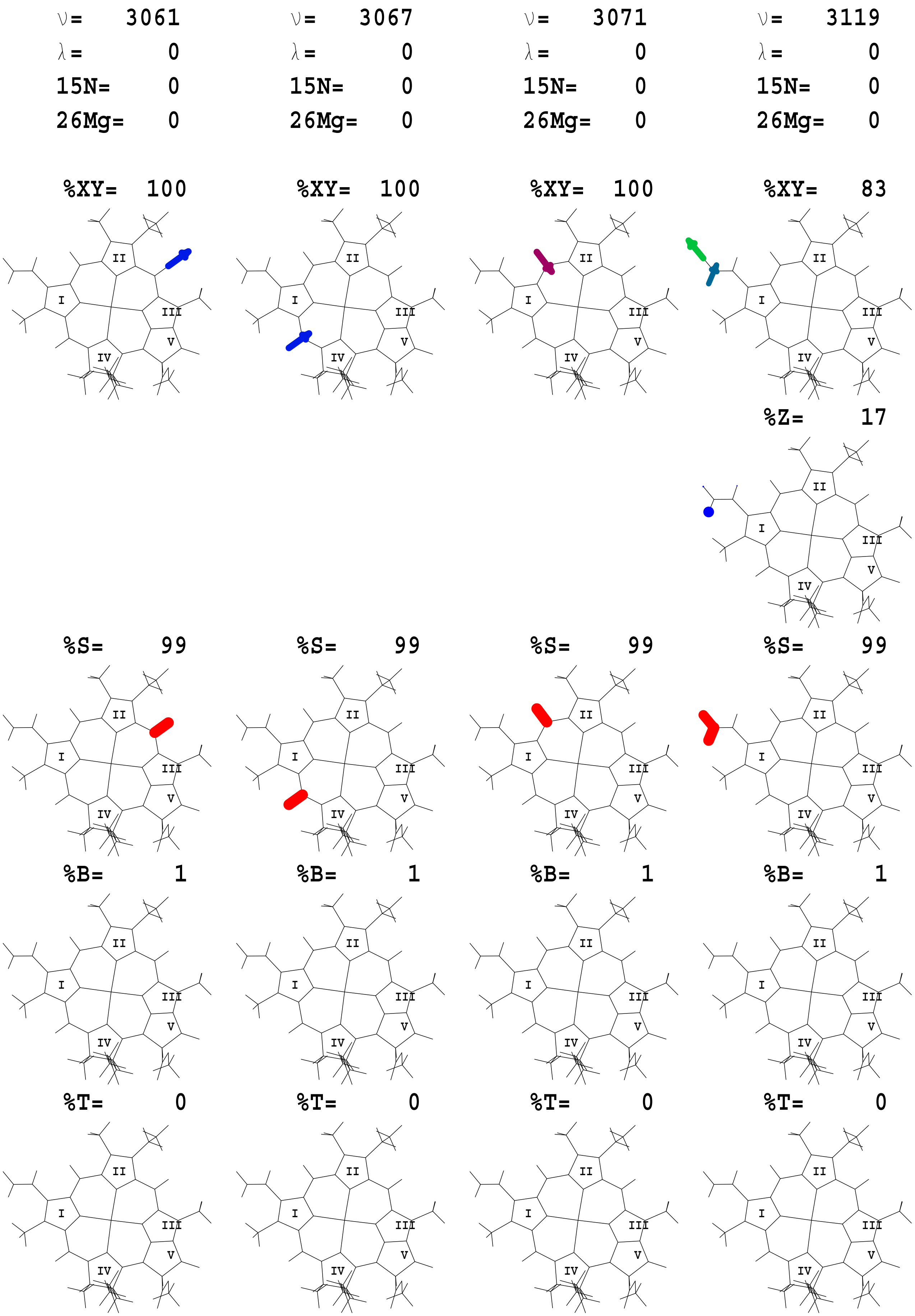

ν= 3061
λ= 0
15N= 0
26Mg= 0
%XY= 100
%S= 99
%B= 1
%T= 0
ν= 3067
λ= 0
15N= 0
26Mg= 0
%XY= 100
%S= 99
%B= 1
%T= 0
ν= 3071
λ= 0
15N= 0
26Mg= 0
%XY= 100
%S= 99
%B= 1
%T= 0
ν= 3119
λ= 0
15N= 0
26Mg= 0
%XY= 83
%Z= 17
%S= 99
%B= 1
%T= 0
